# Amide Bond Formation via the Rearrangement of Nitrile Imines Derived from *N*-2-Nitrophenyl Hydrazonyl Bromides

**DOI:** 10.1021/acs.orglett.1c03993

**Published:** 2021-12-29

**Authors:** Mhairi Boyle, Keith Livingstone, Martyn C. Henry, Jessica M. L. Elwood, J. Daniel Lopez-Fernandez, Craig Jamieson

**Affiliations:** Department of Pure and Applied Chemistry, University of Strathclyde, 295 Cathedral Street, Glasgow G1 1XL, United Kingdom

## Abstract

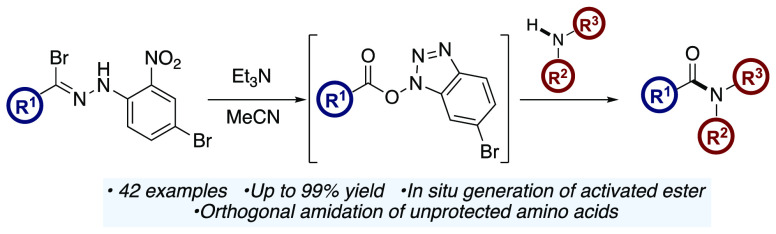

We report how the rearrangement of highly reactive nitrile imines derived from *N*-2-nitrophenyl hydrazonyl bromides can be harnessed for the facile construction of amide bonds. This amidation reaction was found to be widely applicable to the synthesis of primary, secondary, and tertiary amides and was used as the key step in the synthesis of the lipid-lowering agent bezafibrate. The orthogonality and functional group tolerance of this approach was exemplified by the *N*-acylation of unprotected amino acids.

The ability to selectively and efficiently form amide bonds is of paramount importance in organic chemistry. This key linkage comprises the backbone of peptides, proteins, and a range of other biomolecules. Furthermore, amide bonds are abundant motifs in drug discovery, and accordingly, amidation reactions represent a significant proportion of the current synthetic toolkit in medicinal chemistry.^1^ On the basis of this, significant efforts have been dedicated in recent years to the development of novel and efficient amidation reactions.^2^ Recent safety concerns associated with commonly used coupling agents have served to highlight the urgency of developing new amidation processes.^3^ Conventional approaches toward amide bond formation are derived from the generation of an electrophilic carboxylic acid component through the addition of an activating or coupling agent (Scheme 1a). Whereas the widespread applicability of this venerable approach demonstrates its versatility, the reaction invariably suffers from poor atom economy and limited compatibility with other unprotected carboxylic acid moieties. To overcome these limitations, it has been demonstrated that amides can be directly accessed from the corresponding aldehydes by coupling with amines using transition metal catalysis, photoredox catalysis, or organocatalysis under oxidative conditions.^4^ Whereas these methods are an attractive approach to amidation, we were interested in the ability to directly generate an activated carbonyl electrophile through an intramolecular rearrangement from an alternative precursor, derived from simple aldehyde feedstocks, under mild conditions while avoiding the use of transition metals.

**Scheme 1:**
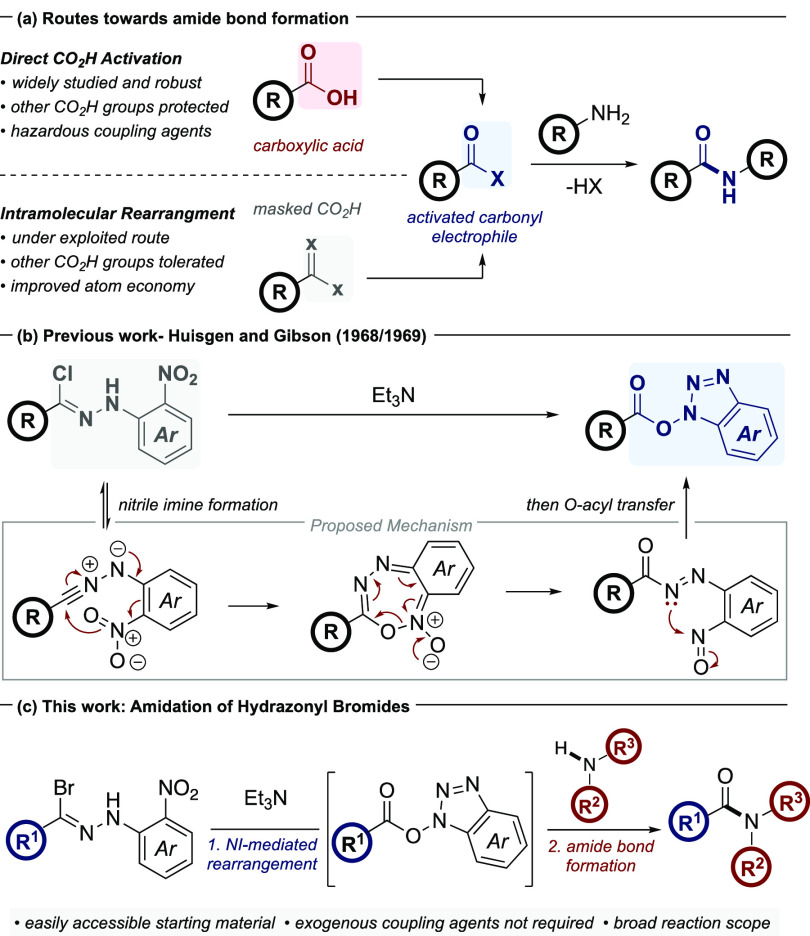
Relevant Antecedence and Proposed Study

During our recent investigations into the applicability of nitrile imines (NIs) in organic synthesis,^5^ we noted a rearrangement of NIs bearing a 2-nitrophenyl motif at the *N*-terminus that was reported independently by Huisgen and Gibson in the late 1960s (Scheme 1b).^6^ After base-induced dehydrohalogenation of the hydrazonyl halide, a 1,7-electrocyclization occurs between the resulting NI and the ancillary *ortho*-nitro group, which results in the formation of a seven-membered intermediate. A similar 1,7-rearrangement involving the *ortho*-nitro group participation has also been invoked in the synthesis of triazines and benzoxazoles from hydrazonyl bromides.^7^

Cycloreversion of the benzannulated seven-membered ring then yields an intermediate nitroso species that undergoes further rearrangement to the *N*-hydroxybenzotriazole followed by acyl group migration resulting in the formation of an *N*-hydroxybenzotriazole activated ester. At the time of these initial reports, the utility of this species was not fully appreciated, especially due to the proclivity to which it may then hydrolyze to the corresponding carboxylic acid.

In 1970, seminal efforts from König and Geiger underlined the utility of *N*-hydroxybenzotriazole (HOBt) as an additive for the synthesis of amide bonds.^8^ Since then, HOBt has found widespread application in organic synthesis, not least in the preparation of peptides.^9^ Often used in combination with a carbodiimide coupling reagent, HOBt is particularly useful in the suppression of side reactions, specifically the rearrangement furnishing the unreactive *N*-acylurea byproduct but also in the avoidance of epimerization through the intermediacy of an oxazalone species.^10^ We reasoned that the application of the unique rearrangement of the aforementioned NI species as an alternative means of accessing the activated ester intermediate could be harnessed to afford a more efficient method of amide bond formation (Scheme 1c). This would potentially avoid the handling and long-term storage of large quantities of HOBt while mitigating the risks associated with the explosive properties of this reagent.^11^ Additionally, the use of our emerging method would obviate the requirement for a carbodiimide coupling reagent and hence improve the overall atom economy of the process. Furthermore, the adoption of a hydrazonyl halide as a masked carbonyl equivalent could enable amide bond formation in the presence of unprotected carboxylic acid moieties, for example, in conjunction with free amino acid derivatives.

The initial development of the proposed approach began with the application of tolyl hydrazonyl bromide **1a** as a NI precursor^12,13^ and benzyl amine as an appropriate amine coupling partner (Table 1). The requisite hydrazonyl bromide substrates were readily accessible from the corresponding aldehyde via acid-mediated condensation with 2-nitrophenylhydrazine and subsequent bromination.^14^ Upon the concurrent addition of triethylamine and benzylamine to **1a**, no amide bond coupling occurred (entry 1). Instead, direct reaction of the amine with the electrophilic pseudoiminium moiety of the NI generated *in situ* resulted in the formation of the *N*-benzylbenzamide phenylhydrazone derivative. To minimize the formation of this byproduct, we employed an activation period of 15 min to allow the base-mediated rearrangement of the NI and the formation of activated ester **2** prior to the addition of benzylamine. This resulted in a 31% conversion to amide **3a** (entry 2). Interestingly, the omission of benzylamine altogether allowed the isolation of activated ester **2** in 52% yield. Encouraged by this result, a solvent screen was performed to improve the conversion and determine the optimal solvent for the transformation. Whereas tetrahydrofuran (THF) resulted in a lower conversion to **3a** of 20%, the use of acetone greatly increased the conversion to 56% (entries 3 and 4). The use of dimethyl carbonate (DMC) was also well tolerated and resulted in 53% conversion to **3a** (entry 5). The conversion was significantly improved upon employing acetonitrile (entry 6). It was also found that performing the reaction at an elevated temperature (50 °C) resulted in the efficient formation of amide **3a**, which was then isolated in 79% yield (entry 7) with a reaction time of <1 h. Control experiments revealed the requirement of the *ortho*-nitro functionality, as no formation of the activated ester was observed in its absence. Lastly, the choice of base was investigated. Performing the reaction with *N*,*N*-diisopropylethylamine (DIPEA) (entry 8) resulted in a comparable conversion to triethylamine; however, the use of inorganic bases resulted in diminished conversion to **3a** (entries 9 and 10).

**Table 1:**
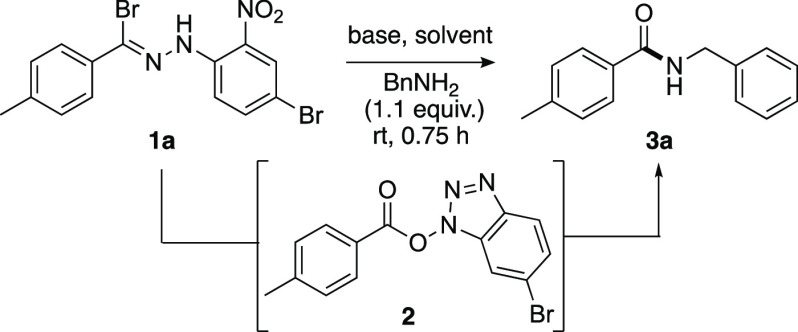
Investigations into Base and Solvent Selection^a^

entry	solvent	base	conversion (%)^b^
1^c^	EtOAc	Et_3_N	0
2	EtOAc	Et_3_N	31
3	THF	Et_3_N	20
4	acetone	Et_3_N	56
5	DMC	Et_3_N	53
6	MeCN	Et_3_N	73
7^d^	MeCN	Et_3_N	79^e^
8	MeCN	DIPEA	72
9	MeCN	K_2_CO_3_	44
10	MeCN	K_3_PO_4_	36

a Reactions performed on a 0.1 mmol scale using 5 equiv of base at a concentration of 0.02 M.

b Conversion determined by HPLC.

c No activation period included.

d Reaction performed at 50 °C.

e Isolated yield.

With the optimized conditions in hand, the scope of the amine was next investigated using tolyl hydrazonyl bromide **1a** (Scheme 2). Under the standard amidation conditions, a variety of primary amines were coupled to give amides **3a**–**3g** in excellent yields. Whereas the presence of an α-methyl group had little effect on the reaction outcome, giving amide **3c** in 75% yield, the increased steric bulk at the α-position of the *gem*-dimethyl-substituted benzylamine nucleophile led to a decrease in the reaction efficiency. Nevertheless, the sterically hindered amide **3d** was isolated in 31% yield. The scope was then extended to include secondary amines, which were efficiently converted to the corresponding amides **3h**–**3m** in 42–89% yield. Additionally, cyclic secondary amines piperidine and morpholine were also competent substrates for the amidation procedure and gave **3n** and **3o** in 63 and 74% yield, respectively. Access to primary amide **3p** was achieved using aqueous ammonia in 64% yield. Aniline was found to undergo *N*-acylation under the standard conditions and gave **3q**, albeit in only 38% yield. The reduced yield is consistent with the less nucleophilic nature of aniline derivatives as compared with other nitrogen nucleophiles. This observation was reinforced by the fact that coupling with electron-rich *p*-anisidine gave amide **3r** in 62% yield, whereas the very electron-deficient 4-nitro analogue failed to form the desired product. Employing benzylamine as the *N*-nucleophile, the scope of the transformation with a range of hydrazonyl bromides was then investigated (Scheme 2). Hydrazonyl bromide substrates featuring electron-deficient and electron-rich aryl groups and para and ortho substituents, were well tolerated in the rearrangement/amidation process and allowed the synthesis of amide analogues **4a**–**4g** in 58–88% yield. The application of hydrazonyl bromides derived from aliphatic aldehydes gave the corresponding amides **4h**–**4k** in 66–94% yield. Moreover, heterocyclic substrates were successfully utilized in the amidation reaction and afforded tetrahydrofuran analogue **4l** and isoxazole-derived lysophosphatidic acid (LPA) antagonist^15^**4m** in 67 and 68% yield, respectively. Attempts to use α-amino acids were unfortunately not successful due to the incompatibility of the corresponding aldehydes with the conditions employed for hydrazonyl bromide formation. Other pharmaceutically relevant targets and building blocks were also synthesized using this methodology. For example, moclobemide (**4n**), a reversible monoamine oxidase inhibitor,^16^ was afforded in 51% yield, whereas compound **4o**, a precursor in the synthesis of sodium channel blocker procainamide,^17^ was isolated in 21% yield. Employing l-valine methyl ester in our manifold gave amide **4p**, a key intermediate in the synthesis of valsartan,^18^ in 75% yield.

**Scheme 2:**
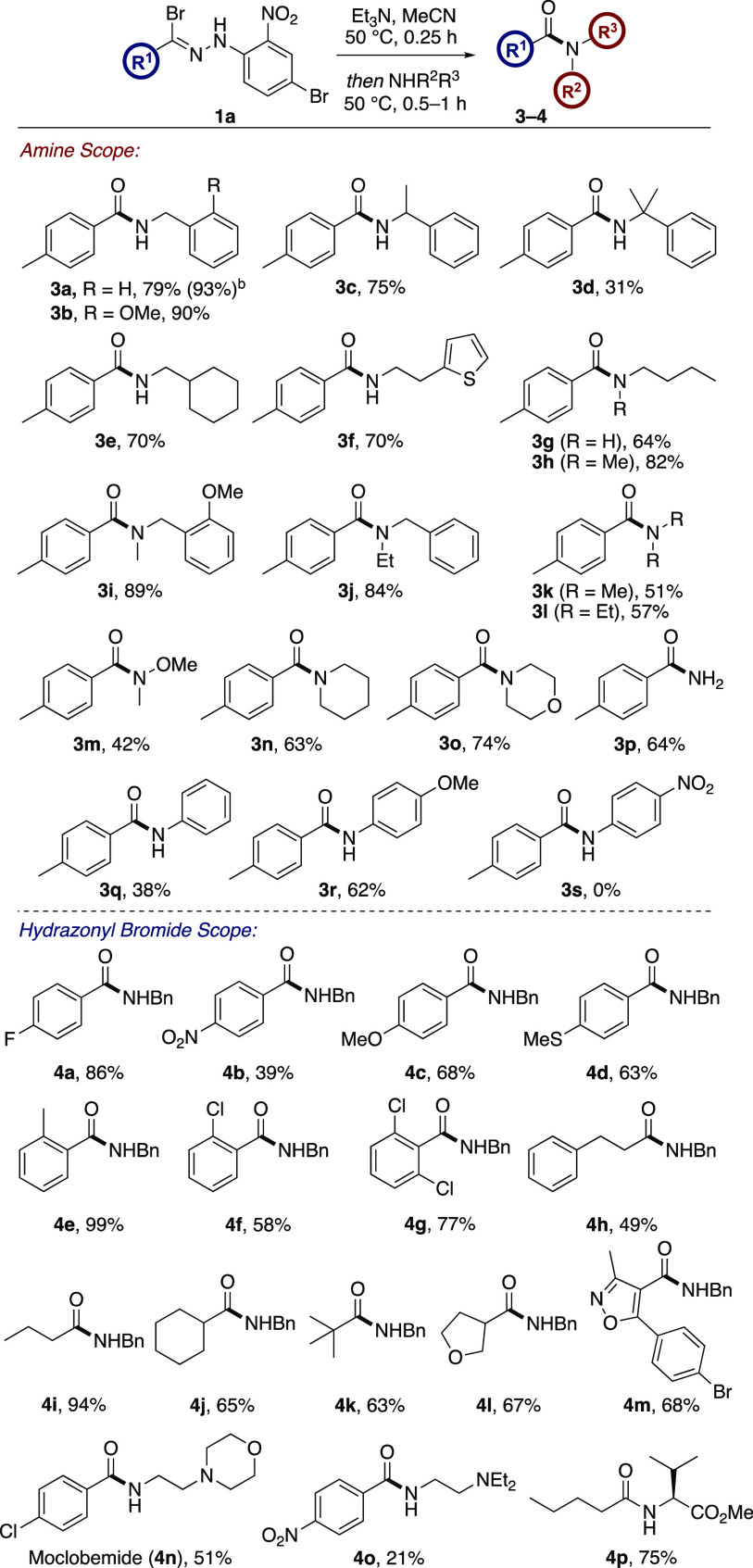
Amidation Substrate Scope Isolated yields. Reaction was performed on a 1.8 mmol scale.

The versatility of the methodology was further elaborated with the synthesis bezafibrate (**5**), a marketed fibrate drug used in the treatment of hyperlipidaemia (Scheme 3).^19^ 4-Chlorophenylhydrazonyl bromide **1m** was subjected to our optimized conditions with tyramine to afford amide **4q** in 72% yield. Alkylation of the phenol moiety with isopropyl 2-bromo-2-methylpropanoate followed by ester hydrolysis under basic conditions completed the three-step synthesis of bezafibrate (**5**).

**Scheme 3:**
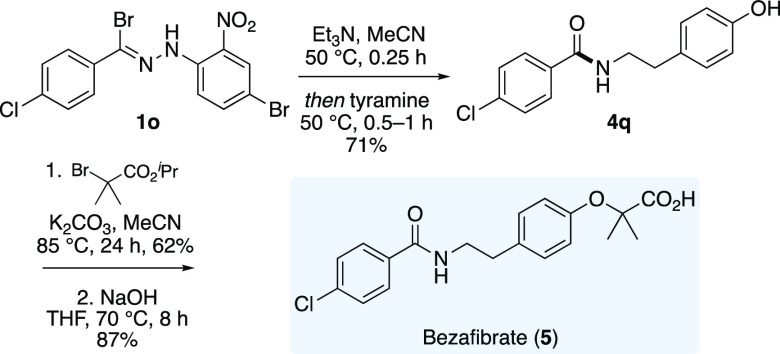
Synthesis of Bezafibrate (**5**)

In the final phase of our study, we sought to apply the amidation methodology to the selective *N*-acylation of proteinogenic amino acids (Scheme 4). Under the optimized protocol, d,l-alanine, d,l-phenylalanine, d,l-valine, and l-isoleucine underwent successful amide coupling with hydrazonyl bromide **1a** in the presence of the unprotected carboxylic acid functionality and led to the isolation of *N*-acyl derivatives **6a**–**6d** in 44–68% yield. The secondary amino acid l-proline was effective in our reaction manifold and gave the coupled adduct **6e** in 50% yield, whereas l-glutamic acid, bearing two free carboxylic acid groups, was tolerated and gave analogue **6f** in 36% yield. When enantiopure amino acids were employed as *N*-nucleophiles in our methodology, no degradation of the stereochemical integrity was observed, indicating that no epimerization of **6d**–**6f** had occurred during the process.^20,21^ The addition of amino acids as a solution in water was also tolerated under the reaction conditions, further demonstrating the robustness of this protocol.

**Scheme 4:**
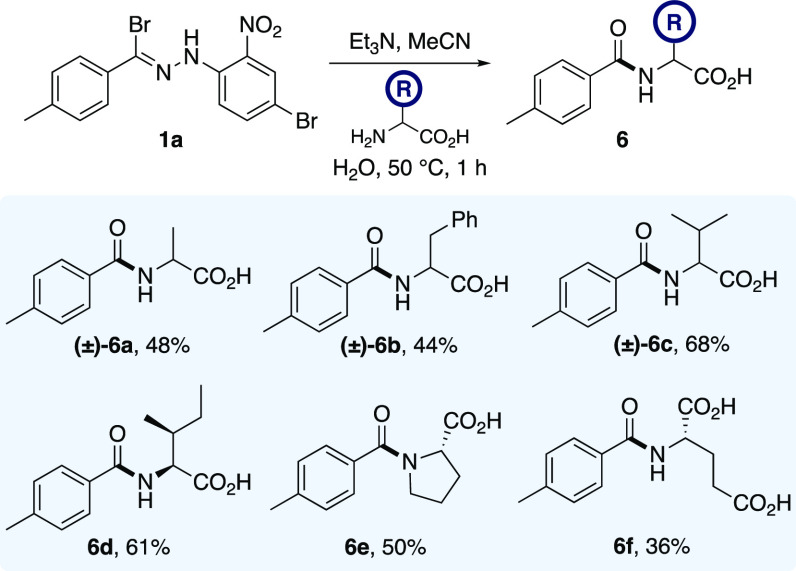
*N*-Acylation of Unprotected Amino Acids

In summary, we have utilized an underexploited rearrangement of *N*-2-nitrophenyl-hydrazonyl-bromide-derived NIs for the mild and rapid formation of amide bonds. The *in situ* generation of *N*-hydroxybenzotriazole activated ester **2** avoided the use of external activating agents and their associated safety issues, particularly in the case of uronium-based coupling reagents.^3a^ Although an HOBt derivative is still produced in small quantities as a byproduct during the process, our method obviates the requirement to transport and store large quantities of this potentially explosive compound. It has been demonstrated that this transformation is tolerant of a wide range of aromatic and aliphatic hydrazonyl bromides with differing electronic properties and a range of primary and secondary amines. In addition to its use as the key step in the short synthesis of bezafibrate, this methodology was applied to the *N*-acylation of natural amino acids in acetonitrile and water. The facile and orthogonal amidation of unprotected proteinogenic amino acids under these conditions could have potential applications in the selective labeling of proteins or other important biomolecules. Work is currently under way within our laboratory to fully explore the transformation in this context. The further development of this formal oxidative coupling process will encompass the employment of chiral aldehyde derivatives as hydrazonyl bromide precursors to facilitate the synthesis of enantioenriched substrates and extension to natural product synthesis.

## Supporting Information Available

Experimental procedures, characterization data, and NMR spectra of all compounds (PDF)
