# Supplementary material for: Amide Bond Formation via the Rearrangement of Nitrile Imines Derived from N-2-Nitrophenyl Hydrazonyl Bromides
Source: Org Lett. 2021 Dec 29;24(1):334–8. doi: 10.1021/acs.orglett.1c03993 (PMC8762704; doi:10.1021/acs.orglett.1c03993)

Supporting Information for:

Amide Bond Formation *via* the Rearrangement of Nitrile Imines Derived from *N*-2-Nitrophenyl Hydrazonyl Bromides

Mhairi Boyle, Keith Livingstone, Martyn C. Henry, Jessica M. L. Elwood, J. Daniel Lopez-Fernandez and Craig Jamieson*

Department of Pure and Applied Chemistry, University of Strathclyde, Glasgow G1 1XL, United Kingdom. Email: craig.jamieson@strath.ac.uk

Table of Contents

1. General Experimental
2. HPLC Assay and Optimization of Reaction Conditions
3. Experimental Procedures and Spectroscopic Data for all Compounds
4. References
5. ^{1}H and ^{13}C NMR Spectra for all Compounds

1. General Experimental

All reagents and starting materials were obtained from commercial sources and used as received without further purification, unless otherwise stated. Acetone, dichloromethane, ethyl acetate, methanol, petroleum ether 40–60 °C, and tetrahydrofuran were used as obtained from suppliers without further purification. All dry solvents were purified using a PureSolv SPS-400-5 Solvent Purification System.

All reactions were performed using round-bottom flasks or microwave vials of appropriate volume. Reactions were carried out at elevated temperatures using a temperature regulated hotplate/stirrer and DrySyn block with a contact thermometer. Room temperature generally refers to ~ 20 °C. Reactions requiring a reduced temperature were performed using an ice bath (0 °C) or a dry ice/acetone slurry (–78 °C) and a temperature probe unless otherwise stated. Brine refers to a saturated aqueous solution of sodium chloride.

Reactions were monitored by thin layer chromatography (TLC) using Merck silica gel 60 covered aluminium backed plated F254. TLC plates were visualised under UV light and staining using potassium permanganate solution, acidic ethanolic anisaldehyde solution, vanillin or ninhydrin. Flash column chromatography was performed with silica gel 60 (40−63 µm). Reverse-phase HPLC purification of *N*-acylated amino acids was conducted using a Gilson preparative HPLC system of 322 pumps coupled to a 151 UV/Vis 163 spectrometer, 234 Autoinjector and a GX-271 liquid handler using an Agilent Zorbax SB-C18 column (21.2 x 150 mm, 5 µm packing diameter) at room temperature. Purifications were performed using gradient methods ranging from 5–95 % MeCN in H_2O over 30 minutes at a flow rate of 15 mL/min, with a 0.1% TFA modifier and UV monitoring at 214 nm. Analysis was conducted using Gilson Trilution v2.0 software.

Infrared spectra were recorded on a FTIR spectrometer; wavenumbers are indicated in cm^{-1}. NMR spectra were recorded using dilute solutions in deuterated solvent on a Bruker NMR spectrometers at either 400 or 500 MHz using the deuterated solvent as the internal deuterium lock. ^1H chemical shift data are given as units δ relative to the residual protic solvent where δ ($CDCl_3$) = 7.26 ppm, (D_6-DMSO) = 2.50 ppm and (CD_3OD) = 3.35 ppm. ^1H signals are described as singlets (s), doublets (d), triplets (t), quartets (q), multiplets (m), broad (br), app (apparent) or a combination of these. ^{13}C chemical shift data were recorded at were recorded on Bruker NMR spectrometers at either 101 or 126 MHz and are given in units δ relative to the solvent where δ (CDCl3) = 77.16 ppm, (D_6-DMSO) = 39.5 ppm and (CD_3OD) = 49.0 ppm.

High-resolution mass spectra were recorded using a Thermoscientific Exactive Plus equipped with a Vanquish LC. Low-resolution mass spectra were obtained using an Agilent

Technologies 1200 series instrument with a 6130 single quadropole LC/MS using a poroshell EC-C18 column. Analysis was performed using a gradient method, eluting with 5–95% MeCN (containing 5nM ammonium acetate)/H_2O (containing 5nM ammonium acetate) over 18 minutes at a flow rate of 1 mL/min, with UV monitoring at 254 nm.

Melting points were recorded using a Gallenkamp melting point apparatus. Where no solvent is indicated, the solids obtained from the described procedure were melting directly without recrystallisation.

Optical rotation values were determined as solutions in methanol irradiating with the sodium D line (λ = 589 nm) using a PerkinElmer 341 polarimeter. $[\alpha]_D$ values are given in units 10^{-1} deg cm^2 g^{-1}.

2. HPLC Assay and Optimization of Reaction Conditions

For reactions using an internal standard, prior HPLC calibration was carried out using samples containing varying molarities of product and caffeine, allowing calculation of the response factor by substituting values into the following equation:

$$Response\ Factor = \frac{\left(\frac{Area}{Molarity}\right)Product}{\left(\frac{Area}{Molarity}\right)Standard}$$

Screening reactions were carried out using a known molarity of caffeine as the internal standard indicated in the relevant general experimental procedures. Unknown molarities of product were Calcd by rearranging the above equation, using the average value for the response factor as determined during calibration. Conversion to product was Calcd as a percentage of the theoretical molarity for the reaction.

Samples for HPLC analysis were prepared by diluting a 100 µL aliquot from the reaction mixture and a 100 µL aliquot of a 1 mM caffeine standard to 1 mL with MeCN.

General Optimised Conditions for HPLC assay

Hydrazonyl bromide (0.1 mmol scale) was dissolved in appropriate solvent (5 mL) and base (5 equiv.) was added and left to stir for 15 minutes before the addition of benzyl amine (1.1 equiv.). Samples (100 µL) were taken from the reaction mixture after 5 and 30 minutes and diluted with 1 mM solution of caffeine (100 µL) and MeCN (800 µL) before analysis by HPLC.

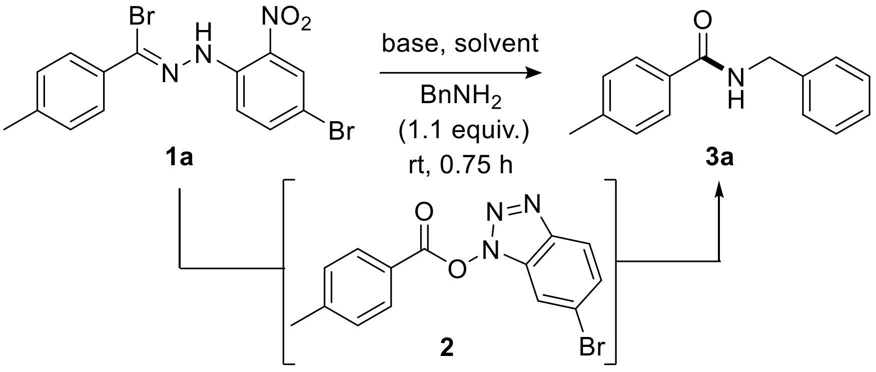

Entry	Solvent	Base	Conversion after 5 mins (%)[a]	Conversion after 30 mins (%)[a]
1[b]	EtOAc	Et_3N	0	0
2	EtOAc	Et_3N	34.7	31.4
3	Acetone	Et_3N	55.7	55.9
4	DMC	Et_3N	52.7	53.0
5	Heptanol	Et_3N	6.60	5.80
6	DMF	Et_3N	2.80	2.40
7	THF	Et_3N	20.5	20.0
8	2-MeTHF	Et_3N	21.1	21.3
9	MeCN	Et_3N	71.6	72.7
10	MeCN	K_3PO_4	35.0	36.1
11	MeCN	DIPEA	71.3	71.6
12	MeCN	K_2CO_3	44.8	44.2
13	MeCN	Cs_2CO_3	11.6	16.7
14[c]	MeCN	Et_3N	48.8	47.2
15[d]	MeCN	Et_3N	44.0	44.2
16[e]	MeCN	Et_3N	36.0[f]	-
17[e, g]	MeCN	Et_3N	80.0[f]	-
18[e, h]	MeCN	Et_3N	51.0[f]	-

[a] Conversion determined by HPLC with reference to caffeine as the internal standard. [b] No activation period included. [c] Reaction performed using 3 equiv. of Et_3N. [d] Reaction performed using 10 equiv. of Et_3N. [e] Reaction performed on 0.5 mmol scale. [f] Isolated yield. [g] Reaction performed at 50 °C. [h] Reaction performed at 80 °C.

3. Experimental Procedures and Spectroscopic Data for all Compounds

General Procedure A: Preparation of Hydrazonyl Bromides

To a stirred solution of 2-nitrophenylhydrazine (1 equiv.) in glacial acetic acid (25 mL/mmol) was slowly added the appropriate aldehyde (1.1 equiv.). The reaction mixture was heated to 110 °C and then a solution of elemental bromine (2 equiv.) in glacial acetic acid (5 mL) was added. The reaction mixture was stirred for 0.5 h at 110 °C, cooled to room temperature and quenched by the addition of water. The resulting precipitate was obtained by filtration, dried under reduced pressure and used without further purification unless otherwise stated. In the instances where the product would fail to precipitate, the mixture was extracted with CH_2Cl_2 (3 × 50 mL) and the combined organic extracts were washed with saturated aqueous $NaHCO_3$ and brine then dried over $MgSO_4$, filtered and concentrated *in vacuo*. The resulting residue was then purified by flash column chromatography and/or recrystallisation to afford the hydrazonyl bromide product.

Due to the instability of the hydrazonyl bromides when subjected to HRMS analysis, in the majority of cases, the HRMS data for the hydrolyzed hydrazide product is reported.

(*Z*)-*N*-(4-Bromo-2-nitrophenyl)-4-methylbenzohydrazonoyl bromide (1a)

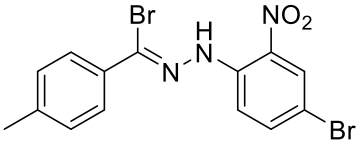

The reaction was performed as described in general procedure A using 2-nitrophenyl hydrazine (3.06 g, 20.0 mmol), 4-methylbenzaldehyde (2.59 mL, 22.0 mmol) and elemental bromine (2.06 mL, 40.2 mmol) to afford (*Z*)-*N*-(4-bromo-2-nitrophenyl)-4-methylbenzohydrazonoyl bromide (**1a**) (4.80 g, 58%) as a red solid. R*f* = 0.71 (pet. ether/Et_2O 9:1); m.p. 135–137 °C; IR vmax (solid) 3291, 3098, 2917, 1616, 1562, 1493, 1143, 1221, 1143, 1070, 882, 813 cm^{-1}; ^1H NMR (500 MHz, $CDCl_3$) 11.32 (s, 1H), 8.37 (d, *J* = 2.2 Hz, 1H), 7.84 (d, *J* = 8.2 Hz, 2H), 7.82 (d, *J* = 9.1 Hz, 1H), 7.65 (dd *J* = 9.1, 2.2 Hz, 1H), 7.24 (d, *J* = 8.2 Hz 2H), 2.42 (s, 3H); ^{13}C NMR (101 MHz, $CDCl_3$) δ 141.4, 139.6, 139.2, 132.5, 129.8, 129.5 (2 × CH), 128.5 (2 × CH), 127.5, 127.3, 118.8, 111.1, 21.5; HRMS (ESI) m/z: $[M – H]^–$ (Hydrazide–H) Calcd for $C_{14}H_{11}BrN_3O_3$ 347.9989; Found 347.9993.

(*Z*)-*N*-(4-Bromo-2-nitrophenyl)-4-fluorobenzohydrazonoyl bromide (1b)

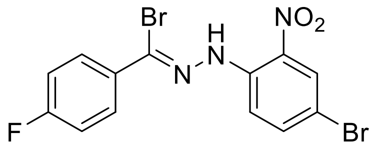

The reaction was performed as described in general procedure A using 2-nitrophenyl hydrazine (100 mg, 653 µmol), 4-fluorobenzaldehyde (76.7 µL, 715 µmol) and elemental bromine (66.7 µL, 1.30 mmol) to afford (*Z*)-*N*-(4-bromo-2-nitrophenyl)-4-fluorobenzohydrazonoyl bromide (**1b**) (190 mg, 70%) as an orange solid. R*f* = 0.74 (pet. ether/EtOAc 9:1); m.p. 176–178 °C; IR vmax (solid) 3278, 3100, 1612, 1562, 1521, 1498, 1420, 1342, 1303, 1269, 1230, 1148, 1137, 1106, 931, 884, 839, 824, 730, 677 cm^{-1}; ^{1}H NMR (500 MHz, $CDCl_3$) δ 11.32 (s, 1H), 8.39 (d, *J* = 2.3 Hz, 1H), 7.98–7.93 (m, 2H), 7.80 (d, *J* = 9.1 Hz, 1H), 7.66 (dd, *J* = 9.1, 2.3 Hz, 1H), 7.17–7.11 (m, 2H); ^{13}C NMR (126 MHz, $CDCl_3$) δ 164.4 (d, $J_{C\text{-}F}$ = 252.4 Hz), 139.4, 139.2, 131.4 (d, $J_{C\text{-}F}$ = 3.7 Hz), 130.9 (d, $J_{C\text{-}F}$ = 8.6 Hz), 128.5, 118.6, 115.9 (d, $J_{C\text{-}F}$ = 22.2 Hz), 111.4; ^{19}F NMR (471 MHz, $CDCl_3$) δ –109.27 (s); HRMS (ESI) m/z: $[M – H]^-$ (Hydrazide–H) Calcd for $C_{13}H_8BrFN_3O_3$ 351.9739; Found 351.9742.

(*Z*)-*N*-(4-Bromo-2-nitrophenyl)-4-nitrobenzohydrazonoyl bromide (1c)

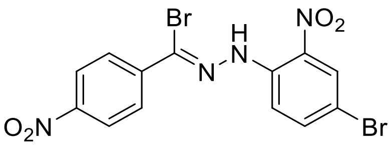

The reaction was performed as described in general procedure A using 2-nitrophenyl hydrazine (76.5 mg, 500 µmol), 4-nitrobenzaldehyde (83.0 mg, 549 µmol) and elemental bromine (51.2 µL, 1.00 mmol) to afford (*Z*)-*N*-(4-bromo-2-nitrophenyl)-4-nitrobenzohydrazonoyl bromide (**1c**) (127 mg, 57%) as a red solid. R*f* = 0.35 (pet. Ether/Et_2O 9:1); m.p. 234–236 °C; IR vmax (solid) 3258, 3088, 1504, 1485, 1338, 1325, 1269, 1143, 1109, 864, 854. 832, 731, 690 cm^{-1}; ^{1}H NMR (500 MHz, $CDCl_3$) δ 11.48 (s, 1H), 8.41 (d, *J* = 2.2 Hz, 1H), 8.30 (d, *J* = 8.9 Hz, 2H), 8.13 (d, *J* = 8.9 Hz, 2H), 7.83 (d, *J* = 9.1 Hz, 1H), 7.72 (dd, *J* = 9.1, 2.2 Hz, 1H); ^{13}C NMR (101 MHz, $CDCl_3$) δ 148.7, 140.7, 139.4 (2 × C), 138.7, 133.0, 129.0, 128.7 (2 × CH), 124.0 (2 × CH), 118.7, 112.6; HRMS (ESI) m/z: $[M – H]^-$ Calcd for $C_{13}H_7Br_2N_4O_4$ 440.8853; Found 440.8847.

(*Z*)-*N*-(4-Bromo-2-nitrophenyl)-4-methoxybenzohydrazonoyl bromide (1d)

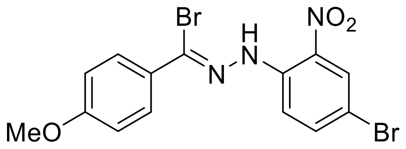

The reaction was performed as described in general procedure A using 2-nitrophenyl hydrazine (76.5 mg, 500 µmol), 4-methoxybenzaldehyde (66.9 µL, 550 µmol) and elemental bromine (51.2 µL, 1.00 mmol) to afford (*Z*)-*N*-(4-bromo-2-nitrophenyl)-4-methoxybenzohydrazonoyl bromide (**1d**) (159 mg, 74%) as a red solid. R*f* = 0.42 (pet. Ether/Et_2O 9:1); m.p. 169–171 °C; IR vmax (solid) 3275, 3107, 3012, 2960, 2927, 2910, 2854, 2832, 1610, 1560, 1485, 1398, 1301, 1255, 1217, 1176, 1145, 1113, 1070, 1039, 932, 882, 826, 730 cm^{-1}; ^{1}H NMR (500 MHz, $CDCl_3$) δ 11.30 (s, 1H), 8.37 (d, *J* = 2.3 Hz, 1H), 7.90 (d *J* = 9.0 Hz, 2H), 7.81 (d, *J* = 9.1 Hz, 1H), 7.64 (dd, *J* = 9.1, 2.3 Hz, 1H), 6.95 (d, *J* = 9.0 Hz, 2H), 3.88 (s, 3H); ^{13}C NMR (101 MHz, $CDCl_3$) δ 161.9, 139.7, 139.2, 132.3, 130.1 (2 × CH), 128.4, 127.8, 127.3, 118.7, 114.2 (2 × CH), 110.9, 55.7; HRMS (ESI) m/z: $[M - H]^-$ (Hydrazide–H) Calcd for $C_{14}H_{11}BrN_3O_4$ 363.9938; Found 363.9943.

(*Z*)-*N*-(4-Bromo-2-nitrophenyl)-4-(methylthio)benzohydrazonoyl bromide (1e)

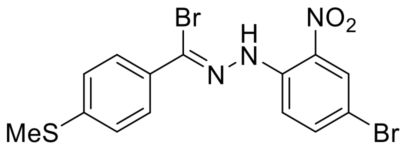

The reaction was performed as described in general procedure A using 2-nitrophenyl hydrazine (153 mg, 1.00 mmol), 4-(methylthio)benzaldehyde (150 µL, 1.10 mmol) and elemental bromine (102 µL, 2.00 mmol) to afford (*Z*)-*N*-(4-bromo-2-nitrophenyl)-4-(methylthio)benzohydrazonoyl bromide (**1e**) (304 mg, 69%) as an orange solid. R*f* = 0.78 (pet. Ether/EtOAc 4:1); m.p. 160–162 °C; IR vmax (solid) 3275, 3100, 2917, 1614, 1562, 1508, 1484, 1400, 1303, 1217, 1139, 1096, 1070, 931, 880, 813, 761 cm^{-1}; ^{1}H NMR (400 MHz, $CDCl_3$) δ 11.3 (s, 1H), 8.38 (d, *J* = 2.2 Hz, 1H), 7.88–7.85 (m, 2H), 7.81 (d, *J* = 9.1 Hz, 1H), 7.65 (dd, *J* = 9.1, 2.2 Hz, 1H), 7.29–7.26 (m, 2H), 2.54 (s, 3H); ^{13}C NMR (101 MHz, $CDCl_3$) δ 142.7, 139.5, 139.2, 132.4, 131.6, 128.7 (2 × CH), 128.5, 126.9, 125.8 (2 × CH), 118.7, 111.2, 15.4; HRMS (ESI) m/z: $[M - H]^-$ (Hydrazide–H) Calcd for $C_{14}H_{11}BrN_3O_3S$ 379.9710; Found 379.9713.

(*Z*)-*N*-(4-Bromo-2-nitrophenyl)-2-methylbenzohydrazonoyl bromide (1f)

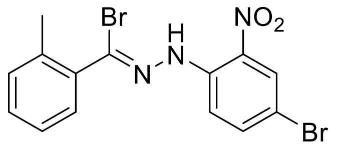

The reaction was performed as described in general procedure A using 2-nitrophenyl hydrazine (76.5 mg, 500 µmol), 2-methylbenzaldehyde (63.6 µL, 500 µmol) and elemental bromine (51.2 µL, 1.00 mmol) to afford (*Z*)-*N*-(4-bromo-2-nitrophenyl)-2-methylbenzohydrazonoyl bromide (**1f**) (106 mg, 52%) as a yellow solid. R*f* = 0.68 (pet. Ether/Et_2O 9:1); m.p. 120–122 °C; IR vmax (solid) 3275, 3100, 2917, 1614, 1562, 1508, 1484, 1400, 1303, 1217, 1139, 1096, 1070, 931, 880, 813, 761 cm^{-1}; ^1H NMR (500 MHz, $CDCl_3$) δ 11.29 (s, 1H), 8.38 (d, *J* = 2.2 Hz, 1H), 7.70 (d, *J* = 9.1 Hz, 1H), 7.63–7.57 (m, 2H), 7.37–7.27 (m, 3H), 2.52 (s, 3H); ^{13}C NMR (101 MHz, $CDCl_3$) δ 139.6, 139.3, 136.6, 136.0, 132.4, 131.2, 130.6, 130.2, 128.4, 126.2, 125.3, 118.6, 111.3, 21.3; HRMS (ESI) m/z: $[M – H]^–$ (Hydrazide–H) Calcd for $C_{14}H_{11}BrN_3O_3$ 347.9989; Found 347.9990.

(*Z*)-*N*-(4-Bromo-2-nitrophenyl)-2-chlorobenzohydrazonoyl bromide (1g)

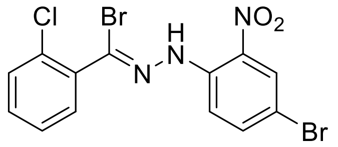

The reaction was performed as described in general procedure A using 2-nitrophenyl hydrazine (198 mg, 1.29 mmol), 2-chlorobenzaldehyde (160 µL, 1.42 mmol) and elemental bromine (133 µL, 2.60 mmol) to afford (*Z*)-*N*-(4-bromo-2-nitrophenyl)-2-chlorobenzohydrazonoyl bromide (**1g**) (245 g, 44%) as an orange solid. R*f* = 0.40 (pet. ether/EtOAc 9:1); m.p 110–112 °C; IR (solid) 3275, 3100, 1614, 1525, 1489, 1413, 1342, 1307, 1268, 1238, 1117, 1065, 932, 828, 754, 726 cm^{-1}; ^1H NMR (500 MHz, $CDCl_3$) *δ* 11.28 (s, 1H), 8.38 (d, *J* = 2.1 Hz, 1H), 7.77 (d, *J* = 9.2 Hz, 1H), 7.62 (dd, *J* = 9.2, 2.1 Hz, 1H), 7.60–7.56 (m, 1H), 7.51–7.46 (m, 1H), 7.41–7.35 (m, 2H); ^{13}C NMR (126 MHz, $CDCl_3$) *δ* 139.2, 139.1, 135.2, 132.8, 132.5, 131.6, 131.1, 130.6, 128.2, 126.9, 121.1, 118.8, 111.6; HRMS (ESI) m/z: $[M – H]^–$ (Hydrazide–H) Calcd for $C_{13}H_8BrClN_3O_3$ 367.9443; Found 367.9444.

(Z)-*N*-(4-Bromo-2-nitrophenyl)-2,6-dichlorobenzohydrazonoyl bromide(1h)

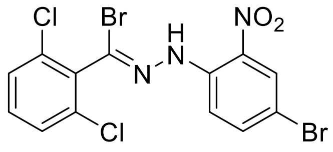

The reaction was performed as described in general procedure A using 2-nitrophenyl hydrazine (159 mg, 1.04 mmol), using 2,6-dichlorobenzaldehyde (200 mg, 1.14 mmol) and elemental bromine (107 µL, 2.09 mmol). Water (30 mL) was added and the mixture was extracted with CH_2Cl_2 (3 × 50 mL). The combined organic extracts were dried over $MgSO_4$, filtered and concentrated *in vacuo*. Purification by flash column chromatography (petroleum ether/diethyl ether, 19:1) followed by recrystallisation from hot EtOH afforded (*Z*)-*N*-(4-bromo-2-nitrophenyl)-2,6-dichlorobenzohydrazonoyl bromide (**1h**) (117 mg, 24%) as a yellow solid. R*f* = 0.40 (pet. ether/EtOAc 9:1); m.p. 138–140 °C (EtOH); IR vmax (solid) 3288, 3256, 1610, 1567, 1528, 1497, 1433, 1409, 1342, 1277, 1152, 1102, 932, 761 cm^{-1}; ^{1}H NMR (500 MHz, $CDCl_3$) δ 11.30 (s, 1H), 8.38 (d, *J* = 2.2 Hz, 1H), 7.69 (d, *J* = 9.1 Hz, 1H), 7.60 (dd, *J* = 9.1, 2.2 Hz, 1H), 7.44–7.39 (m, 2H), 7.37–7.32 (m, 1H); ^{13}C NMR (126 MHz, $CDCl_3$) δ 139.2, 135.2 (2 × C), 134.7, 132.6, 131.5, 128.3 (2 × CH), 128.2, 118.8, 118.1, 111.7 (2 × C); HRMS (ESI) m/z: $[M - H]^-$ (Hydrazide–H) Calcd for $C_{13}H_7BrCl_2N_3O_3$ 401.9053; Found 401.9056.

(Z)-*N*-(4-Bromo-2-nitrophenyl)-3-phenylpropanehydrazonoyl bromide (1i)

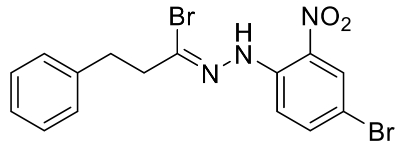

The reaction was performed as described in general procedure A using 2-nitrophenyl hydrazine (200 mg, 1.30 mmol), 3-phenylpropionaldehyde (190 µL, 1.43 mmol) and elemental bromine (102 µL, 2.60 mmol) to afford (*Z*)-*N*-(4-bromo-2-nitrophenyl)-3-phenylpropanehydrazonoyl bromide (**1i**) (107 mg, 19%) as a red amorphous solid. R*f* = 0.65 (pet. ether/Et_2O 9:1); IR vmax (solid) 3282, 3090, 3029, 2932, 2861, 1614, 1567, 1495, 1340, 1271, 1152, 1053, 702 cm^{-1}; ^{1}H NMR (400 MHz, D_6-DMSO) δ 10.71 (s, 1H), 8.25 (d, *J* = 2.3 Hz, 1H), 7.83 (dd, *J* = 9.1, 2.3 Hz, 1H), 7.58 (d, *J* = 9.1 Hz, 1H), 7.31–7.29 (m, 4H), 7.23–7.16 (m, 1H), 3.17 (t, *J* = 7.4 Hz, 2H), 3.03 (t, *J* = 7.4 Hz, 2H); ^{13}C NMR *δ* (101 MHz, D_6-DMSO) δ 139.7, 139.2, 139.0, 132.0, 128.9, 128.4 (2 × CH), 128.4 (2 × CH), 127.6, 126.3, 118.4, 110.0,

42.5, 32.5; HRMS (ESI) m/z: [M – H]⁻ (Hydrazide–H) Calcd for $C_{15}H_{13}BrN_3O_3$ 362.0146; Found 362.0152.

(*Z*)-*N*-(4-Bromo-2-nitrophenyl)butyrohydrazonoyl bromide (1j)

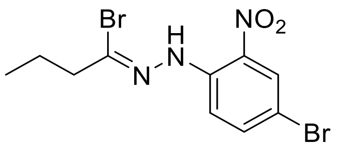

The reaction was performed as described in general procedure A using 2-nitrophenyl hydrazine (200 mg, 1.31 mmol), butanal (90.0 µL, 1.00 mmol) and elemental bromine (102 µL, 2.00 mmol) to afford (*Z*)-*N*-(4-bromo-2-nitrophenyl)butyrohydrazonoyl bromide (**1j**) (91 mg, 25%) as a red solid. R*f* = 0.88 (pet. ether/Et_2O 9:1); m.p. 58–60 °C; IR vmax (solid) 3280, 3098, 2966, 2934, 2876, 1614, 1495, 1342, 1271, 1152 cm^{-1}; ^1H NMR (500 MHz, $CDCl_3$) δ 10.95 (s, 1H), 8.32 (d, *J* = 2.1 Hz, 1H), 7.65 (d, *J* = 9.1 Hz, 1H), 7.58 (dd, *J* = 9.1, 2.1 Hz, 1H), 2.77 (t, *J* = 7.4 Hz, 2H), 1.78 (sext, *J* = 7.4 Hz, 2H), 1.01 (t, *J* = 7.4 Hz, 3H); ^{13}C NMR (101 MHz, $CDCl_3$) δ 139.8, 139.1, 132.0, 130.2, 128.8, 118.6, 110.6, 43.8, 20.8, 13.2; HRMS (ESI) m/z: [M – H]⁻ (Hydrazide–H) Calcd for $C_{10}H_{11}BrN_3O_3$ 299.9989; Found 299.9993.

(*Z*)-*N*-(4-Bromo-2-nitrophenyl)cyclohexanecarbohydrazonoyl bromide (1k)

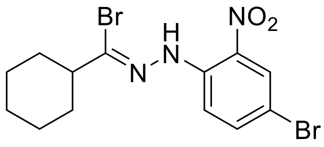

To a stirred solution 2-nitrophenylhydrazine (150 mg, 979 µmol), cyclohexanecarboxaldehyde (108 µL, 892 µmol) in EtOH (5 mL) was added a few drops of concentrated H_2SO_4 and stirred at reflux for 2 h. After cooling to room temperature, the reaction mixture was poured into Et_2O (20 mL) and washed with 1 M aq. HCl (20 mL), saturated aq. $NaHCO_3$ (20 mL) and brine (20 mL). The organic layer was dried over $MgSO_4$, filtered and concentrated *in vacuo* to afford the hydrazone intermediate which was used immediately without further purification. The residue was dissolved in glacial acetic acid (5 mL) and elemental bromine (50.0 µL, 976 µmol) was added dropwise. The resulting reaction mixture was stirred at room temperature for 2 h. Water (10 mL) was added, and the reaction mixture was extracted with diethyl ether (2 × 20 mL). The combined organic extracts were washed with saturated aq. $NaHCO_3$ (20 mL) and brine (20 mL), dried over $MgSO_4$, filtered and concentrated *in vacuo*. The crude residue was passed

through a silica plug (petroleum ether/ethyl acetate, 9:1) afforded (*Z*)-*N*-(4-bromo-2-nitrophenyl)cyclohexanecarbohydrazonoyl bromide (**1k**) (179 mg, 50% over two-steps) as an orange solid. R*f* = 0.60 (pet. ether/EtOAc 9:1); m.p. 78–80 °C; IR vmax (solid) 3271, 2932, 2858, 1641, 1567, 1525, 1495, 1405, 1346, 1271, 1156, 1145, 1104, 1061, 886, 823 cm^{-1}; ^{1}H NMR (500 MHz, $CDCl_3$) δ 10.95 (s, 1H), 8.30 (d, *J* = 2.2 Hz, 1H), 7.63 (d, *J* = 9.1 Hz, 1H), 7.57 (dd, *J* = 9.1, 2.2 Hz, 1H), 2.62 (tt, *J* = 11.3, 3.4 Hz, 1H), 2.11–2.04 (m, 2H), 1.84 (dt, *J* = 13.0, 3.3 Hz, 2H), 1.76–1.68 (m, 1H), 1.54 (qd, *J* = 12.6, 3.2 Hz, 2H), 1.41–1.19 (m, 3H); ^{13}C NMR (126 MHz, $CDCl_3$) δ 139.8, 138.9, 133.3, 131.8, 128.1, 118.5, 110.3, 49.9, 31.4 (2 × CH_2), 25.9, 25.6 (2 × CH_2); HRMS (ESI) m/z: $[M - H]^-$ (Hydrazide–H) Calcd for $C_{13}H_{15}BrN_3O_3$ 340.0302; Found 340.0304.

(*Z*)-*N*-(4-Bromo-2-nitrophenyl)pivalohydrazonoyl bromide (1l)

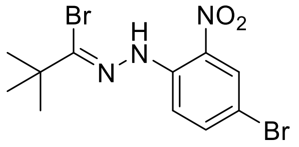

(*Z*)-*N*-(4-bromo-2-nitrophenyl)pivalohydrazonoyl bromide (**1l**) was synthesised as described for (*Z*)-*N*-(4-bromo-2-nitrophenyl)cyclohexanecarbohydazonyl bromide (**1k**) using 2-nitrophenylhydrazine (196 mg, 1.28 mmol), trimethylacetaldehyde (126 µL, 1.16 mmol) and elemental bromine (50.0 µL, 976 µmol) to afford (*Z*)-*N*-(4-bromo-2-nitrophenyl)pivalohydrazonoyl bromide (**1l**) (140 mg, 32% over two-steps) as a yellow solid. R*f* = 0.57 (pet. ether/EtOAc 9:1); m.p. 62–64 °C; IR vmax (solid) 3278, 2973, 1618, 1565, 1526, 1491, 1409, 1344, 1303, 1271, 1147, 1124, 981, 884, 836 cm^{-1}; ^{1}H NMR (500 MHz, $CDCl_3$) δ 11.0 (s, 1H), 8.33 (d, *J* = 1.9 Hz, 1H), 7.64 (d, *J* = 9.1 Hz, 1H), 7.59 (dd, *J* = 9.1, 1.9 Hz, 1H), 1.35 (s, 9H); ^{13}C NMR (126 MHz, $CDCl_3$) δ 140.2, 140.0, 139.0, 132.0, 128.2, 118.5, 110.4, 43.1, 28.9 (3 × CH_3); HRMS (ESI) m/z: $[M – H]^-$ (Hydrazide–H) Calcd for $C_{11}H_{13}BrN_3O_3$ 314.0146; Found 314.0148.

(*Z*)-*N*-(4-Bromo-2-nitrophenyl)tetrahydrofuran-3-carbohydrazonyl bromide (1m)

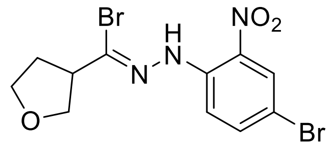

(*Z*)-*N*-(4-Bromo-2-nitrophenyl)tetrahydrofuran-3-carbohydrazonyl bromide (**1m**) was synthesized as described for (*Z*)-*N*-(4-bromo-2-nitrophenyl)cyclohexanecarbohydazonyl bromide (**1k**) using 2-nitrophenylhydrazine hydrochloride (200 mg, 1.05 mmol), tetrahydrofuran-3-carboxaldehyde (192 µL, 959 µmol, 50 wt% in H_2O) and elemental bromine (61.5 µL, 1.20 mmol). The first step was carried out at 80 °C for 3 h and the second step at room temperature for 16 h. Water (20 mL) was added to the reaction mixture and the resulting precipitate was collected. Recrystallisation from hot EtOH afforded (*Z*)-*N*-(4-bromo-2-nitrophenyl)tetrahydrofuran-3-carbohydrazonyl bromide (**1m**) (58.1 mg, 15% over two steps) as a yellow solid. R*f* = 0.48 (pet. ether/EtOAc 7:3); m.p. 68–70 °C (EtOH); IR (solid) 3260, 2960, 1609, 1485, 1416, 1339, 1300, 1267, 1144, 1011, 905, 874, 829 cm^{-1}; ^{1}H NMR (500 MHz, $CDCl_3$) δ 11.02 (s, 1H), 8.34 (d, *J* = 2.1 Hz, 1H), 7.64 (d, *J* = 9.2 Hz, 1H), 7.61 (dd, *J* = 9.2, 2.1 Hz, 1H), 4.08–3.99 (m, 3H), 3.94–3.88 (m, 1H), 3.60–3.52 (m, 1H), 2.37–2.23 (m, 2H); ^{13}C NMR (126 MHz, $CDCl_3$) δ 139.6, 139.2 (2 × C), 130.3, 128.4, 118.5, 111.0, 71.3, 68.3, 50.4, 31.4; HRMS (ESI) m/z: $[M + Na]^+$ Calcd for $C_{11}H_{11}Br_2N_3O_3Na$ 413.9059; Found 413.9059.

5-(4-Bromophenyl)-3-methylisoxazole-4-carbaldehyde (S1)

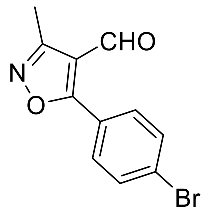

Borane Reduction: To a stirred solution of 5-(4-bromophenyl)-3-methylisoxazole-4-carboxylic acid (1.00 g, 3.54 mmol) in anhydrous THF (20 mL) was added borane tetrahydrofuran complex (8.90 mL, 8.90 mmol; 1 M in THF) dropwise at 0 °C. The reaction mixture was warmed to 60 °C and stirred for 2 h. After cooling to room temperature, the reaction mixture was quenched by the addition of 1 M aq. HCl and stirred for 20 minutes. The phases were separated and the aqueous phase was extracted with EtOAc (3 × 50 mL) and the combined organic extracts were washed with brine (100 mL), dried over $MgSO_4$, filtered and

concentrated *in vacuo*. Purification by flash column chromatography using a gradient system (pet. ether/ EtOAc 7:3 to 3:7) afforded (5-(4-bromophenyl)-3-methylisoxazol-4-yl)methanol (825 mg, 87%) as a white solid which was used immediately in the next step.

Swern Oxidation: To a stirred solution of dimethyl sulfoxide (1.02 mL, 14.4 mmol) in anhydrous CH_2Cl_2 (15 mL) was added oxalyl chloride (741 µL, 8.64 mmol) dropwise and the resulting mixture was stirred at −78 °C for 20 minutes. (5-(4-Bromophenyl)-3-methylisoxazol-4-yl)methanol (773 mg, 2.90 mmol) in anhydrous CH_2Cl_2 (15 mL) and added dropwise over 20 minutes and stirred at −78 °C for 1 h. Triethylamine (2.81 mL, 20.2 mmol) was added dropwise, stirred at −78 °C for 0.5 h and then allowed to warm to room temperature and stirred for 3 h. The reaction was diluted with water (20 mL) and extracted with CH_2Cl_2 (3 × 50 mL). The combined organic extracts were washed with water (100 mL), brine (100 mL), dried over $MgSO_4$, filtered and concentrated *in vacuo*. Purification by flash column chromatography (pet. ether/EtOAc, 9:1) afforded 5-(4-bromophenyl)-3-methylisoxazole-4-carbaldehyde (**S1**) (652 mg, 85%) as a white solid. R*f* = 0.26 (pet. ether/EtOAc 9:1), m.p. 98–100 °C; IR v_{max} (solid) 3086, 2864, 1684, 1595, 1585, 1476, 1435, 1410, 1396, 1383, 1072, 1007, 826, 773, 729, 718 cm^{-1}; ^1H NMR (500 MHz, $CDCl_3$) δ 10.07 (s, 1H), 7.74 (d, *J* = 8.6 Hz, 2H), 7.70 (d, *J* = 8.6 Hz, 2H), 2.55 (s, 3H); ^{13}C NMR (126 MHz, $CDCl_3$) δ 184.1, 174.0, 160.3, 132.3 (2 × CH), 130.1 (2 × CH), 127.3, 124.9, 115.7, 11.4; HRMS (ESI) m/z: $[M + H]^+$ Calcd for $C_{11}H_9BrNO_2$ 265.9811; Found 265.9814.

(*Z*)-*N*-(4-Bromo-2-nitrophenyl)-5-(4-bromophenyl)-3-methylisoxazole-4-carbohydrazonoyl bromide (1n)

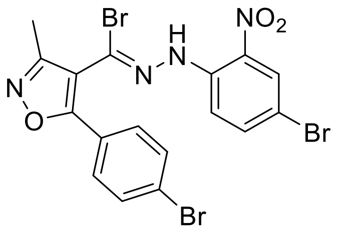

The reaction was performed as described in general procedure A using 2-nitrophenylhydrazine hydrochloride (81.0 mg, 427 µmol), 5-(4-bromophenyl)-3-methylisoxazole-4-carbaldehyde (**S1**) (124 mg, 468 µmol) and elemental bromine (44.0 µL, 859 µmol) to afford (*Z*)-*N*-(4-bromo-2-nitrophenyl)-5-(4-bromophenyl)-3-methylisoxazole-4-carbohydrazonoyl bromide (**1n**) (143 mg, 55%) as a yellow solid. R*f* = 0.68 (pet. ether/EtOAc 7:3); m.p. 73–75 °C; IR (solid) 3260, 3088, 1607, 1650, 1522, 1485, 1416, 1339, 1300, 1275, 1144, 1134, 1063, 1011, 908, 874, 829, 814, 719 cm^{-1}; ^1H NMR (500 MHz, $CDCl_3$) δ 11.37 (s,

1H), 8.39 (s, 1H), 7.67–7.53 (m, 6H), 2.45 (s, 3H); ^{13}C NMR (126 MHz, $CDCl_3$) δ 167.2, 159.4, 139.5, 138.8 (2 × C), 132.8, 132.4 (2 × CH), 129.5 (2 × CH), 128.6, 126.0, 125.6, 118.4, 113.9, 112.2, 11.2; HRMS (ESI) m/z: $[M - H]^-$ Calcd for $C_{17}H_{10}Br_3N_4O_3$ 554.8314; Found 554.8318.

(*Z*)-*N*-(4-Bromo-2-nitrophenyl)-4-chlorobenzohydrazonoyl bromide (1o)

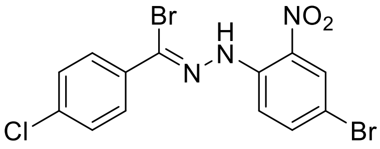

The reaction was performed as described in general procedure A using 2-nitrophenyl hydrazine hydrochloride (1.23 g, 6.48 mmol), 4-chlorobenzaldehyde (1.00 g, 7.11 mmol) and elemental bromine (730 µL, 14.2 mmol) to afford (*Z*)-*N*-(4-bromo-2-nitrophenyl)-4-chlorobenzohydrazonoyl bromide (**1o**) (1.49 g, 53%) as a red solid. R*f* = 0.48 (pet. ether/EtOAc 9:1); m.p. 196–198 °C; IR (solid) 3263, 3100, 1618, 1562, 1485, 1402, 1305, 1264, 1141, 1096, 934, 884, 828 cm^{-1}; ^{1}H NMR (500 MHz, $CDCl_3$) δ 11.34 (s, 1H), 8.38 (d, *J* = 2.3 Hz, 1H), 7.88 (d, *J* = 8.7 Hz, 2H), 7.80 (d, *J* = 9.1 Hz, 1H), 7.66 (dd, *J* = 9.1, 2.3 Hz, 1H), 7.41 (d, *J* = 8.7 Hz, 2H); ^{13}C NMR (101 MHz, $CDCl_3$) δ 139.3 (2 × C), 137.0, 133.7, 132.6, 129.6 (2 × CH), 129.0 (2 × CH), 128.5, 125.8, 118.7, 111.6 ; HRMS (ESI) m/z: $[M–H]^-$ (Hydrazide–H) Calcd for $C_{13}H_8BrClN_3O_3$ 367.9443; Found 367.9446.

***N*-(4-Bromo-2-nitrophenyl)pentanehydazonyl bromide (1p)**

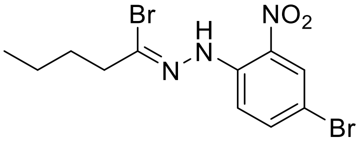

The reaction was performed as described in general procedure A except both steps were carried out at room temperature using 2-nitrophenylhydrazine hydrochloride (500 mg, 2.64 mmol), pentanal (309 µL, 2.91 mmol) and elemental bromine (270 µL, 5.27 mmol). After completion of the bromination step, the reaction mixture was diluted with water and extracted with CH_2Cl_2 (3 × 50 mL). The combined organic extracts were washed with water (80 mL), saturated aqueous $NaHCO_3$ (80 mL), 1 M aq. $Na_2S_2O_3$ (80 mL) and brine (80 mL), dried over $MgSO_4$, filtered and concentrated *in vacuo*. Recrystallisation from hot EtOH afforded *N*-(4-bromo-2-nitrophenyl)pentanehydazonyl bromide (**1p**) (5:1 ratio of *Z* to *E* isomers) (239 mg,

24% over two-steps) as a yellow solid. Spectroscopic data for the major *Z* isomer is reported; R*f* = 0.57 (pet. ether/EtOAc 7:3); m.p. 50–52 °C (EtOH); IR (solid) 3278, 2966, 2938, 1616, 1567, 1409, 1355, 1305, 1147, 1044, 886, 832 cm^{-1}; ^{1}H NMR (500 MHz, $CDCl_3$) δ 10.95 (s, 1H), 8.33 (d, *J* = 2.2 Hz, 1H), 7.66 (d, *J* = 9.1 Hz, 1H), 7.59 (dd, *J* = 9.1, 2.2 Hz, 1H), 2.80 (t, *J* = 7.4 Hz, 2H), 1.74 (pent., *J* = 7.4 Hz, 2H), 1.42 (sext., *J* = 7.4 Hz, 2H), 0.97 (t, *J* = 7.4 Hz, 3H); ^{13}C NMR (101 MHz, $CDCl_3$) δ 139.9, 139.1, 139.0, 130.4, 128.3, 118.6, 110.6, 41.6, 29.4, 21.8, 13.9; HRMS (ESI) m/z: $[M – H]^{–}$ (Hydrazide–H) Calcd for $C_{11}H_{13}BrN_3O_3$ 314.0146; Found 314.0144.

5-Bromo-1H-1,2,3-benzotriazol-1-yl 4-methylbenzoate (2)

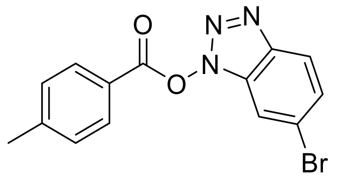

To a stirred solution of *N*-(4-bromo-2-nitrophenyl)-4-methylbenzohydrazonyl bromide (**1a**) (207 mg, 504 µmol) in MeCN (50 mL) was added triethylamine (351 µl, 2.52 mmol). The resulting mixture was heated to 50 °C for 0.25 h. The reaction mixture was cooled to rt and concentrated *in vacuo*. The crude residue was suspended in EtOAc (20 mL) and the resulting solid was filtered. The filtrate was washed with 1 M aqueous HCl (2 × 20 mL), saturated aqueous $NaHCO_3$ (20 mL) and brine (20 mL). The organic layer was then dried over $MgSO_4$, filtered and concentrated *in vacuo*. Purification by flash column chromatography (pet. ether/EtOAc 9:1) afforded 5-bromo-1H-1,2,3-benzotriazol-1-yl 4-methylbenzoate (**2**) (86.0 mg, 52%) as an off-white solid. R*f* = 0.83 (pet. ether/EtOAc 7:3); m.p. 126–128 °C; IR vmax (solid) 3098, 3075, 2925, 1780, 1612, 1232, 1184, 1098, 981, 927, 811, 795, 739 cm^{-1}; ^{1}H NMR (500 MHz, $CDCl_3$) δ (500 MHz, CDCl3) δ 8.16 (d, *J* = 8.3 Hz, 2H), 7.97 (d, *J* = 8.9 Hz, 1H), 7.67 (d, *J* = 1.6 Hz, 1H), 7.54 (dd, *J* = 8.9, 1.6 Hz, 1H), 7.42 (d, *J* = 8.3 Hz, 2H), 2.52 (s, 3H); ^{13}C NMR (101 MHz, $CDCl_3$) δ 162.7, 147.3, 142.6, 131.0 (2 × CH), 130.2, 130.2 (2 × CH), 129.9, 123.4, 121.9, 121.8, 111.6, 22.2; HRMS (ESI) m/z: $[M + H]^{+}$ Calcd for $C_{14}H_{11}BrN_3O_2$ 332.0016; Found 332.0015.

General Procedure B: Preparation of Amide Products

Hydrazonyl bromide (1 equiv.) was dissolved in acetonitrile (50 mL) and triethylamine (5 equiv.) was added. The resulting mixture was heated to 50 °C and stirred for 0.25 h before the appropriate amine (1.1 equiv.) was added. The reaction mixture was stirred for 0.1–1 h and then cooled to room temperature and concentrated *in vacuo*. The crude residue was suspended in EtOAc (20 mL) and the resulting solid was filtered. The filtrate was washed with 1 M aq. HCl (2 × 20 mL), saturated aq. $NaHCO_3$ (20 mL) and brine (20 mL). The organic layer was dried over $MgSO_4$, filtered and concentrated *in vacuo*. The resulting residue was purified by flash column chromatography to afford the desired amide.

***N*-Benzyl-4-methylbenzamide (3a)[1]**

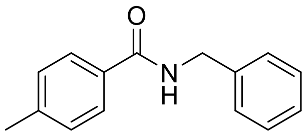

The reaction was performed as described in general procedure B using *N*-(4-Bromo-2-nitrophenyl)-4-methylbenzohydrazonyl bromide (**1a**) (207 mg, 504 µmol), triethylamine (348 µl, 2.50 mmol) and benzylamine (60.0 µl, 550 µmol). Purification by flash column chromatography (pet. ether/EtOAc 17:3) afforded *N*-benzyl-4-methylbenzamide (**3a**) (90.0 mg, 79%) as an off-white solid. R*f* = 0.30 (pet. ether/EtOAc 4:1); ^{1}H NMR (500 MHz, $CDCl_3$) δ 7.69 (d, *J* = 8.2 Hz, 2H), 7.37–7.34 (m, 4H), 7.32–7.28 (m, 1H), 7.23 (d, *J* = 8.2 Hz, 2H), 6.35 (br s, 1H), 4.65 (d, *J* = 5.7 Hz, 2H), 2.39 (s, 3H); ^{13}C NMR (126 MHz, $CDCl_3$) δ 167.4, 142.1, 138.4, 131.7, 129.4, 128.9, 128.1, 127.8, 127.1, 44.3, 21.6; LCMS (EI) m/z: $[M + H]^+$ Calcd for $C_{15}H_{16}NO$ 226.1; Found 226.1 at 7.21 mins. This compound was known in the literature and spectral data are agreed with that of reported values.[1]

Large scale synthesis of *N*-benzyl-4-methylbenzamide (3a)

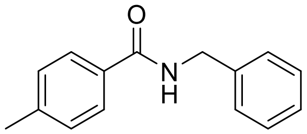

To a stirred suspension of (*Z*)-*N*-(4-bromo-2-nitrophenyl)-4-methylbenzohydazonyl bromide (**1a**) (750 mg, 1.83 mmol) in acetonitrile (150 mL) was added triethylamine (1.27 mL, 9.08 mmol). The mixture was heated to 50 °C and stirred for 0.25 h before benzylamine (218 µL,

2.00 mmol) was added. The reaction mixture was stirred at 50 °C for 1.5 h, cooled to room temperature and concentrated *in vacuo*. Purification by flash column chromatography using a gradient system (pet. ether/EtOAc 9:1 to 4:1) afforded *N*-benzyl-4-methylbenzamide (**3a**) (382 mg, 93%) as a brown solid. Characterization data as reported above for *N*-benzyl-4-methylbenzamide (**3a**).

N-(2-Methoxybenzyl)-4-methylbenzamide (3b)

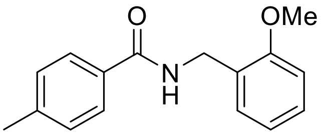

The reaction was performed as described in general procedure B using *N*-(4-bromo-2-nitrophenyl)-4-methylbenzohydrazonyl bromide (**1a**) (207 mg, 504 µmol), triethylamine (348 µl, 2.50 mmol) and 2-methoxybenzylamine (196 µL, 1.50 mmol). Purification by flash column chromatography (pet. ether/EtOAc 17:3) afforded *N*-(2-methoxybenzyl)-4-methylbenzamide (**3b**) (115 mg, 90%) as an off-white solid. R*f* = 0.45 (pet. ether/EtOAc 7:3); m.p. 96–98 °C; IR vmax (solid) 3314, 3000, 3034, 3005, 2925, 2839, 1636, 1539, 1495, 1465, 1292, 1243, 111, 1031, 752 cm^{-1}; ^{1}H NMR (500 MHz, $CDCl_3$) δ 7.66 (d, *J* = 8.0 Hz, 2H), 7.35 (dd, *J* = 7.4, 1.5 Hz, 1H), 7.27 (td, *J* = 8.2, 1.5 Hz, 1H), 7.20 (d, *J* = 8.0 Hz, 2H), 6.93 (t, *J* = 7.4 Hz, 1H), 6.89 (d, *J* = 8.2 Hz, 1H), 6.67 (br s, 1H), 4.64 (d, *J* = 5.8 Hz, 2H), 3.88 (s, 3H), 2.38 (s, 3H); ^{13}C NMR (101 MHz, $CDCl_3$) δ 167.3, 157.8, 141.8, 132.1, 130.1, 129.3 (2 × CH), 129.1, 127.1 (2 × CH), 126.4, 120.9, 110.5, 55.5, 40.1, 21.5; HRMS (ESI) m/z: $[M + H]^+$ Calcd for $C_{16}H_{18}NO_2$ 256.1323; Found 256.1323

4-Methyl-*N*-(1-phenylethyl)benzamide (3c)[2]

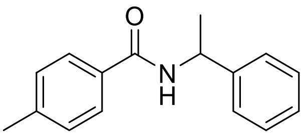

The reaction was performed as described in general procedure B using *N*-(4-bromo-2-nitrophenyl)-4-methylbenzohydrazonyl bromide (**1a**) (207 mg, 504 µmol), triethylamine (348 µl, 2.50 mmol) and 1-phenylethylamine (70.2 µL, 550 µmol). Purification by flash column chromatography (pet. ether/EtOAc 17:3) afforded 4-methyl-*N*-(1-phenylethyl)benzamide (**3c**) (90.0 mg, 75%) as an off-white solid. R*f* = 0.33 (pet. ether/EtOAc 4:1); ^{1}H NMR (500 MHz, $CDCl_3$) δ 7.67 (d, *J* = 8.2 Hz, 2H), 7.42–7.33 (m, 4H), 7.29 (dt, *J* = 7.9, 2.3 Hz, 1H), 7.22 (d, *J*

= 7.9 Hz, 2H), 6.26 (br d, J = 6.6 Hz, 1H), 5.34 (p, J = 7.3 Hz, 1H), 2.39 (s, 3H), 1.61 (d, J = 6.9 Hz, 3H); ^{13}C NMR (101 MHz, $CDCl_3$) 166.6, 143.4, 142.0, 131.9, 129.4 (2 × CH), 128.9 (2 × CH), 127.6, 127.1 (2 × CH), 126.4 (2 × CH), 49.3, 21.9, 21.6; LCMS (EI) m/z: $[M + H]^+$ Calcd for $C_{16}H_{18}NO$ 240.1; Found 240.1 at 7.08 mins. This compound was known in the literature and spectral data are agreed with that of reported values.[2]

4-Methyl-*N*-(2-phenylpropan-2-yl)benzamide (3d)

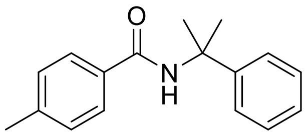

The reaction was performed as described in general procedure B using *N*-(4-bromo-2-nitrophenyl)-4-methylbenzohydrazonyl bromide (**1a**) (207 mg, 504 µmol), triethylamine (348 µl, 2.50 mmol) and phenylisopropylamine (79.1 µL, 0.55 mmol). Purification by flash column chromatography (pet. ether/EtOAc 17:3) afforded 4-methyl-*N*-(2-phenylpropan-2-yl)benzamide (**3d**) (40.1 mg, 31%) as an off-white solid. R*f* = 0.65 (pet. ether/EtOAc 7:3); m.p. 169–171 °C; IR vmax (solid) 3291, 3064, 3027, 2999, 2979, 2930, 1636, 1539, 1508, 1312, 765, 700 cm^{-1}; ^{1}H NMR (500 MHz, $CDCl_3$) δ 7.66 (d, J = 8.2 Hz, 2H), 7.48–7.43 (m, 2H), 7.38–7.31 (m, 2H), 7.26–7.21 (m, 3H), 6.35 (br s, 1H), 2.39 (s, 3H), 1.83 (s, 6H); ^{13}C NMR (101 MHz, $CDCl_3$) δ 166.5, 147.1, 141.8, 132.7, 129.3 (2 × CH), 128.6 (2 × CH), 127.0 (2 × CH), 126.8, 124.9 (2 × CH), 56.3, 29.3 (2 × CH_3), 21.5; HRMS (ESI) m/z: $[M + H]^+$ Calcd for $C_{17}H_{20}NO$ 254.1539; Found 254.1533.

***N*-(Cyclohexylmethyl)-4-methylbenzamide (3e)[3]**

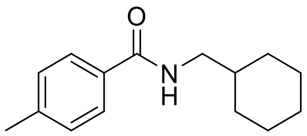

The reaction was performed as described in general procedure B using *N*-(4-bromo-2-nitrophenyl)-4-methylbenzohydrazonyl bromide (**1a**) (207 mg, 504 µmol), triethylamine (348 µl, 2.50 mmol) and cyclohexanemethylamine (71.6 µL, 550 µmol). Purification by flash column chromatography (pet. ether/EtOAc 17:3) afforded *N*-(cyclohexylmethyl)-4-methylbenzamide (**3e**) (81 mg, 70%) as an off-white solid. R*f* = 0.58 (pet. ether/EtOAc 7:3); ^{1}H NMR (500 MHz, $CDCl_3$) δ 7.65 (d, J = 8.1 Hz, 2H), 7.22 (d, J = 8.1 Hz, 2H), 6.07 (br s, 1H), 3.31 (t, J = 6.4 Hz, 2H), 2.39 (s, 3H), 1.83–1.72 (m, 4H), 1.72–1.65 (m, 1H), 1.63–1.55 (m, 1H), 1.32–1.14 (m,

3H), 1.07–0.97 (m, 2H); ^{13}C NMR (101 MHz, $CDCl_3$) δ 167.2, 141.8, 132.2, 129.3 (2 × CH), 127.0 (2 × CH), 46.3, 38.2, 31.1 (2 × CH_2), 26.6, 26.0 (2 × CH_2), 21.6; LCMS (EI) m/z: $[M + H]^+$ Calcd for $C_{15}H_{22}NO$ 232.2; Found 232.1 at 8.51 mins. This compound was known in the literature and spectral data are agreed with that of reported values.[3]

4-Methyl-*N*-(2-(thiophen-2-yl)ethyl)benzamide (3f)[4]

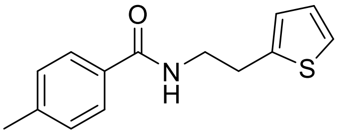

The reaction was performed as described in general procedure B using *N*-(4-bromo-2-nitrophenyl)-4-methylbenzohydrazonyl bromide (**1a**) (207 mg, 504 µmol), triethylamine (350 µl, 2.50 mmol) and 2-thiopheneethylamine (64.4 µL, 550 µmol). Purification by flash column chromatography (pet. ether/EtOAc 17:3) afforded 4-methyl-*N*-(2-(thiophen-2-yl)ethyl)benzamide (**3f**) (87.0 mg, 70%) as a brown solid. R*f* = 0.21 (pet. ether/EtOAc 4:1); ^1H NMR (500 MHz, $CDCl_3$) δ 7.62 (d, *J* = 8.2 Hz, 2H), 7.22 (d, *J* = 8.2 Hz, 2H), 7.18 (dd, *J* = 5.1, 1.2 Hz, 1H), 6.96 (dd, *J* = 5.1, 3.4 Hz, 1H), 6.87 (dd, *J* = 3.4, 1.2 Hz, 1H), 6.22 (br s, 1H), 3.73 (q, *J* = 6.5 Hz, 2H), 3.15 (t, *J* = 6.5 Hz, 2H), 2.39 (s, 3H); ^{13}C NMR (101 MHz, $CDCl_3$) δ 167.7, 142.1, 141.5, 131.8, 129.4 (2 × CH), 127.3, 127.0 (2 × CH), 125.6, 124.2, 41.4, 30.1, 21.6; LCMS (EI) m/z: $[M + H]^+$ Calcd for $C_{15}H_{16}NOS$ 246.1; Found 246.1 at 7.94 mins. This compound was known in the literature and spectral data are agreed with that of reported values.[4]

***N*-Butyl-4-methylbenzamide (3g)[5]**

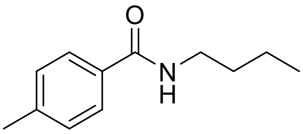

The reaction was performed as described in general procedure B using *N*-(4-Bromo-2-nitrophenyl)-4-methylbenzohydrazonyl bromide (**1a**) (207 mg, 504 µmol), triethylamine (348 µl, 2.50 mmol) and butylamine (54.4 µL, 550 µmol). Purification by flash column chromatography (pet. ether/EtOAc 17:3) afforded *N*-butyl-4-methylbenzamide (**3g**) as an orange solid (62 mg, 64%). R*f* = 0.24 (pet. ether/EtOAc 4:1); ^1H NMR (500 MHz, $CDCl_3$) δ 7.65 (d, *J* = 8.1 Hz, 2H), 7.22 (d, *J* = 8.1 Hz, 2H), 6.09 (br s, 1H), 3.43 (q, *J* = 6.4, 2H), 2.38 (s, 3H), 1.59 (p, *J* = 7.3 Hz, 2H), 1.40 (sext, *J* = 7.3 Hz, 2H), 0.95 (t, *J* = 7.3 Hz, 3H); ^{13}C NMR

(101 MHz, $CDCl_3$) δ 167.6, 141.8, 132.1, 129.3, (2 × CH), 126.9 (2 × CH), 39.9, 31.9, 21.5, 20.3, 13.9; LCMS (EI) m/z: $[M + H]^+$ Calcd for $C_{12}H_{18}NO$ 192.1; Found 192.2 at 7.59 mins. This compound was known in the literature and spectral data are agreed with that of reported values.[5]

_N_-Butyl-_N_,4-dimethylbenzamide (3h)

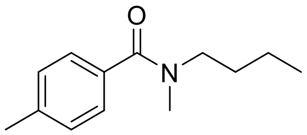

The reaction was performed as described in general procedure B using *N*-(4-bromo-2-nitrophenyl)-4-methylbenzohydrazonyl bromide (**1a**) (207 mg, 504 µmol), triethylamine (350 µl, 2.50 mmol) and *N*-methylbutylamine (65.1 µL, 550 µmol). Purification by flash column chromatography (pet. ether/EtOAc 17:3) afforded *N*-butyl-*N*,4-dimethylbenzamide (**3h**) (85 mg, 82%) as a yellow oil. R*f* = 0.39 (pet. ether/EtOAc 7:3); IR vmax (film) 2962, 2932, 2876, 1636, 1459, 1404, 1074, 894, 757 cm^{-1}; ^1H NMR (500 MHz, D_6-DMSO, 100 °C) δ 7.24 (t, *J* = 9.1 Hz, 4H), 3.33 (d, *J* = 7.1 Hz, 2H), 2.91 (s, 3H), 2.34 (s, 3H), 1.59–1.50 (m, 2H), 1.31–1.20 (m, 2H), 0.87 (t, *J* = 7.1 Hz, 3H); ^{13}C NMR (126 MHz, D_6-DMSO, 100 °C) δ 169.9, 138.0, 133.9, 128.1 (2 × CH), 126.1 (2 × CH), 47.7, 34.7 28.8, 20.2, 18.8, 12.8; HRMS (ESI) m/z: $[M + H]^+$ Calcd for $C_{13}H_{20}NO$ 206.1539; Found 206.1534.

_N_-(2-Methoxybenzyl)-_N_,4-dimethylbenzamide (3i)

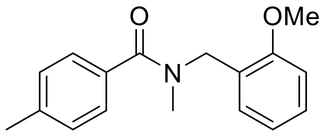

The reaction was performed as described in general procedure B using *N*-(4-bromo-2-nitrophenyl)-4-methylbenzohydrazonyl bromide (**1a**) (207 mg, 504 µmol), triethylamine (348 µl, 2.50 mmol) and 2-methoxy-*N*-benzylmethylamine (81.9 µL, 550 µmol). Purification by flash column chromatography (pet. ether/EtOAc 17:3) afforded *N*-(2-methoxybenzyl)-*N*,4-dimethylbenzamide (**3i**) (121 mg, 89%) as an orange solid. R*f* = 0.39 (pet. ether/EtOAc 7:3); m.p. 40–42 °C; IR vmax (solid) 3007, 2949, 2923, 2841, 1634, 1398. 1243, 1070, 1026, 832, 754 cm^{-1}; ^1H NMR (500 MHz, D_6-DMSO, 100 °C) δ 7.35–7.14 (m, 6H), 7.03–6.94 (m, 2H), 4.56 (s, 2H), 3.78 (s, 3H), 2.86 (s, 3H), 2.34 (s, 3H); ^{13}C NMR (126 MHz, D_6-DMSO, 100 °C) δ 170.4, 156.9, 138.3, 133.5, 128.1 (2 × CH), 127.9, 127.6, 126.2 (2 × CH), 124.6, 120.0,

110.8, 55.0, 46.94, 34.40, 20.18; HRMS (ESI) m/z: $[M + H]^+$ Calcd for $C_{17}H_{20}NO_2$ 270.1475; Found 270.1479.

_N_-Benzyl-_N_-ethyl-4-methylbenzamide (3j)

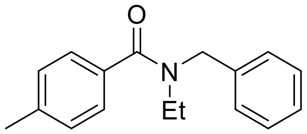

The reaction was performed as described in general procedure B using *N*-(4-bromo-2-nitrophenyl)-4-methylbenzohydrazonyl bromide (**1a**) (207 mg, 504 µmol), triethylamine (348 µl, 2.50 mmol) and *N*-ethylbenzylamine (71.0 µL, 550 µmol). Purification by flash column chromatography (pet. ether/EtOAc 17:3) afforded *N*-benzyl-*N*-ethyl-4-methylbenzamide (**3j**) (107 mg, 84%) as a brown oil. R*f* = 0.55 (pet. ether/EtOAc 7:3); IR vmax (film) 3064, 3033, 2977, 2938, 2878, 1633, 1456, 1426, 1290, 1080, 832, 756, 705 cm^{-1} ^1H NMR (500 MHz, D_6-DMSO, 100 °C) δ 7.38–7.32 (m, 2H), 7.32–7.21 (m, 7H), 4.60 (s, 2H), 3.29 (q, *J* = 7.2 Hz, 2H), 2.34 (s, 3H), 1.05 (t, *J* = 7.2 Hz, 3H); ^{13}C NMR (126 MHz, D_6-DMSO, 100 °C) δ 170.3, 138.2, 137.4, 133.7, 128.3 (2 × CH), 127.9 (2 × CH), 126.7 (2 × CH), 126.5, 125.8 (2 × CH), 48.4, 41.0, 20.2, 12.4; HRMS (ESI) m/z: $[M + H]^+$ Calcd for $C_{17}H_{20}NO$ 254.1526; Found 254.1529.

_N_,_N_,4-Trimethylbenzamide (3k)[6]

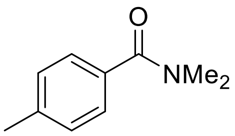

The reaction was performed as described in general procedure B using using *N*-(4-bromo-2-nitrophenyl)-4-methylbenzohydrazonyl bromide (**1a**) (100 mg, 243 µmol), triethylamine (170 µl, 1.22 mmol) and dimethylamine hydrochloride (22.0 mg, 270 µmol). Purification by flash column chromatography (pet. Ether/EtOAc, 4:1 to 1:1) gave *N*,*N*-dimethyl-4-methylbenzamide (**3k**) (20.2 mg, 51%) as a yellow oil. R*f* = 0.22 (pet. Ether/EtOAc 1:1); ^1H NMR (500 MHz, $CDCl_3$) δ 7.24 (d, *J* = 8.1 Hz, 2H), 7.12 (d, *J* = 8.1 Hz, 2H), 3.02 (br s, 3H), 2.91 (br s, 3H), 2.30 (s, 3H); ^{13}C NMR (126 MHz, $CDCl_3$) δ 171.9, 139.7, 135.5, 129.0 (2 × CH), 127.3 (2 × CH), 39.8, 35.5, 21.5 (2 × CH_3); LCMS (EI) m/z: $[M + H]^+$ Calcd for $C_{10}H_{14}NO$ 164.1; Found 164.2 at 6.67 mins. This compound was known in the literature and spectral data are agreed with that of reported values.[6]

N,N-Diethyl-4-methylbenzamide (3l)[7]

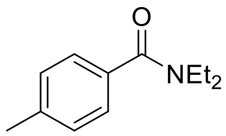

The reaction was performed as described in general procedure B using *N*-(4-bromo-2-nitrophenyl)-4-methylbenzohydrazonyl bromide (**1a**) (207 mg, 504 µmol), triethylamine (348 µl, 2.50 mmol) and diethylamine (56.9 µL, 550 µmol). Purification by flash column chromatography (pet. ether/EtOAc 17:3) afforded *N,N*-diethyl-4-methylbenzamide (**3l**) (55.0 mg, 57%) as a yellow oil. R*f* = 0.31 pet. ether/EtOAc 7:3); ^{1}H NMR (500 MHz, $CDCl_3$, 50 °C) δ 7.26 (d, *J* = 8.0 Hz, 2H), 7.18 (d, *J* = 7.9 Hz, 2H), 3.41 (br s, 4H), 2.36 (s, 3H), 1.17 (br s, 6H); ^{13}C NMR (126 MHz, $CDCl_3$, 50 °C) δ 171.7, 139.2, 134.7, 129.1 (2 × CH), 126.6 (2 × CH), 43.4, 29.4, 13.7; LCMS (EI) m/z: $[M + H]^+$ Calcd for $C_{12}H_{18}NO$ 192.1; Found 192.2 at 7.79 mins. This compound was known in the literature and spectral data are agreed with that of reported values.[7]

***N*-Methoxy-*N*-4-dimethylbenzamide (3m)**[8]

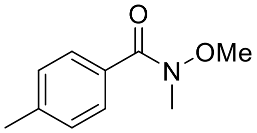

The reaction was performed as described in general procedure B using *N*-(4-bromo-2-nitrophenyl)-4-methylbenzohydrazonyl bromide (**1a**) (100 mg, 242 µmol), triethylamine (170 µl, 1.21 mmol) and *N*,*O*-dimethylhydroxylamine hydrochloride (26.0 mg, 267 µmol). Purification by flash column chromatography using a gradient system (pet. ether/EtOAc, 4:1 to 3:2) gave *N*-methoxy-*N*-methyl-4-methylbenzamide (**3m**) (18.3 mg, 42%) as a yellow oil. R*f* = 0.32 (pet. ether/EtOAc 1:1); ^{1}H NMR (500 MHz, $CDCl_3$) δ 7.59 (d, *J* = 8.1 Hz, 2H), 7.20 (d, *J* = 8.1 Hz, 2H), 3.56 (s, 3H), 3.35 (s, 3H), 2.38 (s, 3H); ^{13}C NMR (126 MHz, $CDCl_3$) δ 170.0, 140.9, 131.1, 128.7 (2 × CH), 128.3 (2 × CH), 61.0, 33.9, 21.5; LCMS (EI) m/z: $[M + H]^+$ Calcd for $C_{10}H_{14}NO_2$ 180.2; Found 180.2 at 7.05 mins. This compound was known in the literature and spectral data are agreed with that of reported values.[8]

1-(4-Methylbenzoyl)piperidine (3n)[9]

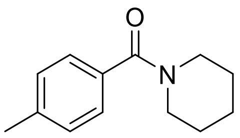

The reaction was performed as described in general procedure B using *N*-(4-bromo-2-nitrophenyl)-4-methylbenzohydrazonyl bromide (**1a**) (207 mg, 504 µmol), triethylamine (348 µl, 2.50 mmol) and piperidine (54.3 µL, 550 µmol). Purification by flash column chromatography (pet. ether/EtOAc 17:3) afforded 1-(4-methylbenzoyl)piperidine (**3n**) (65 mg, 63%) as a red oil. R*f* = 0.18 pet. ether/EtOAc 4:1); ^{1}H NMR (500 MHz, $CDCl_3$, 50 °C) δ 7.29 (d, *J* = 8.1 Hz, 2H), 7.18 (d, *J* = 8.1 Hz, 2H), 3.52 (br s, 4H), 2.36 (s, 3H), 1.71–1.52 (m, 6H); ^{13}C NMR (126 MHz, $CDCl_3$) δ 170.6, 139.6, 133.7, 129.1 (2 × CH), 127.1 (2 × CH), 48.3, 42.7, 26.0, 25.2, 24.1, 20.8.; LCMS (EI) m/z: $[M + H]^+$ Calcd for $C_{13}H_{18}NO$ 204.1; Found 204.2 at 7.85 mins. This compound was known in the literature and spectral data are agreed with that of reported values.[9]

4-(4-Methylbenzoyl)morpholine (3o)[9]

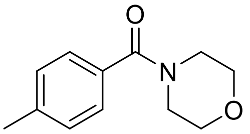

The reaction was performed as described in general procedure B using *N*-(4-bromo-2-nitrophenyl)-4-methylbenzohydrazonyl bromide (**1a**) (207 mg, 504 µmol), triethylamine (348 µl, 2.50 mmol) and morpholine (47.4 µL, 504 µmol). Purification by flash column chromatography (pet. ether/EtOAc 17:3) afforded 4-(4-methylbenzoyl)morpholine (**3o**) (76 mg, 74%) as a red oil. R*f* = 0.18 (pet. ether/EtOAc 7:3); ^{1}H NMR (500 MHz, D_4-MeOD) δ 7.37 (d, *J* = 8.2 Hz, 2H), 7.32 (d, *J* = 8.2 Hz, 2H), 3.92–3.41 (m, 8H), 2.43 (s, 3H); ^{13}C NMR (101 MHz, D_4-MeOD) δ 172.8, 141.7, 133.5, 130.3 (2 × CH), 128.3 (2 × CH), 43.9, 67.8, 21.4; LCMS (EI) m/z: $[M + H]^+$ Calcd for $C_{12}H_{16}NO_2$ 206.1; Found 206.2 at 6.49 mins. This compound was known in the literature and spectral data are agreed with that of reported values.[9]

4-Methylbenzamide (3p)[10]

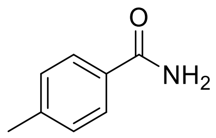

The reaction was performed as described in general procedure B using *N*-(4-bromo-2-nitrophenyl)-4-methylbenzohydrazonyl bromide (**1a**) (100 mg, 240 µmol), triethylamine (170 µL, 1.20 mmol) and NH_3 (20.0 µL, 388 µmol, as a 33% aq. solution). Purification by flash column chromatography a gradient system (pet. ether/EtOAc 7:3 to 1:1) afforded 4-methylbenzamide (**3p**) (20.9 mg, 64%) as a colourless solid. R*f* = 0.50 (EtOAc); ^{1}H NMR (400 MHz, $CDCl_3$) δ 7.71 (d, *J* = 8.2 Hz, 2H), 7.25 (d, *J* = 8.2 Hz, 2H), 6.01 (br s, 2H), 2.41 (s, 3H); ^{13}C NMR (101 MHz, $CDCl_3$) δ 169.5, 142.7, 130.6, 129.4 (2 × CH), 127.5 (2 × CH), 21.6; LCMS (EI) m/z: $[M + H]^+$ Calcd for $C_8H_{10}NO$ 136.1; Found 136.2 at 5.885 mins. This compound was known in the literature and spectral data are agreed with that of reported values.[10]

4-Methyl-*N*-phenylbenzamide (3q)[11]

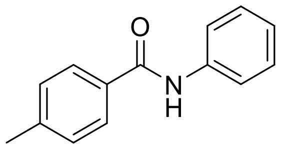

The reaction was performed as described in general procedure B using *N*-(4-bromo-2-nitrophenyl)-4-methylbenzohydrazonyl bromide (**1a**) (207 mg, 504 µmol), triethylamine (348 µl, 2.50 mmol) and aniline (50.2 µL, 550 µmol). Purification by flash column chromatography (pet. ether/EtOAc 17:3) afforded 4-methyl-*N*-phenylbenzamide (**3q**) (40 mg, 38%) as an off-white solid. R*f* = 0.45 (pet. ether/EtOAc 4:1); ^{1}H NMR (500 MHz, $CDCl_3$) δ 7.83 (br s, 1H), 7.77 (d, *J* = 8.2 Hz, 2H), 7.64 (d, *J* = 7.8 Hz, 2H), 7.36 (t, *J* = 7.8 Hz, 2H), 7.28 (d, *J* = 8.Hz, 2H), 7.14 (t, *J* = 7.8 Hz, 1H), 2.42 (s, 3H); ^{13}C NMR (101 MHz, $CDCl_3$) δ 165.8, 142.5, 138.2, 132.3, 129.6 (2 × CH), 129.2 (2 × CH), 127.2 (2 × CH), 124.6, 120.3 (2 × CH), 21.6; LCMS (EI) m/z: $[M + H]^+$ Calcd for $C_{14}H_{14}NO$ 212.1; Found 212.1 at 7.95 mins. This compound was known in the literature and spectral data are agreed with that of reported values.[11]

N-(4-Methoxyphenyl)-4-methylbenzamide (3r)[12]

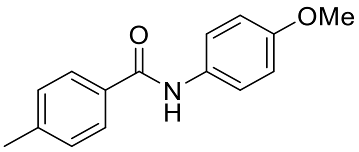

The reaction was performed as described in general procedure B using *N*-(4-bromo-2-nitrophenyl)-4-methylbenzohydrazonyl bromide (**1a**) (207 mg, 504 µmol), triethylamine (348 µL, 2.50 mmol) and 4-methoxyaniline (63.9 µL, 550 µmol). Purification by flash column chromatography (pet. ether/EtOAc 17:3) afforded *N*-(4-methoxyphenyl)-4-methylbenzamide (**3r**) (75.0 mg, 62%) as a brown solid. R*f* = 0.45 (pet. ether/EtOAc 7:3); ^{1}H NMR (500 MHz, $CDCl_3$) δ 7.75 (d, *J* = 8.2 Hz, 2H), 7.73 (br s, 1H), 7.53 (d, *J* = 8.9 Hz, 2H), 7.32 (d, *J* = 8.2 Hz, 2H), 6.92 (d, *J* = 8.9 Hz, 2H), 3.81 (s, 3H), 2.42 (s, 3H); ^{13}C NMR (101 MHz, $CDCl_3$) δ 165.7, 156.7, 142.3, 132.3, 131.3, 129.5 (2 × CH), 127.1 (2 × CH), 122.2 (2 × CH), 114.4 (2 × CH), 55.7, 21.6; LCMS (EI) m/z: $[M + H]^+$ Calcd for $C_{15}H_{16}NO_2$ 242.1; Found 242.1 at 7.90 mins. This compound was known in the literature and spectral data are agreed with that of reported values.[12]

N-Benzyl-4-fluorobenzamide (4a)[13]

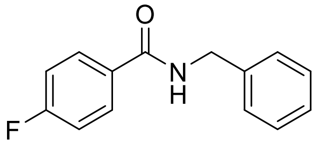

The reaction was performed as described in general procedure B using (*Z*)-*N*-(4-bromo-2-nitrophenyl)-4-fluorobenzohydrazonoyl bromide (**1b**) (100 mg, 241 µmol), triethylamine (170 µL, 1.22 mmol) and benzylamine (30.0 µL, 275 µmol). Purification by flash column chromatography using a gradient system (pet. ether/EtOAc 19:1 to 9:1) afforded *N*-benzyl-4-fluorobenzamide (**4a**) (47.4 mg, 86%) as a colourless solid R*f* = 0.28 (pet. ether/EtOAc 4:1); ^{1}H NMR (500 MHz, $CDCl_3$) δ 7.82–7.78 (m, 2H), 7.39–7.28 (m, 5H), 7.13–7.08 (m, 2H), 6.37 (br s, 1H), 4.46 (2H, d, *J* = 5.6 Hz); ^{13}C NMR (126 MHz, $CDCl_3$) δ 165.6, 164.9 (d, $J_{C\text{-}F}$ = 252.0 Hz), 138.1, 130.6 (d, $J_{C\text{-}F}$ = 3.1 Hz), 129.4 (d, $J_{C\text{-}F}$ = 8.8 Hz), 128.9, 128.0, 127.8, 115.8 (d, $J_{C\text{-}F}$ = 21.8 Hz), 44.4; ^{19}F NMR (471 MHz, $CDCl_3$) δ –108.0 (s); LCMS (EI) m/z: $[M + H]^+$ Calcd for $C_{14}H_{13}FNO$ 230.1; Found 230.1 at 11.05 mins. This compound was known in the literature and spectral data are agreed with that of reported values.[13]

***N*-Benzyl-4-nitrobenzamide (4b)**[13]

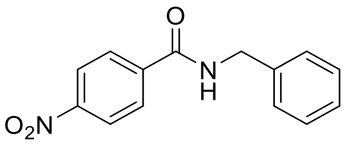

The reaction was performed as described in general procedure B using (*Z*)-*N*-(4-bromo-2-nitrophenyl)-4-nitrobenzohydrazonoyl bromide (**1c**) (111 mg, 251 µmol), triethylamine (174 µL, 1.25 mmol) and benzylamine (30.1 µL, 0.275 mmol). Purification by flash column chromatography (pet. ether/EtOAc 17:3) afforded *N*-benzyl-4-nitrobenzamide (**4b**) (25 mg, 39%) as an orange solid. R*f* = 0.47 (pet. ether/EtOAc 7:3); ^{1}H NMR (400 MHz, $CDCl_3$) δ 8.33–8.25 (m, 2H), 7.97–7.93 (m, 2H), 7.41–7.30 (m, 5H), 6.41 (br s, 1H), 4.67 (d, *J* = 5.7 Hz, 2H); ^{13}C NMR (101 MHz, $CDCl_3$) δ 165.4, 149.8, 140.1, 137.6, 129.1 (2 × CH), 128.3 (2 × CH), 128.2 (2 × CH), 128.2, 124.0 (2 × CH), 44.7; LCMS (EI) m/z: $[M + H]^+$ Calcd for $C_{14}H_{11}N_2O_3$ 255.1; Found 255.1 7.79 mins. This compound was known in the literature and spectral data are agreed with that of reported values.[13]

***N*-Benzyl-4-methoxybenzamide (4c)**[1]

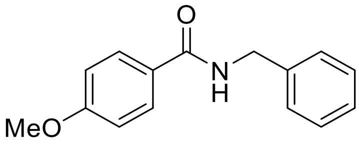

The reaction was performed as described in general procedure B using (*Z*)-*N*-(4-bromo-2-nitrophenyl)-4-methoxybenzohydrazonoyl bromide (**1d**) (111 mg, 260 µmol), triethylamine (181 µL, 1.30 mmol) and benzylamine (31.1 µL, 285 µmol). Purification by flash column chromatography (pet. ether/EtOAc 17:3) afforded *N*-benzyl-4-methoxybenzamide (**4c**) (43 mg, 68%) as an orange solid. R*f* = 0.33 (pet. ether/EtOAc 7:3); ^{1}H NMR (500 MHz, $CDCl_3$) δ 7.76 (d, *J* = 8.8 Hz, 2H), 7.36–7.32 (m, 4H), 7.31–7.26 (m, 1H), 6.90 (d, *J* = 8.8 Hz, 2H), 6.43 (br s, 1H), 4.62 (d, *J* = 5.6 Hz, 2H), 3.84 (s, 3H).; ^{13}C NMR (101 MHz, $CDCl_3$) δ (101 MHz, $CDCl_3$) δ 167.1, 162.4, 138.5, 128.9 (2 × CH), 128.9 (2 × CH), 128.0 (2 × CH), 127.7, 126.8, 113.9 (2 × CH), 55.5, 44.2; LCMS (EI) m/z: $[M + H]^+$ Calcd for $C_{15}H_{16}NO_2$ 242.1; Found 242.2 at 7.54 mins. This compound was known in the literature and spectral data are agreed with that of reported values.[1]

N-Benzyl-4-(methylthio)benzamide (4d)[14]

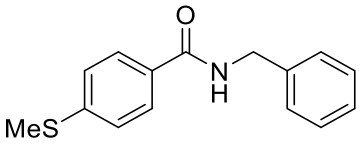

The reaction was performed as described in general procedure B using (*Z*)-*N*-(4-bromo-2-nitrophenyl)-4-(methylthio)benzohydrazonoyl bromide (**1e**) (97.9 mg, 221 µmol), triethylamine (153 µL, 1.10 mmol) and benzylamine (26.2 µL, 240 µmol). Purification by flash column chromatography using a gradient system (pet. ether/EtOAc 19:1 to 9:1) afforded *N*-benzyl-4-(methylsulfanyl)benzamide (**4d**) (36.0 mg, 63%) as a colourless solid. R*f* = 0.18 (pet. ether/EtOAc 4:1); ^{1}H NMR (500 MHz, D_6-DMSO) δ 8.98 (t, *J* = 5.8 Hz, 1H), 7.88–7.81 (d, *J* = 8.6 Hz, 2H), 7.35–7.29 (m, 6H), 7.26–7.21 (1H, 6H), 4.47 (d, *J* = 5.8 Hz, 2H), 2.51 (s, 3H); ^{13}C NMR (126 MHz, D_6-DMSO) δ 165.6, 142.5, 139.7, 130.4, 128.2 (2 × CH), 127.7 (2 × CH), 127.2 (2 × CH), 126.7, 124.9 (2 × CH), 42.5, 14.1; LCMS (EI) m/z: $[M + H]^+$ Calcd for $C_{15}H_{16}NOS$ 258.1; Found 258.1 at 7.98 mins. This compound was known in the literature and spectral data are agreed with that of reported values.[14]

N-Benzyl-2-methylbenzamide (4e)[15]

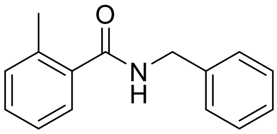

The reaction was performed as described in general procedure B using (*Z*)-*N*-(4-bromo-2-nitrophenyl)-2-methylbenzohydrazonoyl bromide (**1f**) (22.0 mg, 53.5 µmol), triethylamine (37.3 µL, 268 µmol) and benzylamine (6.43 µL, 58.9 µmol). Purification by flash column chromatography (pet. ether/EtOAc 17:3) afforded *N*-benzyl-2-methylbenzamide (**4e**) (12.0 mg, 99%) as an orange solid. R*f* = 0.53 (pet. ether/EtOAc 7:3); ^{1}H NMR (500 MHz, $CDCl_3$) δ 7.39–7.34 (m, 5H), 7.33–7.27 (m, 2H), 7.24–7.16 (m, 2H), 6.05 (br s, 1H), 4.63 (d, *J* = 5.8 Hz, 2H), 2.47 (s, 3H); ^{13}C NMR (101 MHz, $CDCl_3$) δ 170.1, 138.3, 136.4, 136.3, 131.2, 130.1, 129.0 (2 × CH), 128.0 (2 × CH), 127.8, 126.8, 125.9, 44.1, 20.0; LCMS (EI) m/z: $[M + H]^+$ Calcd for $C_{15}H_{16}NO$ 226.3; Found 226.3 at 7.64 mins. This compound was known in the literature and spectral data are agreed with that of reported values.[15]

N-Benzyl-2-chlorobenzamide (4f)[16]

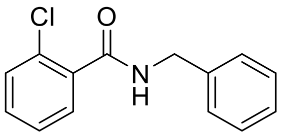

The reaction was performed as described in general procedure B using (*Z*)-*N*-(4-bromo-2-nitrophenyl)-2-chlorobenzohydrazonoyl bromide (**1g**) (230 g, 534 µmol), triethylamine (372 µL, 2.67 mmol) and benzylamine (64.1 µL, 587 µmol). Purification by flash column chromatography (pet. ether/EtOAc 7:3) afforded *N*-benzyl-2-chlorobenzamide (**4f**) (76.0 g, 58%) as a yellow solid. R*f* = 0.17 (pet. ether/EtOAc 4:1); ^{1}H NMR (500 MHz, $CDCl_3$) δ 7.69 (dd, *J* = 7.4, 1.9 Hz, 1H), 7.42–7.27 (m, 8H), 6.50 (br s, 1H), 4.67 (d, *J* = 5.7 Hz, 2H); ^{13}C NMR (126 MHz, $CDCl_3$) δ 166.5, 137.8, 135.1, 131.5, 130.8, 130.4 (2 × CH), 128.9 (2 × CH), 120.0 (2 × CH), 127.8, 127.3, 44.4; LCMS (EI) m/z: $[M + H]^+$ Calcd for $C_{14}H_{13}ClNO$ 246.1; Found 246.2 at 7.70 mins. This compound was known in the literature and spectral data are agreed with that of reported values.[16]

N-Benzyl-2,6-dichlorobenzamide (4g)[17]

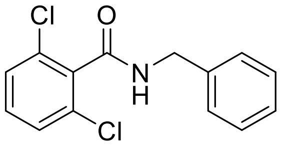

The reaction was performed as described in general procedure B using (*Z*)-*N*-(4-bromo-2-nitrophenyl)-2,6-dichlorobenzohydazonyl bromide (**1h**) (107 mg, 230 µmol), triethylamine (160 µL, 1.15 mmol) and benzylamine (27.6 µL, 253 µmol). Purification by flash column chromatography (pet. ether/EtOAc 7:3) afforded *N*-benzyl-2,6-dichlorobenzamide (**4g**) (49.6 mg, 77%) as a colourless solid. R*f* = 0.35 (pet. ether/EtOAc 7:3); ^{1}H NMR (500 MHz, $CDCl_3$) δ 7.39–7.23 (m, 7H), 7.23–7.18 (m, 1H), 6.05 (br s, 1H), 4.63 (d, *J* = 5.7 Hz, 2H); ^{13}C NMR (126 MHz, $CDCl_3$) δ 164.5, 137.4, 136.0, 132.4 (2 × C), 130.8, 128.8 (2 × CH), 128.2 (2 × CH), 128.2 (2 × CH), 127.9, 44.2; LCMS (EI) m/z: $[M + H]^+$ Calcd for $C_{14}H_{12}Cl_2NO$ 280.1; Found 280.1 at 8.10 mins. This compound was known in the literature and spectral data are agreed with that of reported values.[17]

N-Benzyl-3-phenylpropanamide (4h)[18]

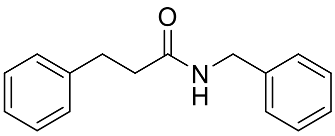

The reaction was performed as described in general procedure B using (*Z*)-*N*-(4-bromo-2-nitrophenyl)-3-phenylpropanehydrazonoyl bromide (**1i**) (555 mg, 1.31 mmol), triethylamine (913 µL, 6.55 mmol) and benzylamine (157 µL, 1.44 mmol). Purification by flash column chromatography (pet. ether/EtOAc 7:3) afforded *N*-benzyl-3-phenylpropanamide (**4h**) (155mg, 49%) as an orange solid. R*f* = 0.21 (pet. ether/EtOAc 7:3); ^{1}H NMR (500 MHz, D_6-DMSO) δ 8.31 (t, *J* = 5.8 Hz, 1H), 7.30–7.25 (m, 4H), 7.23–7.17 (m, 4H), 7.16–7.12 (m, 2H), 4.25 (d, *J* = 5.8 Hz, 2H), 2.85 (t, *J* = 7.8 Hz, 2H), 2.46 (t, *J* = 7.8 Hz, 2H); ^{13}C (101 MHz, D_6-DMSO) δ 171.2, 141.3, 139.5, 128.3 (2 × CH), 128.2 (2 × CH), 128.2 (2 × CH), 127.1 (2 × CH), 126.6, 125.9, 41.9, 36.9, 31.1; LCMS (EI) m/z: $[M + H]^+$ Calcd for $C_{16}H_{18}NO$ 240.3; Found 240.3 at 7.83 mins. This compound was known in the literature and spectral data are agreed with that of reported values.[18]

N-Benzylbutyramide (4i)[18]

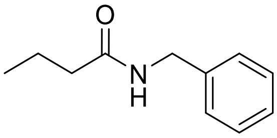

The reaction was performed as described in general procedure B using (*Z*)-*N*-(4-bromo-2-nitrophenyl)butyrohydrazonoyl bromide (**1j**) (181 mg, 500 µmol), triethylamine (348 µL, 2.50 mmol) and benzylamine (60.1 µL, 550 µmol). Purification by flash column chromatography (pet. ether/EtOAc 7:3) afforded *N*-benzylbutyramide (**4i**) (83.0 mg, 94%) as an orange solid. R*f* = 0.26 (pet. ether/EtOAc 7:3) ^{1}H NMR (400 MHz, D_6-DMSO) δ 8.27 (br s, 1H), 7.34–7.28 (m, 2H), 7.26–7.19 (m, 3H), 4.26 (d, *J* = 6.0 Hz, 2H), 2.11 (t, *J* = 7.4 Hz, 2H), 1.54 (sext, *J* = 7.4 Hz, 2H), 0.86 (t, *J* = 7.4 Hz, 3H); ^{13}C NMR (101 MHz, D_6-DMSO) δ 171.9, 139.8, 128.2 (2 × CH), 127.1 (2 × CH), 126.6, 41.9, 37.3, 18.7, 13.6; LCMS (EI) m/z: $[M + H]^+$ Calcd for $C_{11}H_{16}NO$ 178.1; Found 178.2 at 6.91 mins. This compound was known in the literature and spectral data are agreed with that of reported values.[18]

N-Benzylcyclohexanecarboxamide (4j)[19]

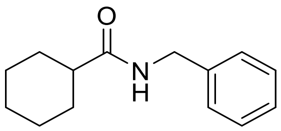

The reaction was performed as described in general procedure B using (*Z*)-*N*-(4-bromo-2-nitrophenyl)cyclohexanecarbohydrazonoyl bromide (**1k**) (121 mg, 300 µmol), triethylamine (209 µL, 1.50 mmol) and benzylamine (36.0 µL, 330 µmol). Purification by flash column chromatography (pet. ether/EtOAc 4:1) afforded *N*-benzylcyclohexanecarboxamide (**4j**) (42.5 mg, 65%) as a yellow solid R*f* = 0.24 (pet. ether/EtOAc 7:3); ^{1}H NMR (500 MHz, $CDCl_3$) δ 7.34–7.30 (m, 2H), 7.29–7.23 (m, 3H), 5.87 (br s, 1H), 4.42 (d, *J* = 5.6 Hz, 2H), 2.11 (tt, *J* = 11.8, 3.4 Hz, 1H), 1.93–1.74 (m, 4H), 1.72–1.63 (m, 1H), 1.52–1.41 (m, 2H), 1.31–1.18 (m, 3H); ^{13}C NMR (126 MHz, $CDCl_3$) δ 176.1, 138.7, 128.8 (2 × CH), 127.8 (2 × CH), 127.5, 45.7, 43.5, 29.8 (2 × CH_2) , 25.8 (3 × CH_2); LCMS (EI) m/z: [M + H]$^+$ Calcd for $C_{14}H_{20}NO$ 218.1; Found 218.2 at 8.03 mins. This compound was known in the literature and spectral data are agreed with that of reported values.[19]

N-Benzylpivalamide (4k)[20]

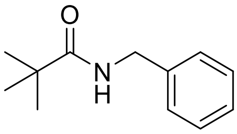

The reaction was performed as described in general procedure B using (*Z*)-*N*-(4-bromo-2-nitrophenyl)pivalohydrazonoyl bromide (**1l**) (155 mg, 410 µmol), triethylamine (286 µL, 2.05 mmol) and benzylamine (49.3 µL, 451 µmol). Purification by flash column chromatography (pet. ether/EtOAc 7:3) afforded *N*-benzylpivalamide (**4k**) (53.2 mg, 63%) as a yellow oil. R*f* = 0.28 (pet. ether/EtOAc 7:3); ^{1}H NMR (500 MHz, $CDCl_3$) δ 7.28–7.22 (m, 2H), 7.21–7.15 (m, 3H), 5.92 (br s, 1H), 4.35 (d, *J* = 5.7 Hz, 2H), 1.15 (s, 9H); ^{13}C NMR (126 MHz, $CDCl_3$) δ 178.4, 138.8, 128.8 (2 × CH), 127.7 (2 × CH), 127.5, 43.7, 38.8, 27.7 (3 × CH_3); LCMS (EI) m/z: [M + H]$^+$ Calcd for $C_{12}H_{18}NO$ 192.3; Found 192.3 at 7.51 mins. This compound was known in the literature and spectral data are agreed with that of reported values.[20]

N-Benzyltetrahydrofuran-3-carboxamide (4l)[21]

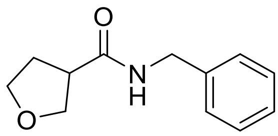

The reaction was performed as described in general procedure B using (*Z*)-*N*-(4-bromo-2-nitrophenyl)tetrahydrofuran-3-carbohydrazonyl bromide (**1m**) (51.8 mg, 133 µmol), triethylamine (92.7 µL, 665 µmol) and benzylamine (16.0 µL, 146 µmol). Purification by flash column chromatography using a gradient system (CH_2Cl_2/EtOAc, 7:3 to 1:1) afforded *N*-benzyltetrahydrofuran-3-carboxamide (**4l**) (18.2 mg, 67%) as a yellow oil. R*f* = 0.15 (pet. ether/EtOAc 7:3); ^{1}H NMR (500 MHz, $CDCl_3$) δ 7.37–7.27 (m, 5H), 5.90 (br s, 1H), 4.45 (d, *J* = 5.7 Hz, 2H), 3.99–3.88 (m, 3H), 3.81 (q, *J* = 7.5 Hz, 1H), 2.96–2.88 (m, 1H), 2.25–2.12 (m, 2H); ^{13}C NMR (126 MHz, $CDCl_3$) δ 173.7, 138.2, 128.9 (2 × CH), 127.9 (2 × CH), 127.8, 71.0, 68.3, 45.7, 43.8, 30.6; LCMS (EI) m/z: $[M + H]^+$ Calcd for $C_{12}H_{16}NO_2$ 206.1; Found 206.1 at 7.29 mins. This compound was known in the literature and spectral data are agreed with that of reported values.[21]

N-Benzyl-5-(4-bromophenyl)-3-methylisoxazole-4-carboxamide (4m)

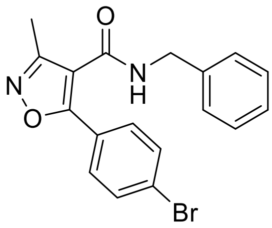

The reaction was performed as described in general procedure B using (*Z*)-*N*-(4-bromo-2-nitrophenyl)-5-(4-bromophenyl)-3-methylisoxazole-4-carbohydrazonyl bromide (**1n**) (100 mg, 180 µmol), triethylamine (125 µL, 900 µmol) and benzylamine (21.5 µL, 197 µmol). Purification by flash column chromatography (CH_2Cl_2/EtOAc, 9:1) gave *N*-benzyl-5-(4-bromophenyl)-3-methylisoxazole-4-carboxamide (**4m**) (45.1 mg, 68%) as an off-white solid. R*f* = 0.28 (pet. ether/EtOAc 7:3); m.p 132–134 °C; IR vmax (solid) 3293, 2932, 1641, 1586, 1144, 1011, 829, 700, 677 cm^{-1}^{1}H NMR (400 MHz, $CDCl_3$) δ 7.48–7.40 (m, 4H), 7.31–7.23 (m, 3H), 7.20–7.15 (m, 2H), 5.85 (br s, 1H), 4.47 (d, *J* = 5.9 Hz, 2H), 2.37 (s, 3H); ^{13}C NMR (126 MHz, $CDCl_3$) δ 166.7, 161.8, 160.0, 137.3, 132.5 (2 × CH), 129.5 (2 × CH), 129.0 (2 × CH), 128.2 (2 × CH), 128.1, 125.8, 125.5, 112.3, 44.1, 11.2; HRMS (ESI) m/z: $[M + H]^+$ Calcd for $C_{18}H_{16}BrN_2O_2$ 371.0390; Found 371.0387.

4-Chloro-*N*-(2-morpholinoethyl)benzamide, moclobemide (4n)[22]

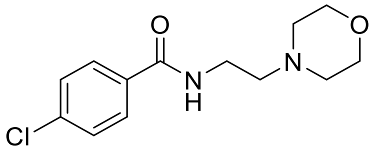

The reaction was performed as described in general procedure B using (*Z*)-*N*-(4-bromo-2-nitrophenyl)-4-chlorobenzohydrazonoyl bromide (**1o**) (200 mg, 464 µmol), triethylamine (323 µL, 2.32 mmol) and 4-(2-aminoethyl)morpholine (66.7 µL, 508 µmol). Purification by flash column chromatography (EtOAc/MeOH 9:1) afforded 4-chloro-*N*-(2-morpholinoethyl)benzamide, moclobemide (**4n**) (62.9 mg, 51%) as an off-white solid. R*f* = 0.26 (EtOAc/MeOH 9:1); ^{1}H NMR (500 MHz, $CDCl_3$) δ 7.70 (d, *J* = 8.4 Hz, 2H), 7.39 (d, *J* = 8.4 Hz, 2H), 6.79 (br s, 1H), 3.70 (t, *J* = 4.5 Hz, 4H), 3.52 (app q, *J* = 5.7 Hz, 2H), 2.58 (t, *J* = 6.0 Hz, 2H), 2.48 (t, *J* = 4.3, 4H); ^{13}C NMR (126 MHz, $CDCl_3$) δ 166.4, 137.7, 133.1, 128.9 (2 × CH), 128.5 (2 × CH), 67.1 (2 × CH_2), 56.9, 53.4 (2 × CH_2), 36.2; LCMS (EI) m/z: $[M + H]^+$ Calcd for $C_{13}H_{18}ClN_2O_2$ 269.1; Found 269.2 at 6.49 mins. This compound was known in the literature and spectral data are agreed with that of reported values.[22]

***N*-[2-(diethylamino)ethyl]-4-nitrobenzamide (4o)**[23]

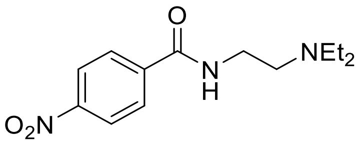

The reaction was performed as described in general procedure B using (*Z*)-*N*-(4-bromo-2-nitrophenyl)-4-nitrobenzohydrazonoyl bromide (**1c**) (100 mg, 226 µmol), triethylamine (160 µL, 1.15 mmol) and *N*,*N*-diethylethylenediamine (35.1 µL, 250 µmol). Purification by flash column chromatography (CH_2Cl_2/MeOH 9:1) afforded *N*-[2-(diethylamino)ethyl]-4-nitrobenzamide (**4o**) (12.7 mg, 21%) as a viscous yellow oil. R*f* = 0.33 (CH_2Cl_2/MeOH 9:1); ^{1}H NMR (500 MHz, $CDCl_3$) δ 8.29 (d, *J* = 8.8 Hz, 2H), 7.98 (d, *J* = 8.8 Hz, 2H), 7.45 (br s, 1H), 3.56 (app q, *J* = 5.4 Hz, 2H), 2.76 (t, *J* = 5.4 Hz, 2H), 2.67 (q, *J* = 7.2 Hz, 4H), 1.10 (t, *J* = 7.2 Hz, 6H); ^{13}C NMR (126 MHz, $CDCl_3$) δ 165.4, 149.7, 140.3, 128.3 (2 × CH), 123.9 (2 × CH), 51.5, 47.1 (2 × CH_2), 37.3, 11.6 (2× CH_3); LCMS (EI) m/z: $[M + H]^+$ Calcd for $C_{13}H_{20}N_3O_3$ 266.3; Found 266.2 at 5.54 mins. This compound was known in the literature and spectral data are agreed with that of reported values.[23]

Methyl pentanoyl-*L*-valinate (4p)

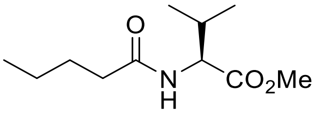

The reaction was performed as described in general procedure B using *N*-(4-bromo-2-nitrophenyl)pentanehydazonyl bromide (**1p**) (202 mg, 536 µmol), triethylamine (374 µL, 2.68 mmol) and *L*-valine methyl ester hydrochloride (98.0 mg, 585 µmol). Purification by flash column chromatography (CH_2Cl_2/EtOAc, 9:1) afforded methyl pentanoyl-*L*-valinate (**4p**) (86.2 g, 75%) as a yellow oil. R*f* = 0.28 (CH_2Cl_2/EtOAc 9:1); IR vmax (neat) 3302, 2959, 2934, 1744, 1647, 1533, 1261, 1200, 752 cm^{-1}; ^{1}H NMR (500 MHz, $CDCl_3$) δ 5.90 (br d, *J* = 8.8 Hz, 1H), 4.59 (dd, *J* = 8.8, 4.9 Hz, 1H), 3.74 (s, 3H), 2.25 (t, *J* = 7.7 Hz, 2H), 2.19–2.11 (m, 1H), 1.67–1.60 (m, 2H), 1.36 (sext. *J* = 7.3 Hz, 2H), 0.96–0.89 (m, 9H); ^{13}C NMR (126 MHz, $CDCl_3$) δ 173.2, 172.9, 57.0, 52.3, 36.6, 31.5, 27.9, 22.5, 19.1, 18.0, 13.9; HRMS (ESI) m/z: $[M + H]^+$ Calcd for $C_{11}H_{22}NO_3$ 216.1594; Found 216.1591; $[\alpha]_D^{25}$ = –50.0 (*c* 0.05, MeOH).

4-Chloro-*N*-(4-hydroxyphenethyl)benzamide (4q)[24]

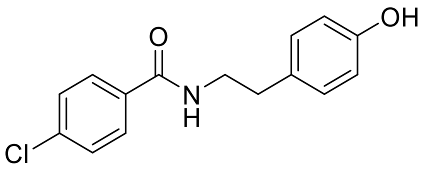

The reaction was performed as described in general procedure B using (*Z*)-*N*-(4-bromo-2-nitrophenyl)-4-chlorobenzohydazonyl bromide (**1o**) (150 mg, 348 µmol), triethylamine (241 µL, 1.73 mmol) and tyramine (53.0 mg, 386 µmol). Purification by flash column chromatography using a gradient system (CH_2Cl_2 to CH_2Cl_2/MeOH 98:2) gave 4-chloro-*N*-(4-hydroxyphenethyl)benzamide (**4q**) (68.4 mg, 71%) as a brown solid. R*f* = 0.60 (CH_2Cl_2/MeOH 9:1); ^{1}H NMR (500 MHz, D_6-DMSO) δ 9.15 (s, 1H), 8.59 (t, *J* = 5.6 Hz, 1H), 7.83 (d, *J* = 8.6 Hz, 2H), 7.53 (d, *J* = 8.6 Hz, 2H), 7.01 (d, *J* = 8.4 Hz, 2H), 6.67 (d, *J* = 8.4 Hz, 2H), 3.44–3.38 (m, 2H), 2.71 (t, *J* = 7.8 Hz, 2H); ^{13}C NMR (126 MHz, D_6-DMSO) δ 165.0, 156.6, 135.8, 133.4, 129.5 (2 × CH), 129.0 (2 × CH), 128.3 (2 × CH), 115.1 (2 × CH), 41.3, 34.2; LCMS (EI) m/z: $[M + H]^+$ Calcd for $C_{15}H_{15}ClNO_2$ 276.1; Found 276.1 at 7.46 mins. This compound was known in the literature and spectral data are agreed with that of reported values.[24]

Isopropyl 2-(4-(2-(4-chlorobenzamido)ethyl)phenoxy)-2-methylpropanoate (S2)

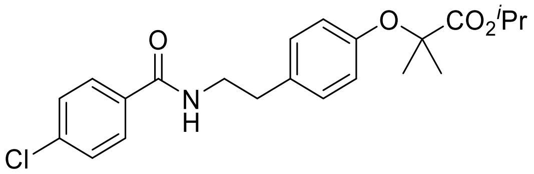

To a solution of 4-chloro-*N*-(4-hydroxyphenethyl)benzamide (**4q**) (125 mg, 454 µmol) in acetonitrile (10 mL) was added potassium carbonate (188 mg, 1.36 mmol) and the mixture was stirred at room temperature for 10 minutes. Isopropyl 2-bromo-2-methylpropanoate (90.1 µL, 543 µmol) was added and the reaction mixture was stirred under reflux for 16 h. A further portion of isopropyl 2-bromo-2-methylpropanoate (90.1 µL, 543 µmol) and potassium carbonate (62.6 mg, 453 µmol) were added and the mixture was stirred under reflux for 6 h. The reaction mixture was cooled to room temperature and quenched by the addition of 1 M aq. HCl (10 mL). The mixture was extracted with CH_2Cl_2 (3 × 20 mL) and the combined organic extracts were washed with brine (50 mL), dried over $MgSO_4$, filtered and concentrated *in vacuo*. Purification by column chromatography using a gradient system (pet. ether/EtOAc, 4:1 to 3:2) gave isopropyl 2-(4-(2-(4-chlorobenzamido)ethyl)phenoxy)-2-methylpropanoate (**S2**) (113 mg, 62%) as a colourless oil. R*f* = 0.20 (pet. ether/EtOAc 4:1); IR (neat) 3314, 2982, 2936, 1726, 1638, 1539, 1508, 1485, 1282, 1234, 1177, 1148, 1101, 847, 756 cm^{-1}; ^{1}H NMR (500 MHz, $CDCl_3$) δ 7.60 (d, *J* = 8.5 Hz, 2H), 7.34 (d, *J* = 8.5 Hz, 2H), 7.05 (d, *J* = 8.5 Hz, 2H), 6.78 (d, *J* = 8.5 Hz, 2H), 6.27 (br s, 1H), 5.06 (hept., *J* = 6.3 Hz, 1H), 3.62 (app q, *J* = 6.9 Hz, 2H), 2.83 (t, *J* = 7.0 Hz, 2H), 1.56 (s, 6H), 1.20 (d, *J* = 6.3 Hz, 6H); ^{13}C NMR (126 MHz, $CDCl_3$) δ 173.8, 166.5, 154.3, 137.7, 133.1, 132.3, 129.5 (2 × CH), 128.9 (2 × CH), 128.4 (2 × CH), 119.5 (2 × CH), 79.2, 69.0, 41.4, 34.8, 25.5 (2 × CH_3), 21.7 (2 × CH_3); HRMS (ESI) m/z: $[M + H]^+$ Calcd for $C_{22}H_{27}ClNO_4$ 404.1610; Found 404.1606.

2-(4-(2-(4-Chlorobenzamido)ethyl)phenoxy)-2-methylpropanoic acid, bezafibrate (5)[25]

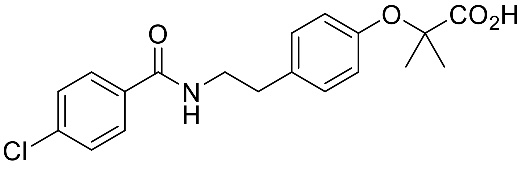

To a solution of isopropyl 2-(4-(2-(4-chlorobenzamido)ethyl)phenoxy)-2-methylpropanoate (**S2**) (113 mg, 280 µmol) in THF (10 mL) was added 4 M aq. NaOH (2 mL) and the resulting mixture was stirred at reflux for 8 h. Further portions of NaOH (100 mg, 2.50 mmol) were

added at 2 h intervals throughout the course of the reaction. The reaction mixture was cooled to room temperature and concentrated *in vacuo*. The residue was dissolved in water (20 mL) and concentrated HCl was added dropwise. The resulting precipitate was filtered, washed with water and dried under high vacuum to afford 2-(4-(2-(4-chlorobenzamido)ethyl)phenoxy)-2-methylpropanoic acid, bezafibrate (**5**) (88.3 mg, 87%) as an off-white solid. R*f* = 0.20 (CH_2Cl_2/MeOH 9:1); ^{1}H NMR (500 MHz, D_6-DMSO) δ 12.96 (br s, 1H), 8.63 (t, *J* = 5.6 Hz, 1H), 7.83 (d, *J* = 8.4 Hz, 2H), 7.53 (d, *J* = 8.4 Hz, 2H), 7.12 (d, *J* = 8.3 Hz, 2H), 6.75 (d, *J* = 8.3 Hz, 2H), 3.43 (app q, *J* = 6.7 Hz, 2H), 2.76 (t, *J* = 7.4 Hz, 2H), 1.48 (s, 6H); ^{13}C NMR (126 MHz, D_6-DMSO) δ 175.1, 165.1, 153.6, 135.8, 133.3, 132.6, 129.3 (2 × CH), 129.2 (2 × CH), 128.3 (2 × CH), 118.5 (2 × CH), 78.3, 41.0, 34.1, 25.0 (2 × CH_3); LCMS (EI) m/z: $[M + H]^+$ Calcd for $C_{19}H_{21}ClNO_4$ 362.1; Found 362.1 at 6.30 mins. This compound was known in the literature and spectral data are agreed with that of reported values.[25]

(±)-(4-Methylbenzoyl)alanine (6a)

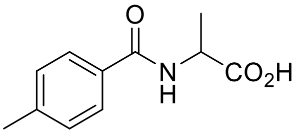

The reaction was performed as described in general procedure B using *N*-(4-bromo-2-nitrophenyl)-4-methylbenzohydrazonyl bromide (**1a**) (103 mg, 250 µmol), triethylamine (174 µl, 1.25 mmol) and DL-alanine (33.0 mg, 370 µmol). Purification by flash column chromatography (CH_2Cl_2/MeOH 19:1) afforded (±)-(4-methylbenzoyl)alanine (**6a**) (25.0 mg, 48%) as an off-white solid. R*f* = 0.31 (CH_2Cl_2/MeOH 9:1); m.p. 172–174 °C; IR vmax (solid) 3317, 3030, 2998, 2925, 1726, 1630, 1616, 1539, 1506, 1458. 1215, 1191, 1173, 757 cm^{-1}; ^{1}H NMR (500 MHz, D_6-DMSO) δ 12.49 (br s, 1H), 8.55 (d, *J* = 7.3 Hz, 1H), 7.80 (d, *J* = 8.1 Hz, 2H), 7.28 (d, *J* = 8.1 Hz, 2H), 4.41 (p, *J* = 7.4 Hz, 1H), 2.36 (s, 3H), 1.39 (d, *J* = 7.4 Hz, 3H); ^{13}C NMR (126 MHz, D_6-DMSO) δ 174.3, 166.0, 141.2, 131.1, 128.7 (2 × CH), 127.4 (2 × CH), 48.1, 20.9, 16.9; HRMS (ESI) m/z: $[M + H]^+$ Calcd for $C_{11}H_{14}NO_3$ 208.0974; Found 208.0968.

(±)-(4-Methylbenzoyl)phenylalanine (6b)

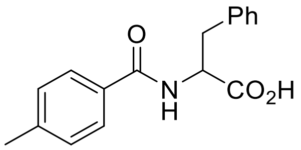

The reaction was performed as described in general procedure B using *N*-(4-bromo-2-nitrophenyl)-4-methylbenzohydrazonyl bromide (**1a**) (30.0 mg, 73.0 µmol), triethylamine (50.9 µL, 365 µmol) and DL-phenylalanine (13.0 mg, 78.7 µmol) in H_2O (500 µL) was added. Purification of the residue by reverse phase column chromatography on a SB-C18 column using a gradient system (H_2O (0.1% TFA)/MeCN (0.1% TFA); 5–95%) afforded (±)-(4-methylbenzoyl)phenylalanine (**6b**) (9.00 mg, 44%) as a colourless oil. R*f* = 0.31 (CH_2Cl_2 /MeOH 9:1); IR vmax (film) 3355, 3269, 2925, 2854, 1716, 1610, 1532, 1502, 1350, 1217, 1191, 1173, 1033, 918, 838, 752, 703, 685 cm^{-1}; ^1H NMR (500 MHz, D_6-DMSO) δ 12.7 (br s, 1H), 8.58 (d, *J* = 8.2 Hz, 1H), 7.70 (d, *J* = 8.2 Hz, 2H), 7.32–7.23 (m, 6H), 7.19–7.15 (m, 1H), 4.61 (ddd, *J* = 10.6, 8.2, 4.5 Hz, 1H), 3.18 (dd, *J* = 13.8, 4.5 Hz, 1H), 3.07 (dd, *J* = 13.8, 10.6 Hz, 1H), 2.35 (s, 3H); ^{13}C NMR (126 MHz, D_6-DMSO) δ 173.2, 166.2, 141.2, 138.2, 131.1, 129.0 (2 × CH), 128.7 (2 × CH), 128.1 (2 × CH), 127.3 (2 × CH), 126.3, 54.1, 36.2, 20.9; HRMS (ESI) m/z: $[M + H]^+$ Calcd for $C_{17}H_{18}NO_3$ 284.1273; Found 284.1280.

(±)-(4-Methylbenzoyl)valine (6c)

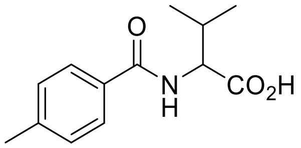

The reaction was performed as described in general procedure B using *N*-(4-bromo-2-nitrophenyl)-4-methylbenzohydrazonyl bromide (**1a**) (103 mg, 250 µmol), triethylamine (174 µl, 1.25 mmol) and DL-valine (43.3 mg, 370 µmol). Purification by flash column chromatography (CH_2Cl_2/MeOH 19:1) afforded (±)-(4-methylbenzoyl)valine (**6c**) (40 mg, 68%) as an off-white solid. R*f* = 0.52 (CH_2Cl_2/MeOH 9:1); IR vmax (solid) 3444, 2967, 2921, 2952, 1705, 1646, 1424, 1536, 1504, 1363, 1227, 756 cm^{-1}; ^1H NMR (400 MHz, D_6-DMSO) δ 12.58 (br s, 1H), 8.34 (d, *J* = 8.2 Hz, 1H), 7.80 (d, *J* = 8.1 Hz, 2H), 7.27 (d, *J* = 8.1 Hz, 2H), 4.25 (dd, *J* = 8.2, 7.1 Hz, 1H), 2.36 (s, 3H), 2.23–2.13 (m, 1H), 0.95 (app t, *J* = 7.4 Hz, 6H); ^{13}C NMR (101 MHz, D_6-DMSO) δ 173.3, 166.7, 141.3, 131.3, 128.8 (2 × CH), 127.7 (2 × CH), 58.4,

29.5, 21.0, 19.4, 18.9; HRMS (ESI) m/z: $[M + H]^+$ Calcd for $C_{13}H_{18}NO_3$ 236.1287 Found 236.1281.

(2*S*,3*S*)-3-Methyl-2-(4-methylbenzamido)pentanoic acid (6d)

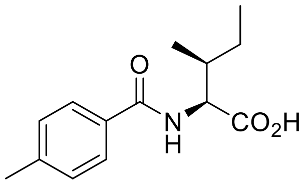

The reaction was performed as described in general procedure B using *N*-(4-bromo-2-nitrophenyl)-4-methylbenzohydrazonyl bromide (**1a**) (103 mg, 250 µmol), triethylamine (174 µl, 1.25 mmol) and L-isoleucine (42.0 mg, 320 µmol). Purification by flash column chromatography (CH_2Cl_2/MeOH 19:1) afforded (2*S*,3*S*)-3-methyl-2-(4-methylbenzamido)pentanoic acid (**6d**) (38.0 mg, 61%) as a colourless oil. R*f* = 0.54 (CH_2Cl_2/MeOH 9:1); IR vmax (neat) 3400, 3300, 2970, 2918, 2882, 1647, 1533, 1501, 1362, 1221, 1026, 765 cm^{-1}; ^1H NMR (500 MHz, CD_3OD) 7.74 (d, *J* = 8.2 Hz, 2H), 7.29 (d, *J* = 8.2 Hz, 2H), 7.26 (br s, 1H), 4.55 (d, *J* = 6.4 Hz, 1H), 2.40 (s, 3H), 2.08–1.97 (m, 1H), 1.67–1.56 (m, 1H), 1.38–1.28 (m, 2H), 1.03 (d, *J* = 7.1 Hz, 3H), 0.97 (t, *J* = 7.5 Hz, 3H); ^{13}C NMR (126 MHz, CD_3OD) 175.1, 170.5, 132.6, 130.1 (2 × CH), 128.6 (2 × CH), 127.3, 58.8, 38.2, 26.6, 21.4, 16.1, 11.7;HRMS (ESI) m/z: $[M + H]^+$ Calcd for $C_{14}H_{20}NO_3$ 250.1430; Found 250.1436; $[\alpha]_D^{25}$ = +84.6 (*c* 0.05, MeOH).

The methodology was also carried out using racemic 2-amino-3-methylpentanoic acid and a (1:1) mixture of diastereomers was obtained. The spectral data for racemic (±)-3-methyl-2-(4-methylbenzamido)pentanoic acid (**(±)-6d**) is provided below.

^1H NMR (500 MHz, D_6-DMSO) δ 12.6 ((2*S*,3*S* + 2*R*,3*R)*, br s, 1H; (2*R*,3*S* + 2*S*,3*R),* br s, 1H)), 8.29 ((2*S*,3*S* + 2*R*,3*R)*, d, *J* = 8.1 Hz, 1H), 8.17 ((2*R*,3*S* + 2*S*,3*R)*, d, *J* = 8.1 Hz, 1H), 7.79 ((2*S*,3*S* + 2*R*,3*R)*, d, *J* = 8.2 Hz, 2H; (2*R*,3*S* + 2*S*,3*R)*, d, *J* = 8.2 Hz, 2H), 7.27 ((2*S*,3*S* + 2*R*,3*R)*, d, *J* = 8.2 Hz, 2H; (2*R*,3*S* + 2*S*,3*R)*, d, *J* = 8.2 Hz, 2H), 4.52 ((2*R*,3*S* + 2*S*,3*R)*, dd, *J* = 8.1, 5.7 Hz, 1H), 4.32 ((2*S*,3*S* + 2*R*,3*R)*, apt. t, *J* = 7.5 Hz, 1H), 2.36 ((2*S*,3*S* + 2*R*,3*R)*, s, 3H; (2*R*,3*S* + 2*S*,3*R)*, s, 3H), 2.05–1.89 ((2*S*,3*S* + 2*R*,3*R)*, m, 1H; (2*R*,3*S* + 2*S*,3*R)*, m, 1H), 1.57–1.35 ((2*S*,3*S* + 2*R*,3*R)*, m, 1H; (2*R*,3*S* + 2*S*,3*R)*, m, 1H), 1.32–1.14 ((2*S*,3*S* + 2*R*,3*R)*, m, 1H; (2*R*,3*S* + 2*S*,3*R)*, m, 1H), 0.97–0.90 ((2*S*,3*S* + 2*R*,3*R)*, m, 3H; (2*R*,3*S* + 2*S*,3*R)*, m, 3H), 0.90–0.84 ((2*S*,3*S* + 2*R*,3*R)*, m, 3H; (2*R*,3*S* + 2*S*,3*R)*, m, 3H); ^{13}C NMR (126 MHz, D_6-

DMSO) δ 172.4, 172.2, 166.3, 166.1, 140.5, 131.3, 131.2, 128.1 (2 × CH), 126.9 (2 × CH), 126.9 (2 ×CH), 56.0, 55.7, 35.9, 35.7, 25.3, 24.7, 20.3, 15.1, 14.5, 10.7, 10.4.

(2*S*)-(4-Methylbenzoyl)proline (6e)

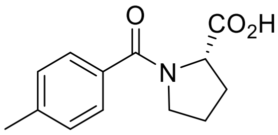

The reaction was performed as described in general procedure B using *N*-(4-bromo-2-nitrophenyl)-4-methylbenzohydrazonyl bromide (**1a**) (103 mg, 250 µmol), triethylamine (174 µl, 1.25 mmol) and L-proline (43.2 mg, 375 µmol). Purification by flash column chromatography (CH_2Cl_2/MeOH 19:1) afforded (2*S*)-(4-methylbenzoyl)proline (**6e**) (29.0 mg, 50%) as a colourless oil. R*f* = 0.43 (CH_2Cl_2/MeOH 9:1); IR vmax (neat) 3444, 2967, 2921, 2952, 1705, 1646, 1424, 1536, 1504, 1363, 1227, 756 cm^{-1}; ^{1}H NMR (500 MHz, D_6-DMSO, 100 °C) δ 7.38 (d, *J* = 6.8 Hz, 2H), 7.21 (d, *J* = 6.8 Hz, 2H), 5.66 (s, 1H), 4.37 (s, 1H), 3.52 (s, 2H), 2.36 (s, 3H), 2.25–2.14 (m, 1H), 2.00–1.78 (m, 3H); ^{13}C NMR (126 MHz, D_6-DMSO, 100 °C) δ 172.7, 169.6, 128.1 (2 × CH), 126.5, 126.4, 120.2, 112.1, 59.1, 48.1, 20.3, 20.0, 13.5; HRMS (ESI) m/z: $[M + H]^+$ Calcd for $C_{13}H_{15}NO_3$ 234.1130; Found 234.1128; $[\alpha]_D^{25}$ = –21.5 (*c* 0.05, MeOH).

(2*S*)-2-[(4-Methylphenyl)formamido]pentanedioic acid (6f)

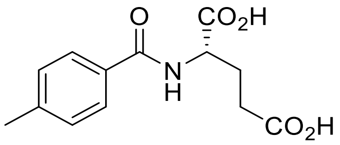

The reaction was performed as described in general procedure B using *N*-(4-bromo-2-nitrophenyl)-4-methylbenzohydrazonyl bromide (**1a**) (30.0 mg, 73.0 µmol), triethylamine (50.9 µL, 365 µmol) and L-glutamic acid (55.2 mg, 375 µmol). The reaction mixture was cooled to room temperature and concentrated *in vacuo*. The residue was diluted with EtOAc (10 mL) and washed with aq. 2 M NaOH (10 mL of a 2 M solution in H_2O). The aqueous layer was acidified with aq. HCl (3 mL of a 2 M solution in H_2O), extracted with CH_2Cl_2 (3 × 10 mL), dried over $MgSO_4$, filtered and concentrated *in vacuo*. Purification of the residue by reverse phase column chromatography on a SB-C18 column using a gradient system (H_2O (0.1% TFA)/MeCN (0.1% TFA); 5–95%) afforded (2*S*)-2-[(4-methylphenyl)formamido]pentanedioic acid (**6f**) (7.00 mg, 36%) as a colourless oil. R*f* = 0.19 (CH_2Cl_2/MeOH 9:1); IR vmax (film)

2956, 2928, 2859, 1646, 1616, 1541, 1508, 1415, 1191, 1148, 1040, 1027, 1113, 839, 802, 757, 726 cm^{-1}; ^{1}H NMR (500 MHz, D_6-DMSO) δ 8.50 (d, *J* = 7.6 Hz, 1H), 7.80 (d, *J* = 8.2 Hz, 2H), 7.28 (d, *J* = 8.2 Hz, 2H), 4.39 (ddd, *J* = 9.8, 7.6, 4.9 Hz, 1H), 2.36 (s, 3H), 2.35–2.33 (m, 2H), 2.13 2.04 (m, 1H), 2.00–1.90 (m, 1H); ^{13}C NMR (126 MHz, D_6-DMSO) δ 174.9, 173.4, 166.4, 141.3, 131.1, 128.7 (2 × CH), 127.4 (2 × CH), 51.9, 30.4, 25.9, 21.0; HRMS (ESI) m/z: Calcd for $C_{13}H_{16}NO_5$ $[M+H]^+$ 266.1015; Found 266.1021; $[\alpha]_D^{25}$ = +85.4 (*c* 0.05, MeOH).

4. References

1. Singh, A.; Narula, A. K. *New. J. Chem*., **2021**, *45*, 7486–7490.

2. Dai, C.; Genovino, J.; Bechle, B. M.; Corbett, M. S.; Huh, C. -W.; Rose, C. R.; Sun, J.; Warmus, J. S.; Blakemore, D. C.; *Org. Lett*., **2017**, *19*, 1064–1067.

3. Li, Z.-L.; Sun, K.-K.; Cai, C. *Org. Lett*., **2018**, *20*, 6420–6424.

4. Zhu, L.; Le, L.; Yan, M.; Au, C.-T.; Qiu, R.; Kambe, N. *J. Org. Chem*. **2019**, *84*, 5635–5644.

5. Reddy, K. R.; Maheswari, C. U.; Venkateshwar, M.; Kantam, M. L. *Eur. J. Org. Chem*., **2008**, *2008*, 3619–3622.

6. Gross, U.; Koos, P.; O'Brien, M.; Polyzos, A.; Ley, S. V.; *Eur. J. Org. Chem*., **2014**, *2014*, 6418–6430.

7. Jahn, A.; Kim, S. Y.; Choi, J.-H.; Kim, D.-D.; Ahn, Y.-J.; Yong, C. S.; Kim, J. S. *J. Pharm. Pharmacol*., **2010**, *62*, 91–97.

8. Yedage, S. L.; Bhanage, B. M.; *Synthesis*, **2015**, *47*, 526–532.

9. Jo, Y.; Ju, J.; Choe, J.; Song, K. H.; Lee, S. *J. Org. Chem*., **2009**, *74*, 6358–6361.

10. Govindan, K.; Lin, W.-Y. *Org. Lett*., **2021**, *23*, 1600–1605.

11. Han, K.-J.; Seok Tae, B.; Kim, M. *Org. Prep. Proced. Int.,* **2009**, *37*, 198–203.

12. Yao, Z.; Wei, X. *Chin. J. Chem*., **2010**, *28*, 2260–2268.

13. Nikitas, N. F.; Apostolopoulou, M. K.; Skolia, E.; Tsoukaki, A.; Kokotos, C. G.; *Chem. Eur. J*., **2021**, *27*, 7915–7922.

14. Movahed, F.-S.; Sawant, D. N.; Bagal, D. B.; Saito, S. *Synthesis*, **2020**, 52, 3253–3262.

15. Huang, Z.; Reilly, J. E.; Buckle, R. N., *Synlett*., **2007**, *2007*, 1026–1030.

16. Sawant, D. N.; Bagal, D. B.; Ogawa, S.; Selvam, K.; Saito, S. *Org. Lett*., **2018**, *20*, 4397–4400.

17. Cheng, M.-C.; Lo, W.-C.; Chang, Y.-W.; Lee, S-S.; Chang, C.-C. *Bioorg. Chem*., **2020**, *104*, 104166.

18. Allen, C. L.; Davulcu, S.; Williams, J. M. J., *Org. Lett*., **2010**, *12*, 5096–5099.

19. Gaspa, S.; Farina, A.; Tilocca, M.; Porcheddu, A.; Pisano, L,; Carraro, M.; Azzena, U.; and De Luca, L. *J. Org. Chem*., **2020**, *85*, 11679–11687.

20. Molander, G. A.; Beaumard, F.; *Org. Lett*., **2011**, *13*, 1242–1245.

21. Veeraraghavan Ramachandran, P.; Hamann, H. J. *Org. Lett*., **2021**, *23*, 2938–2942.

22. Kumar, V.; Connon, S. J. *Chem. Commun*., **2017**, *53*, 10212–10215.

23. Braddock, D. C.; Lickiss, P. D.; Rowley, B. C.; Pugh, D.; Purnomo, T.; Santhakumar, G.; Fussell, S. J.; *Org. Lett.,* **2018**, *20*, 950–953.

24. Umair Tariq, M.; Moran, W. J. *Eur. J. Org. Chem*., **2020**, 5153–5160.

25. Liu, M,; Wang, S.; Qu, C.; Zhang, Z.; Qu, Y. *J. Chem. Eng. Data*., **2020**, *65*, 2156–2169.

5. ¹H and ¹³C NMR Spectra for all Compounds

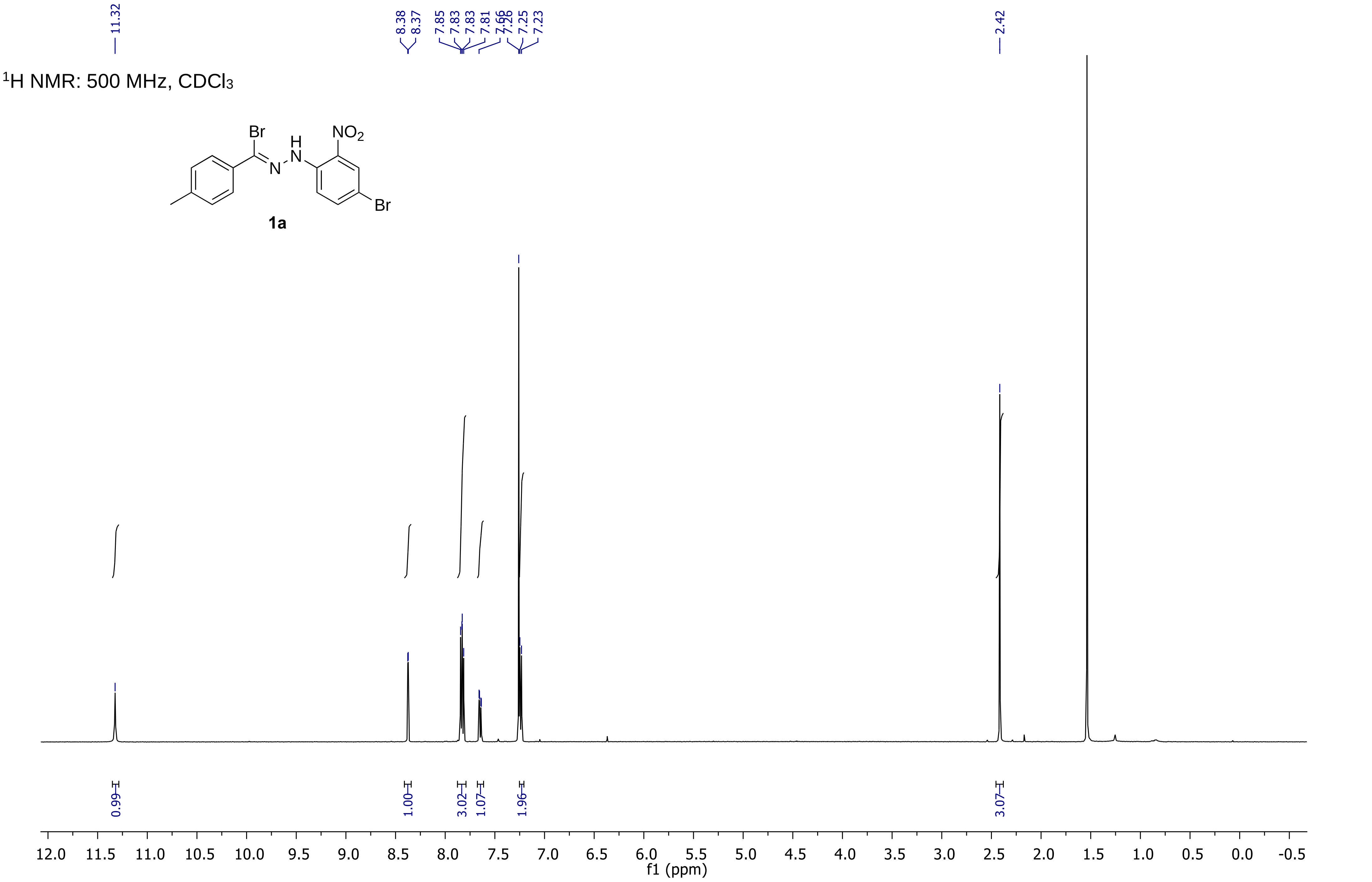

$^{13}C\{^1H\}$ NMR: 101 MHz, $CDCl_3$

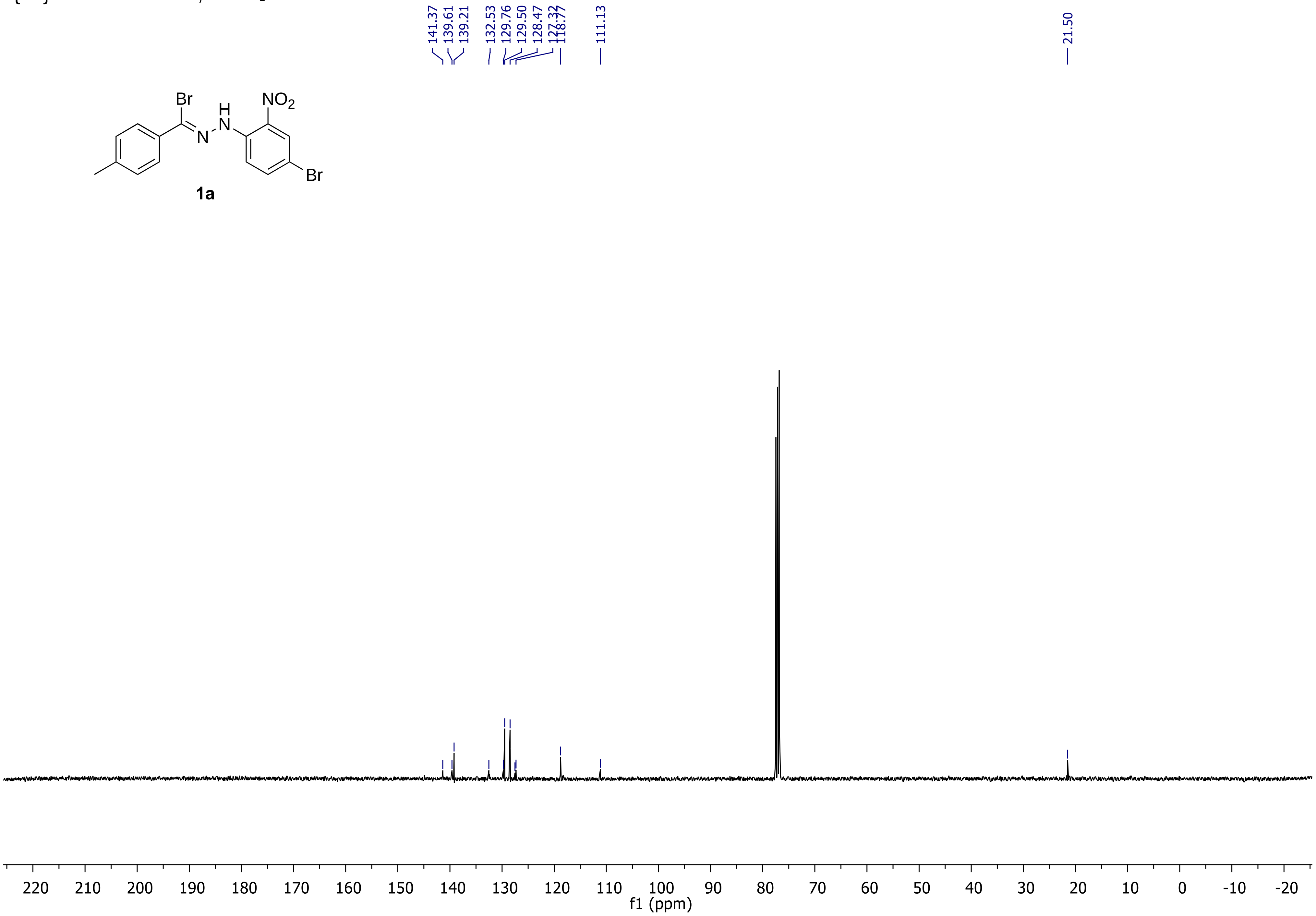

^{1}H NMR: 500 MHz, $CDCl_3$

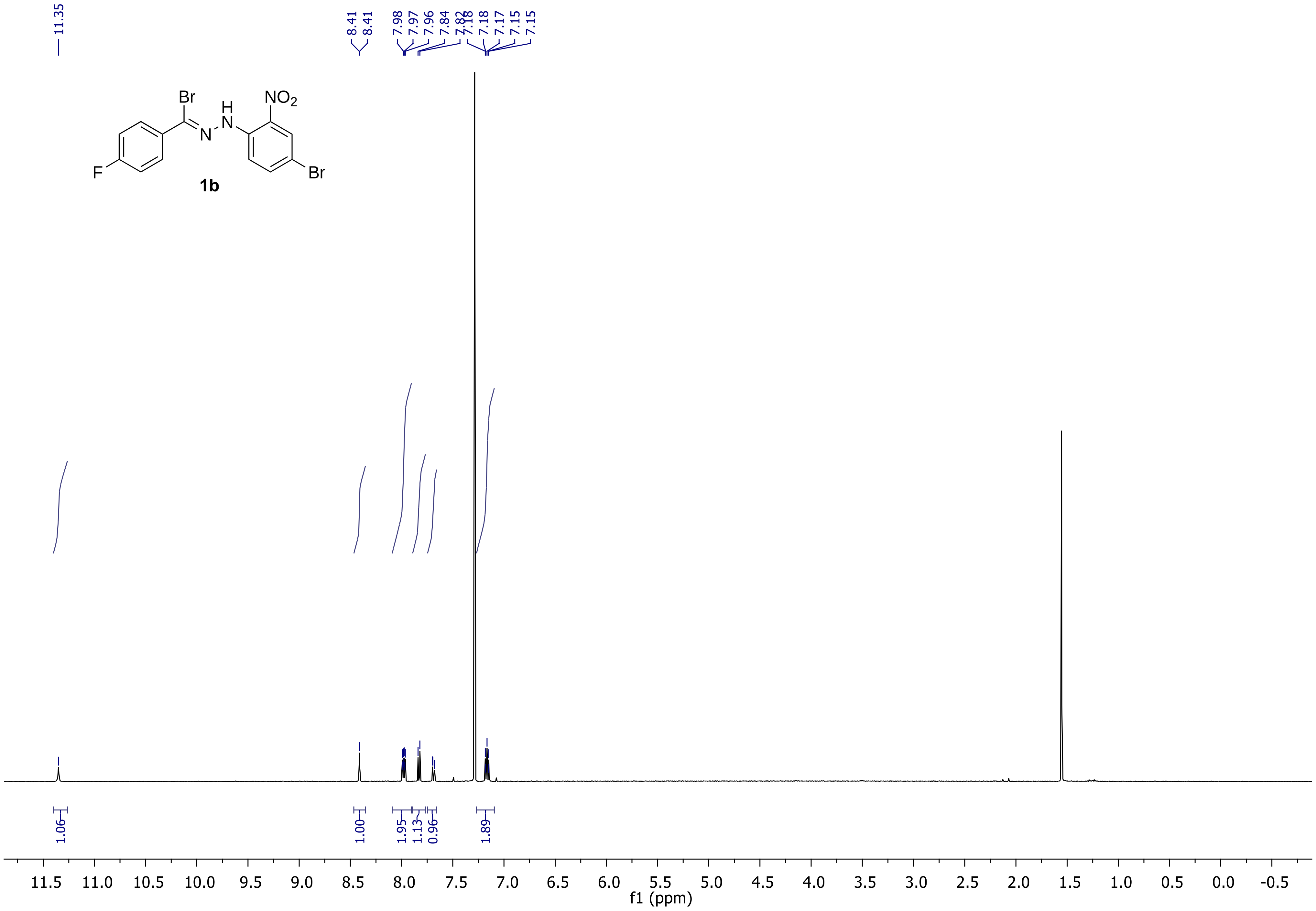

$^{13}C\{^1H\}$ NMR: 126 MHz, $CDCl_3$

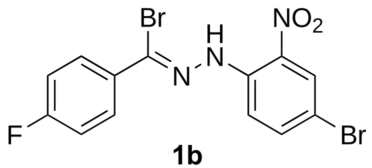

1b

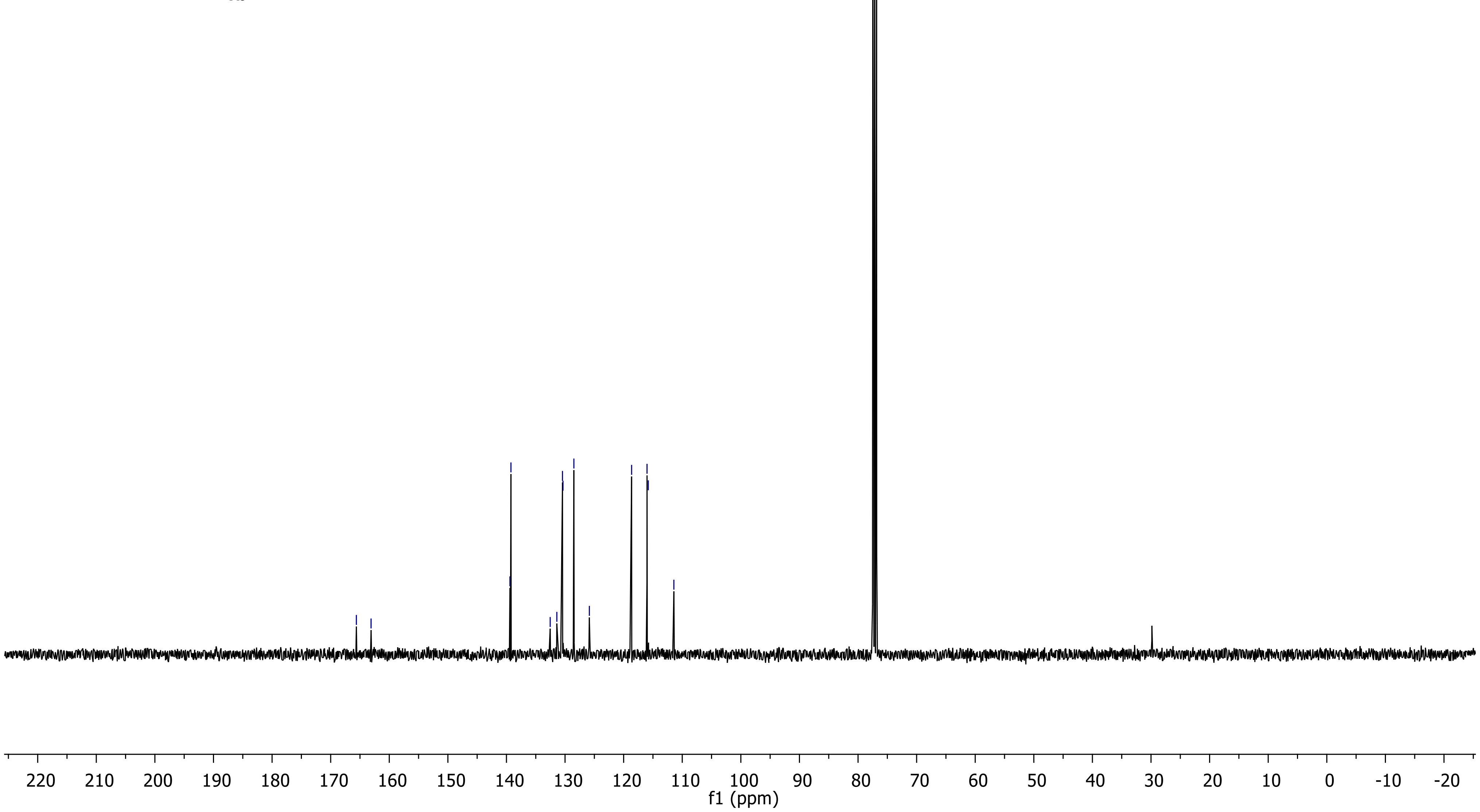

^1H NMR: 500 MHz, $CDCl_3$

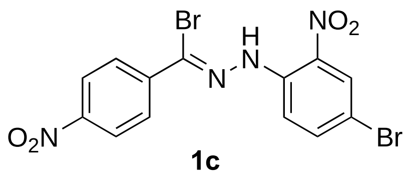

1c

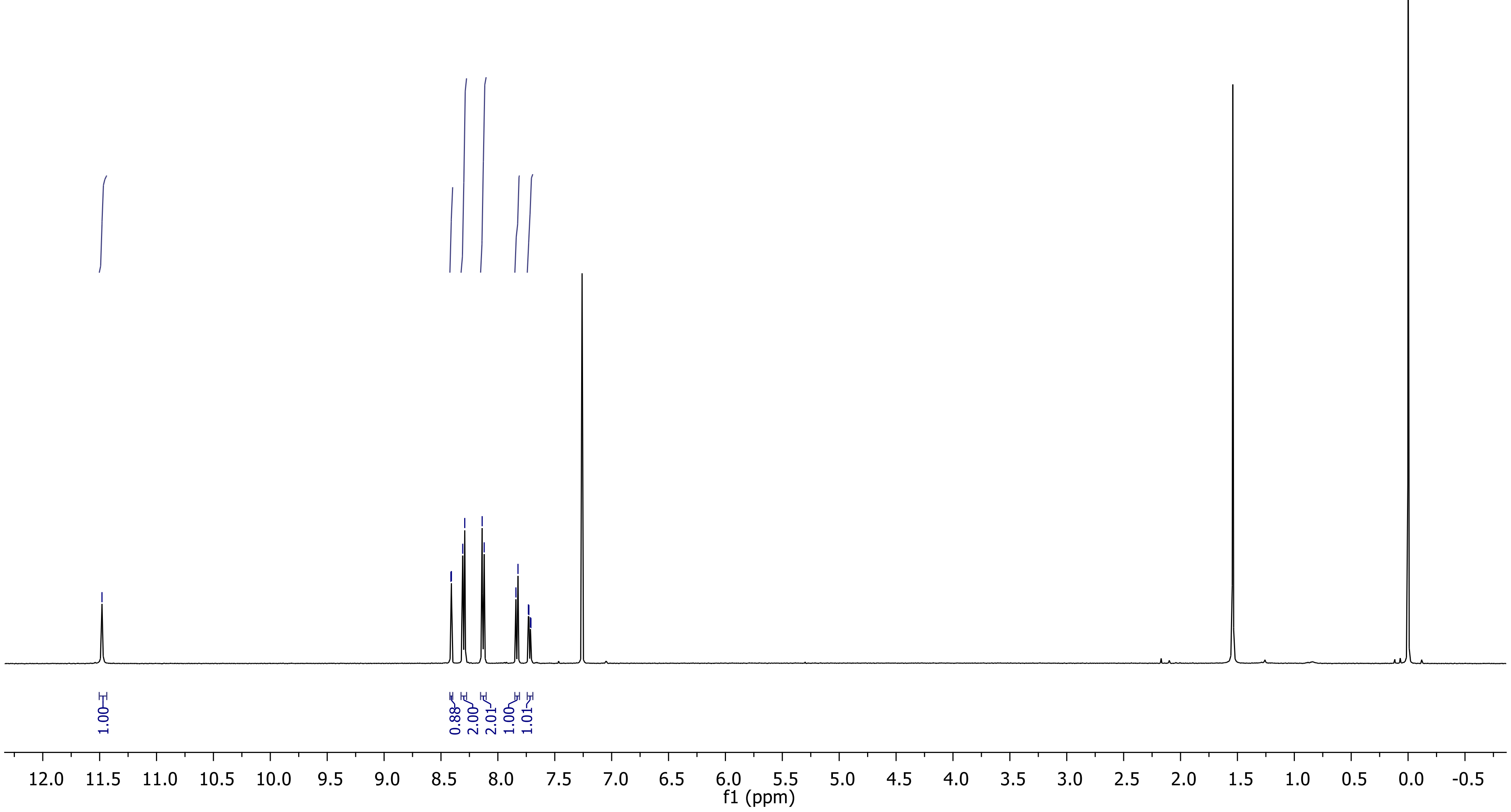

$^{13}C\{^{1}H\}$ NMR: 101 MHz, $CDCl_3$

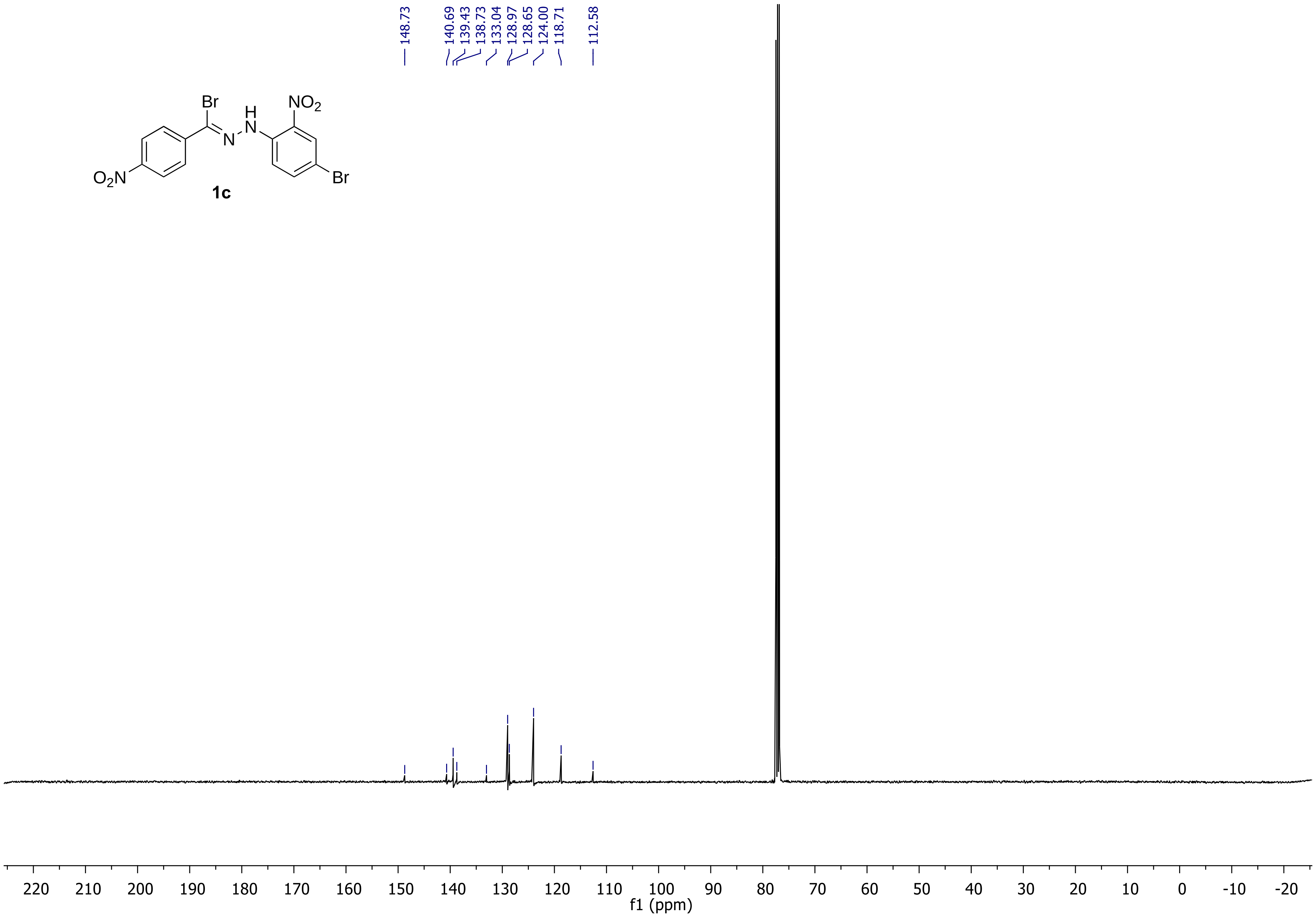

^{1}H NMR: 500 MHz, $CDCl_3$

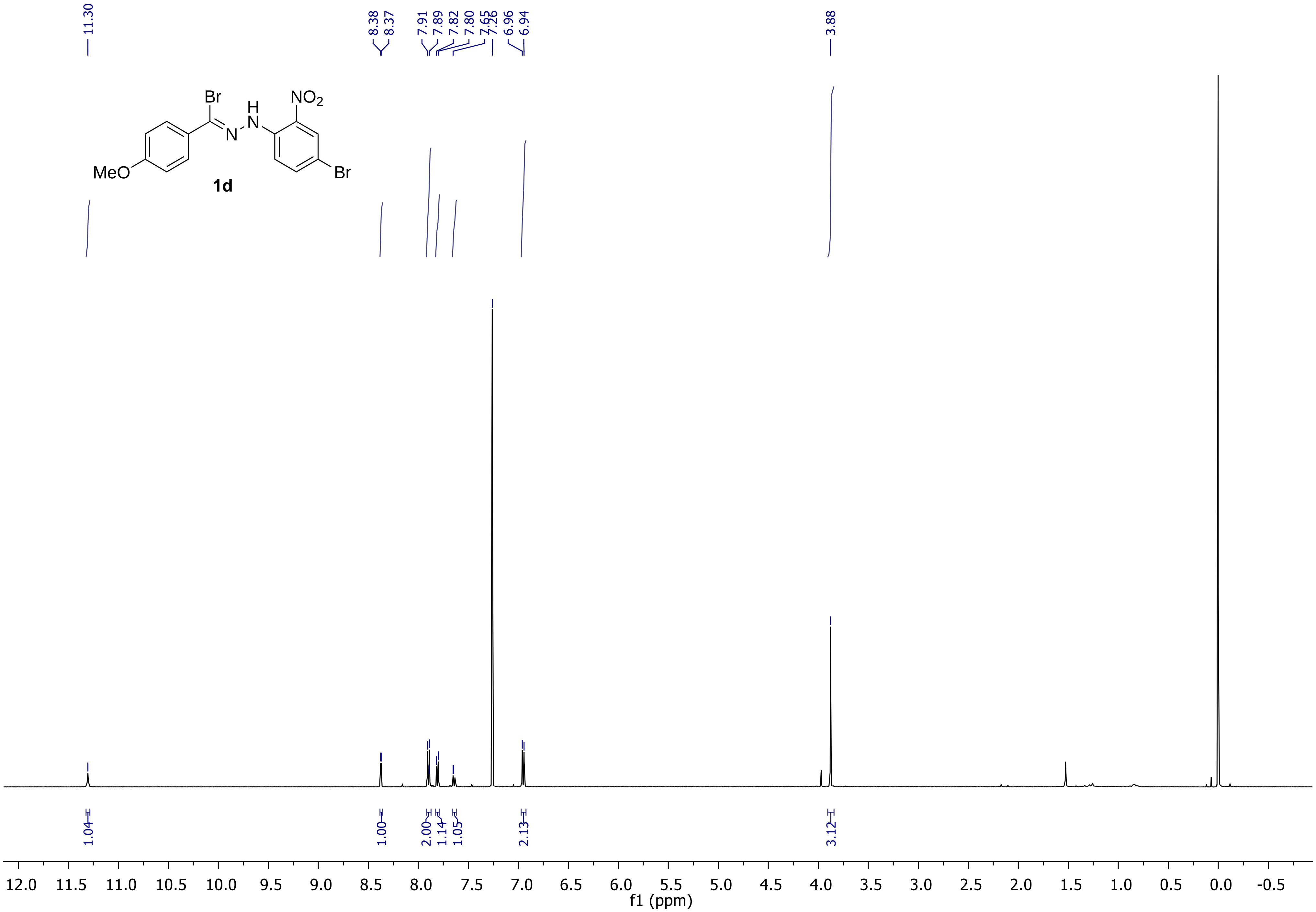

$^{13}C\{^1H\}$ NMR: 101 MHz, $CDCl_3$

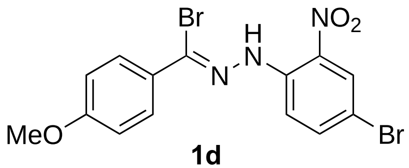

1d

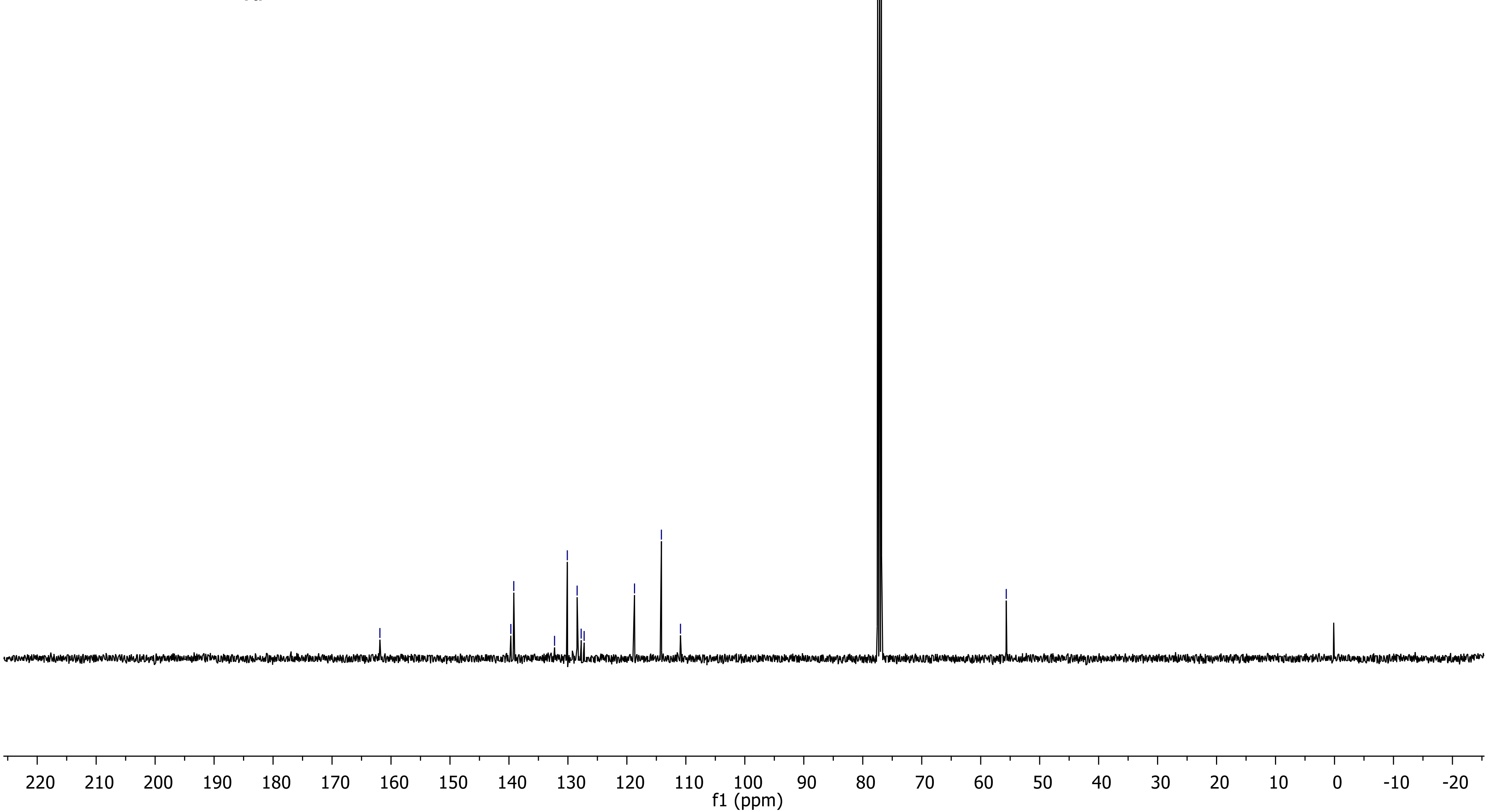

^{1}H NMR: 500 MHz, $CDCl_3$

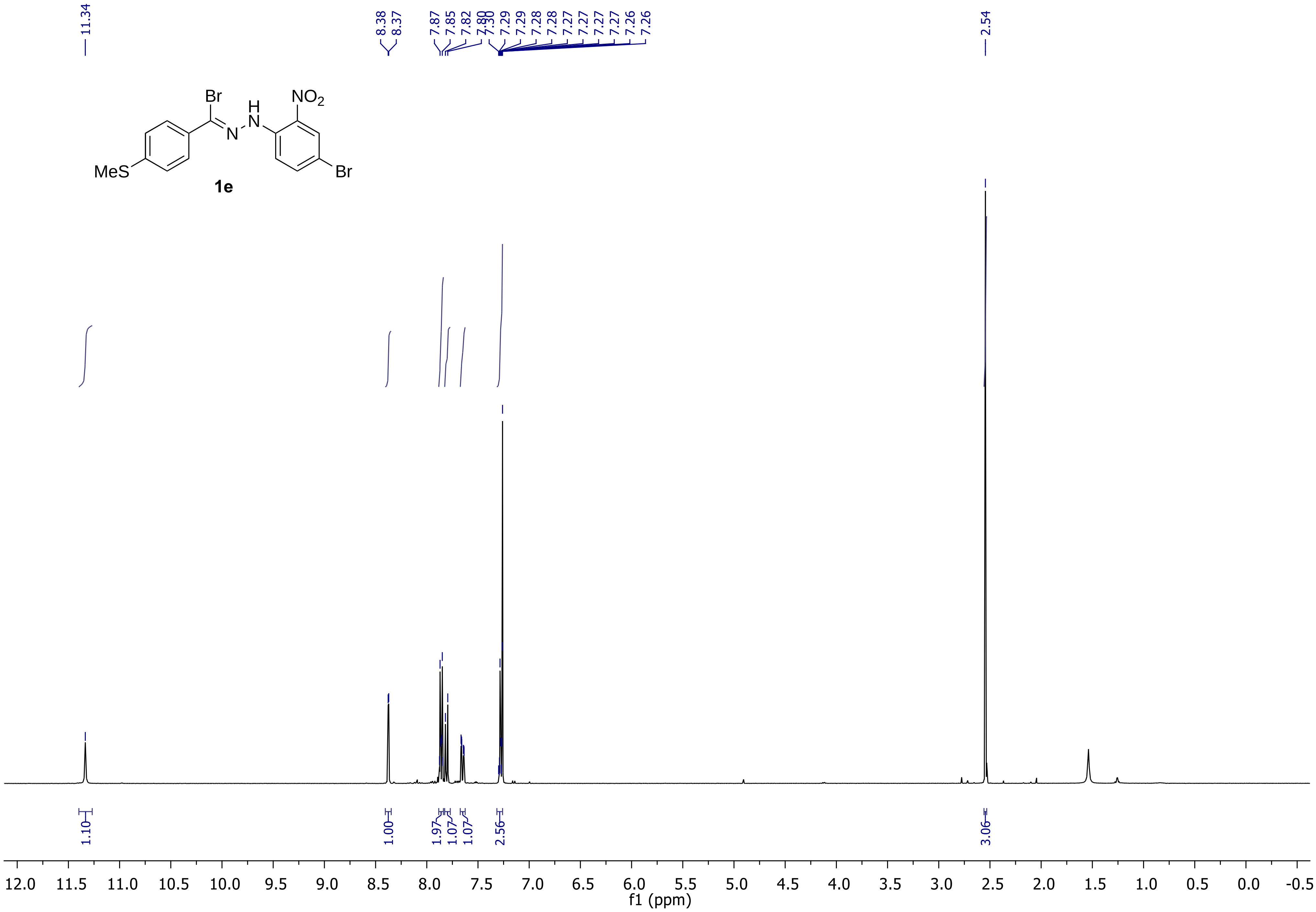

$^{13}C\{^1H\}$ NMR: 101 MHz, $CDCl_3$

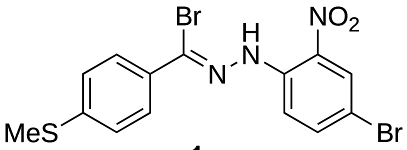

1e

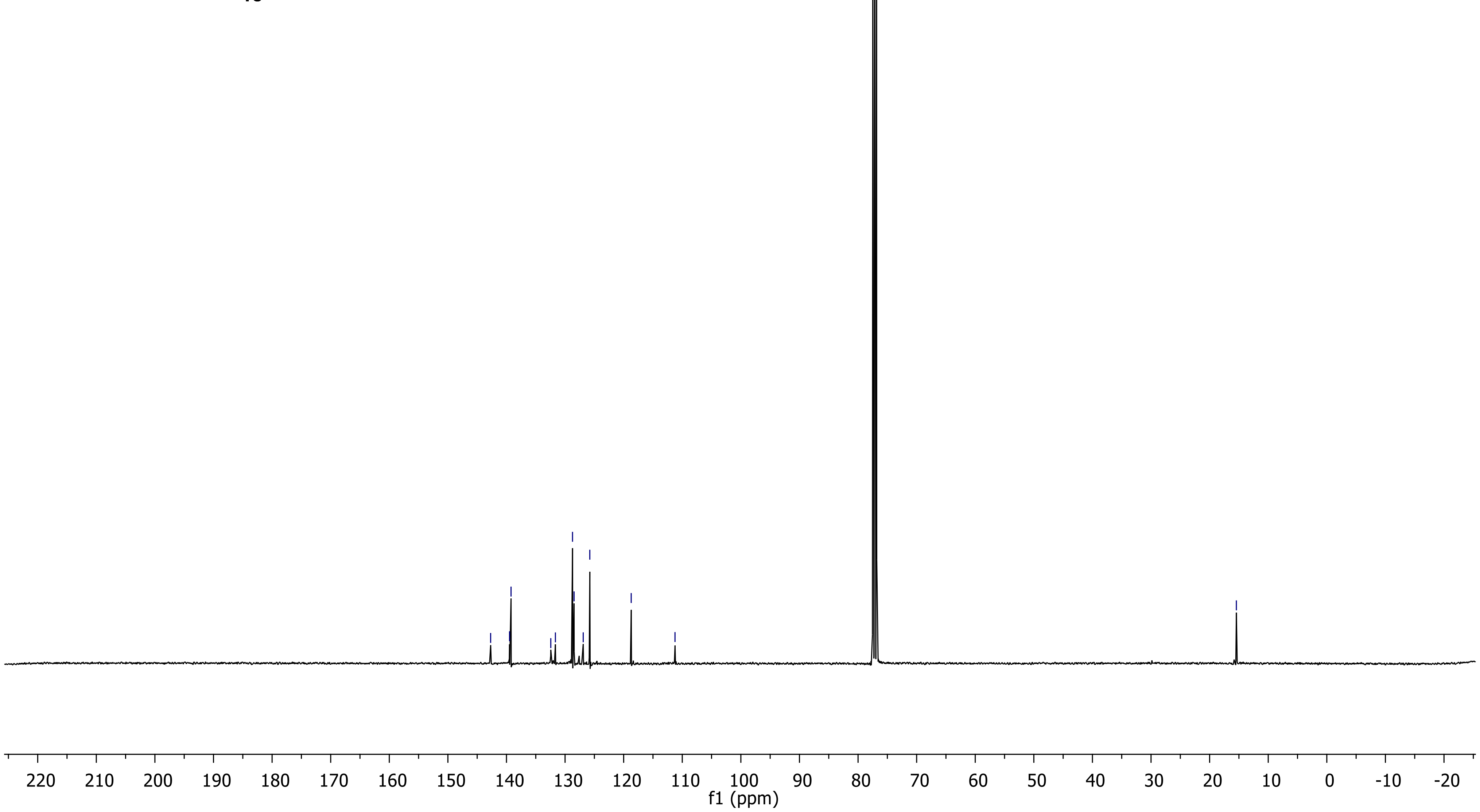

^1H NMR: 500 MHz, $CDCl_3$

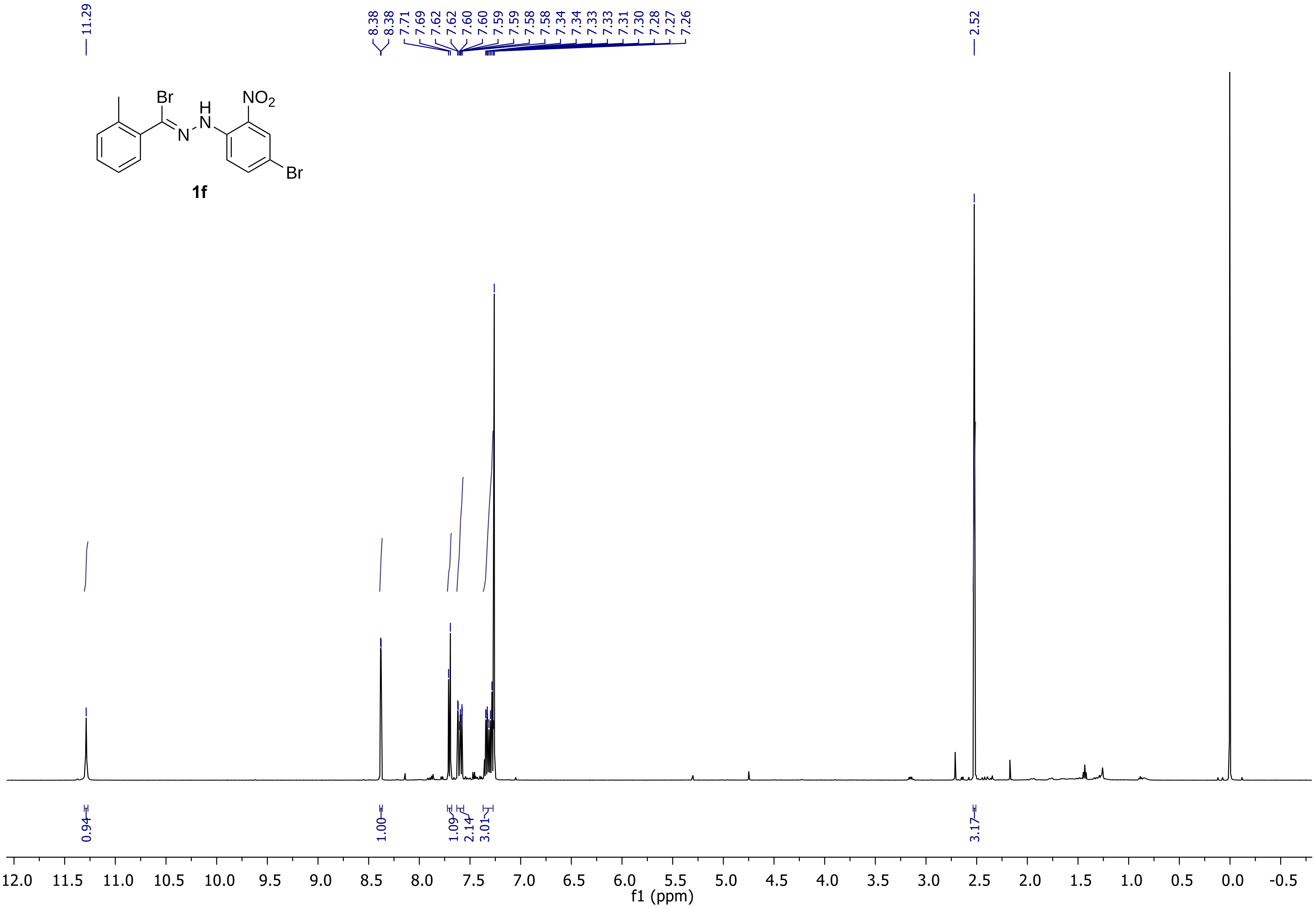

$^{13}C\{^1H\}$ NMR: 101 MHz, $CDCl_3$

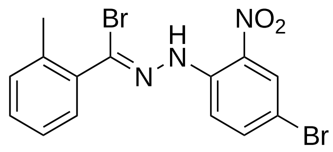

1f

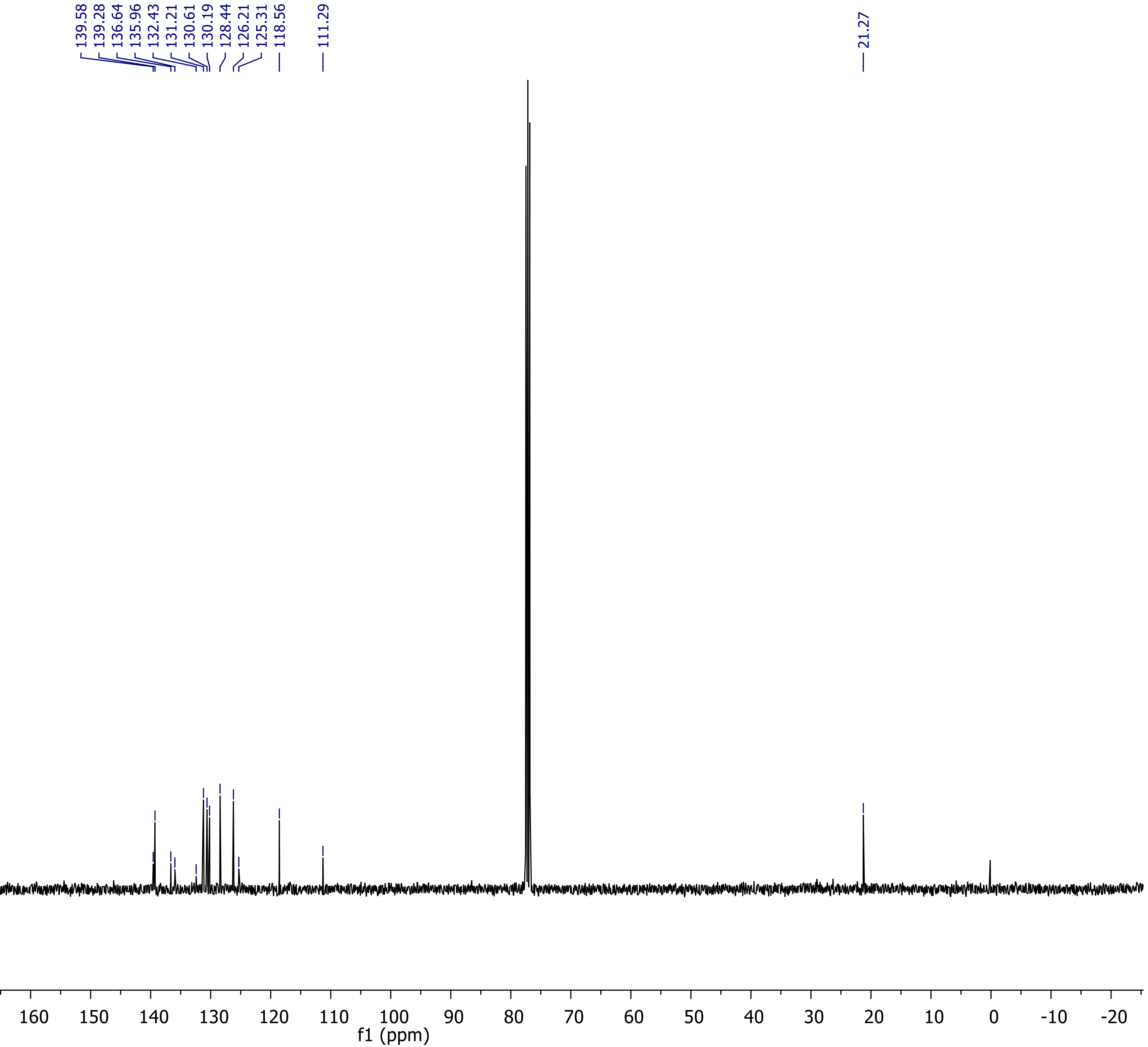

^{1}H NMR: 500 MHz, $CDCl_3$

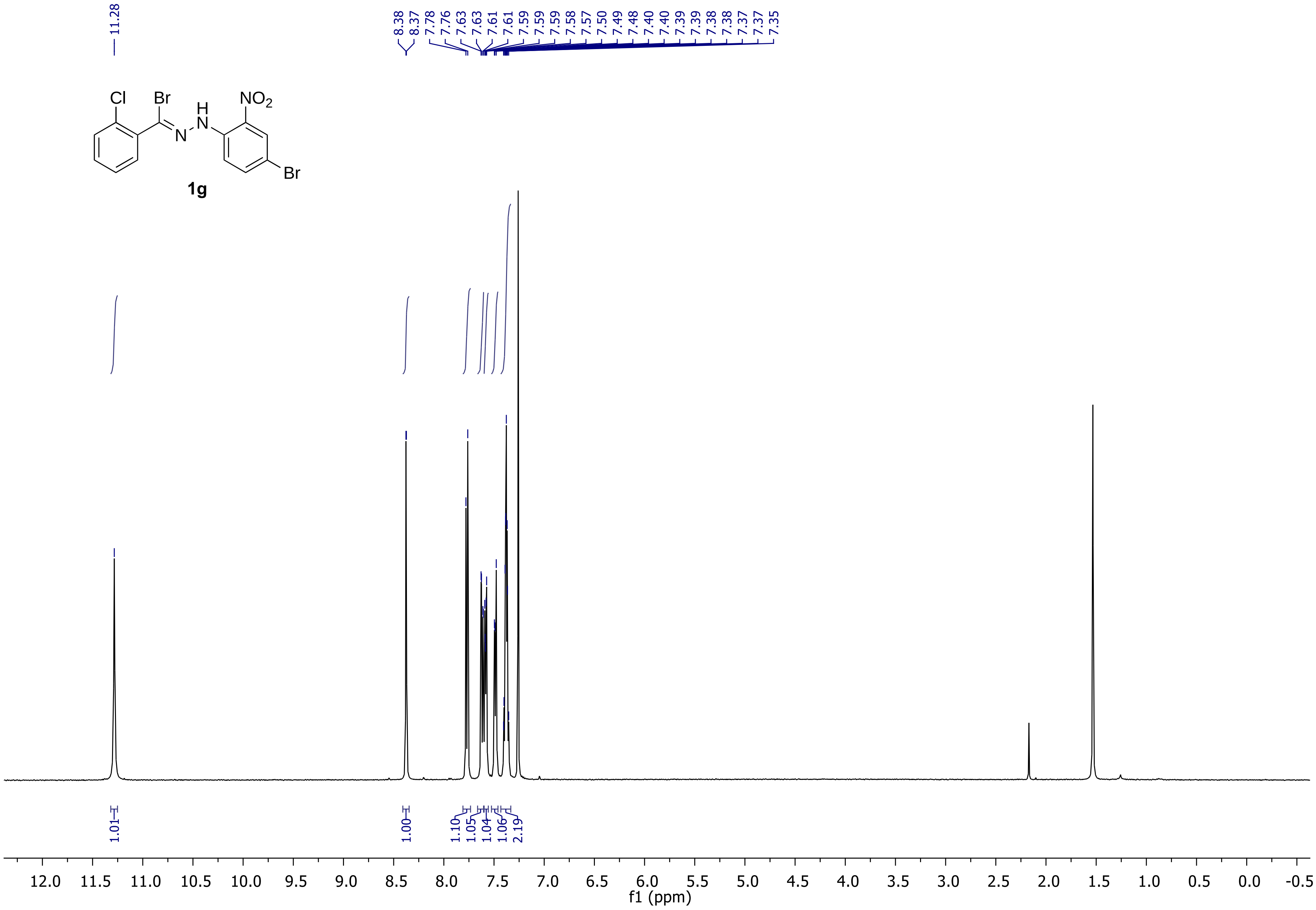

$^{13}C\{^{1}H\}$ NMR: 126 MHz, $CDCl_3$

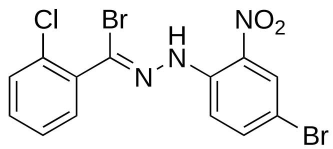

1g

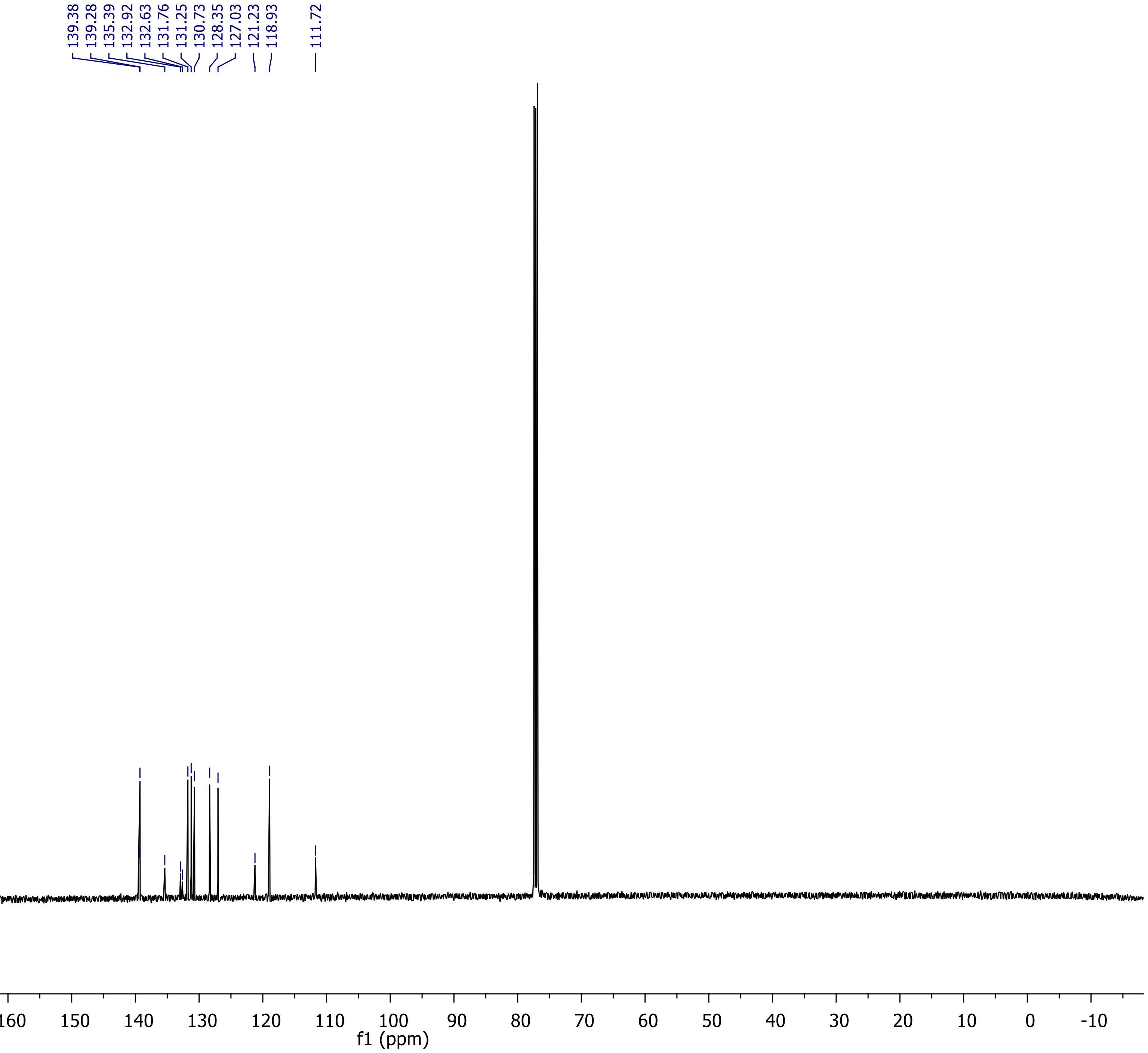

^{1}H NMR: 500 MHz, $CDCl_3$

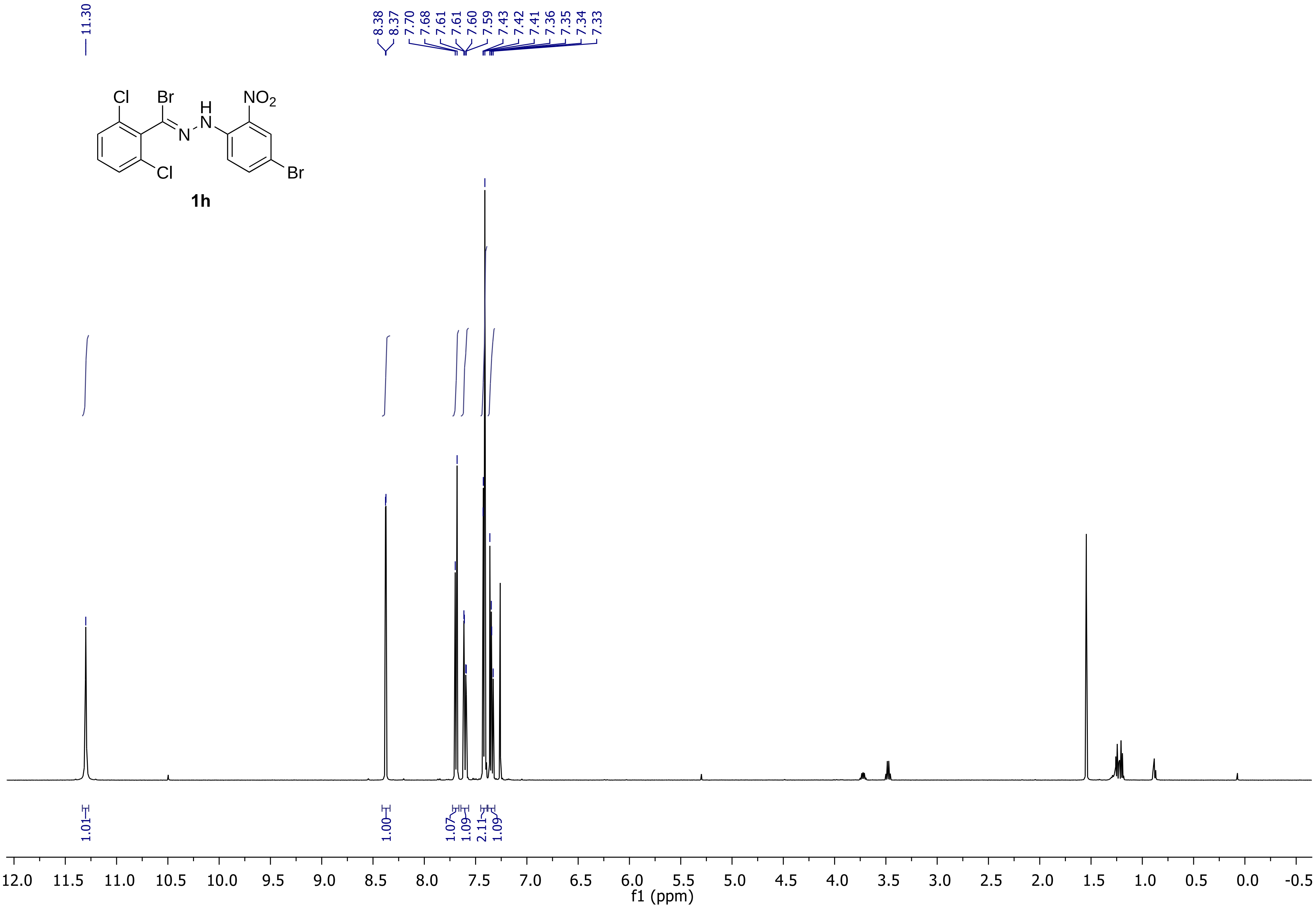

$^{13}C\{^{1}H\}$ NMR: 126 MHz, $CDCl_3$

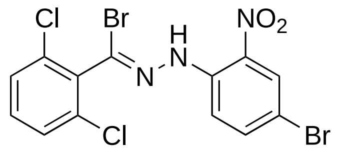

1h

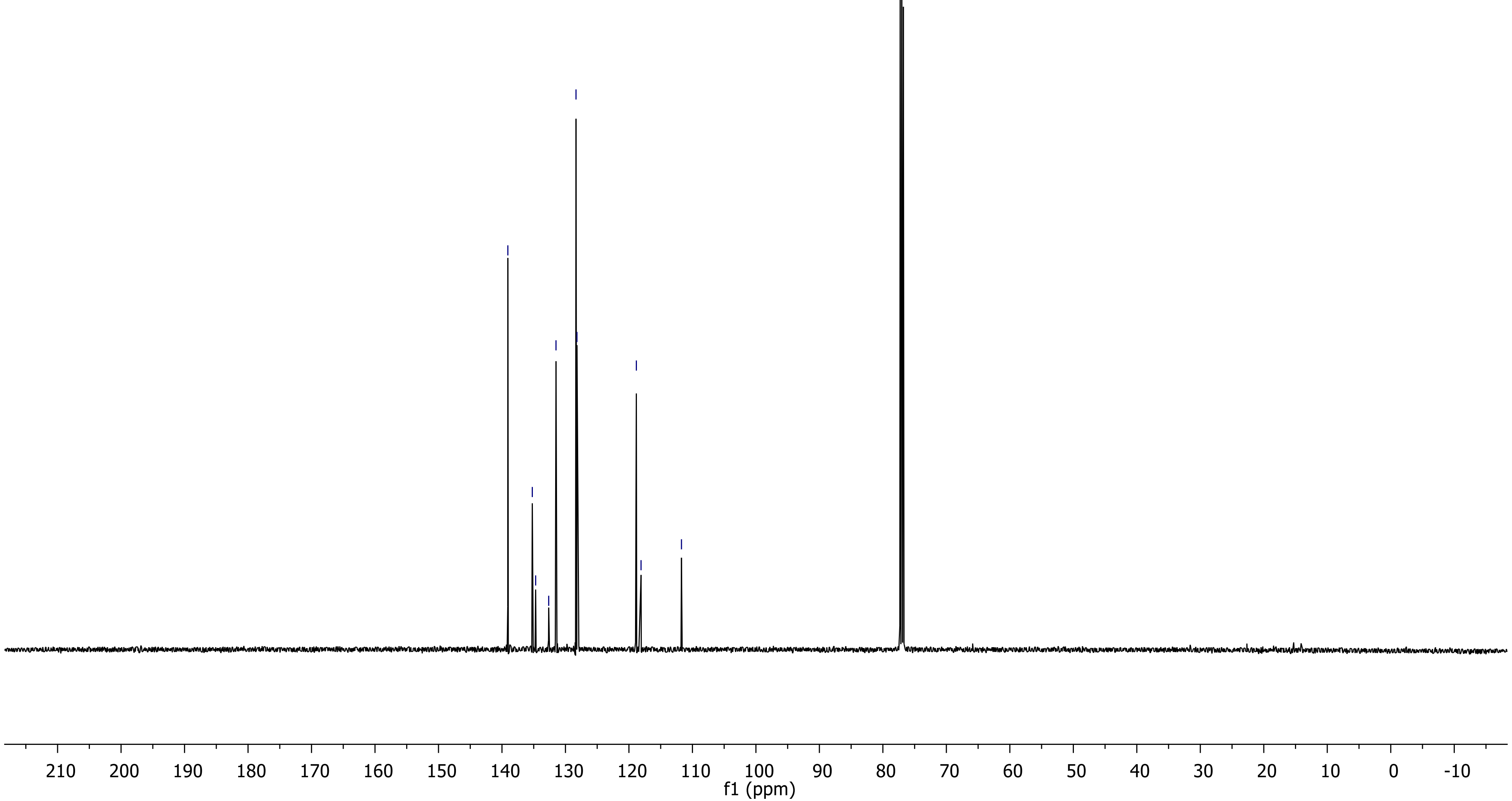

^{1}H NMR: 400 MHz, D_6-DMSO

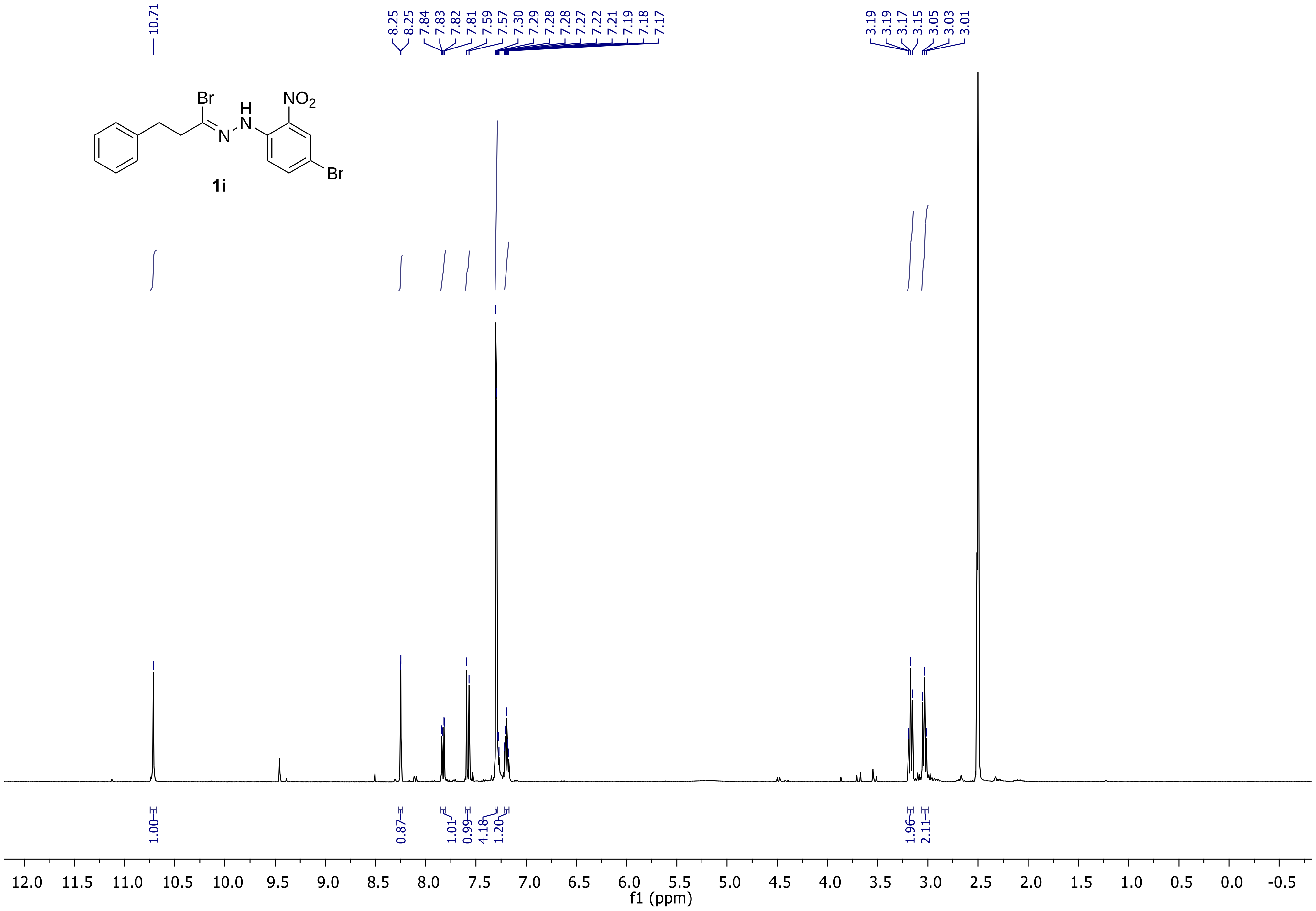

$^{13}C\{^{1}H\}$ NMR: 101 MHz, D_6-DMSO

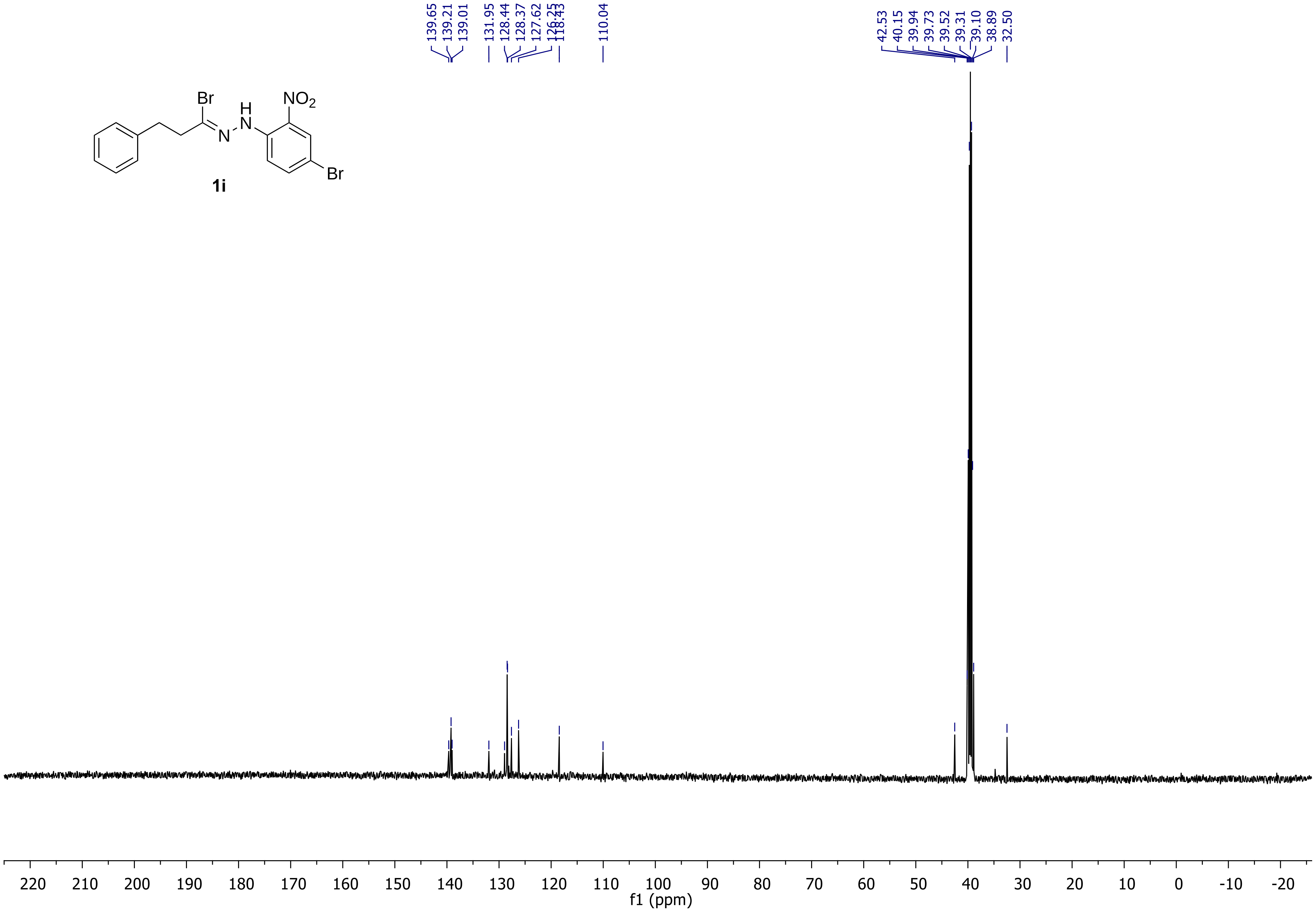

^{1}H NMR: 500 MHz, $CDCl_3$

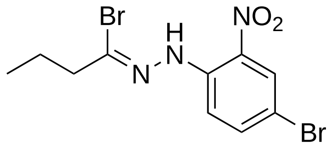

1j

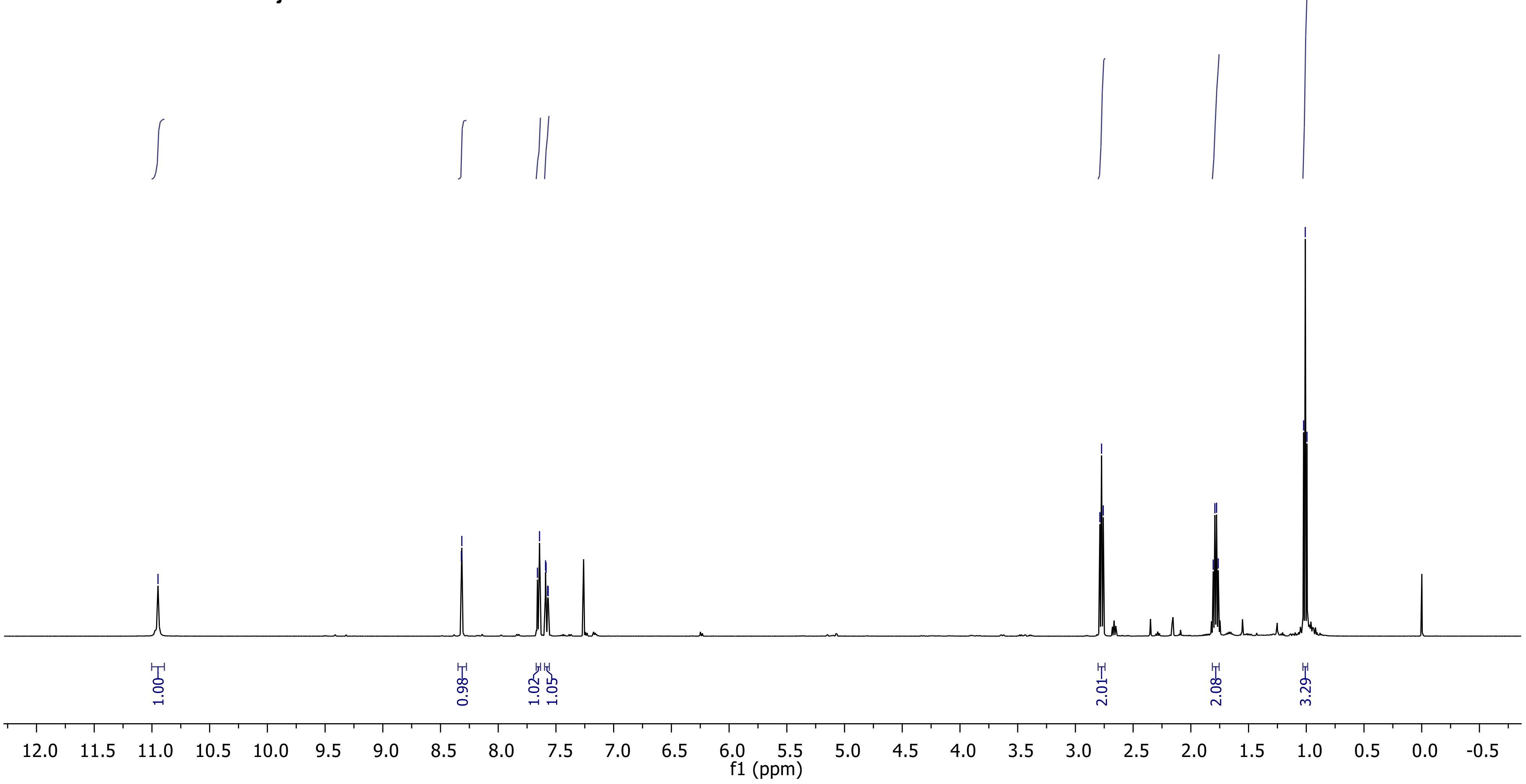

$^{13}C\{^1H\}$ NMR: 101 MHz, $CDCl_3$

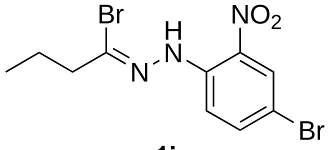

1j

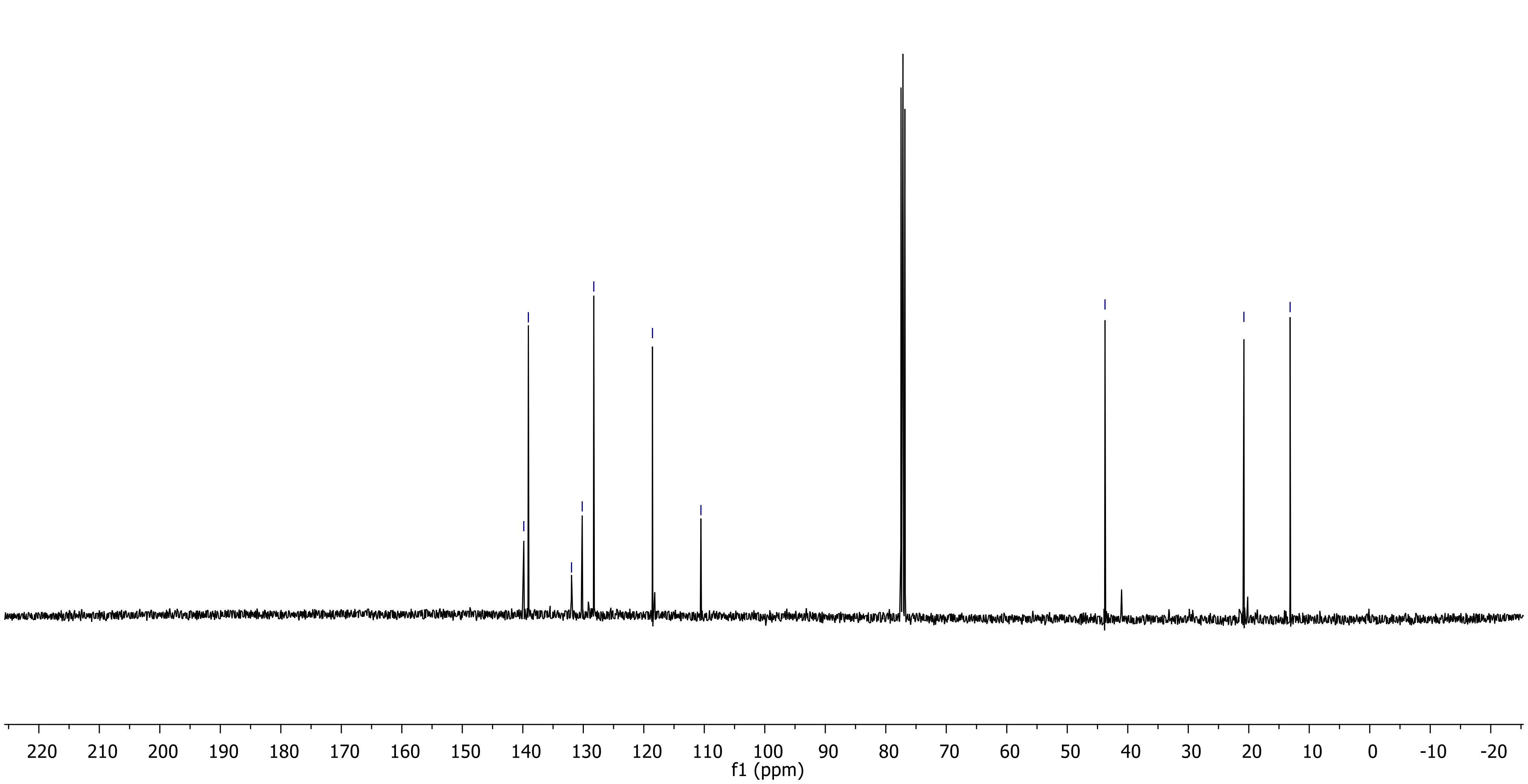

^{1}H NMR: 500 MHz, $CDCl_3$

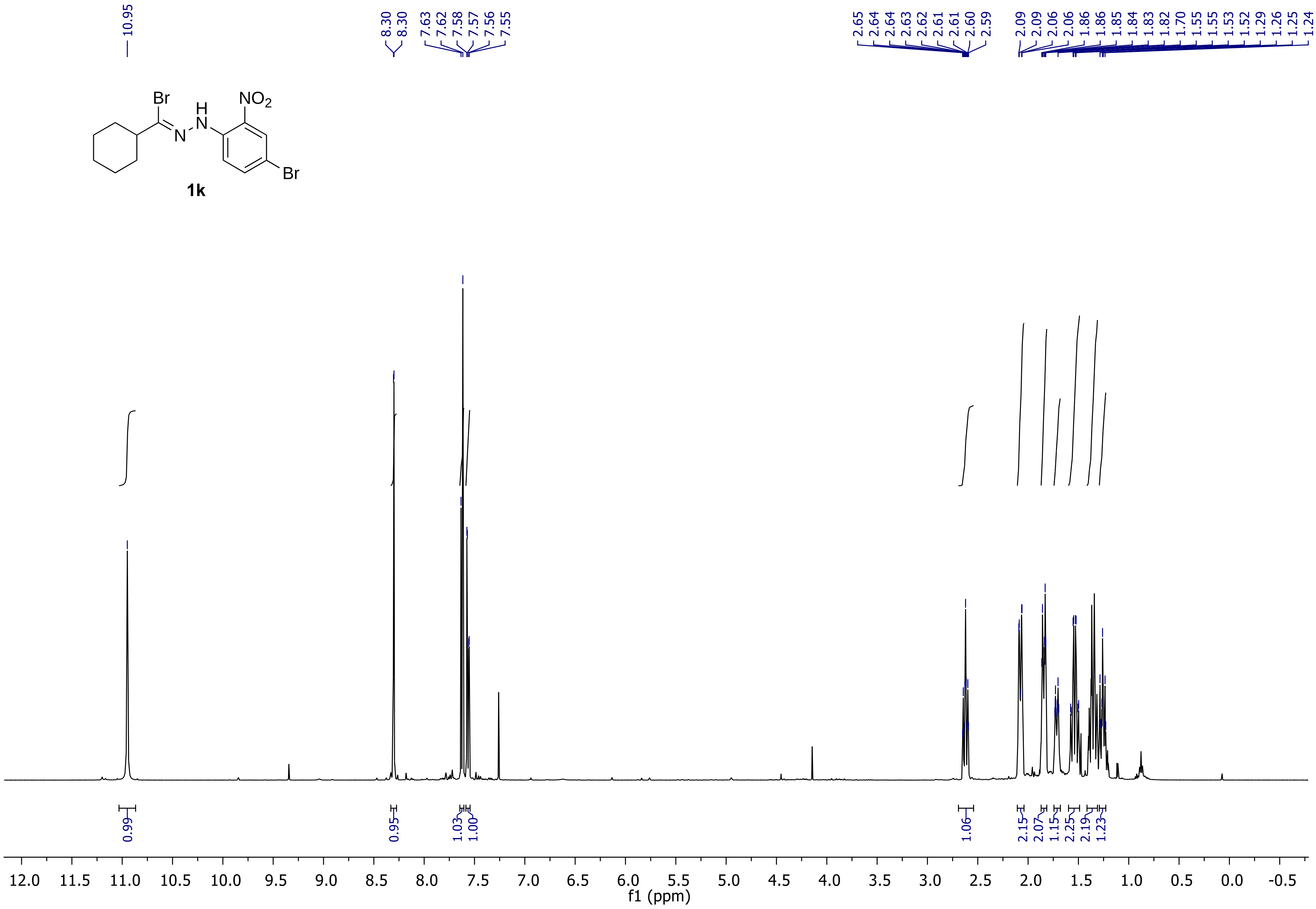

$^{13}C\{^1H\}$ NMR: 126 MHz, $CDCl_3$

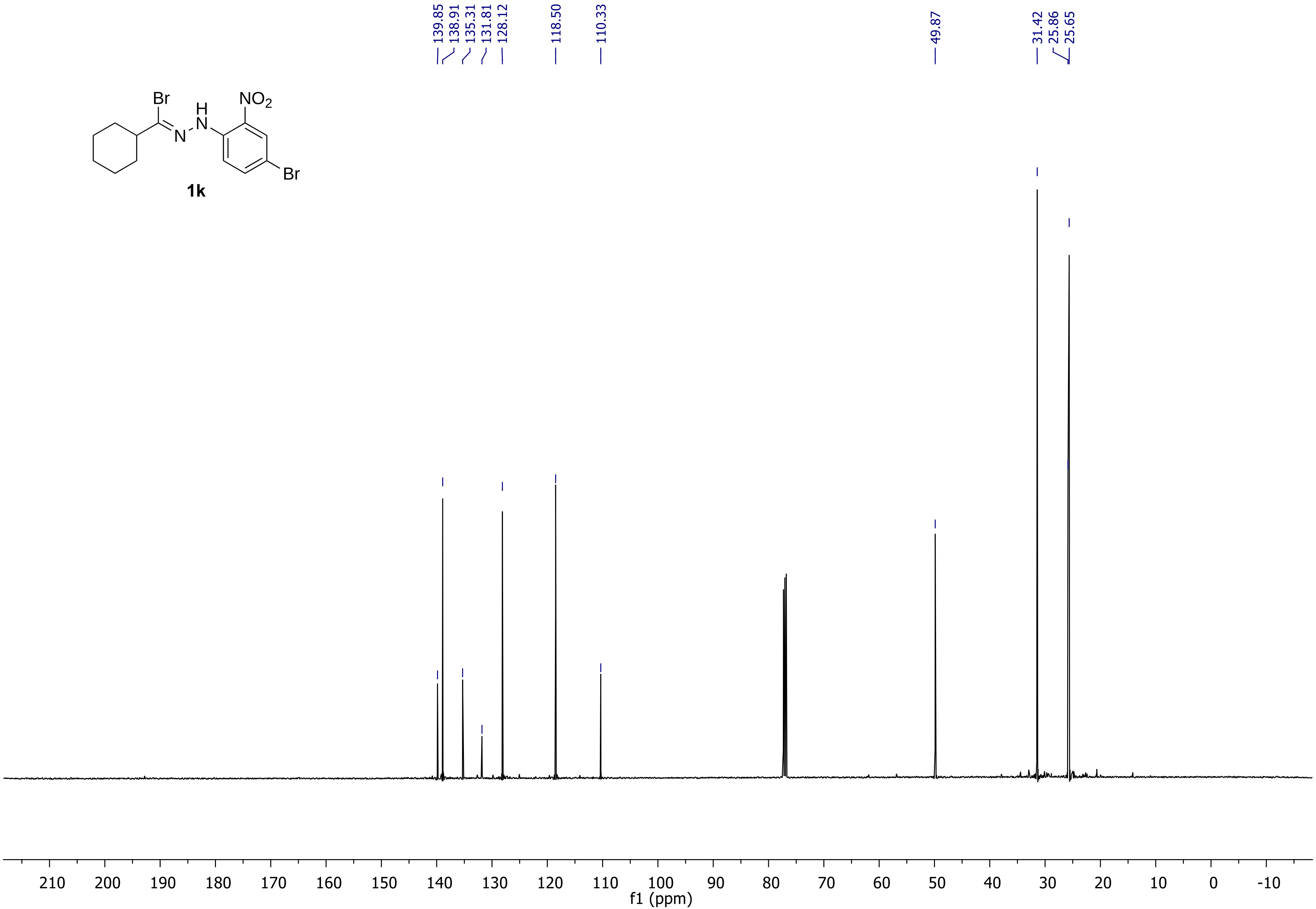

^{1}H NMR: 500 MHz, $CDCl_3$

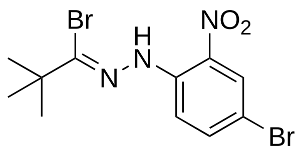

1l

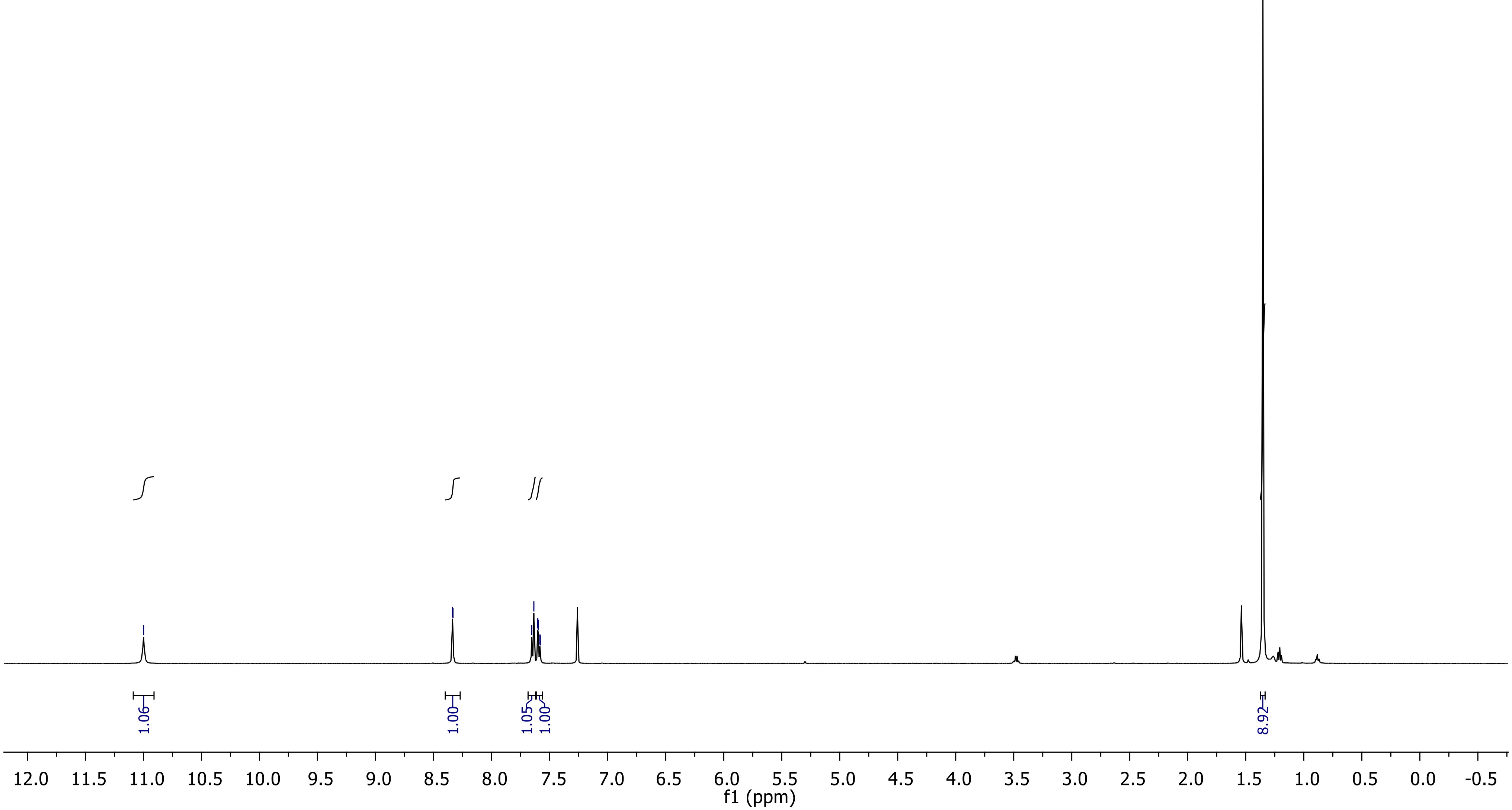

$^{13}C\{^{1}H\}$ NMR: 126 MHz, $CDCl_3$

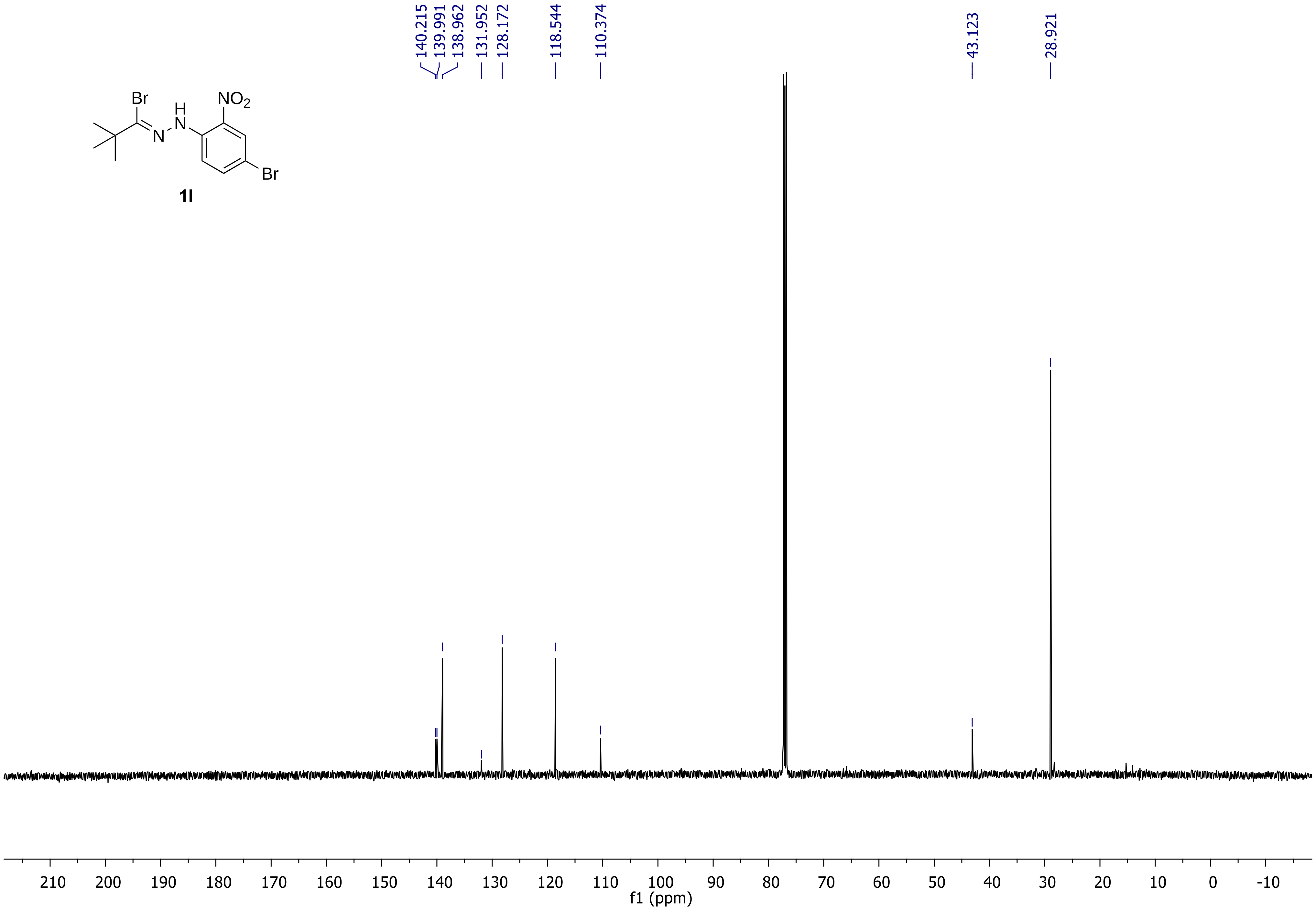

^1H NMR: 500 MHz, $CDCl_3$

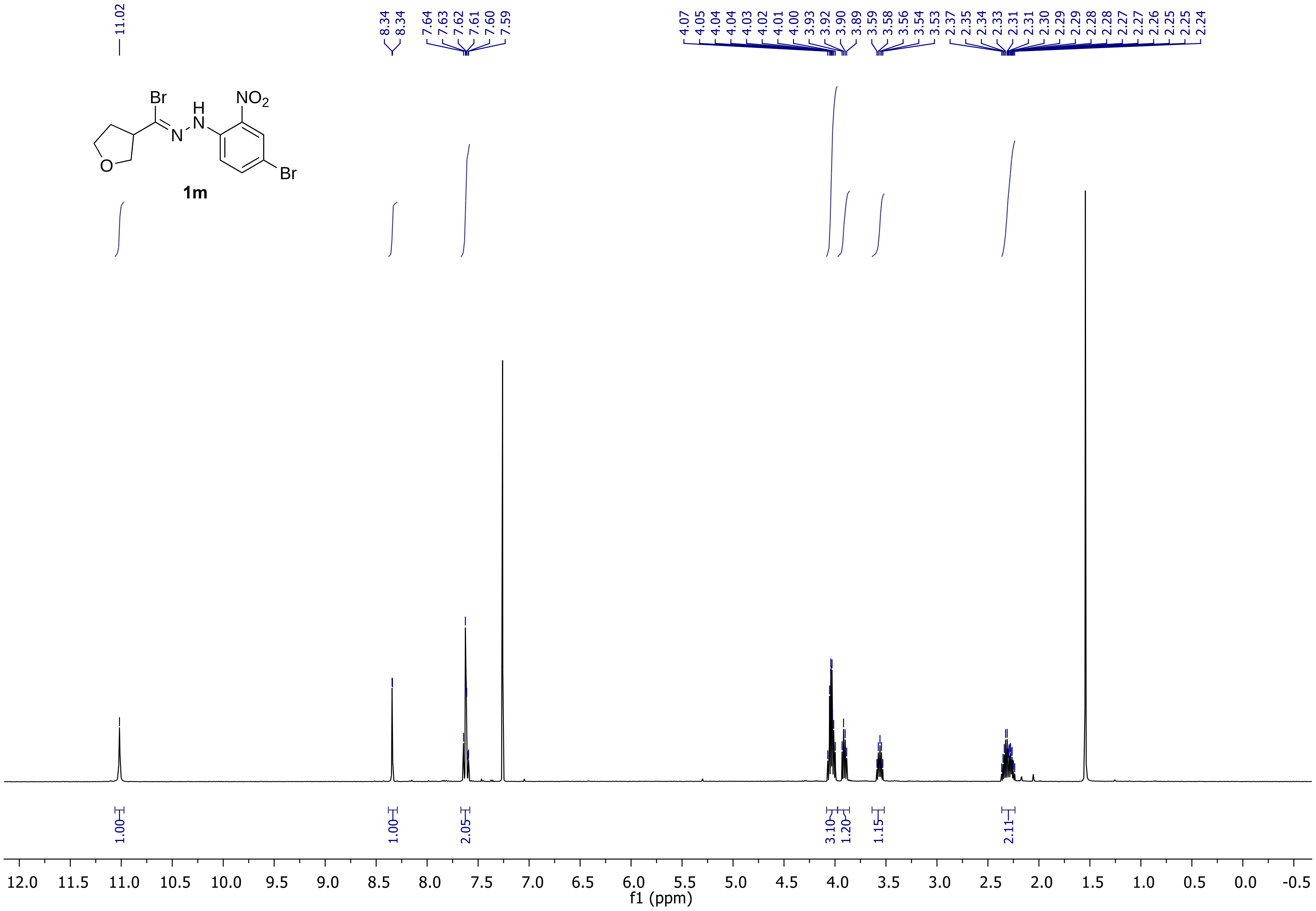

^{1}H NMR: 500 MHz, $CDCl_3$

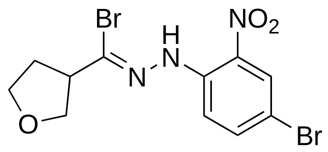

1m

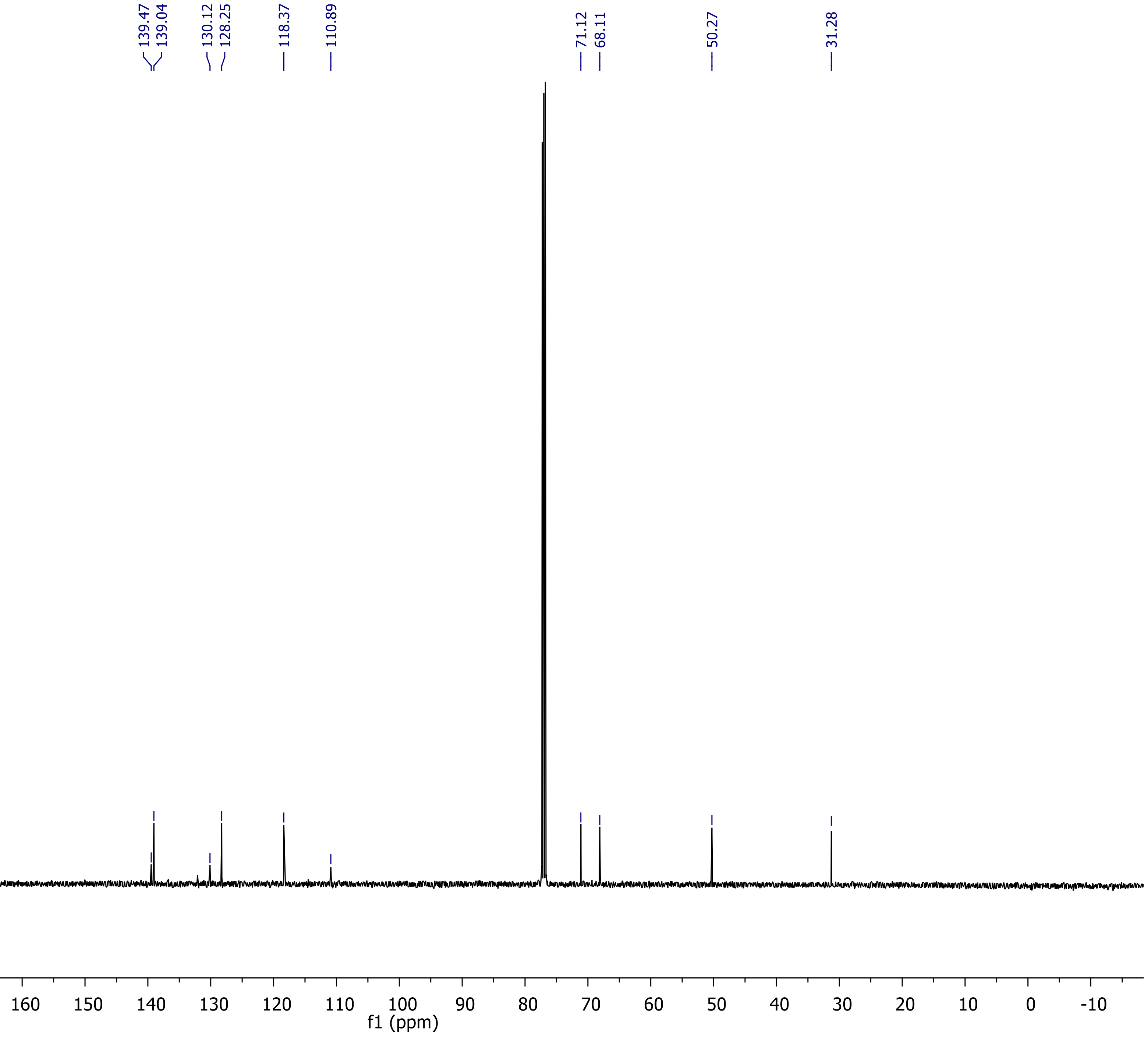

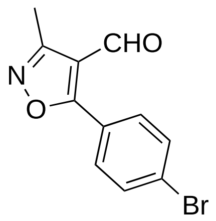
CHO
N
O
Br

S1

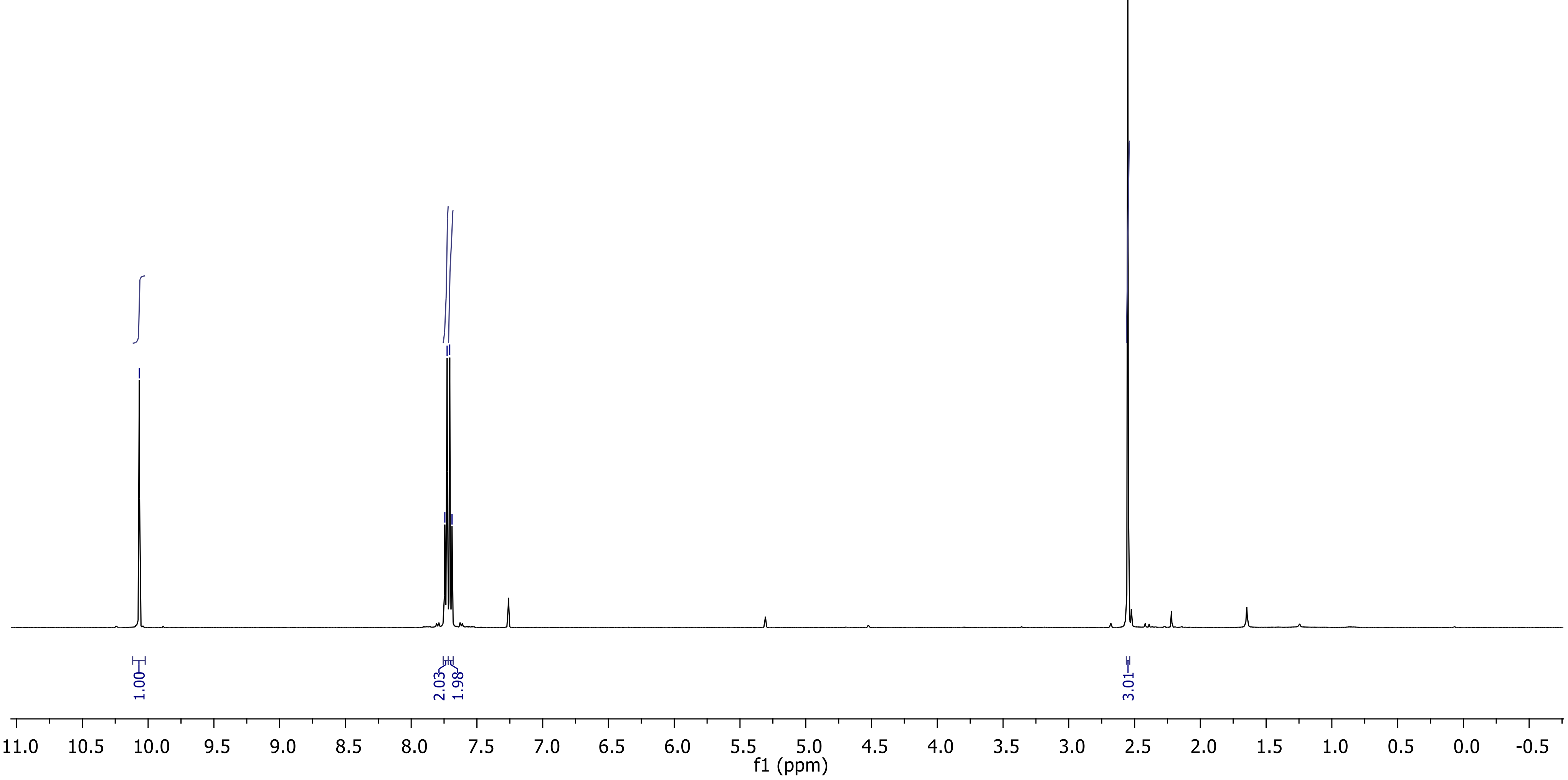
10.07
7.74
7.73
7.71
7.69
2.55
1.00
2.03
1.98
3.01
11.0 10.5 10.0 9.5 9.0 8.5 8.0 7.5 7.0 6.5 6.0 5.5 5.0 4.5 4.0 3.5 3.0 2.5 2.0 1.5 1.0 0.5 0.0 -0.5
f1 (ppm)

$^{13}C\{^{1}H\}$ NMR: 126 MHz, $CDCl_3$

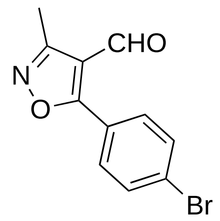

S1

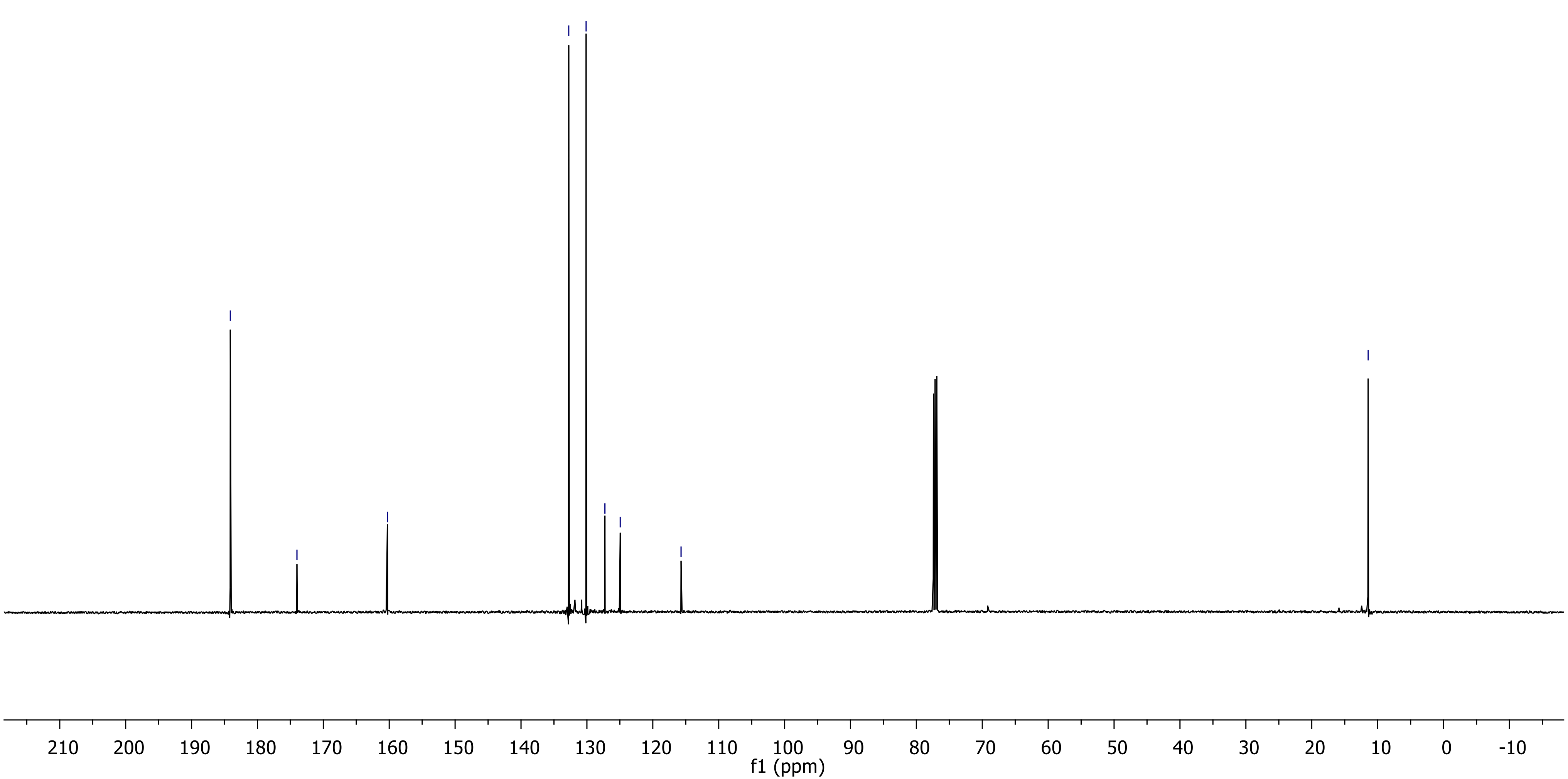

^{1}H NMR: 500 MHz, $CDCl_3$

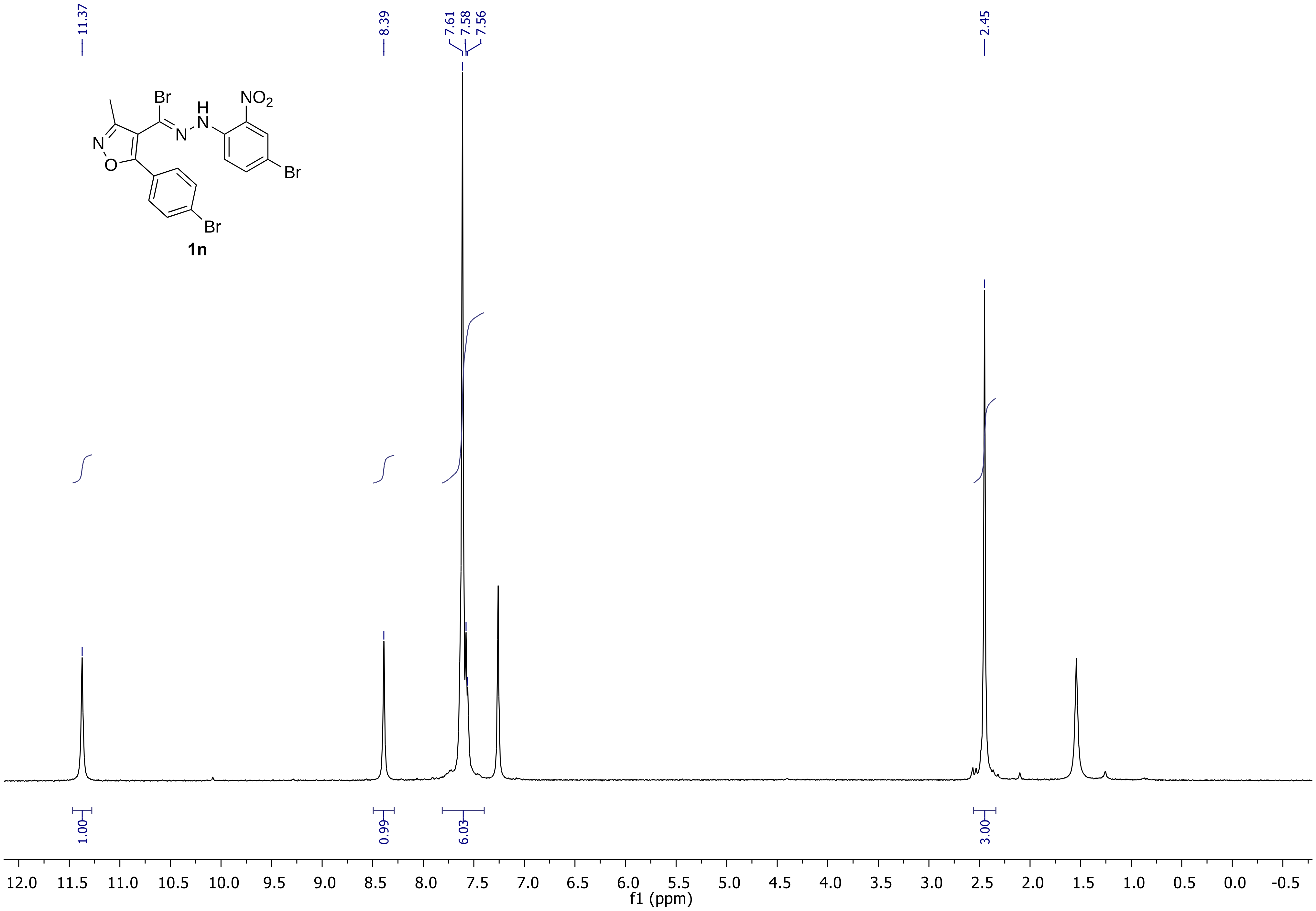

$^{13}C\{^1H\}$ NMR: 126 MHz, $CDCl_3$

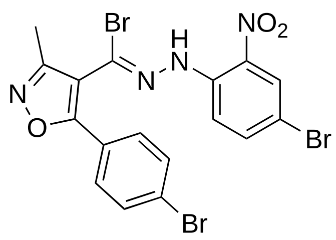

1n

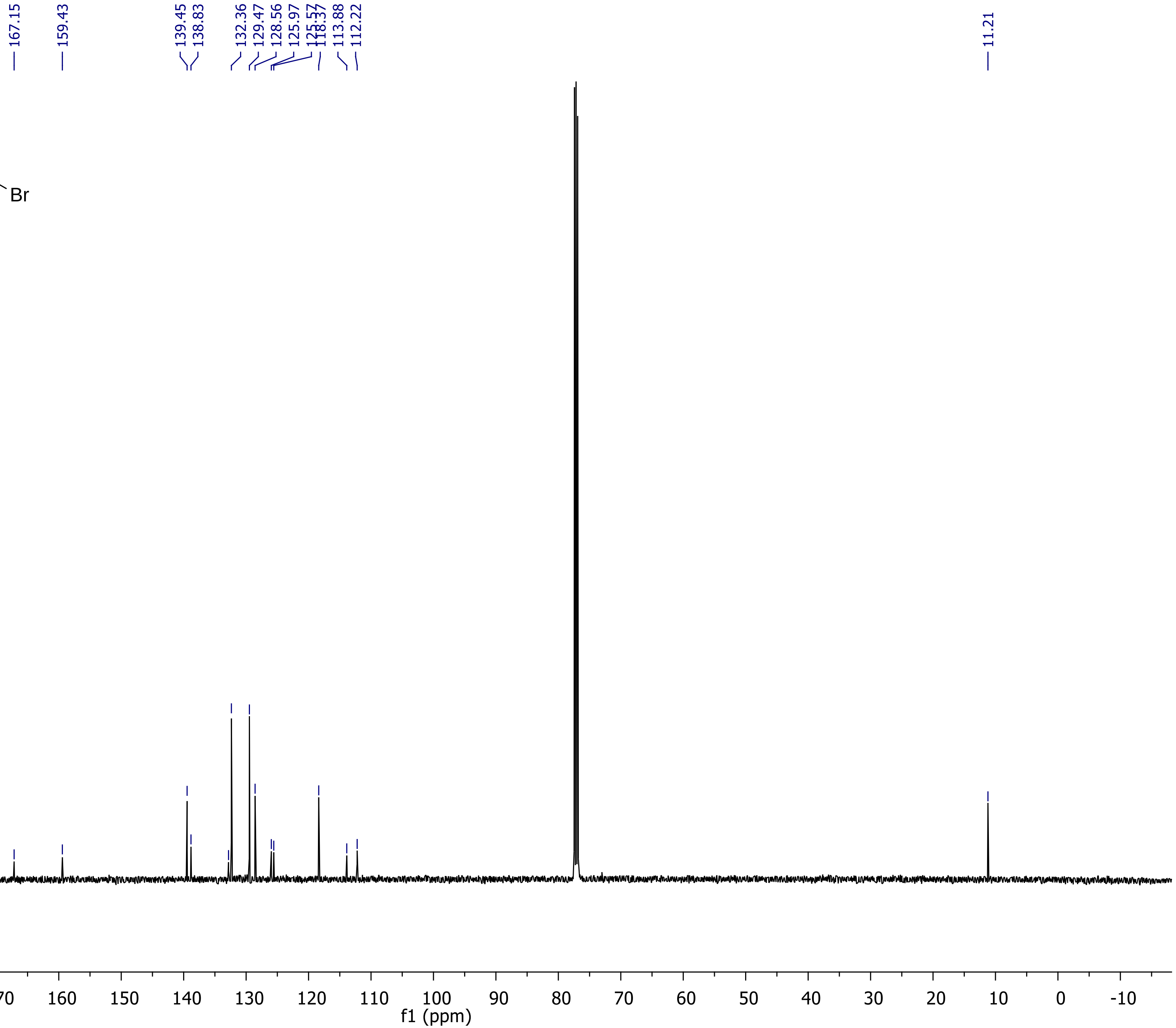

^{1}H NMR: 500 MHz, $CDCl_3$

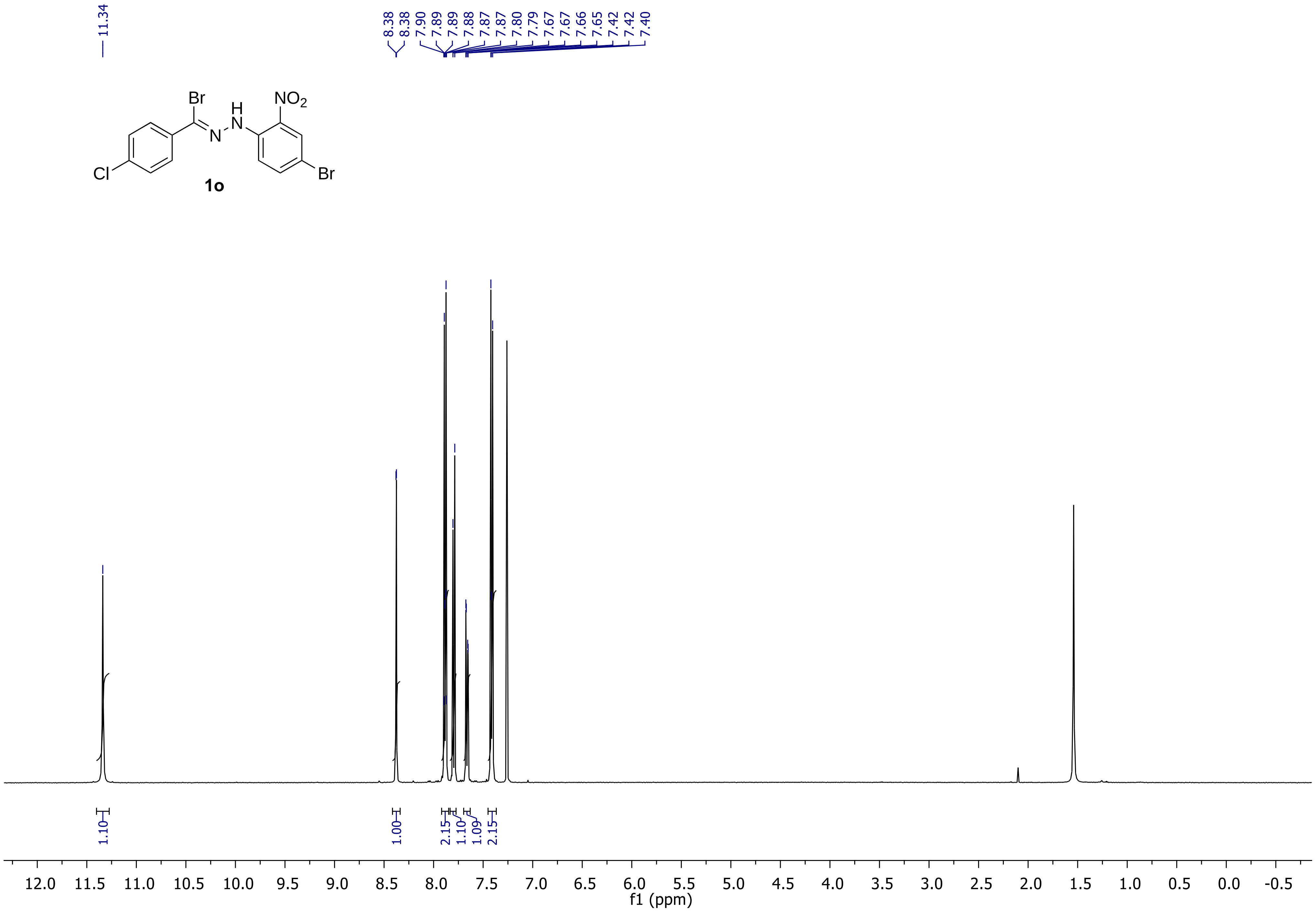

$^{13}C\{^1H\}$ NMR: 126 MHz, $CDCl_3$

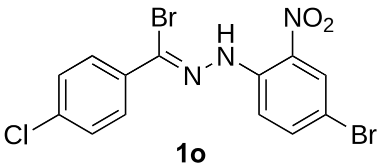

1o

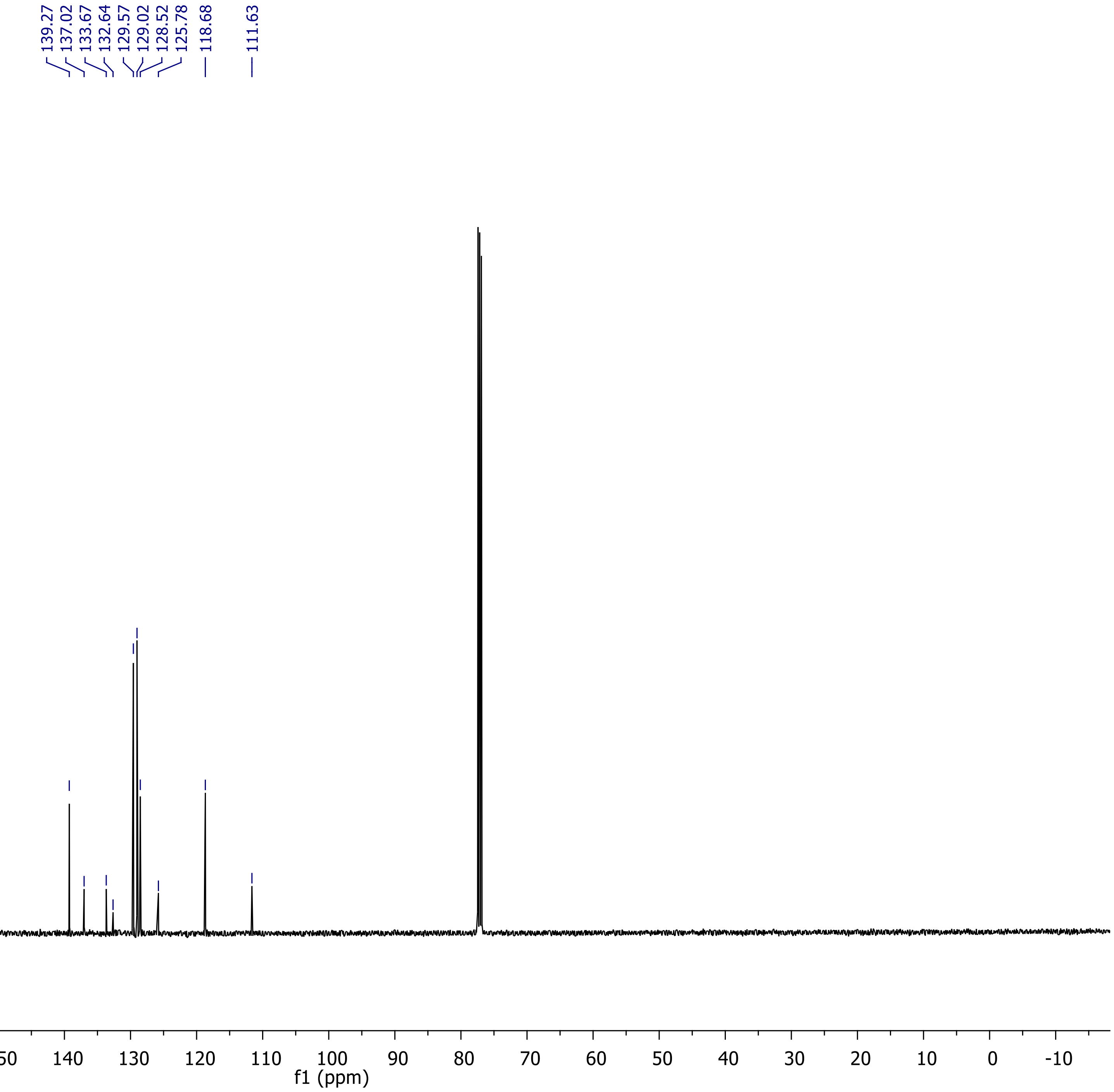

^{1}H NMR: 500 MHz, $CDCl_3$

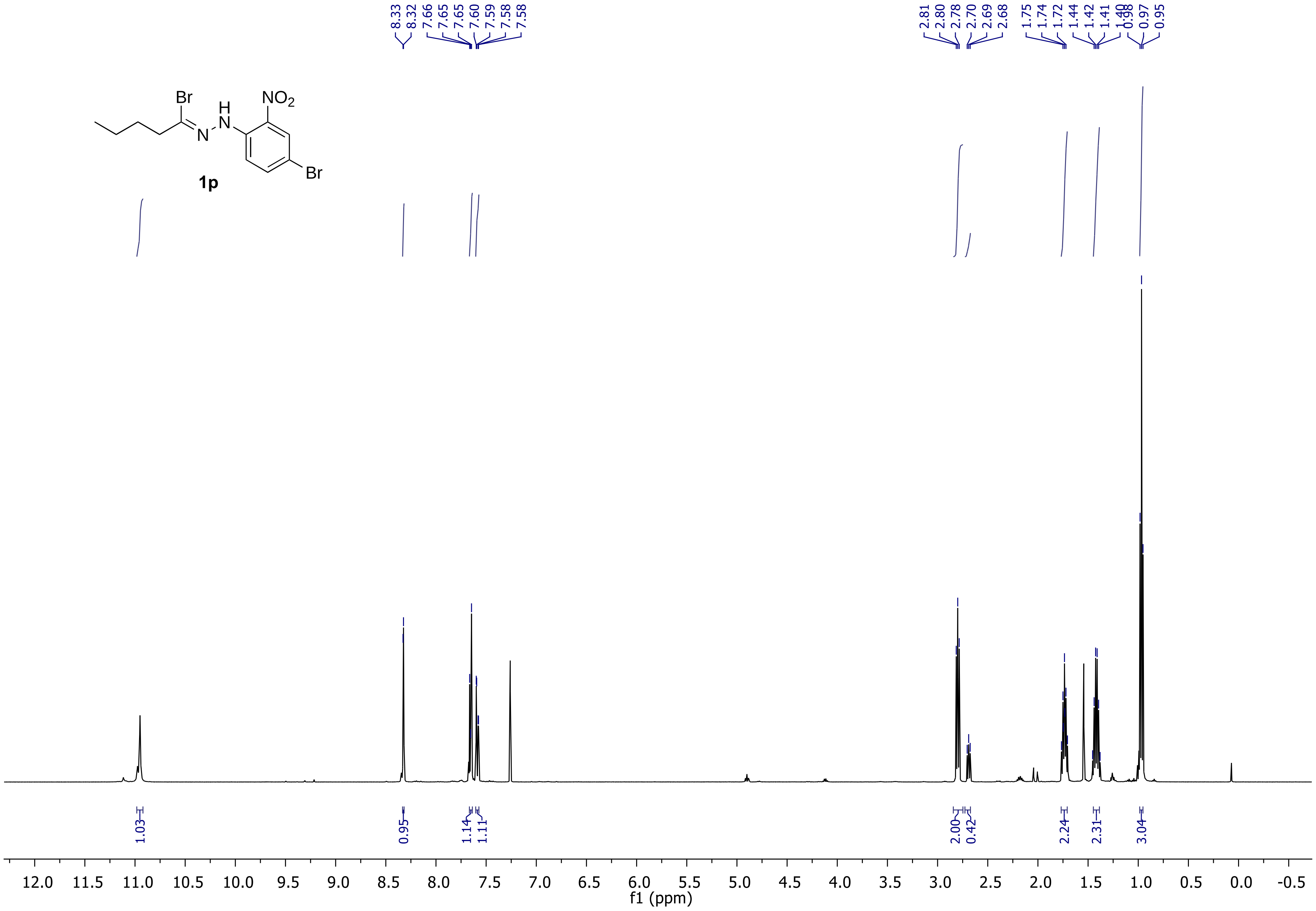

$^{13}C\{^1H\}$ NMR: 101 MHz, $CDCl_3$

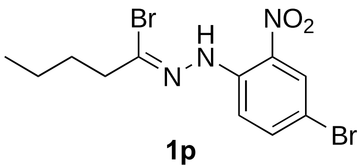

1p

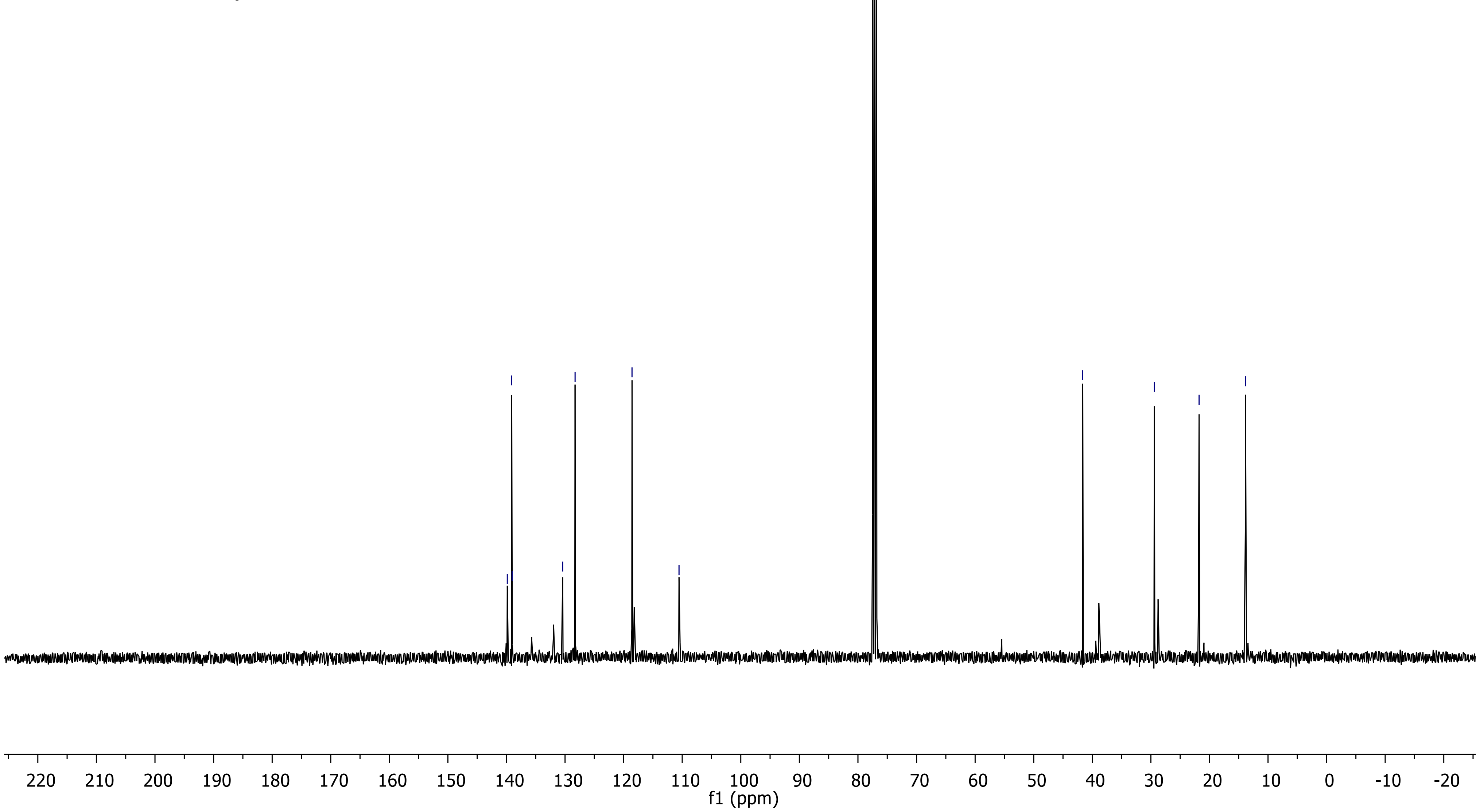

^{1}H NMR: 500 MHz, $CDCl_3$

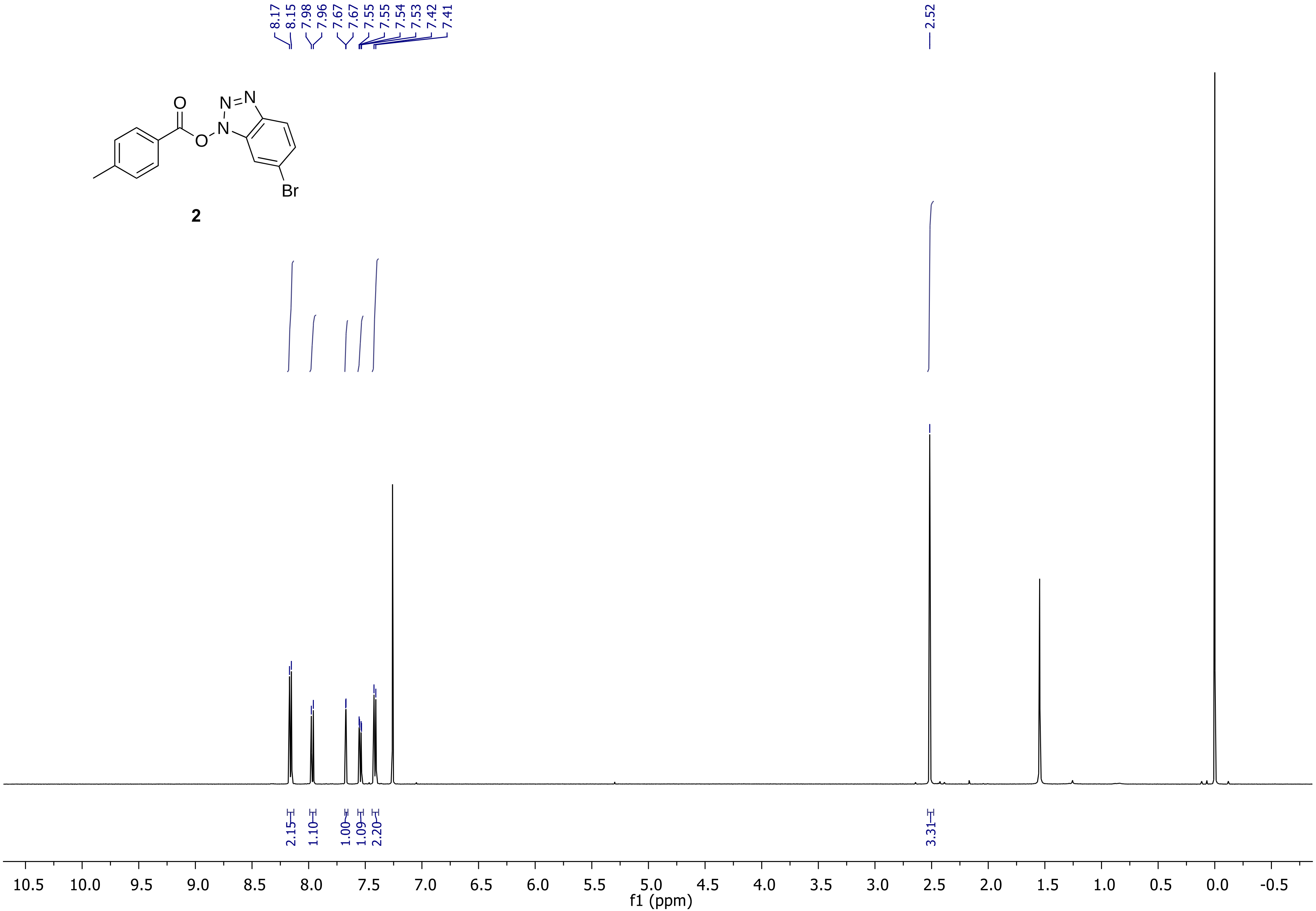

$^{13}C\{^{1}H\}$ NMR: 101 MHz, $CDCl_3$

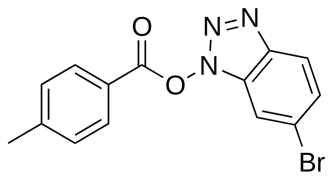

2

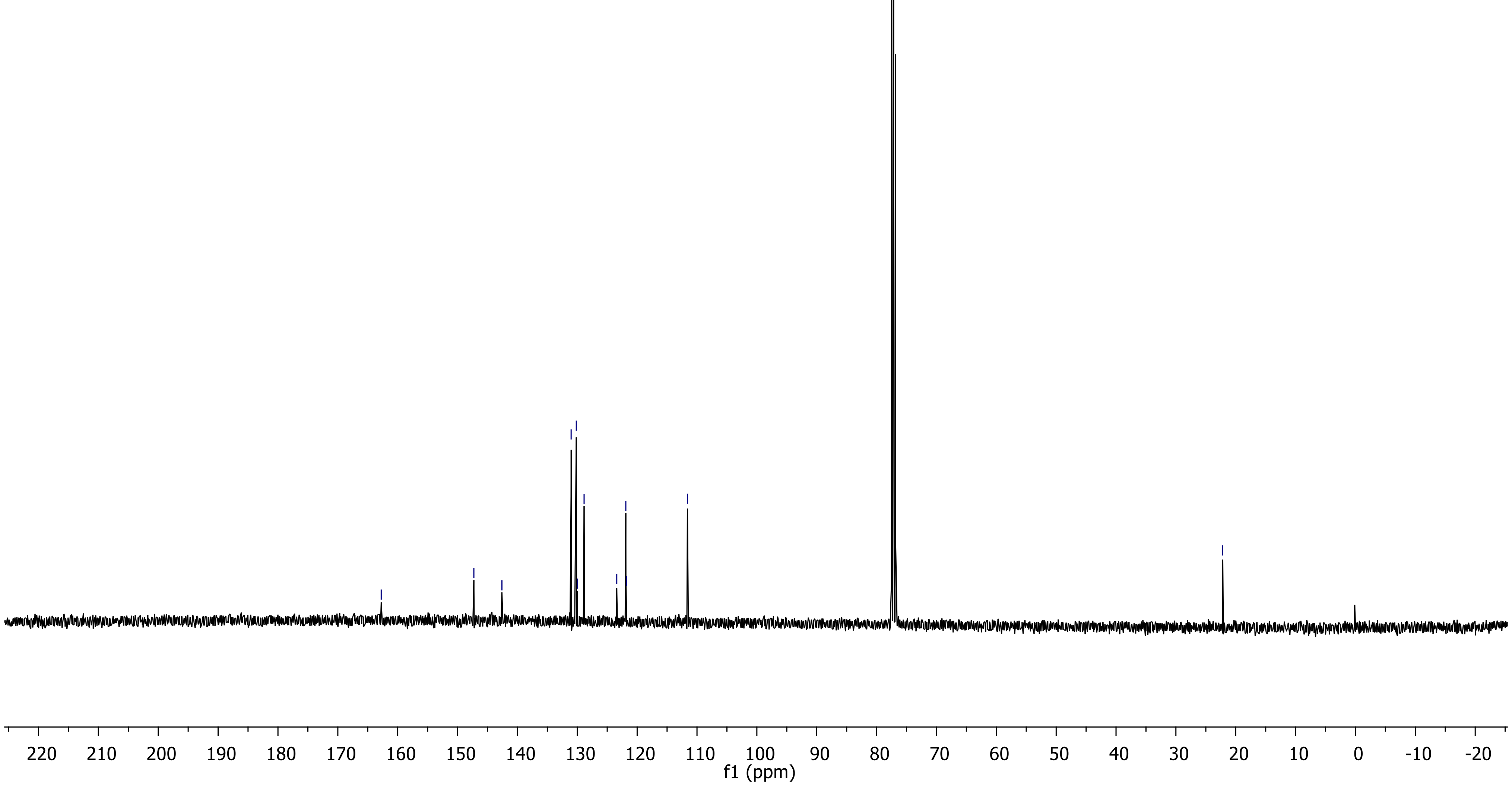

^{1}H NMR: 500 MHz, $CDCl_3$

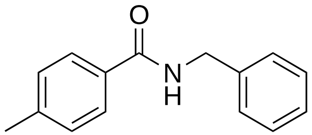

3a

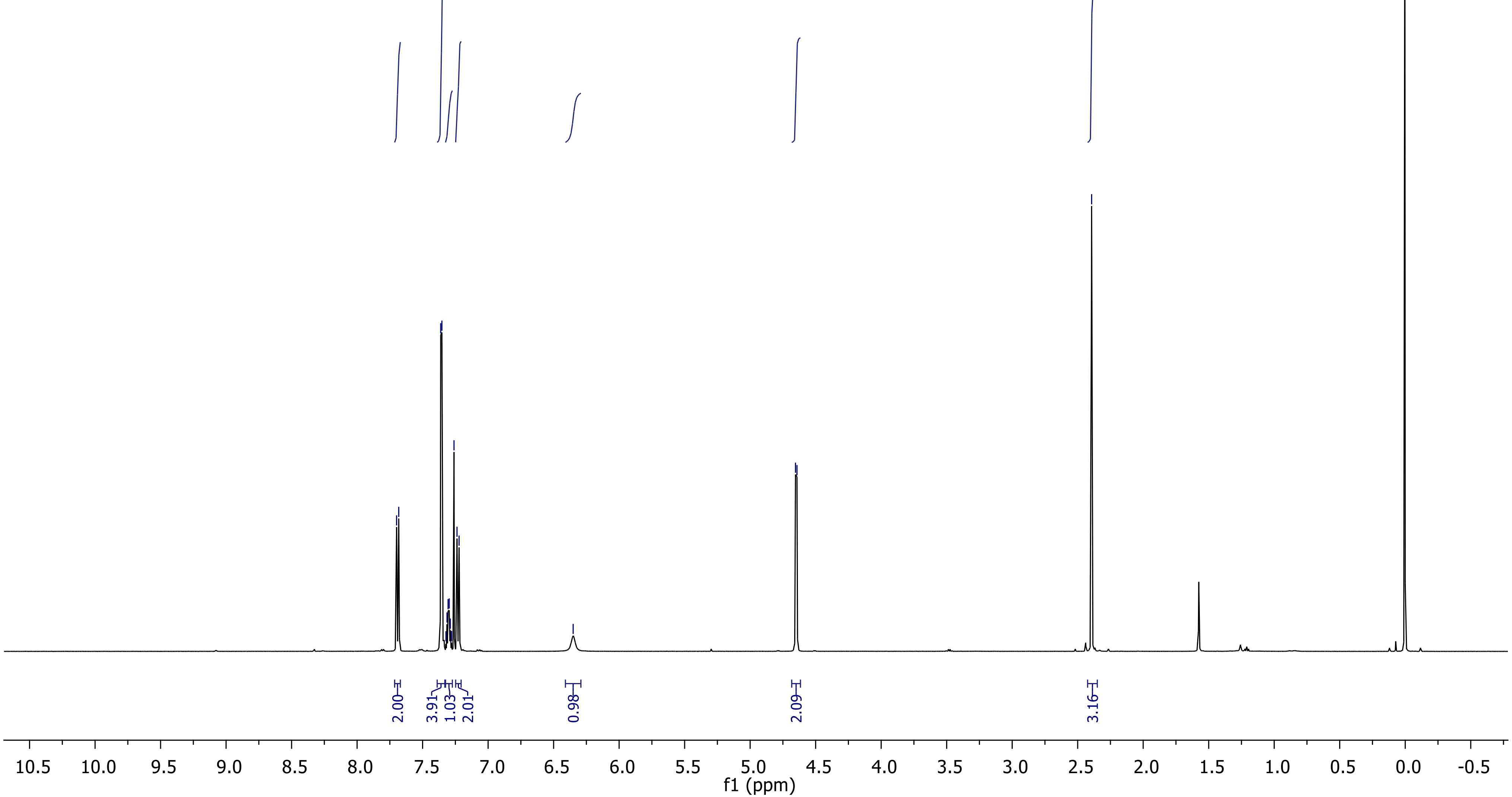

$^{13}C\{^{1}H\}$ NMR: 126 MHz, $CDCl_3$

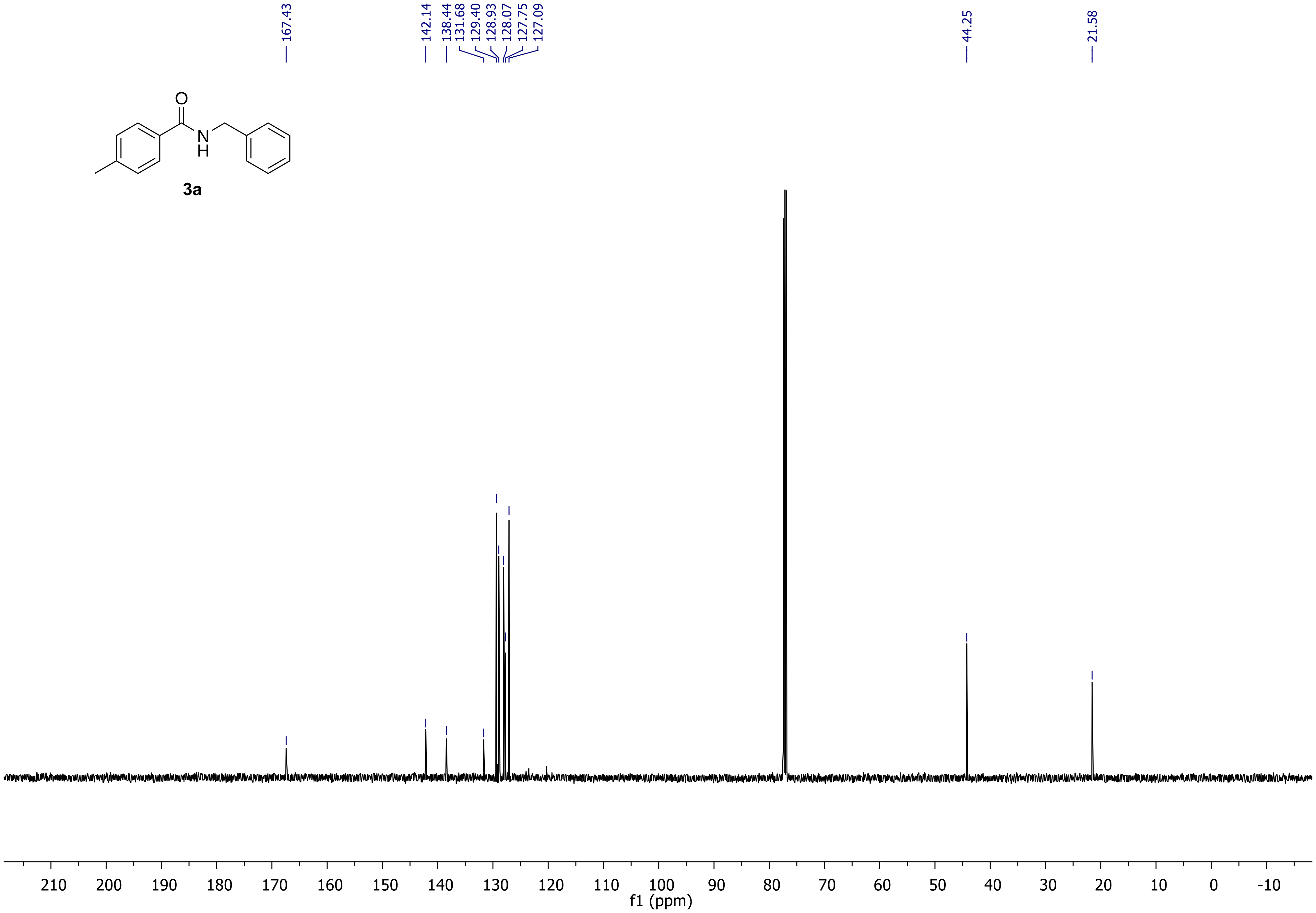

^{1}H NMR: 500 MHz, $CDCl_3$

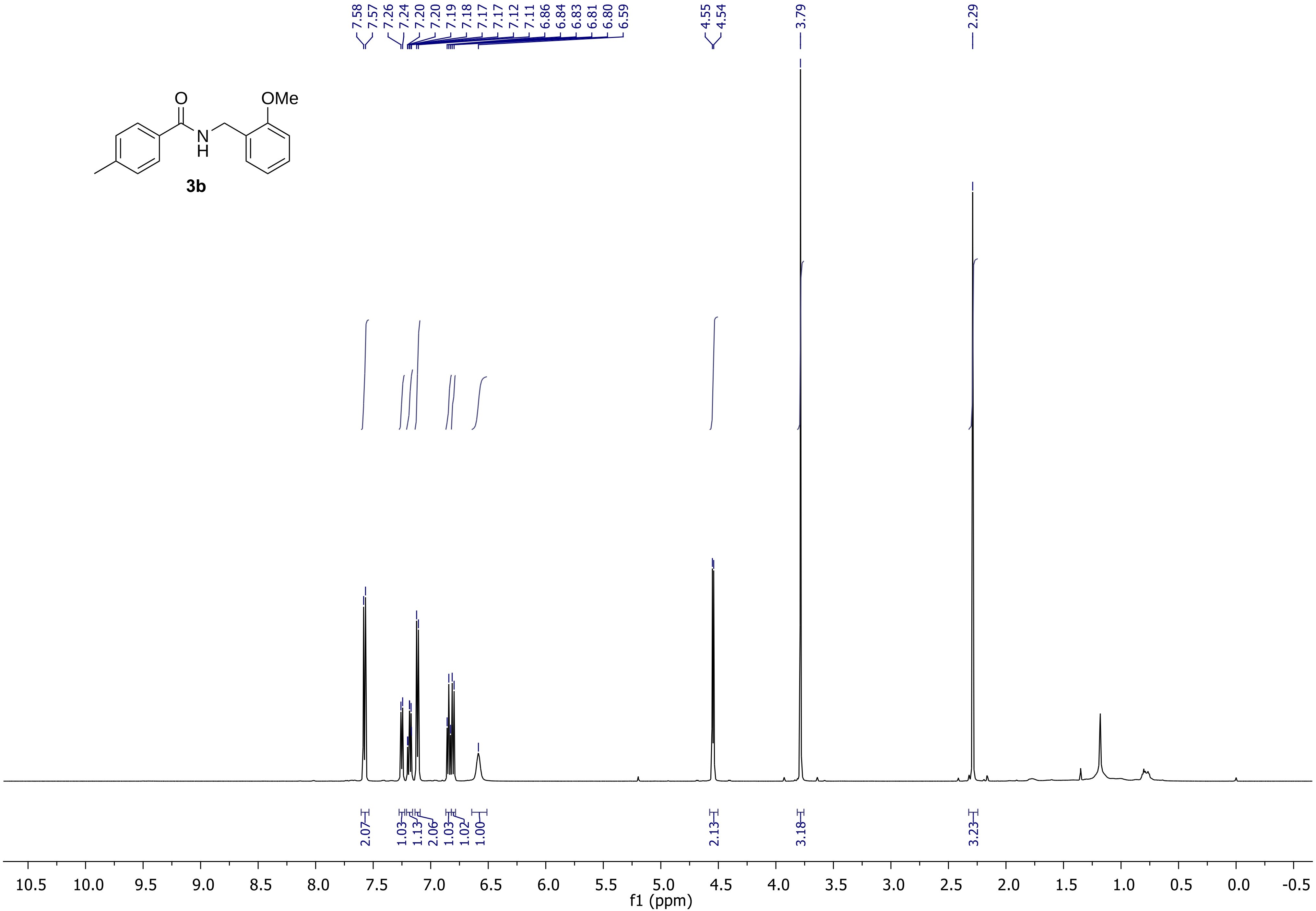

$^{13}C\{^{1}H\}$ NMR: 101 MHz, $CDCl_3$

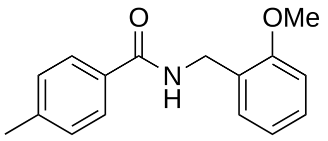

3b

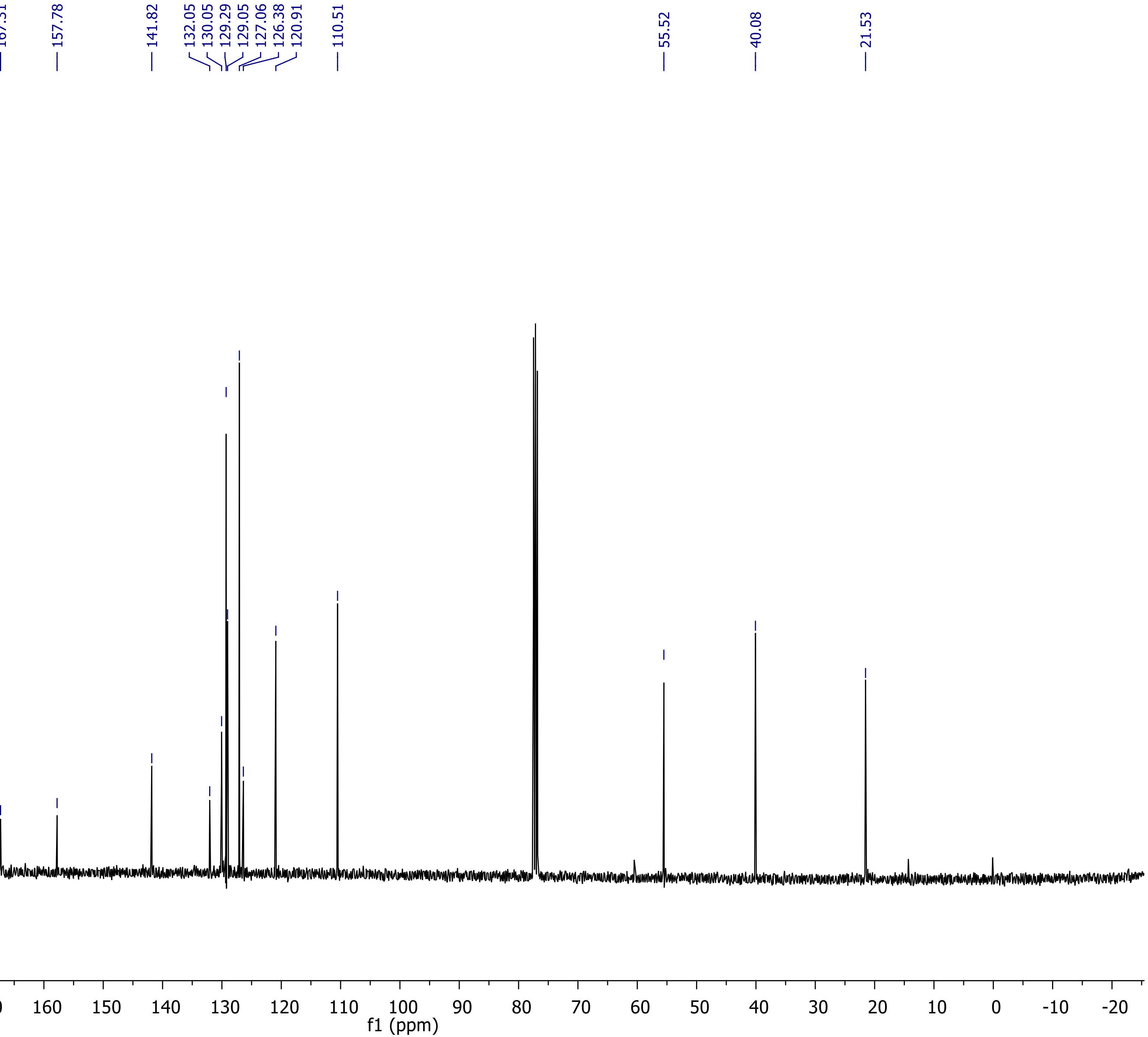

^{1}H NMR: 500 MHz, $CDCl_3$

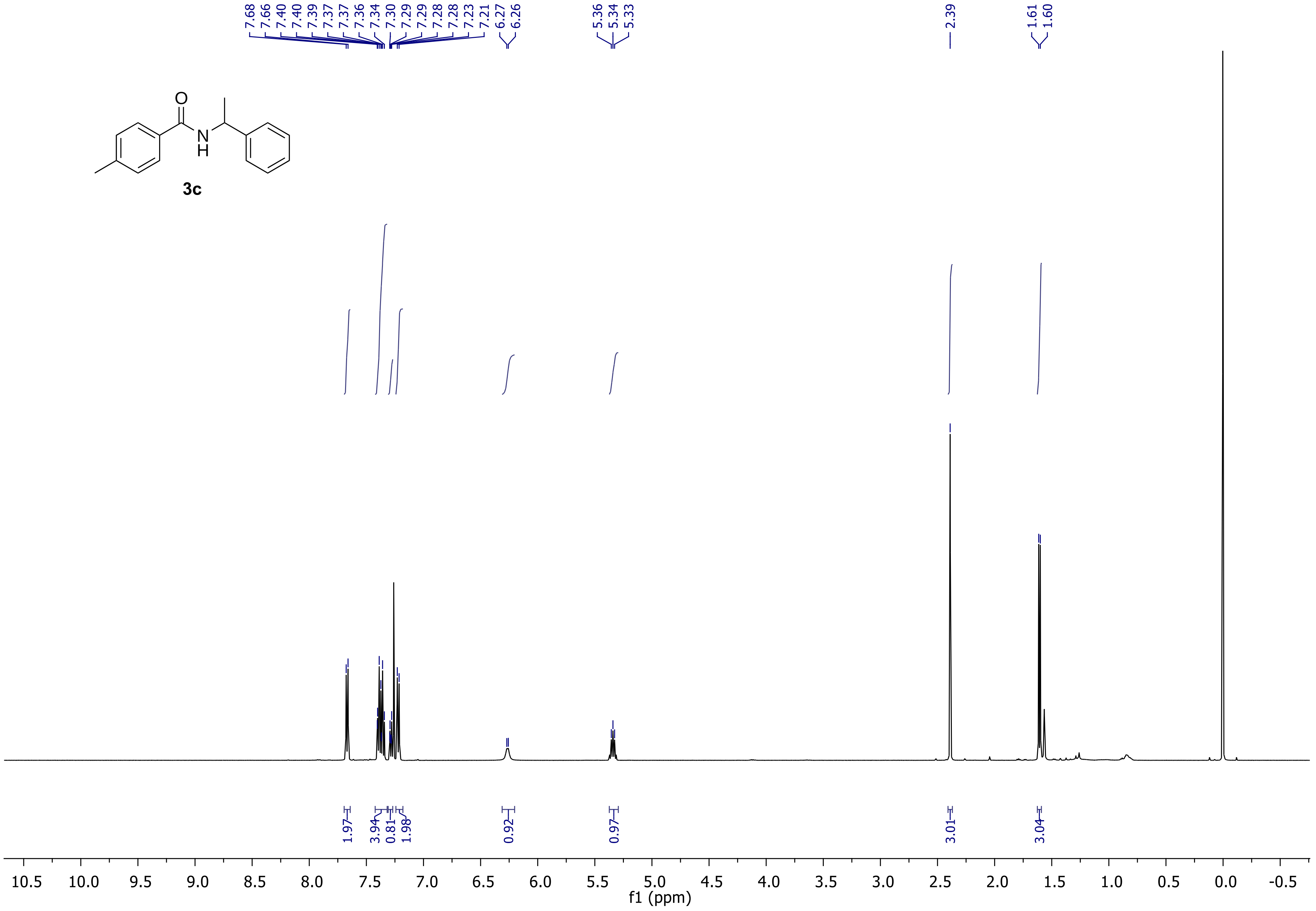

$^{13}C\{^{1}H\}$ NMR: 101 MHz, $CDCl_3$

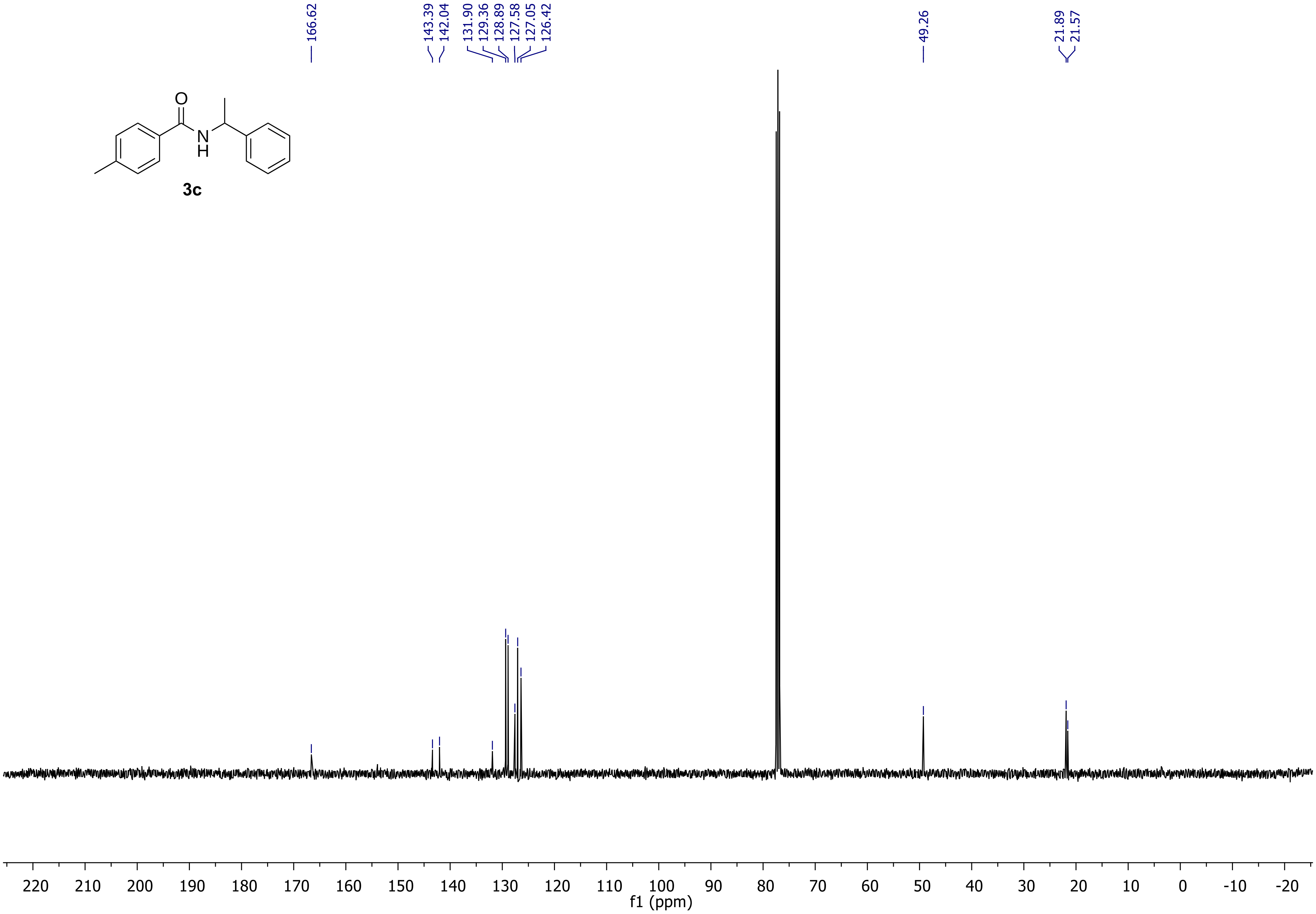

^{1}H NMR: 500 MHz, $CDCl_3$

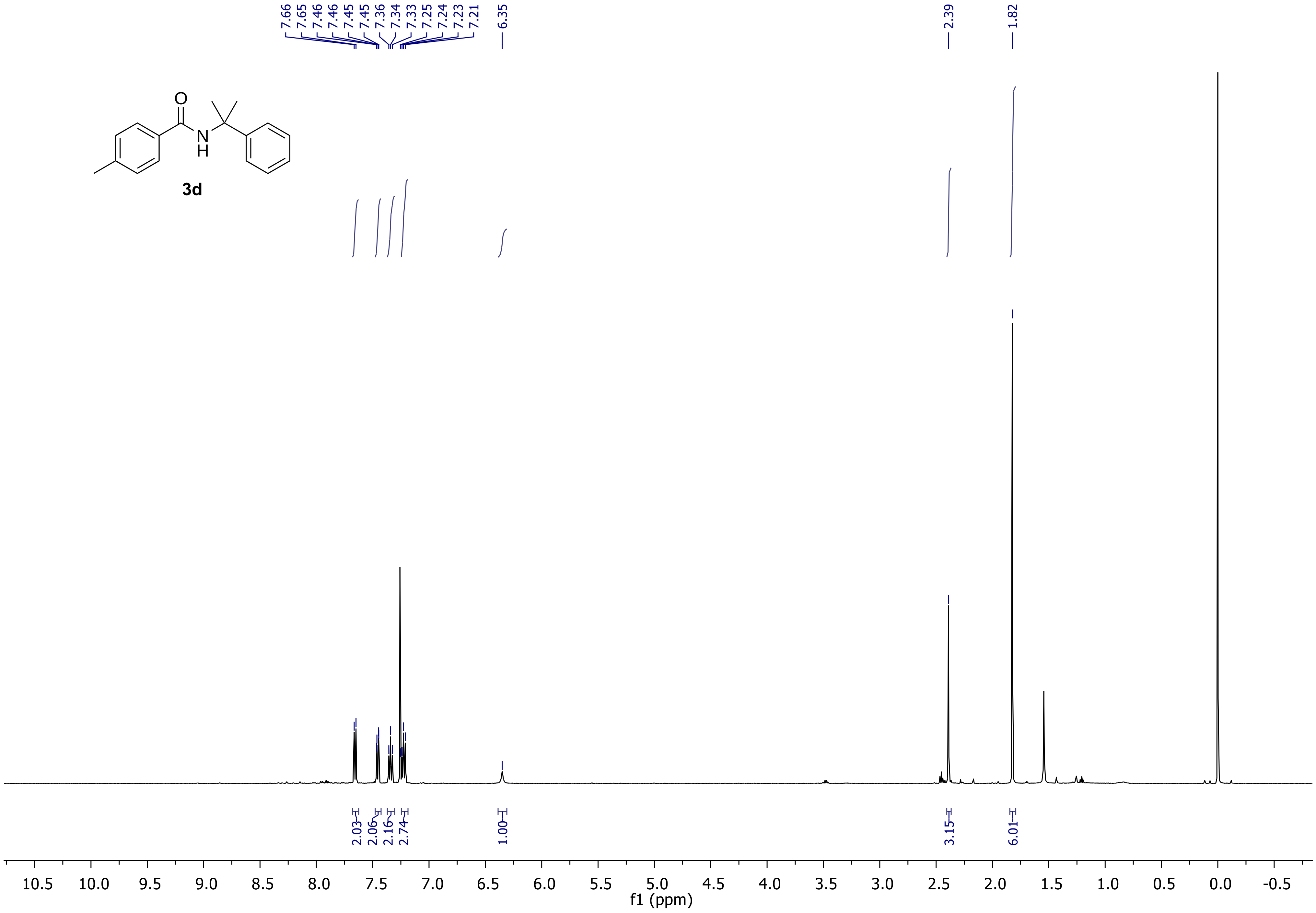

$^{13}C\{^{1}H\}$ NMR: 101 MHz, $CDCl_3$

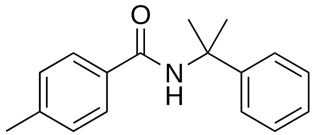

3d

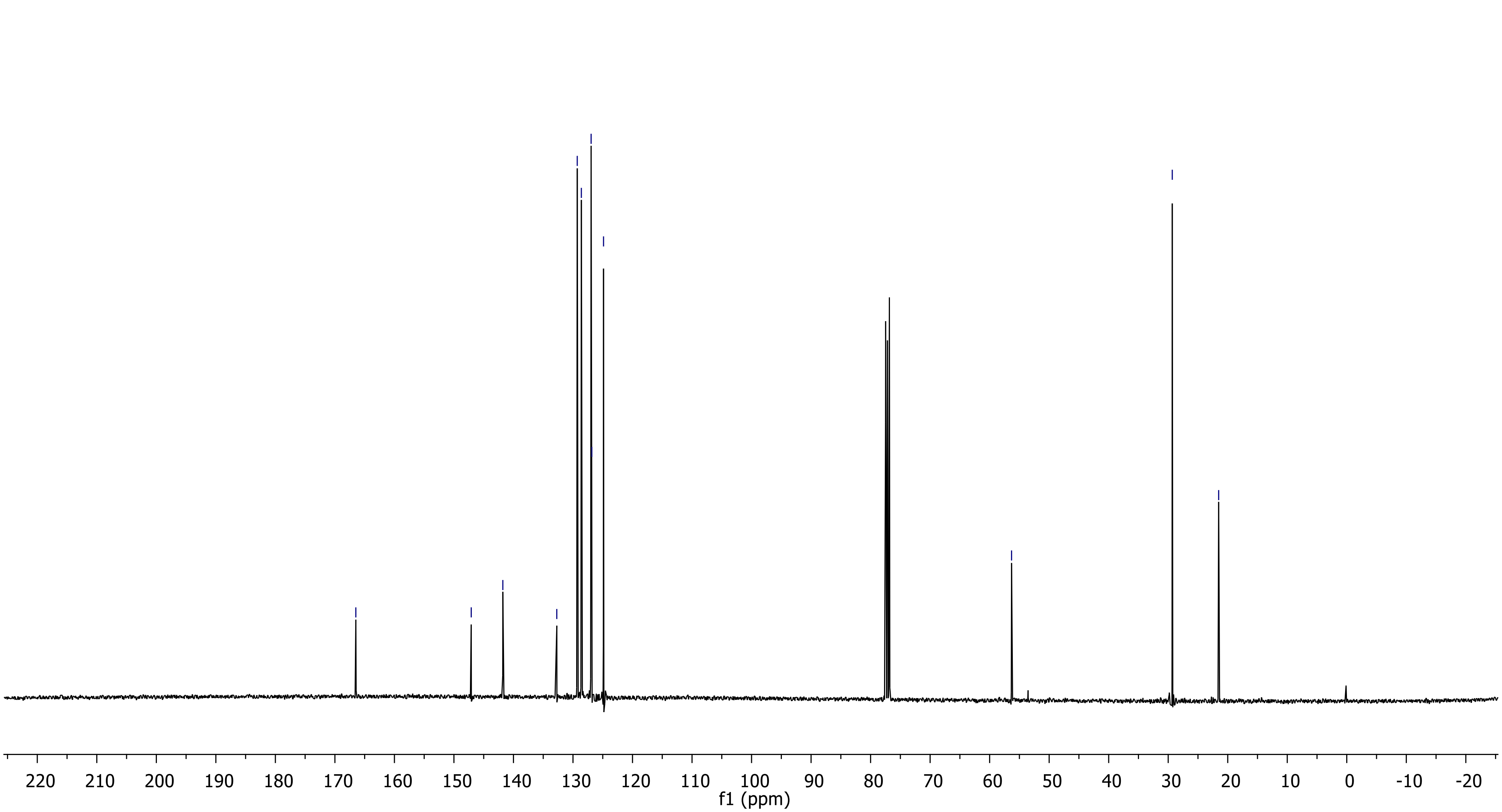

^1H NMR: 500 MHz, $CDCl_3$

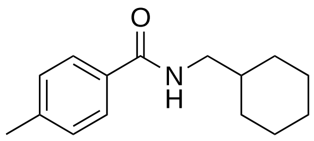

3e

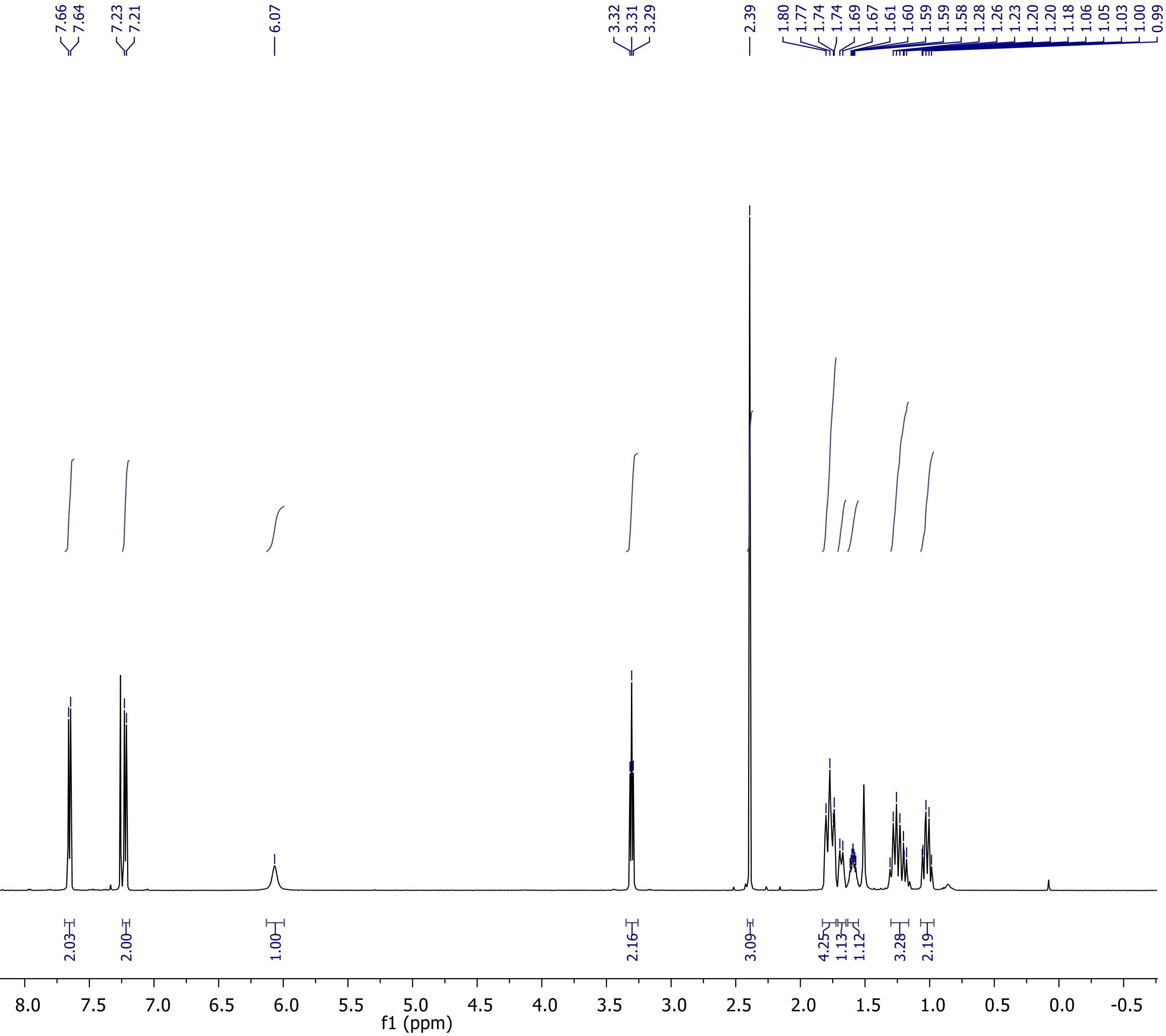

$^{13}C\{^{1}H\}$ NMR: 101 MHz, $CDCl_3$

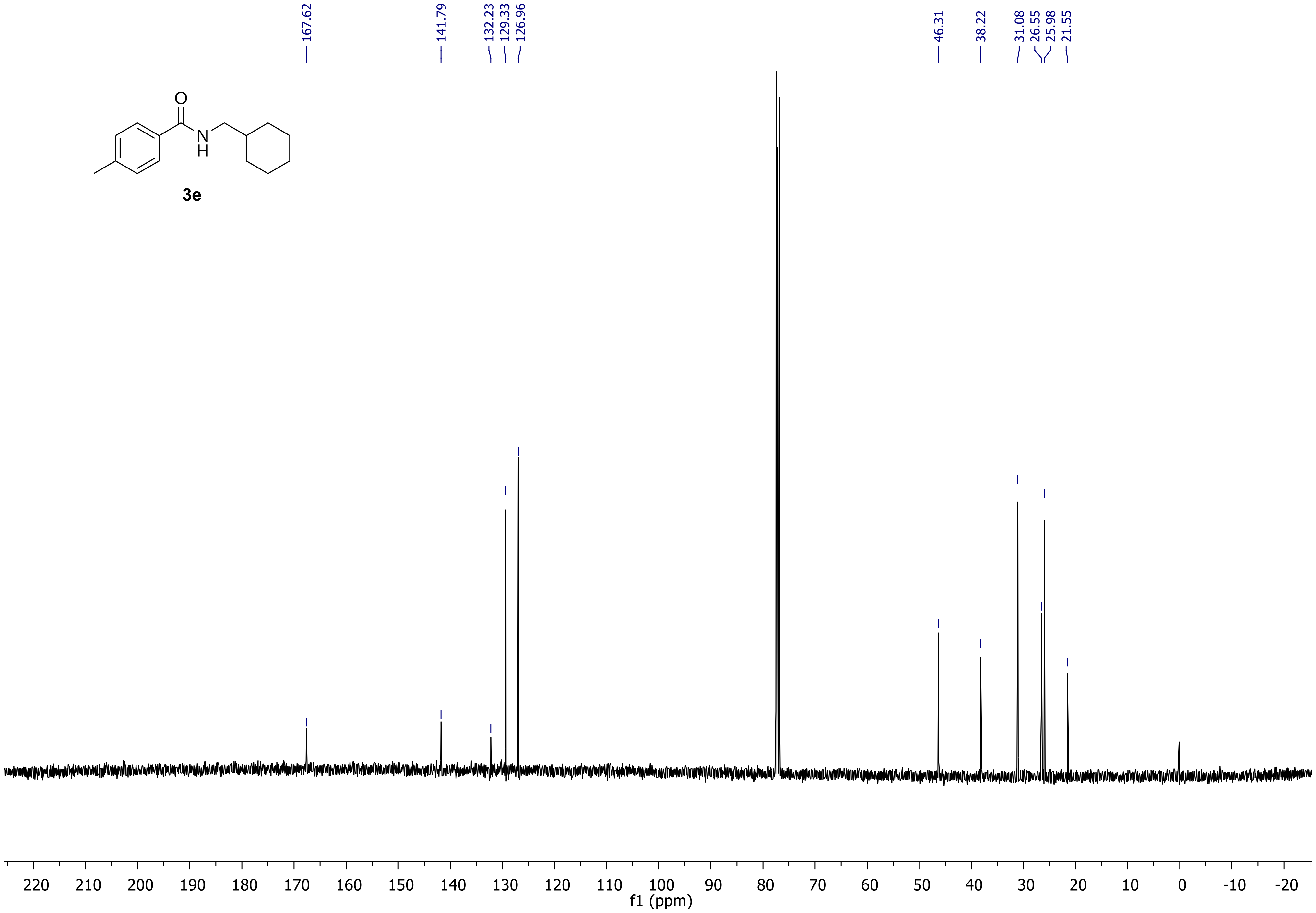

^{1}H NMR: 500 MHz, $CDCl_3$

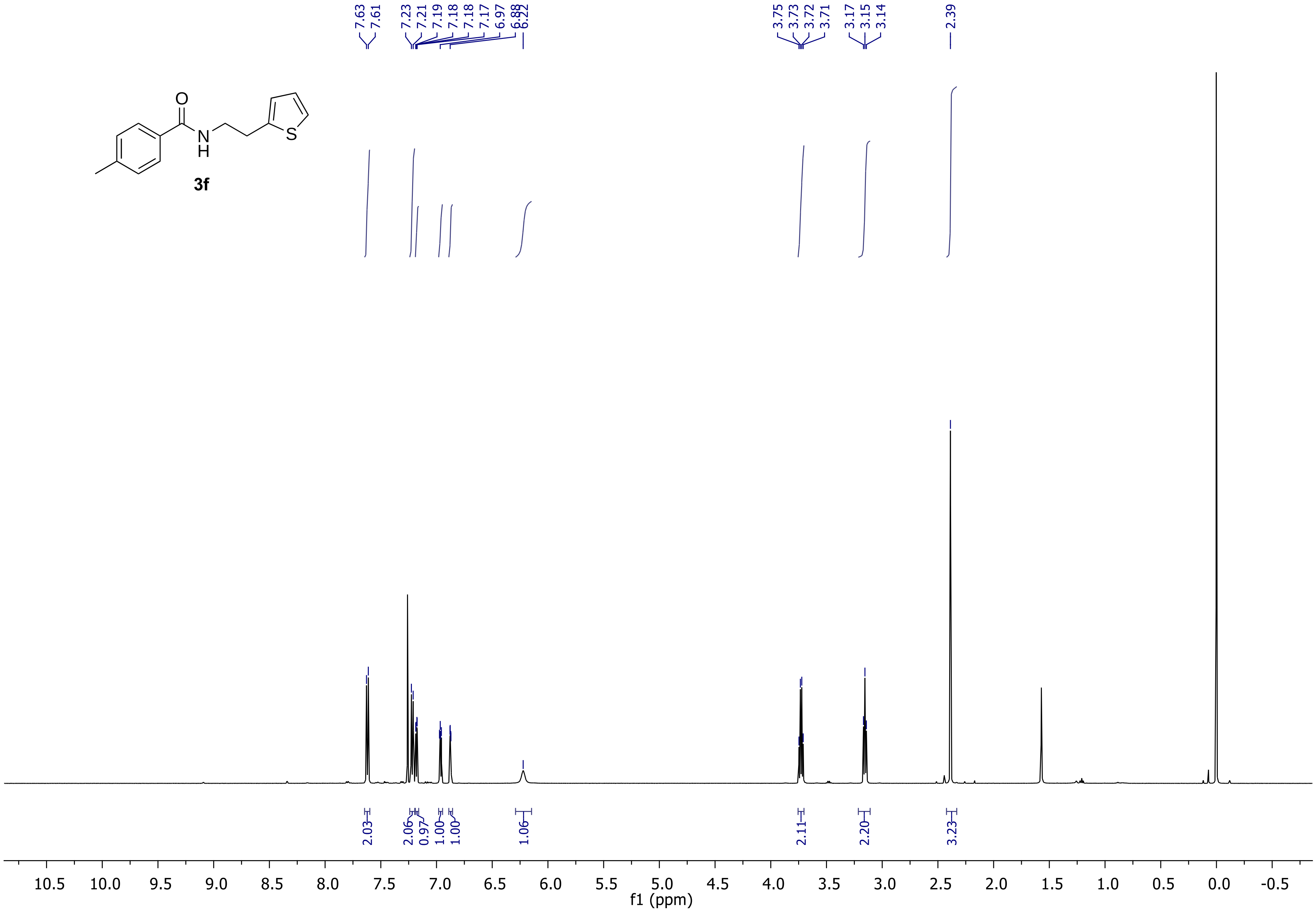

$^{13}C\{^{1}H\}$ NMR: 101 MHz, $CDCl_3$

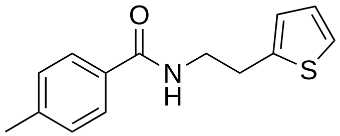

3f

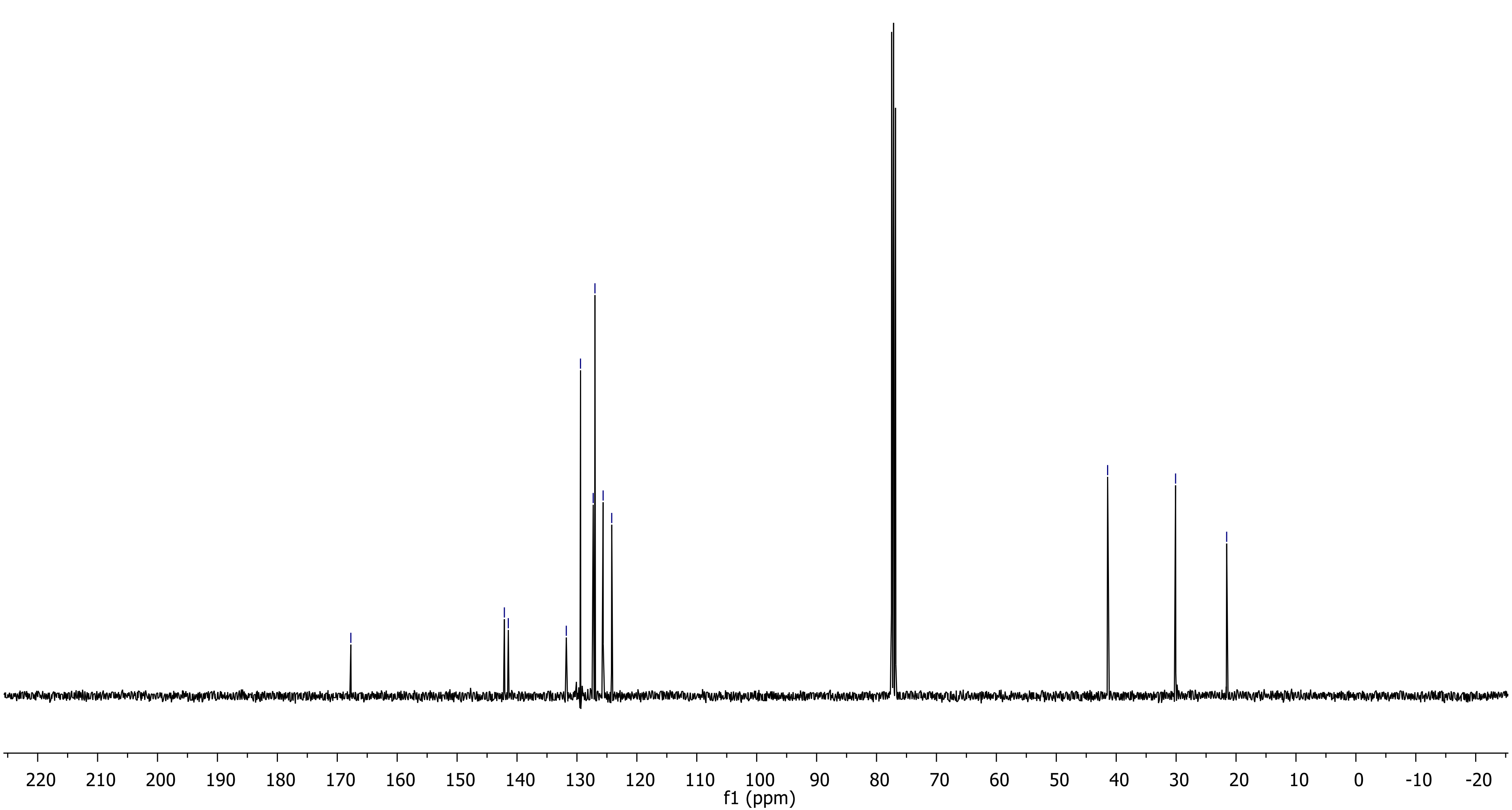

^{1}H NMR: 500 MHz, $CDCl_3$

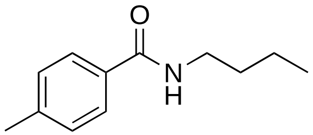

3g

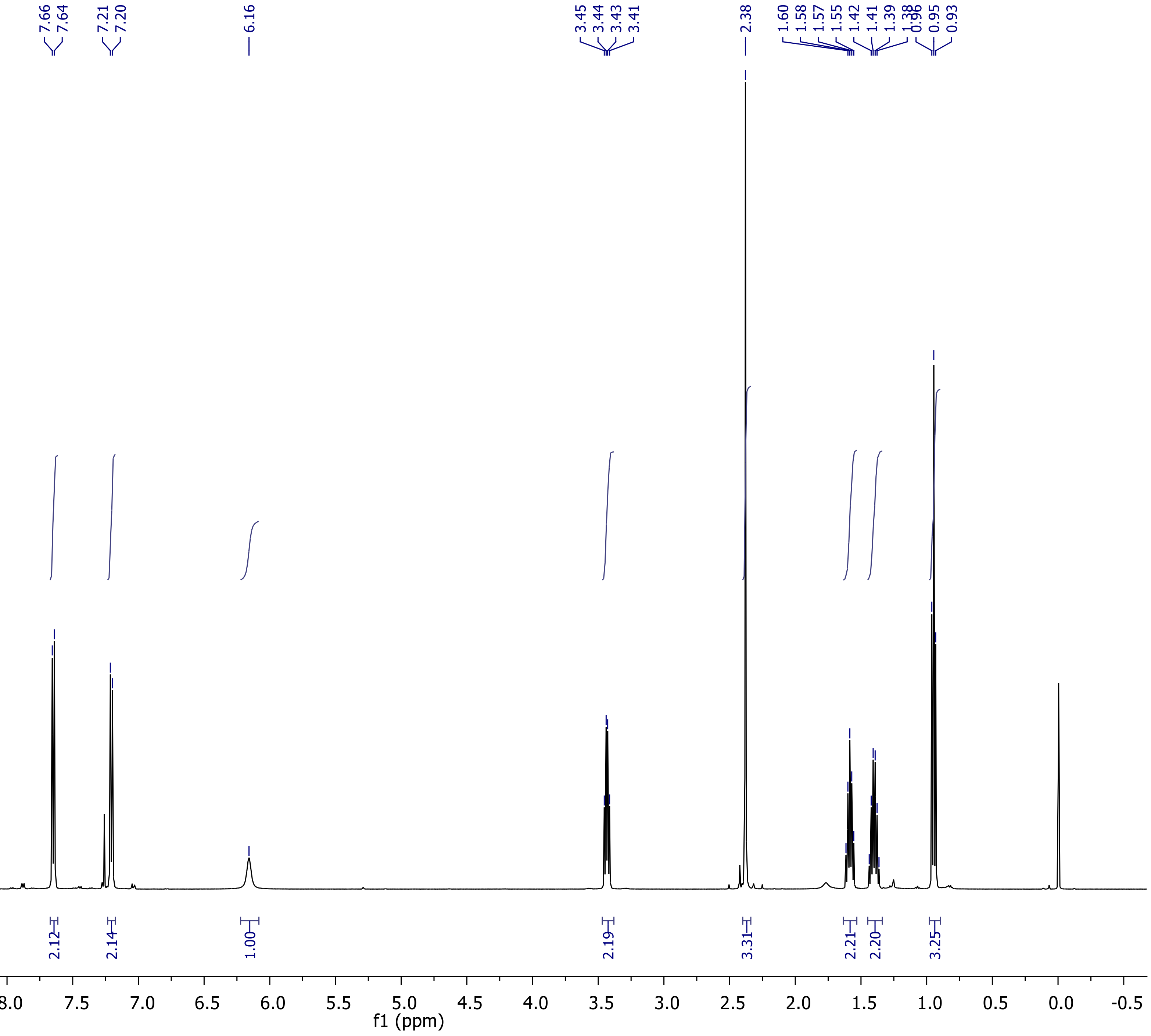

$^{13}C\{^1H\}$ NMR: 101 MHz, $CDCl_3$

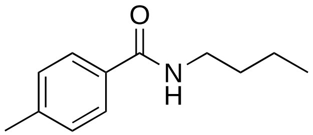

3g

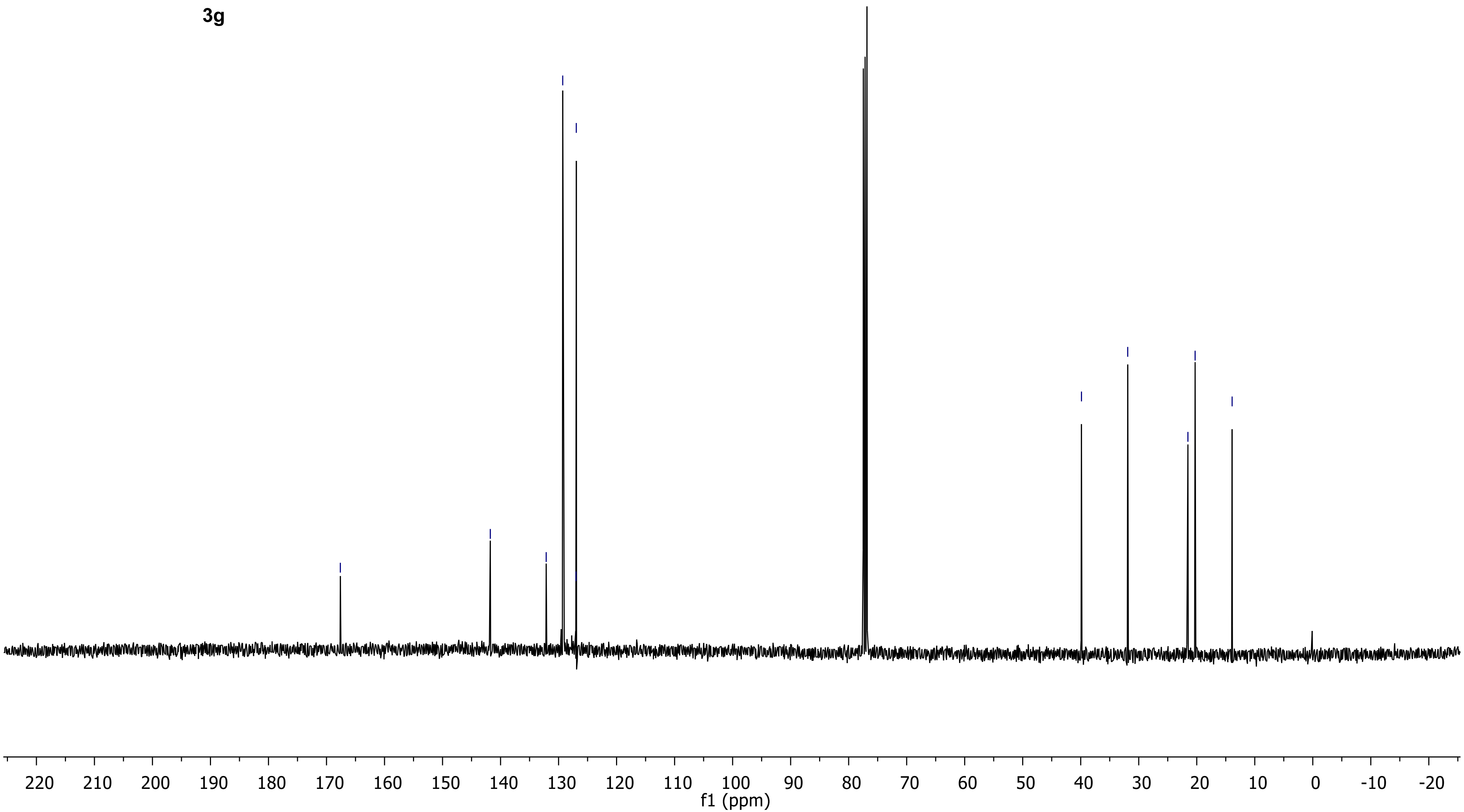

^{1}H NMR: 500 MHz, D_6-DMSO, 100 °C

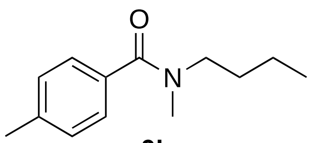
3h

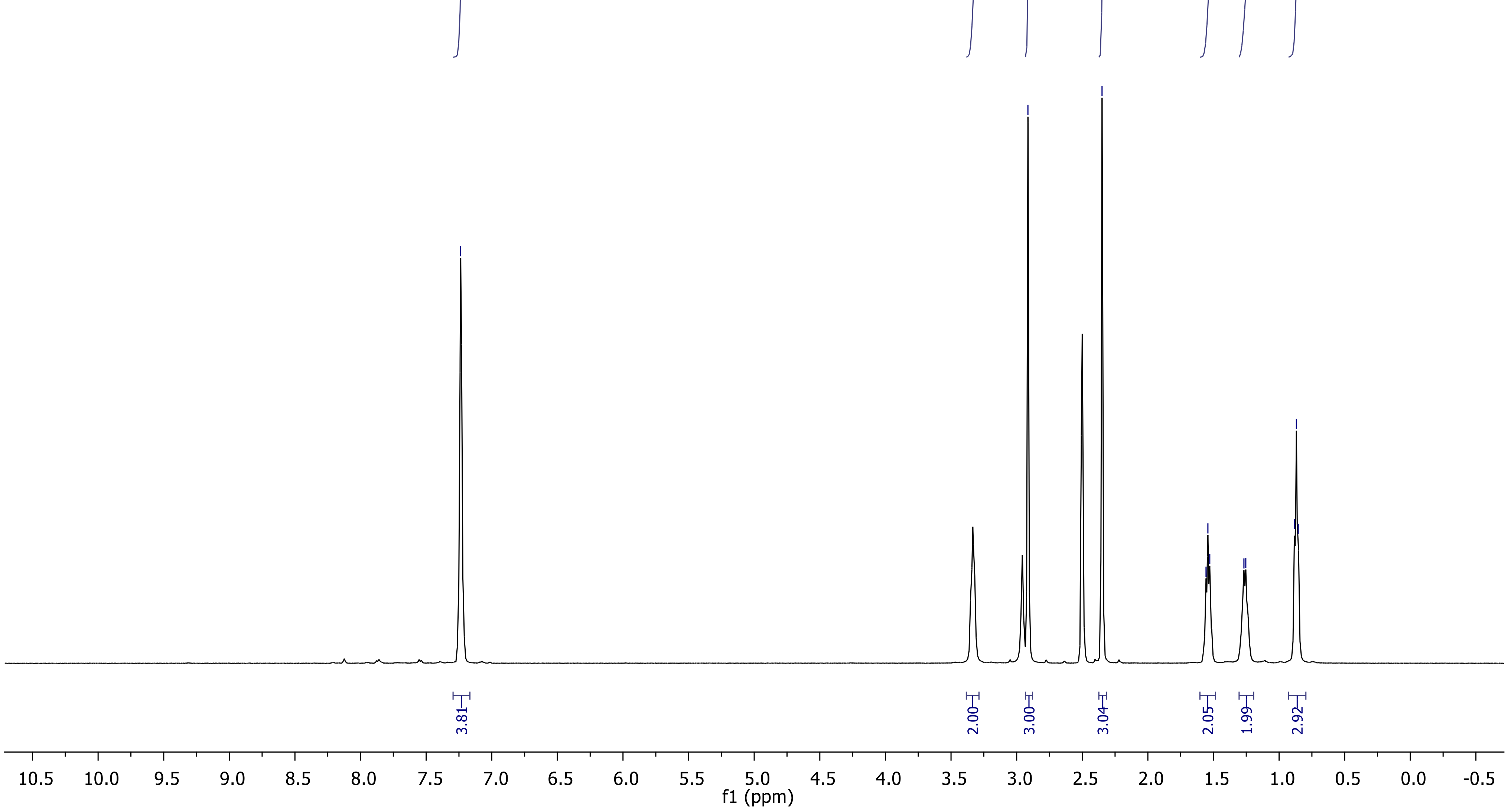

$^{13}C\{^1H\}$ NMR: 126 MHz, D6-DMSO, 100 °C

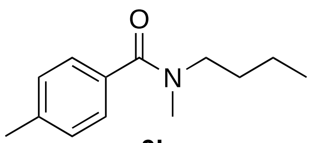
3h

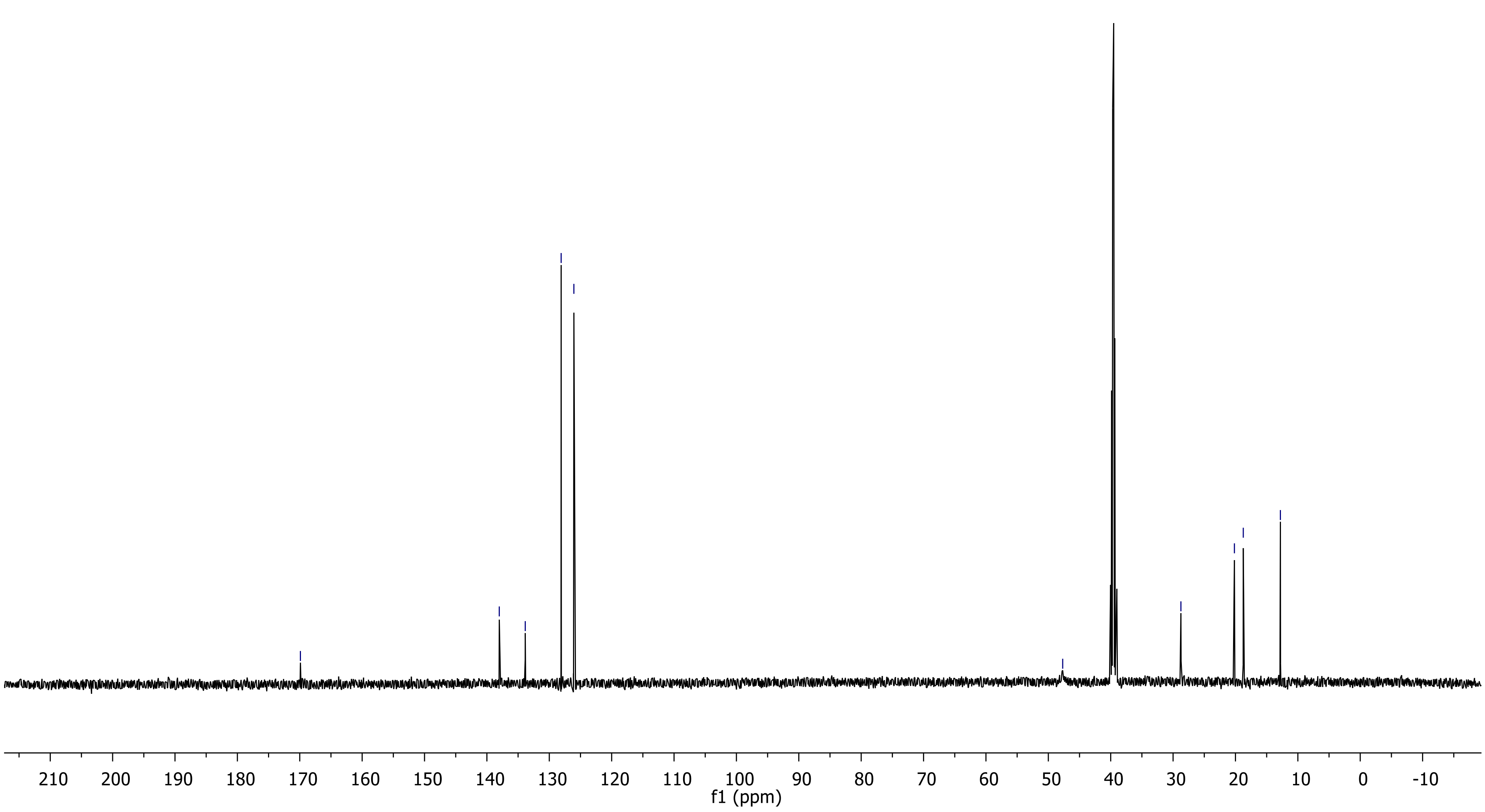

^{1}H NMR: 500 MHz, D_6-DMSO, 100 °C

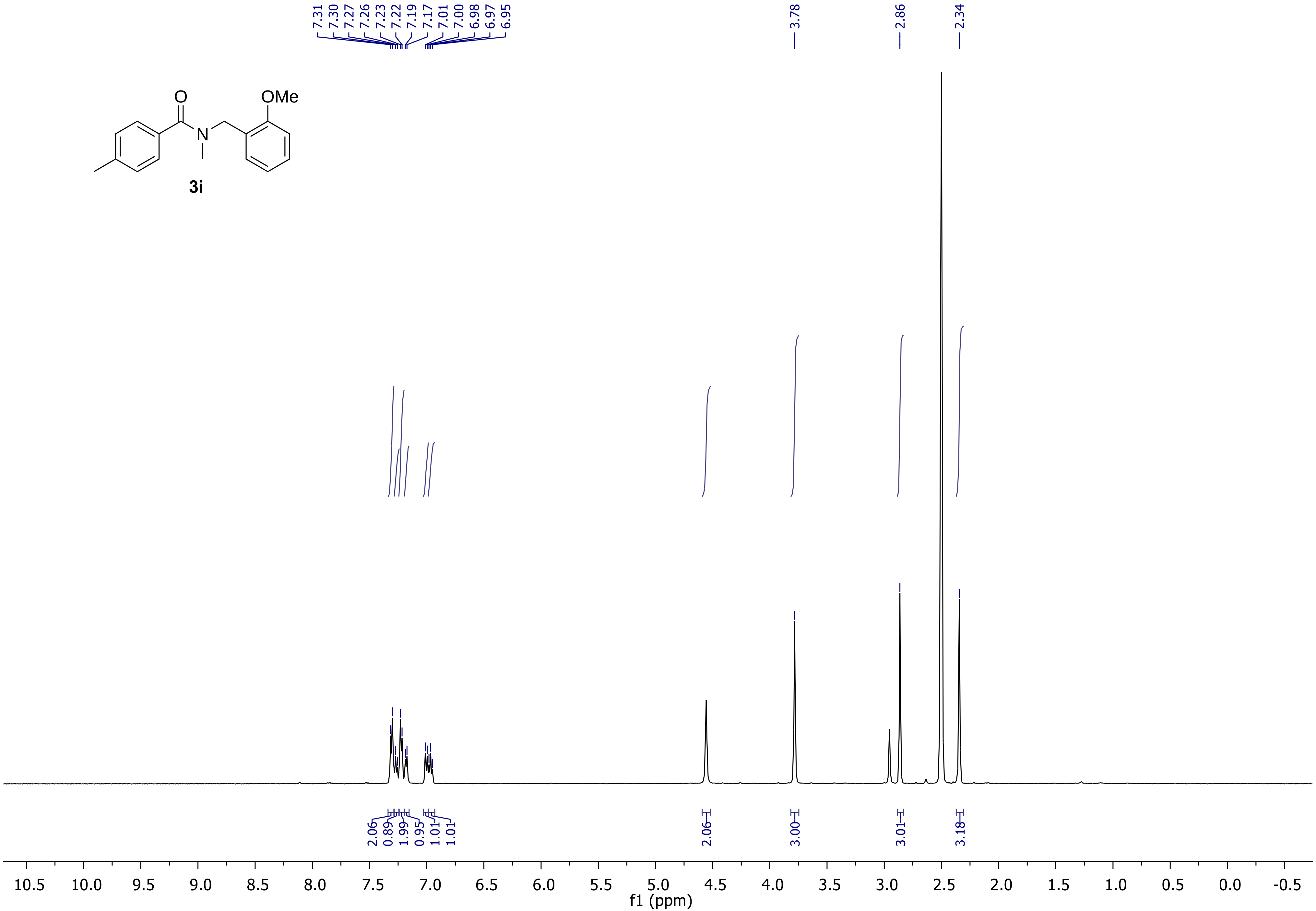

3i

$^{13}C\{^1H\}$ NMR: 126 MHz, D_6-DMSO, 100 °C

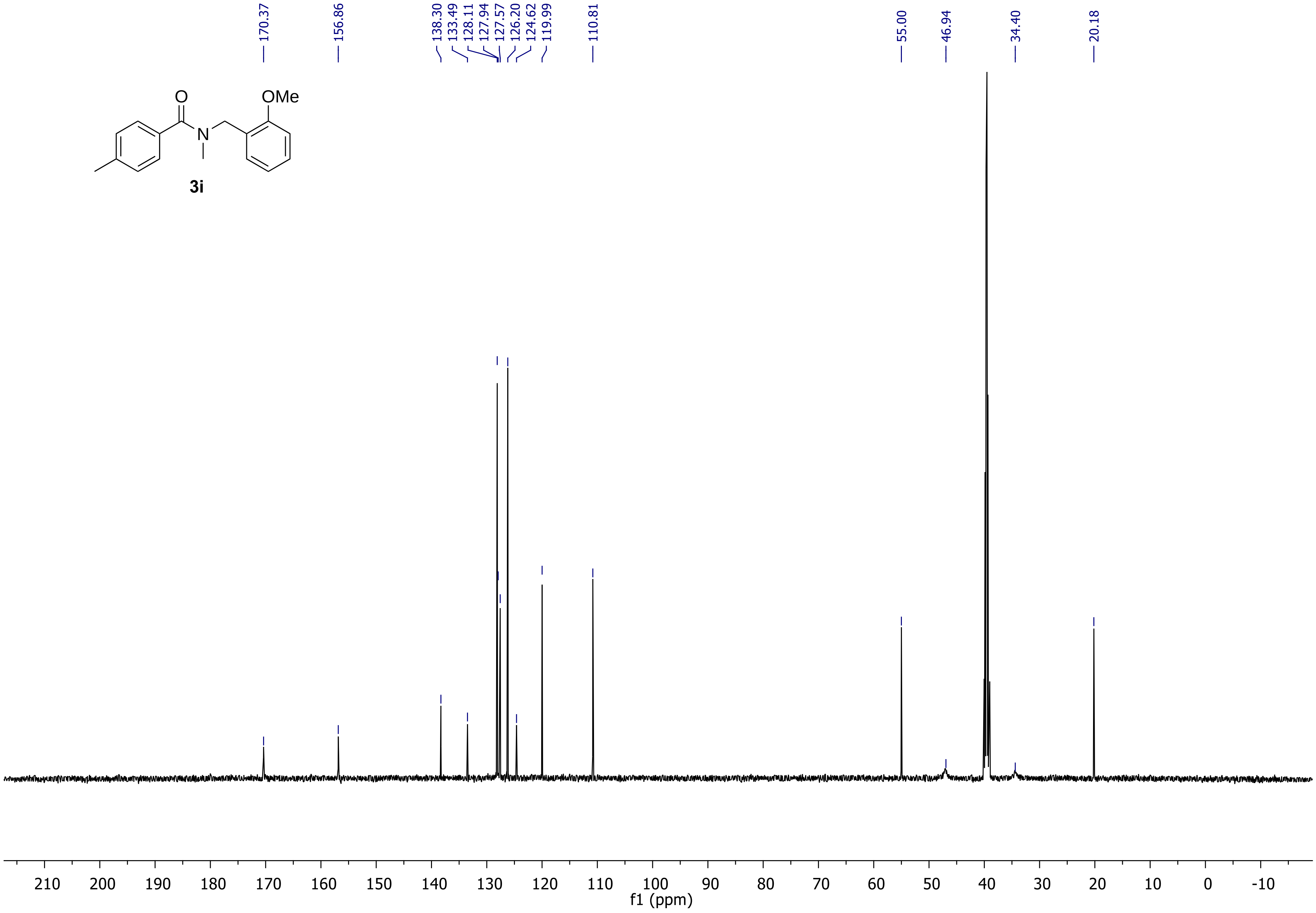

^{1}H NMR: 500 MHz, D_6-DMSO, 100 °C

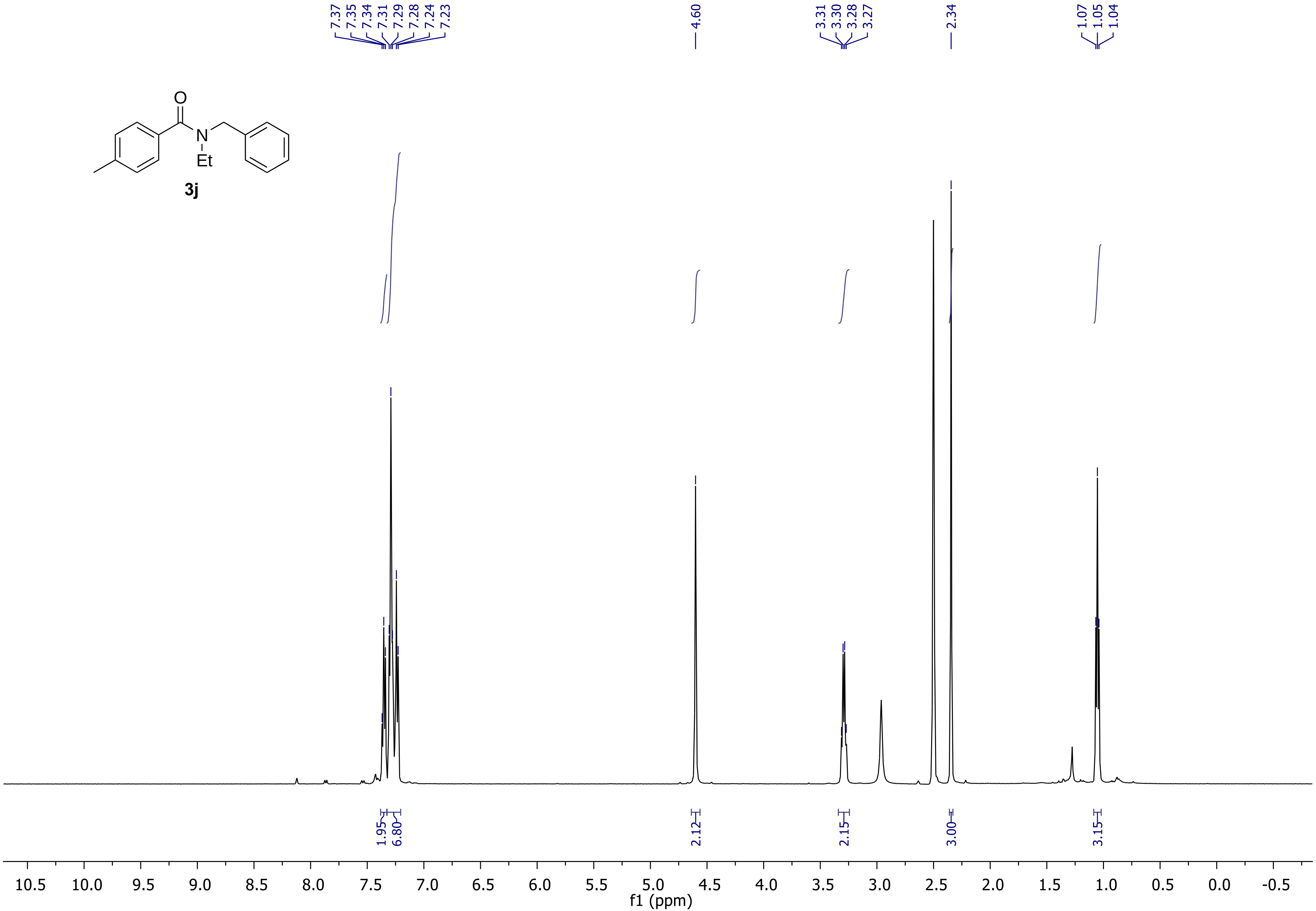

$^{13}C\{^1H\}$ NMR: 126 MHz, D_6-DMSO, 100 °C

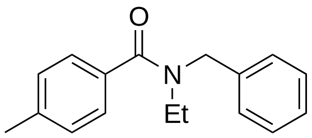

3j

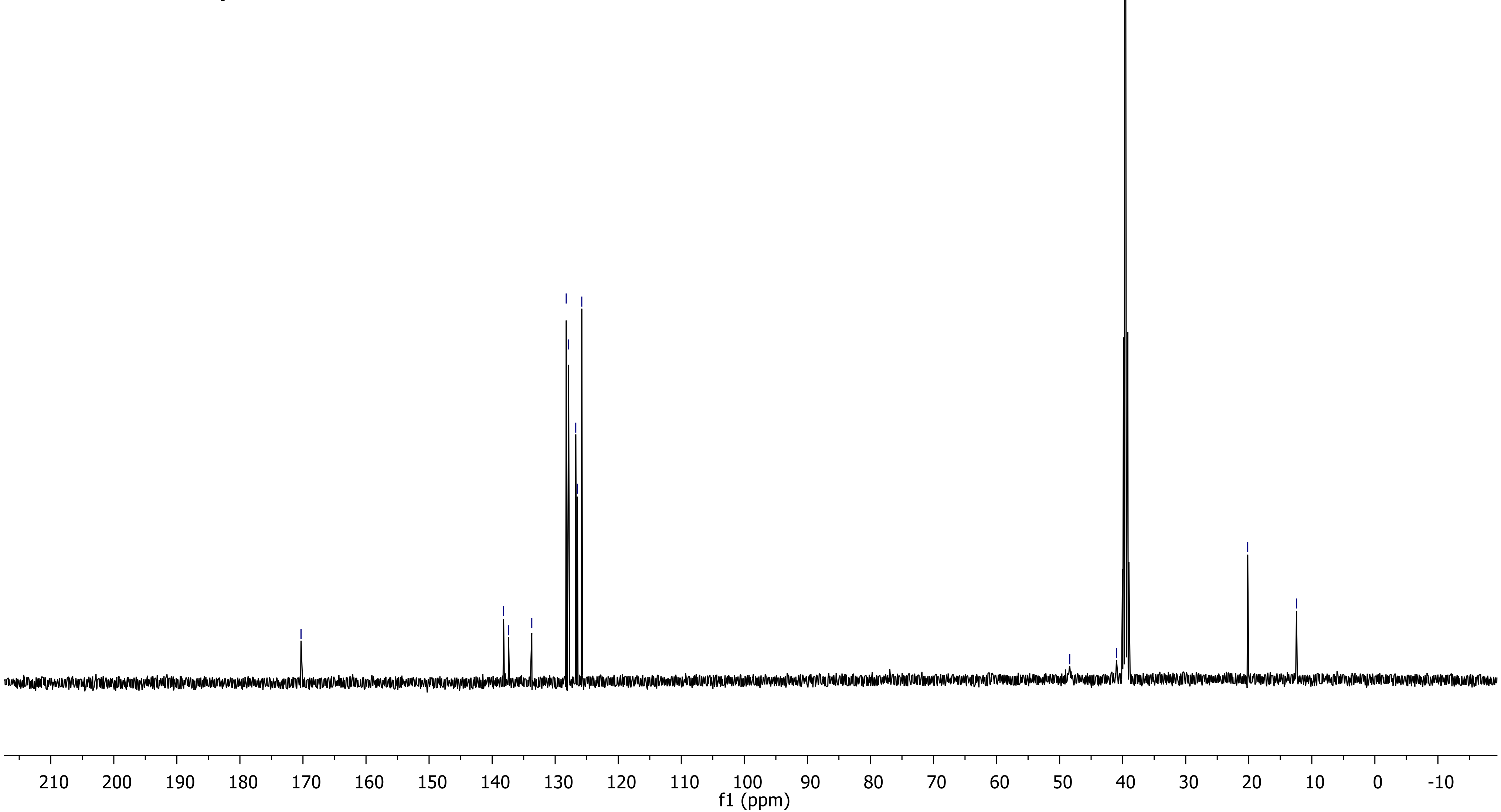

^{1}H NMR: 500 MHz, $CDCl_3$

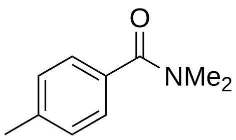

3k

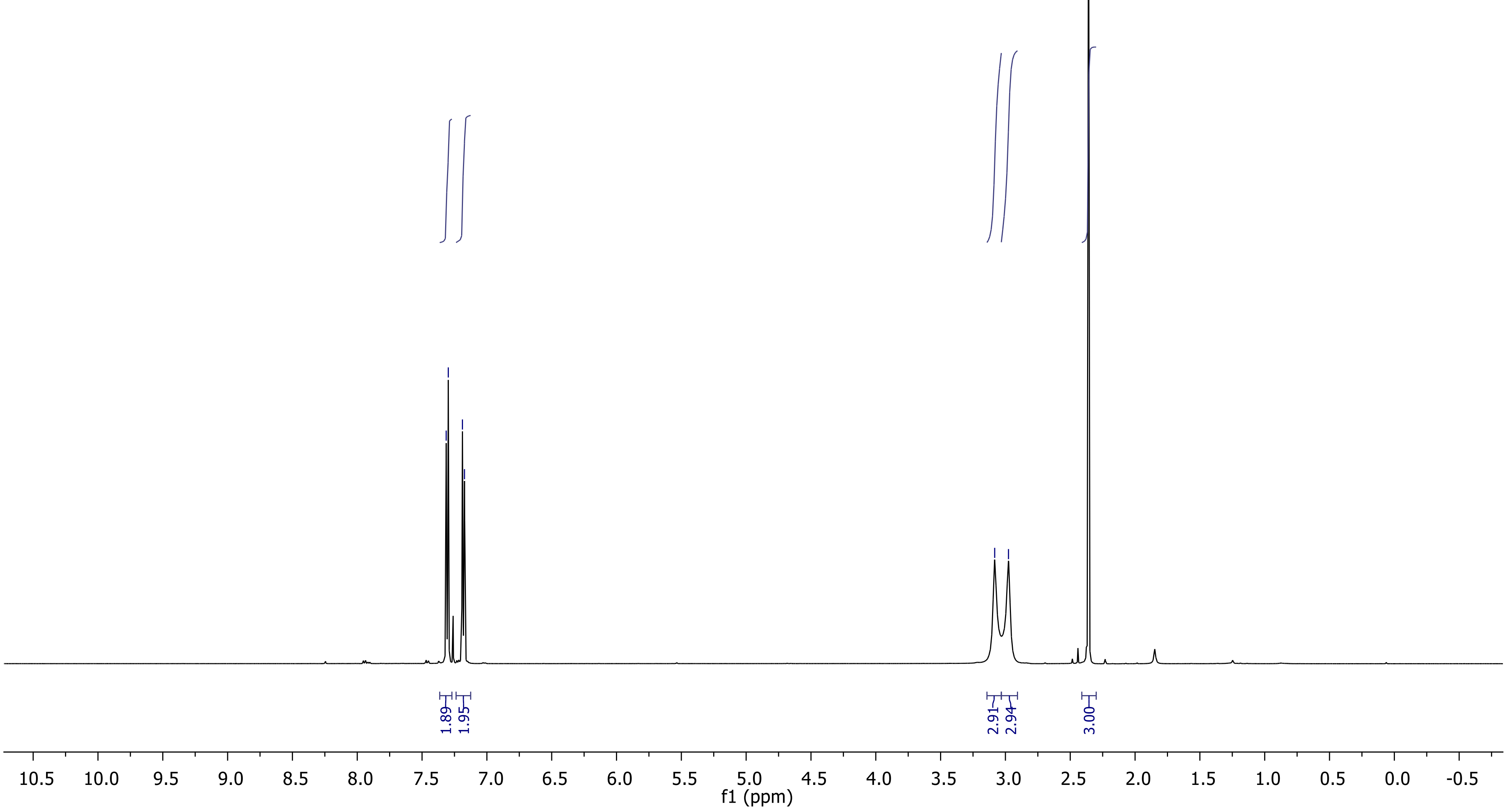

$^{13}C\{^1H\}$ NMR: 126 MHz, $CDCl_3$

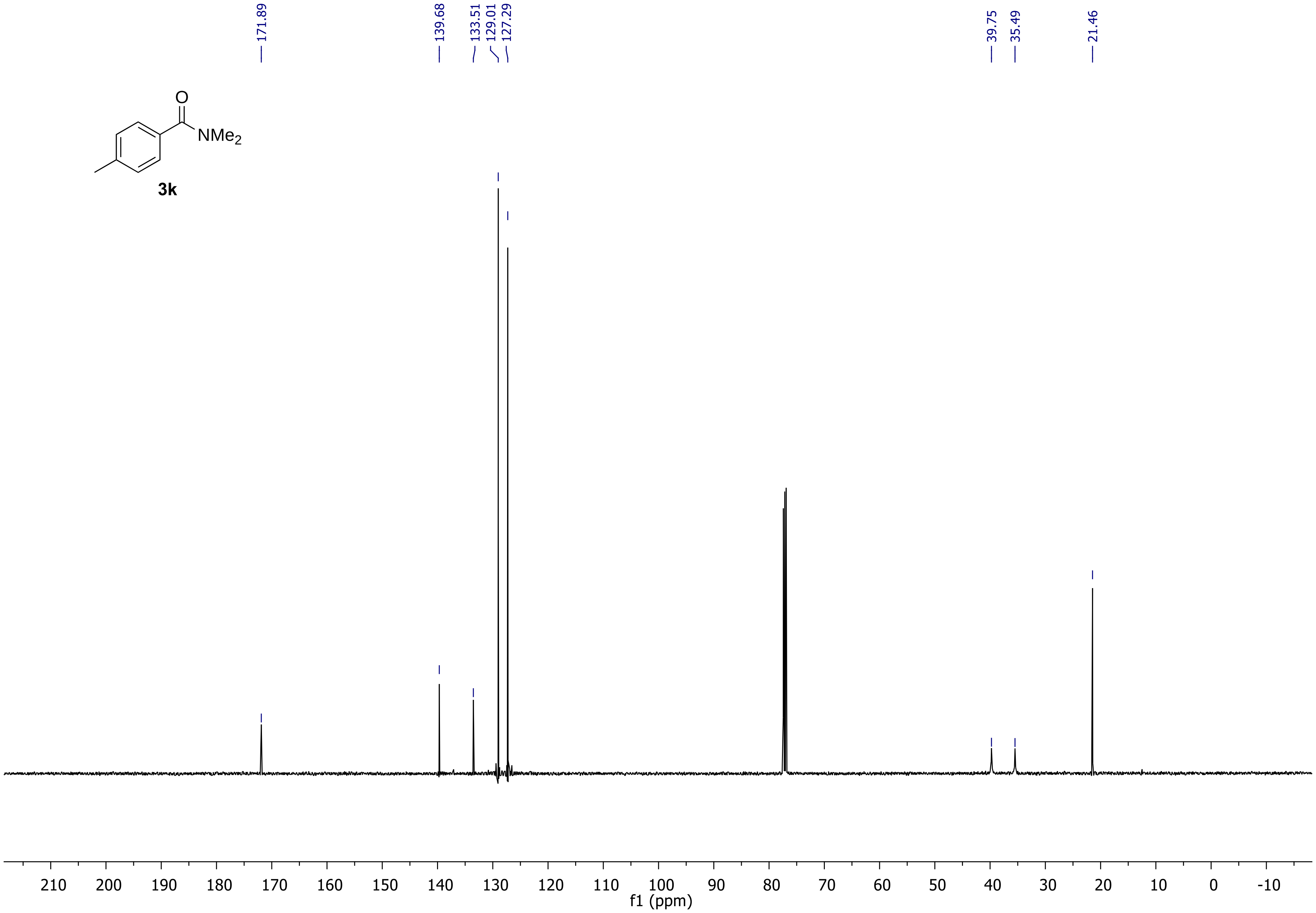

^{1}H NMR: 500 MHz, $CDCl_3$, 50 °C

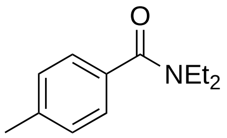

3l

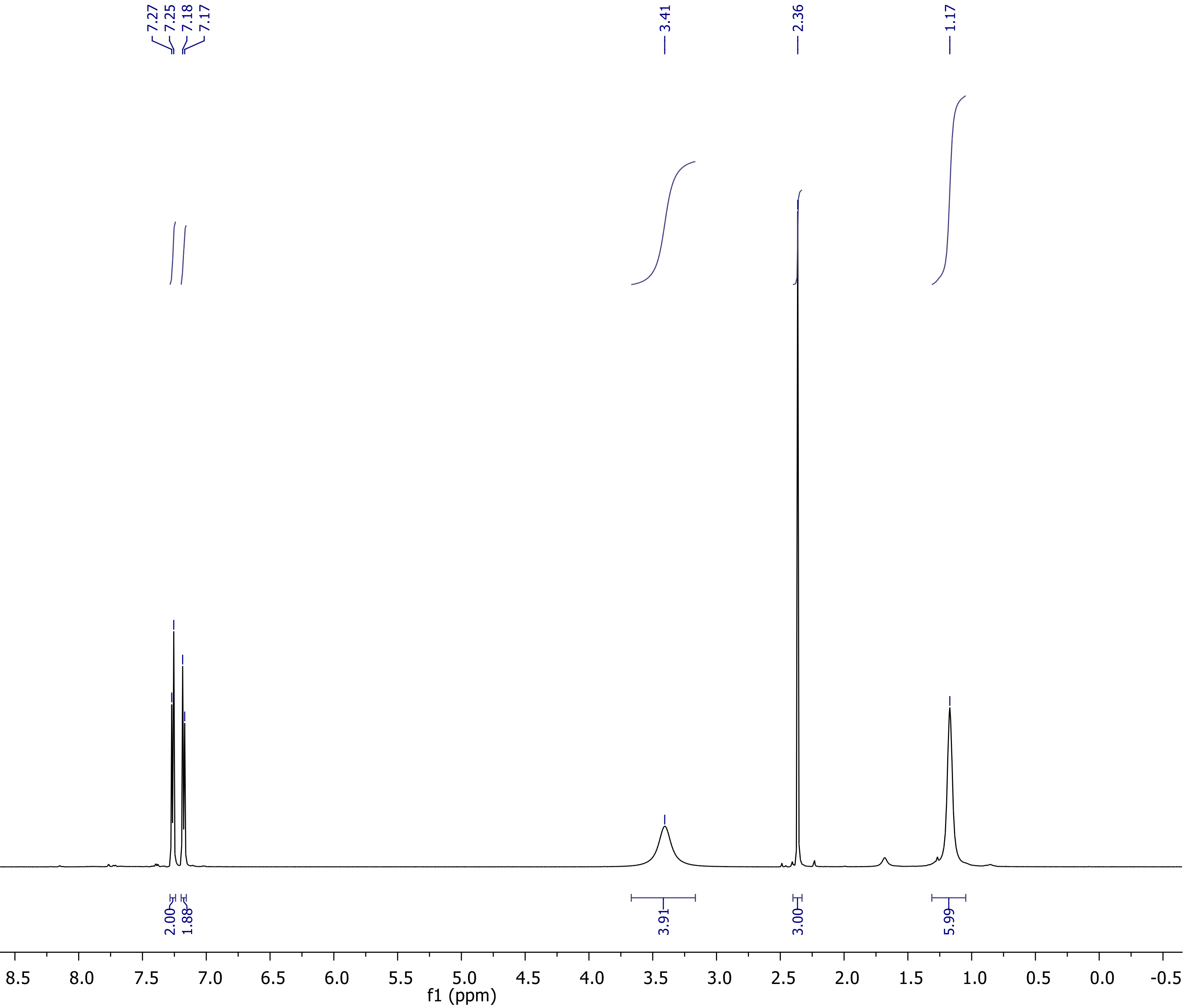

$^{13}C\{^{1}H\}$ NMR: 126 MHz, $CDCl_3$, 50 °C

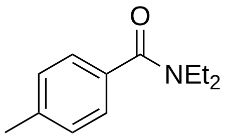

3l

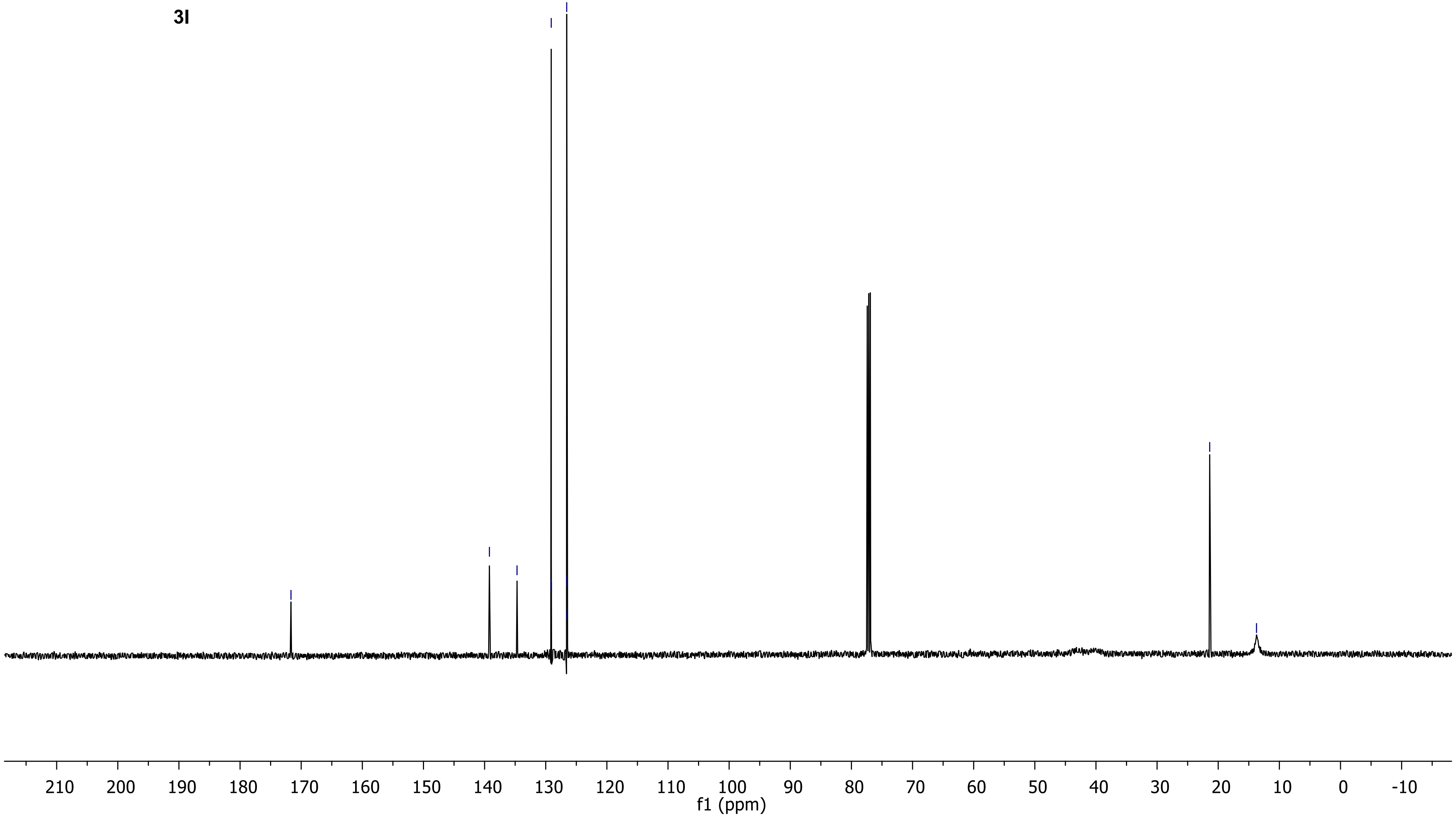

^{1}H NMR: 500 MHz, $CDCl_3$

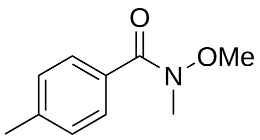

3m

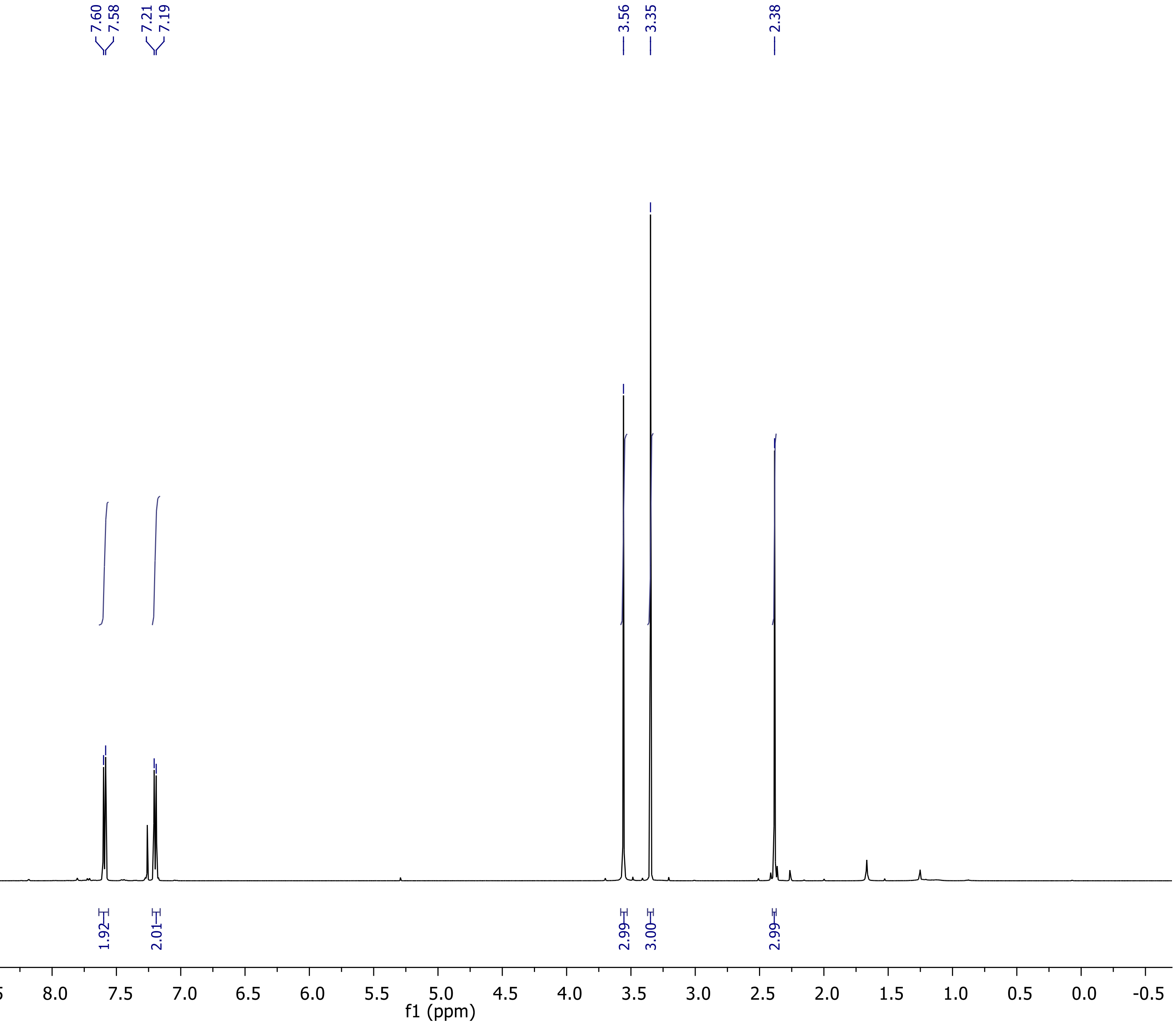

$^{13}C\{^{1}H\}$ NMR: 126 MHz, $CDCl_3$

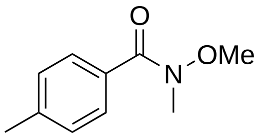

3m

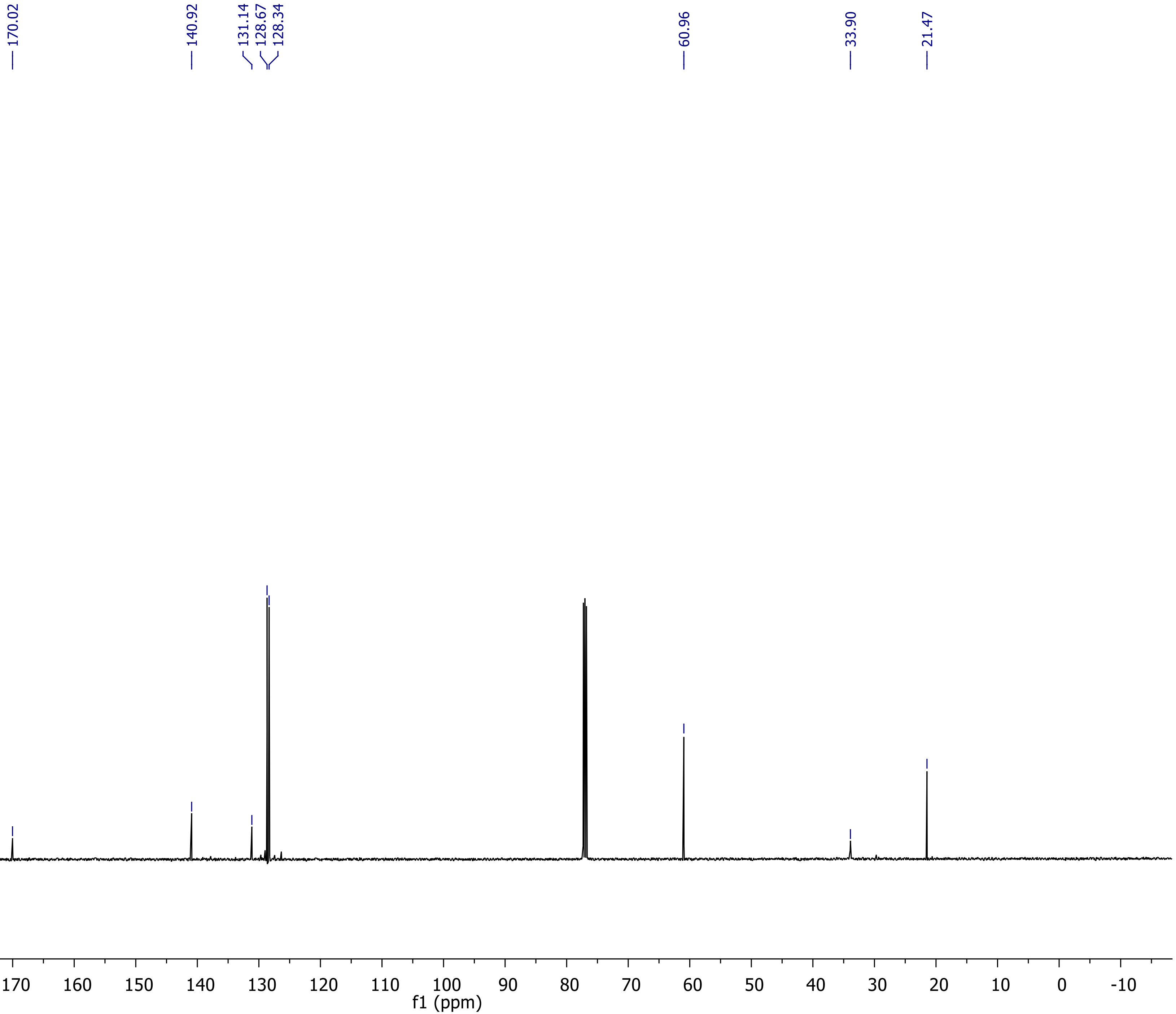

^{1}H NMR: 500 MHz, $CDCl_3$, 50 °C

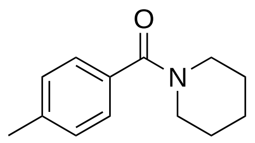

3n

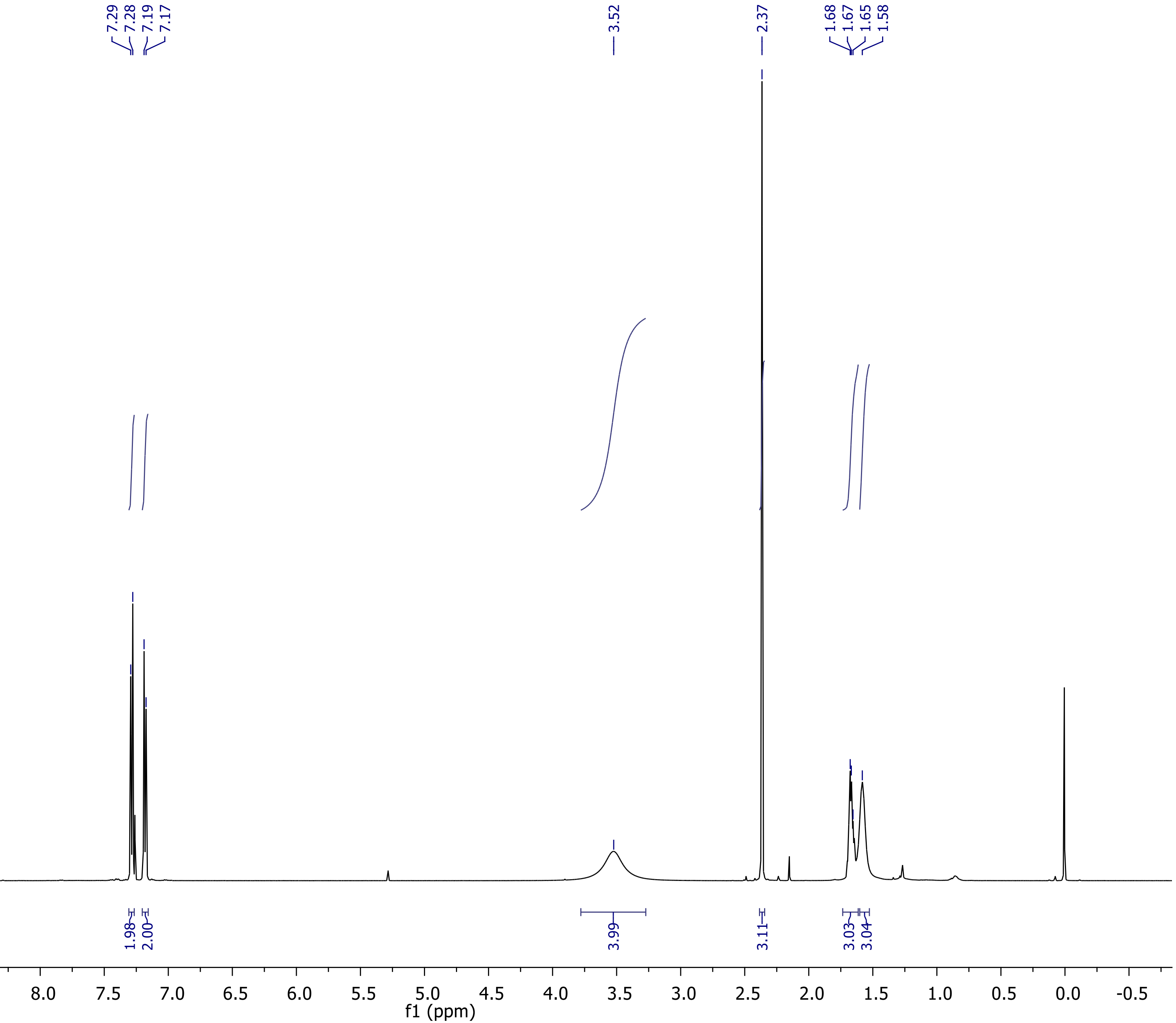

$^{13}C\{^1H\}$ NMR: 126 MHz, $CDCl_3$

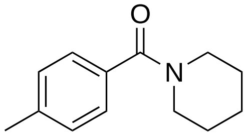

3n

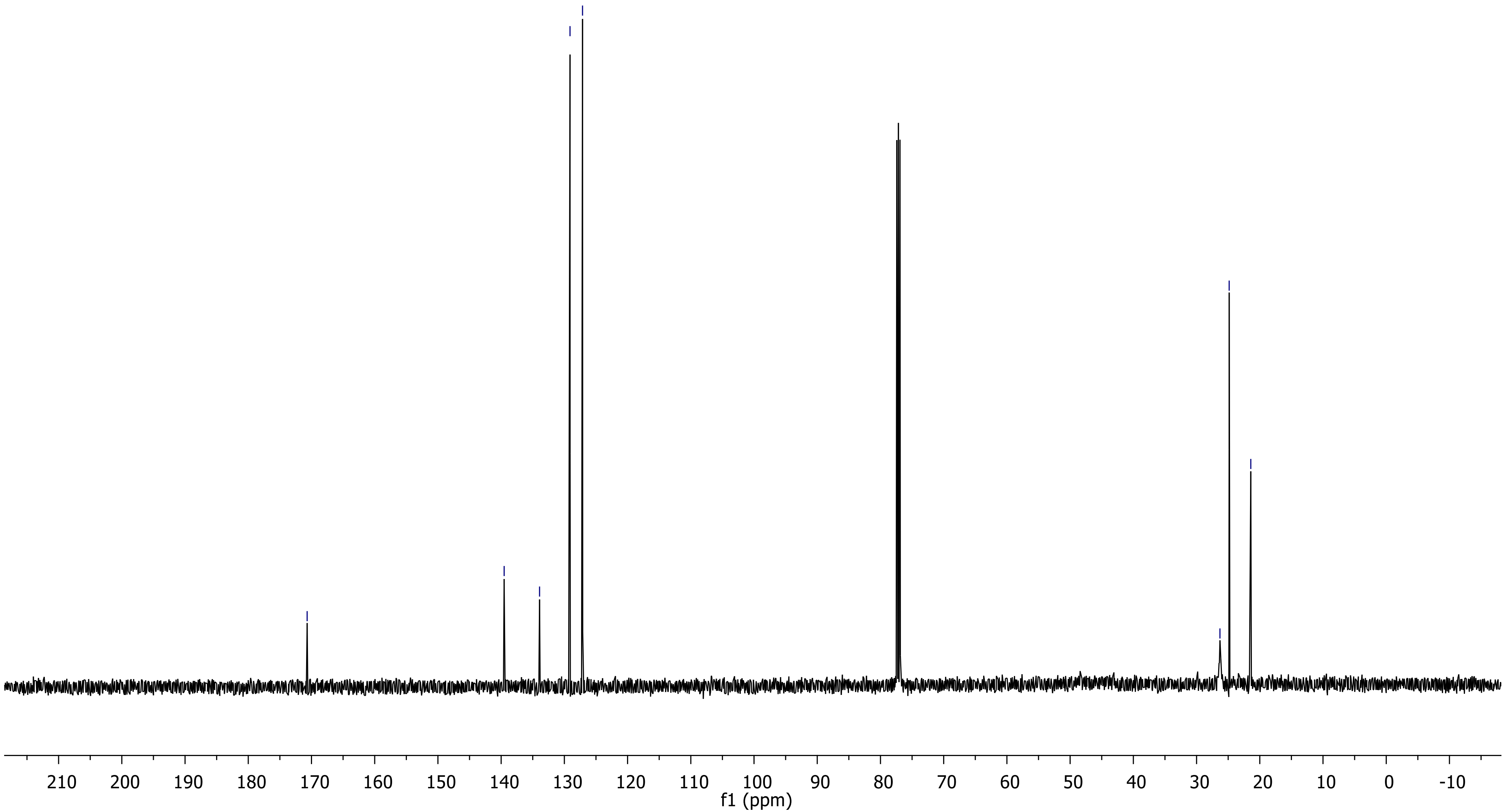

^{1}H NMR: 500 MHz, CD_3OD

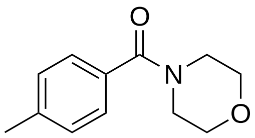
3o

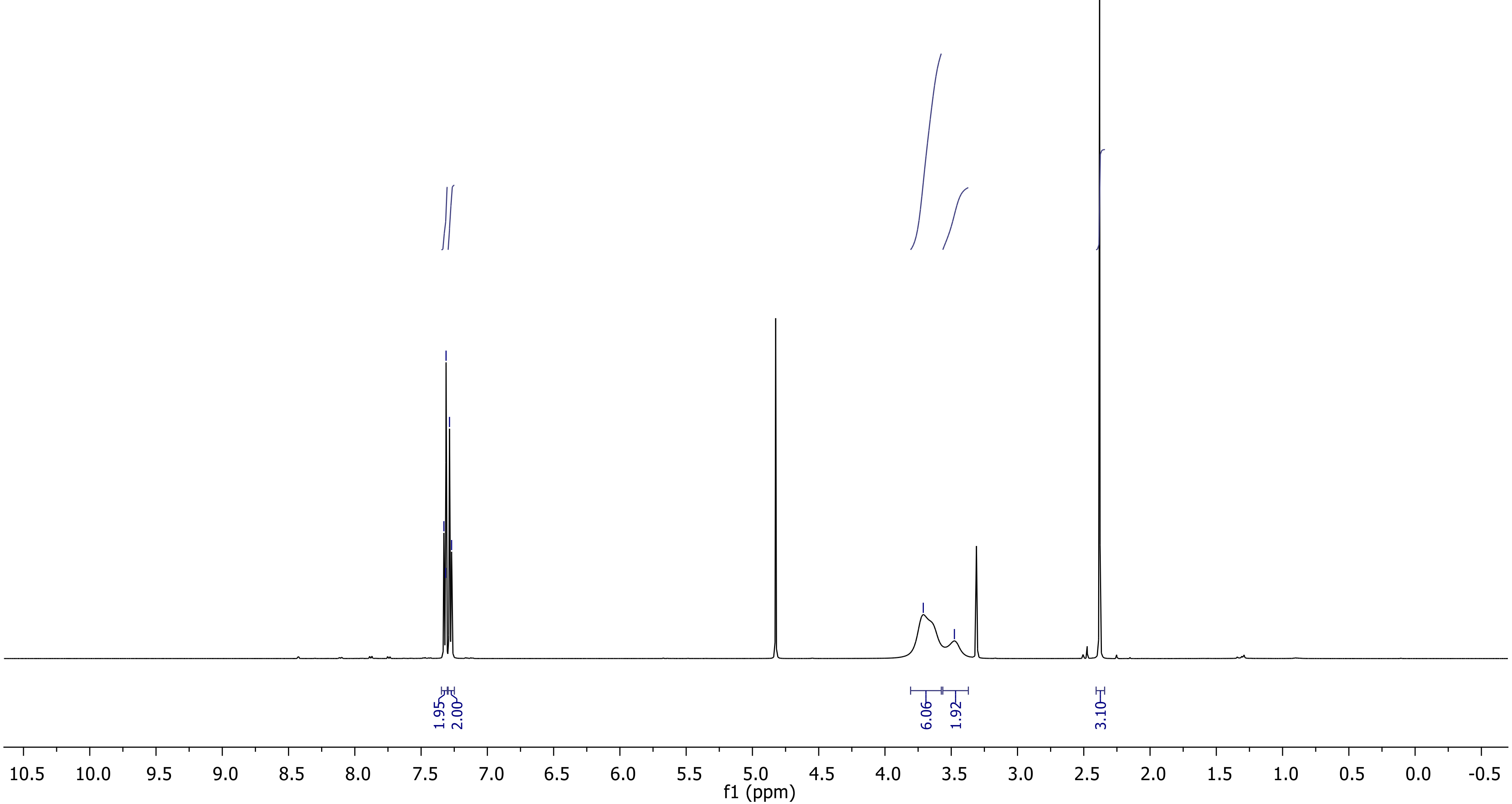

$^{13}C\{^{1}H\}$ NMR: 126 MHz, CD_3OD

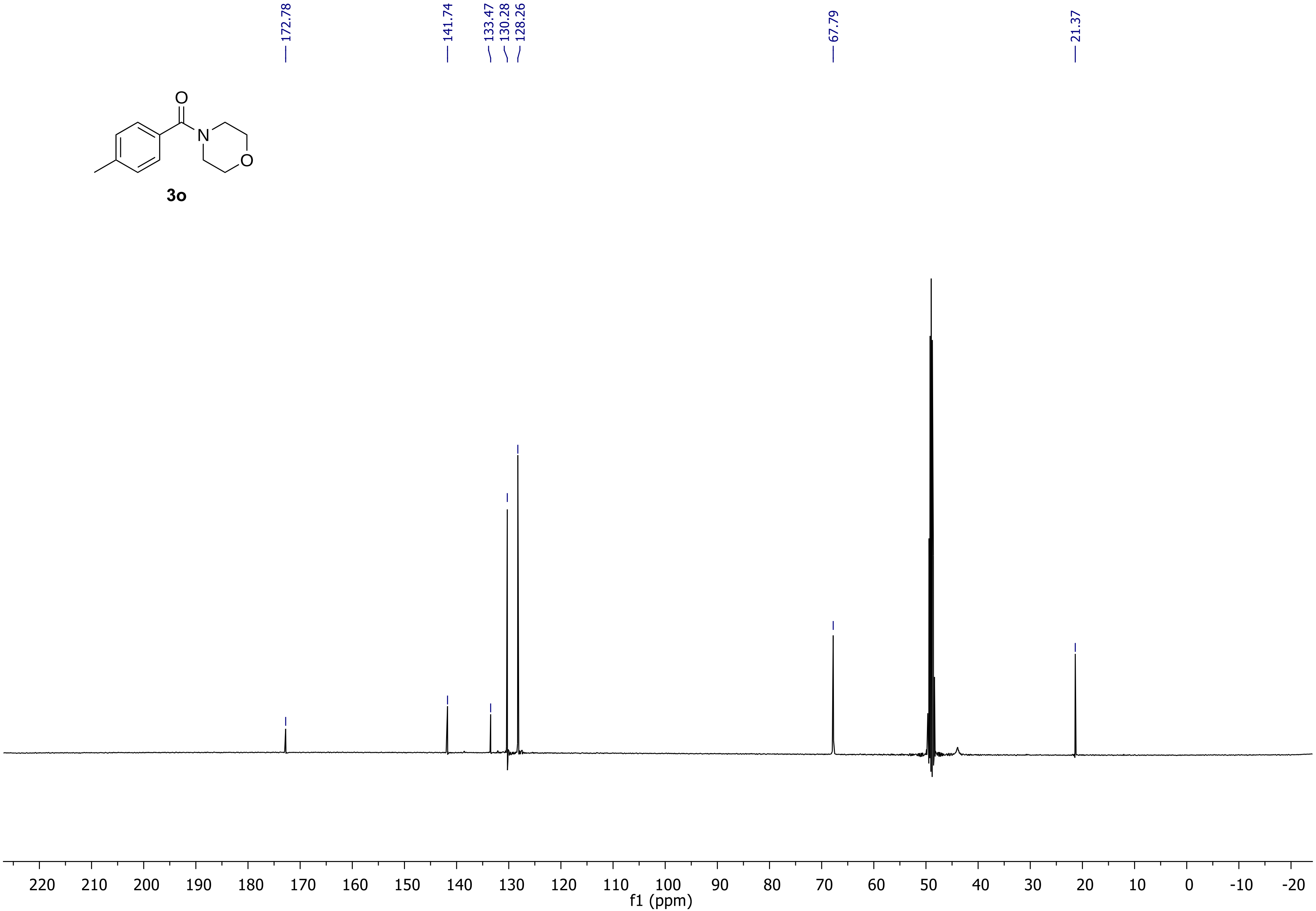

^1H NMR: 400 MHz, $CDCl_3$

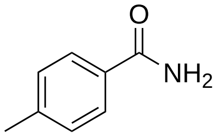

3p

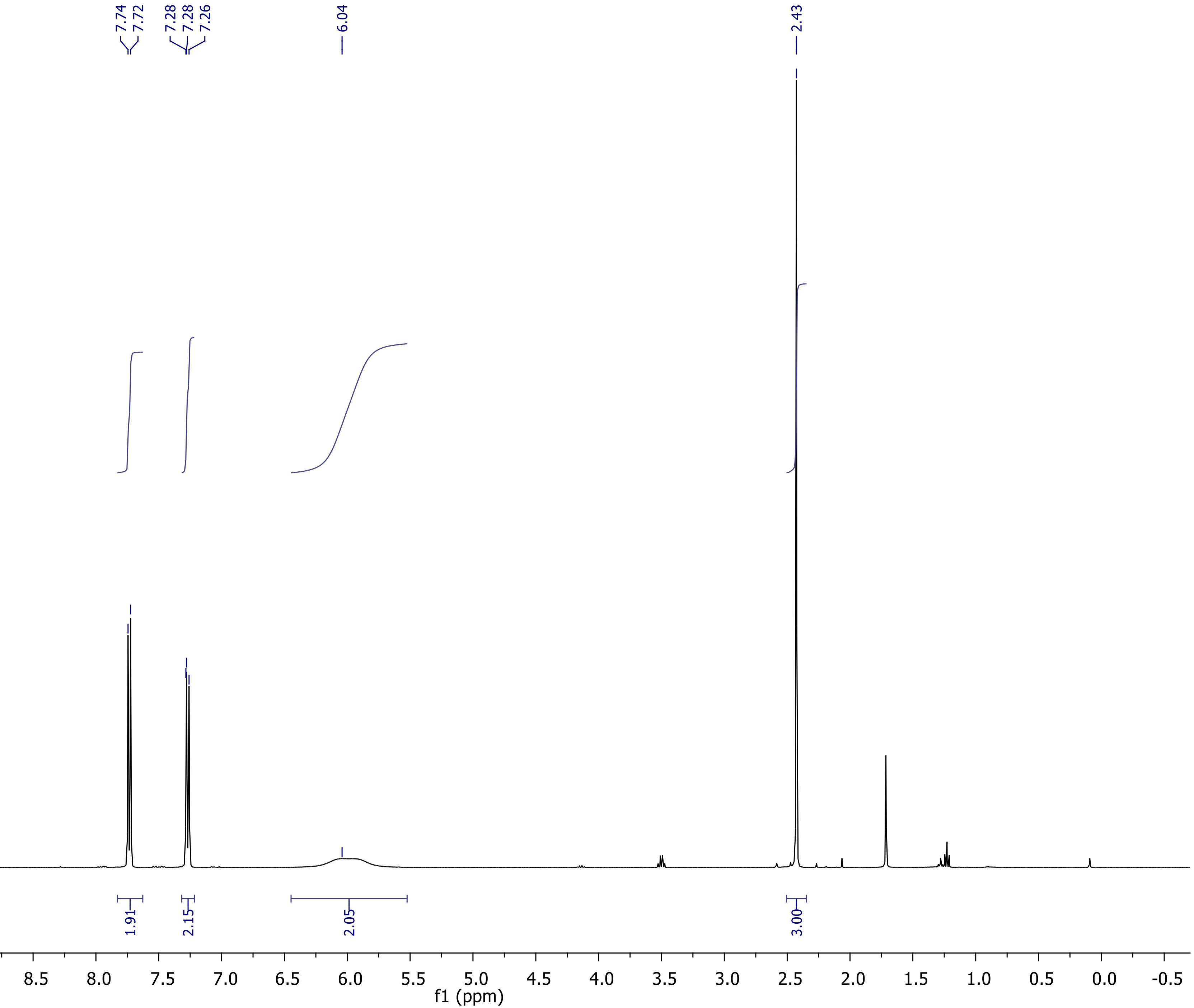

$^{13}C\{^1H\}$ NMR: 101 MHz, $CDCl_3$

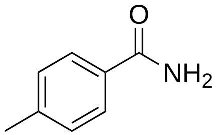

3p

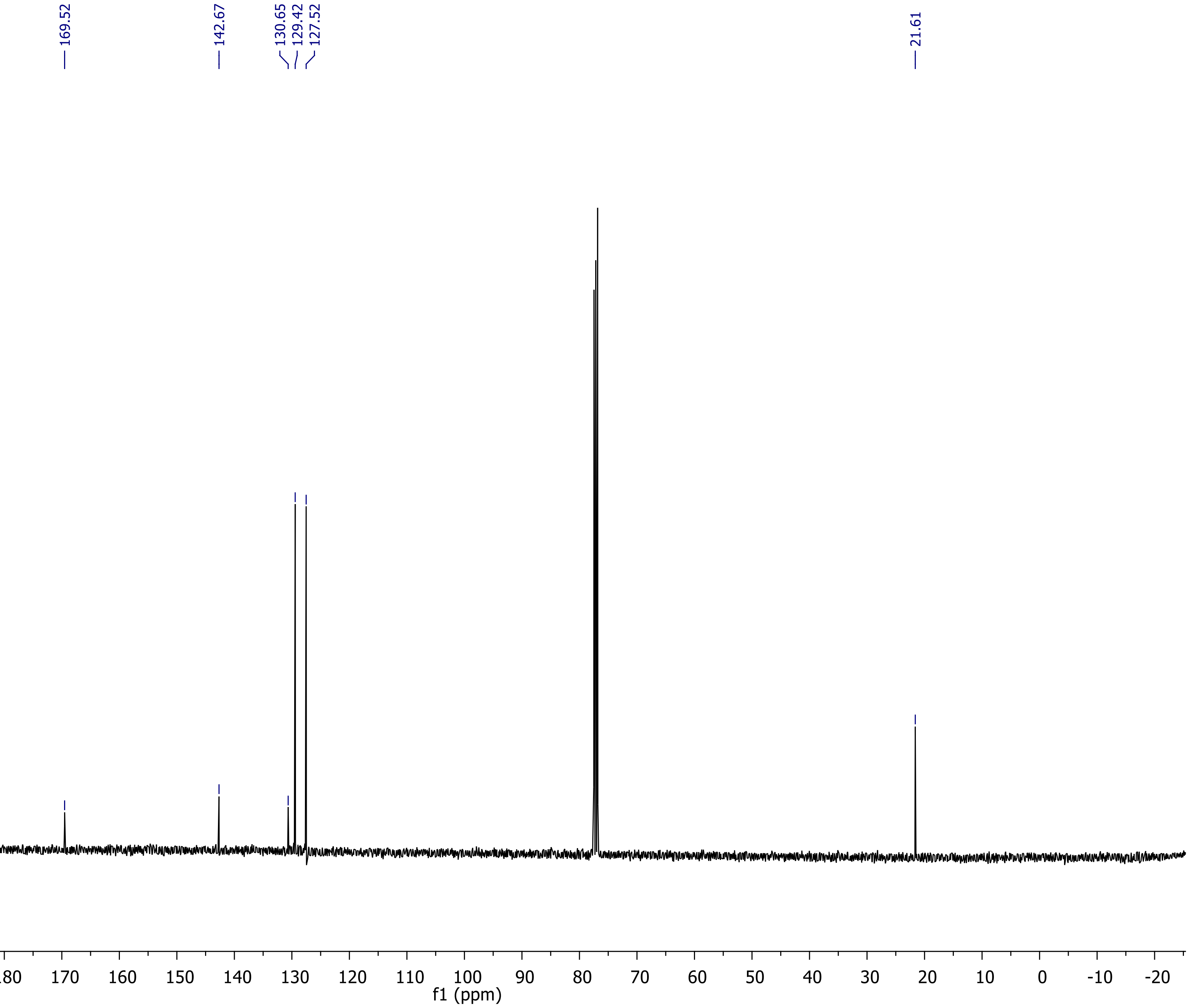

^{1}H NMR: 500 MHz, $CDCl_3$

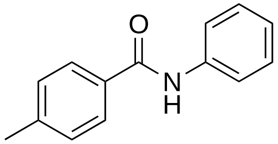

3q

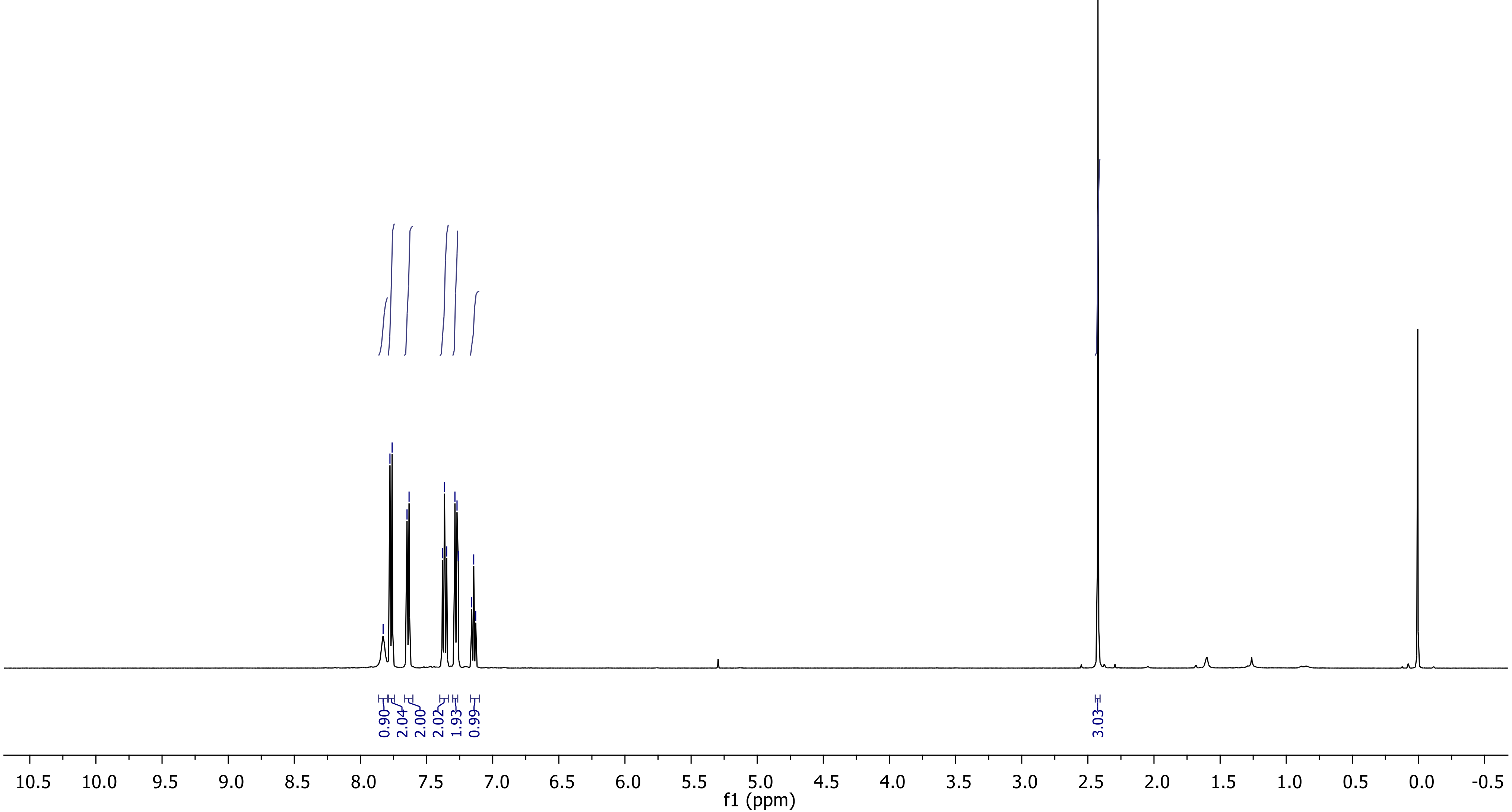

$^{13}C\{^1H\}$ NMR: 101 MHz, $CDCl_3$

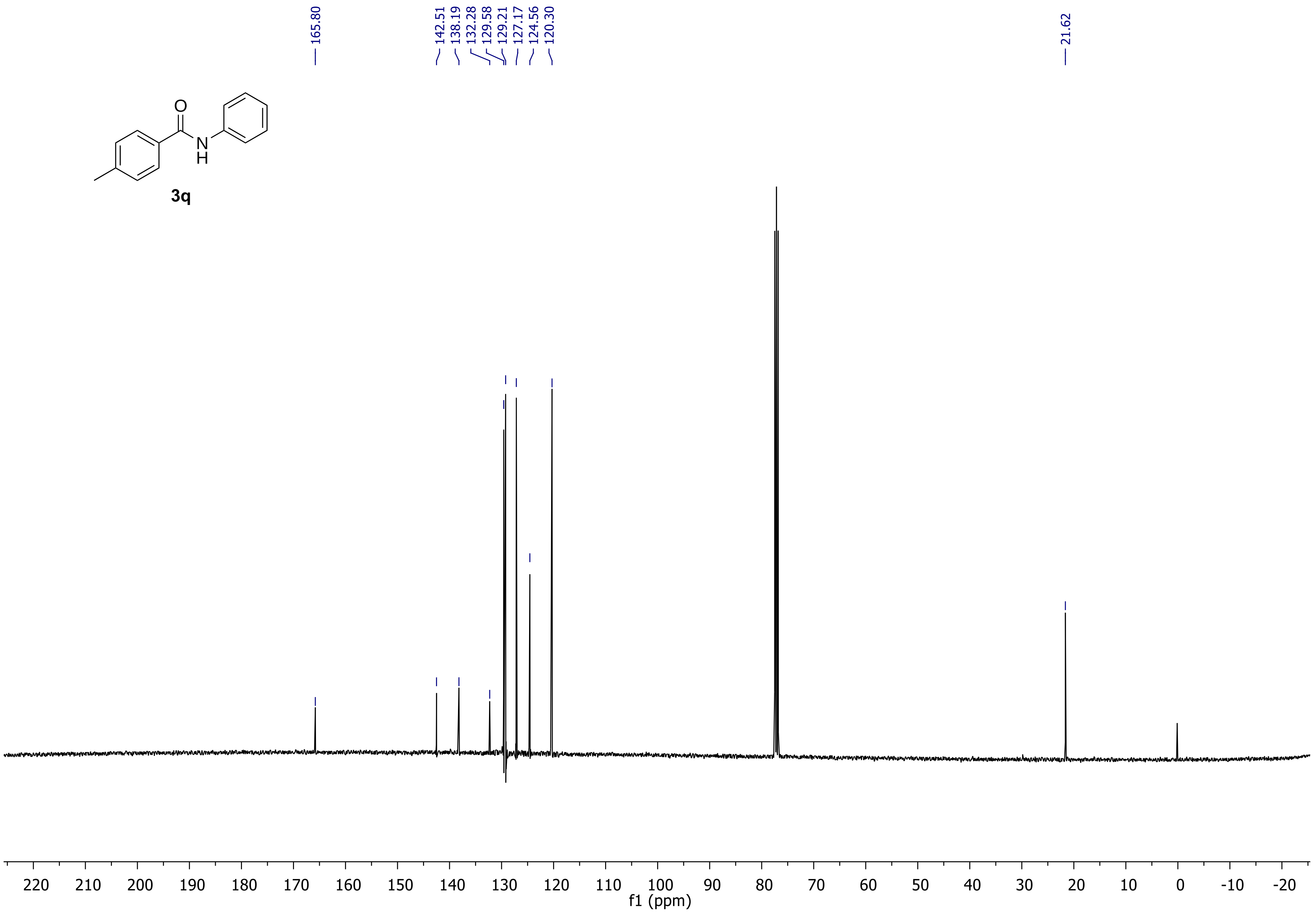

^{1}H NMR: 500 MHz, $CDCl_3$

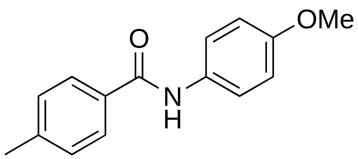

3r

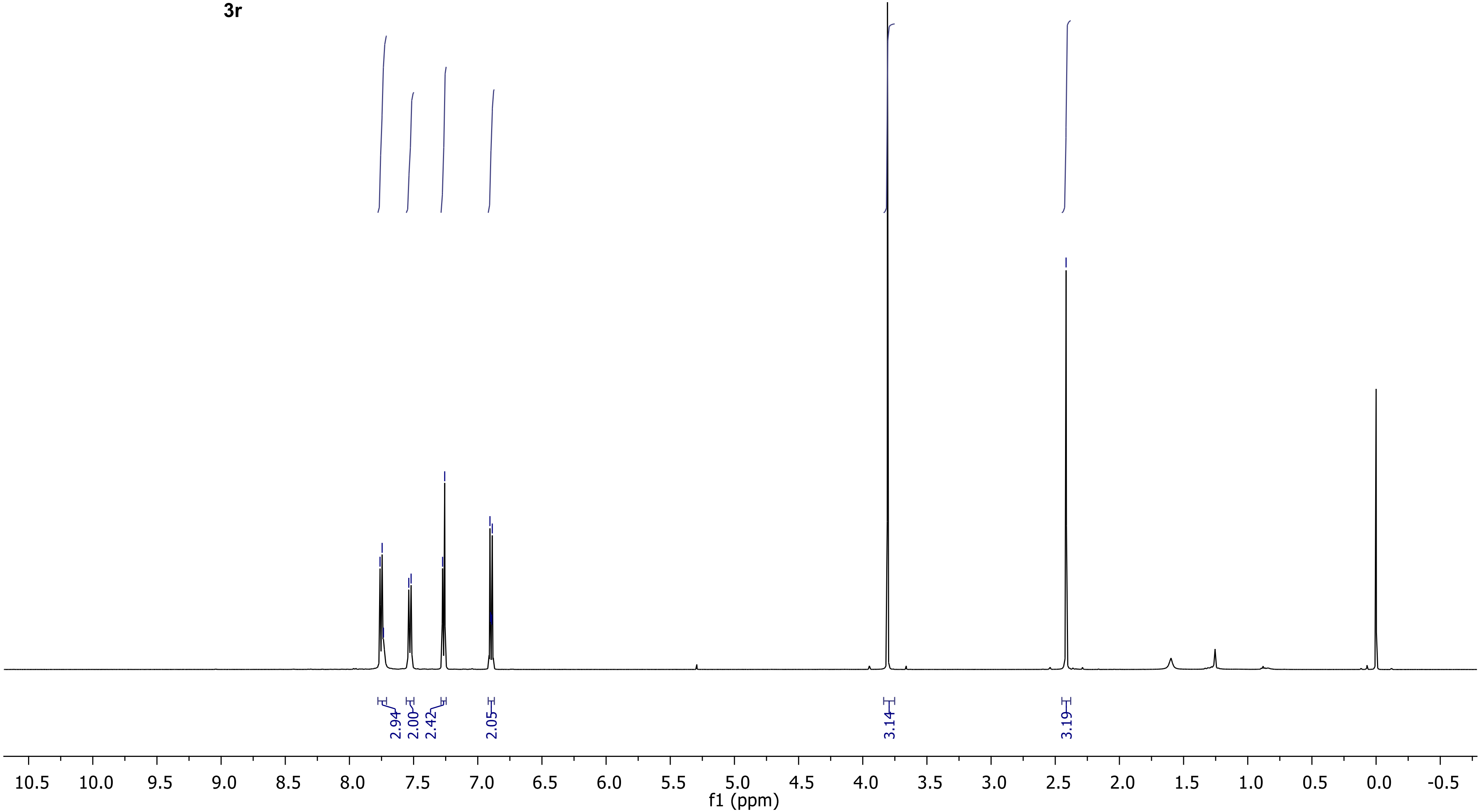

$^{13}C\{^{1}H\}$ NMR: 101 MHz, $CDCl_3$

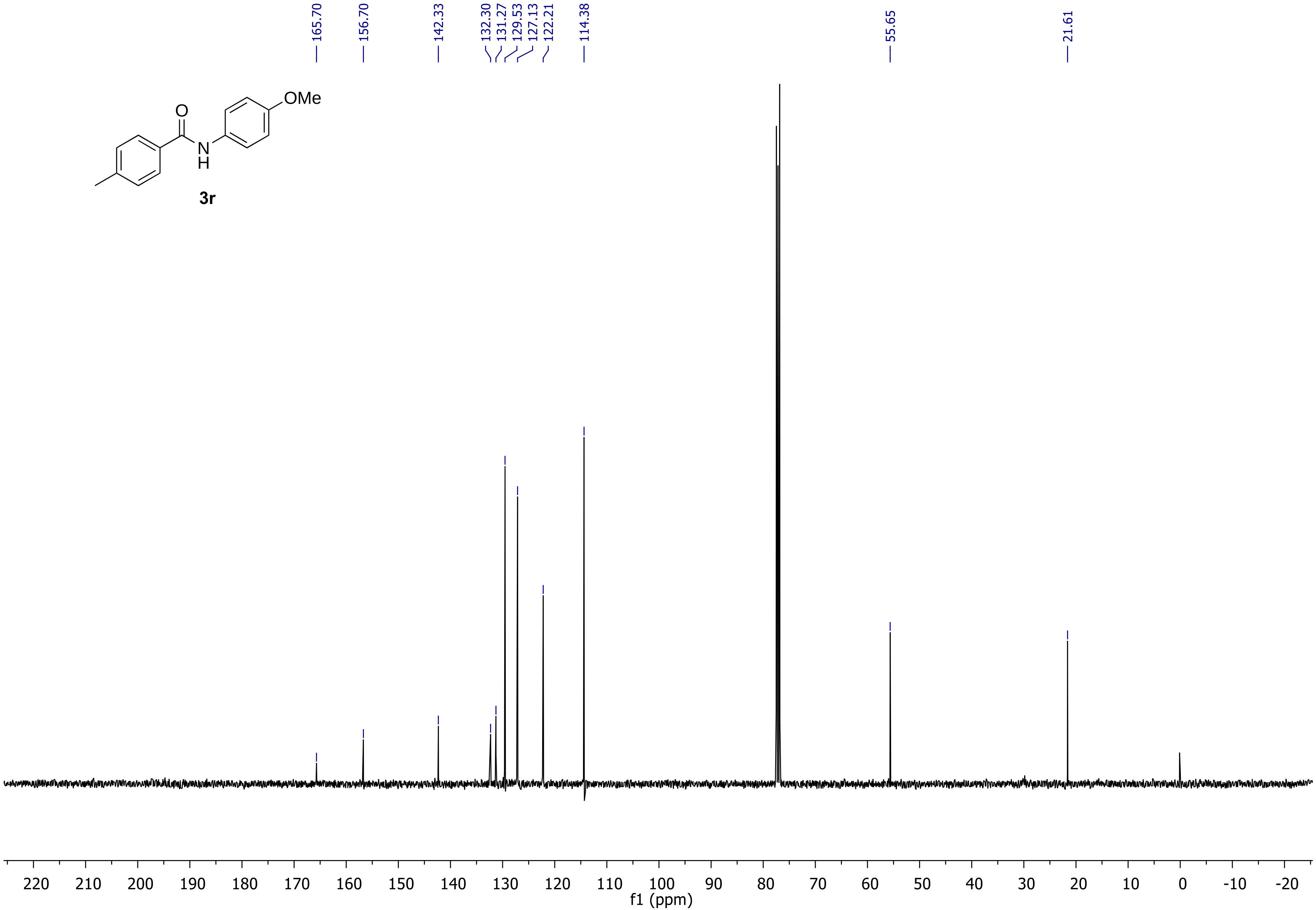

^{1}H NMR: 500 MHz, $CDCl_3$

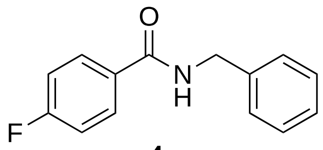

4a

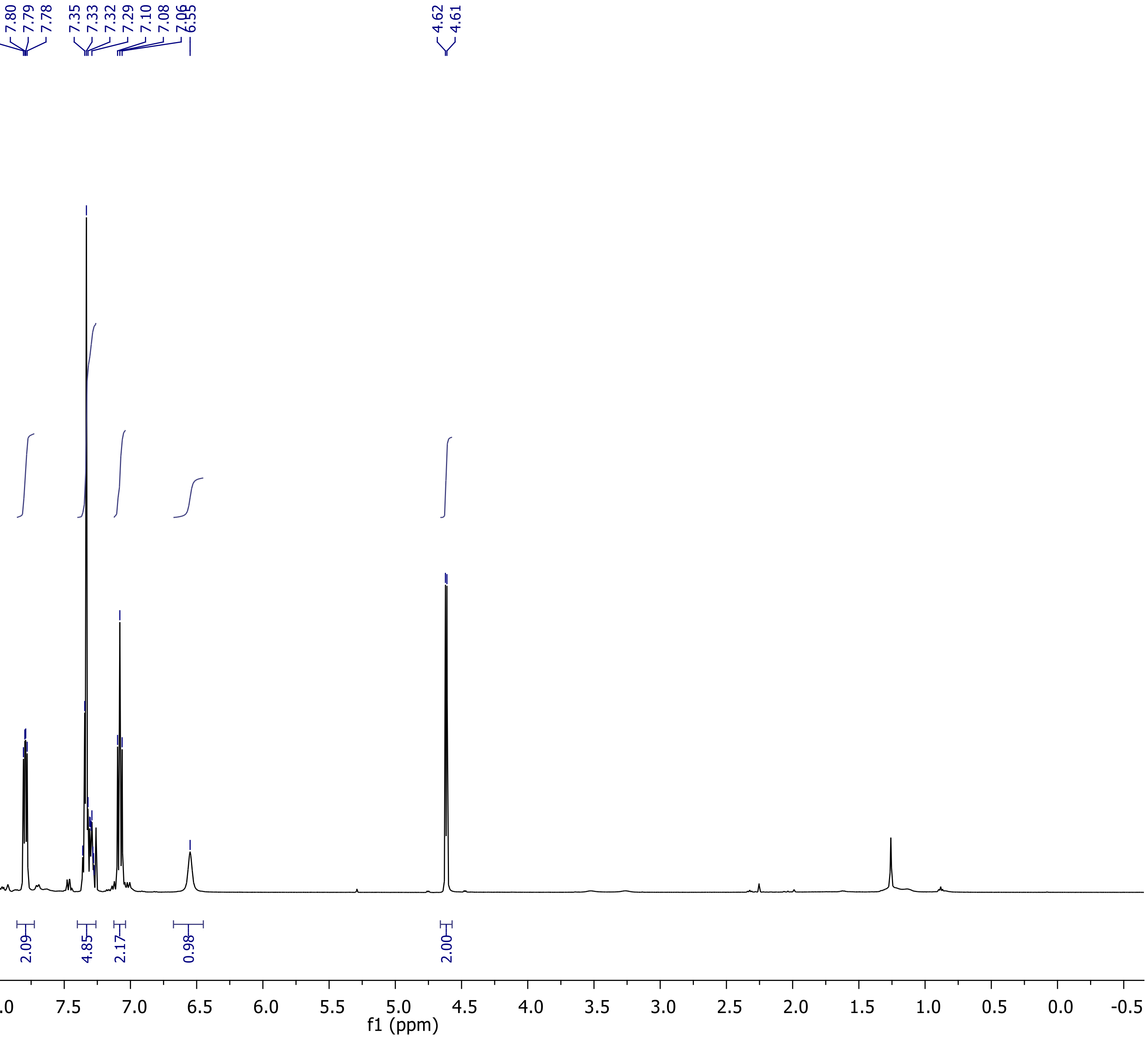

$^{13}C\{^1H\}$ NMR: 126 MHz, $CDCl_3$

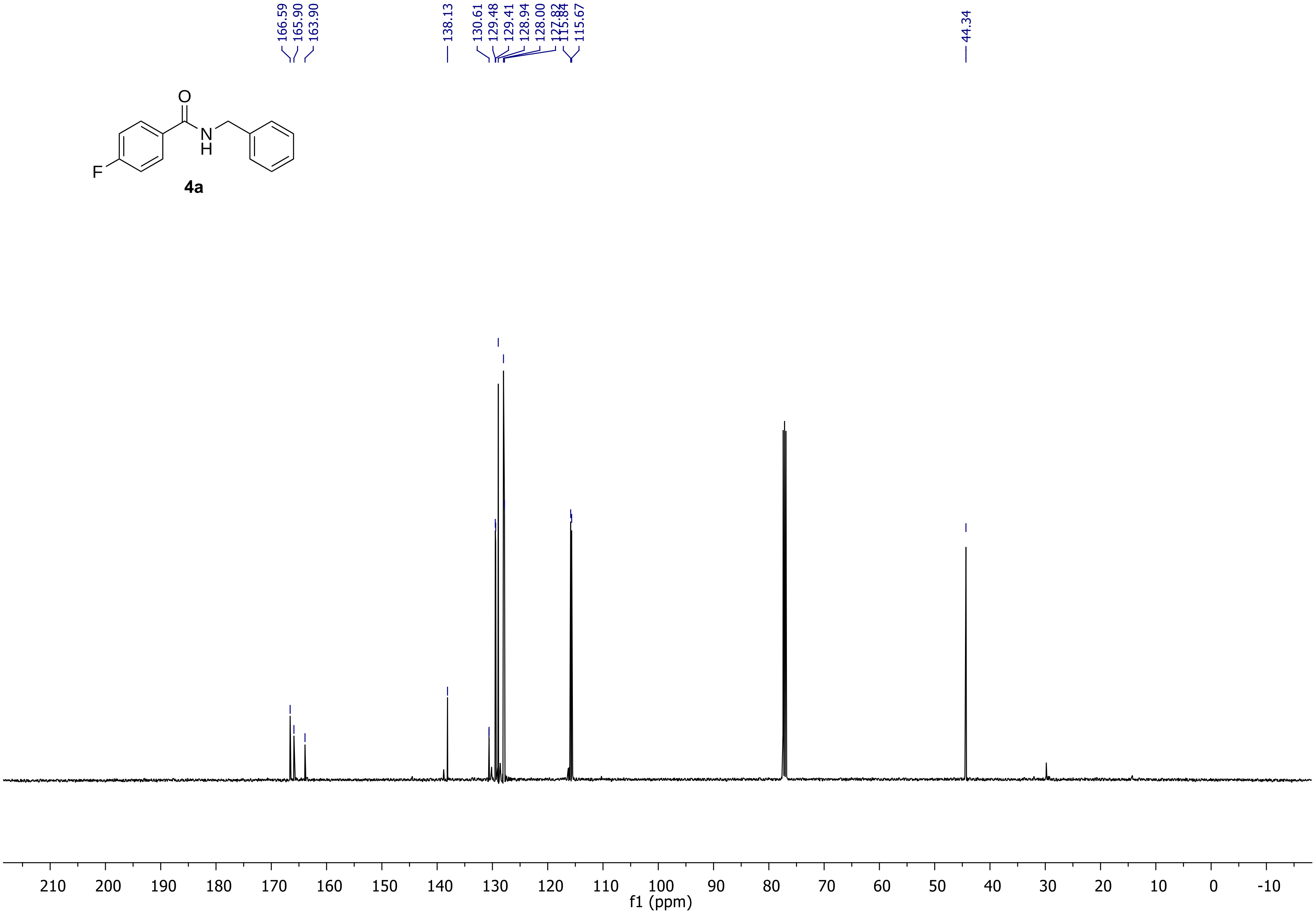

^{1}H NMR: 400 MHz, $CDCl_3$

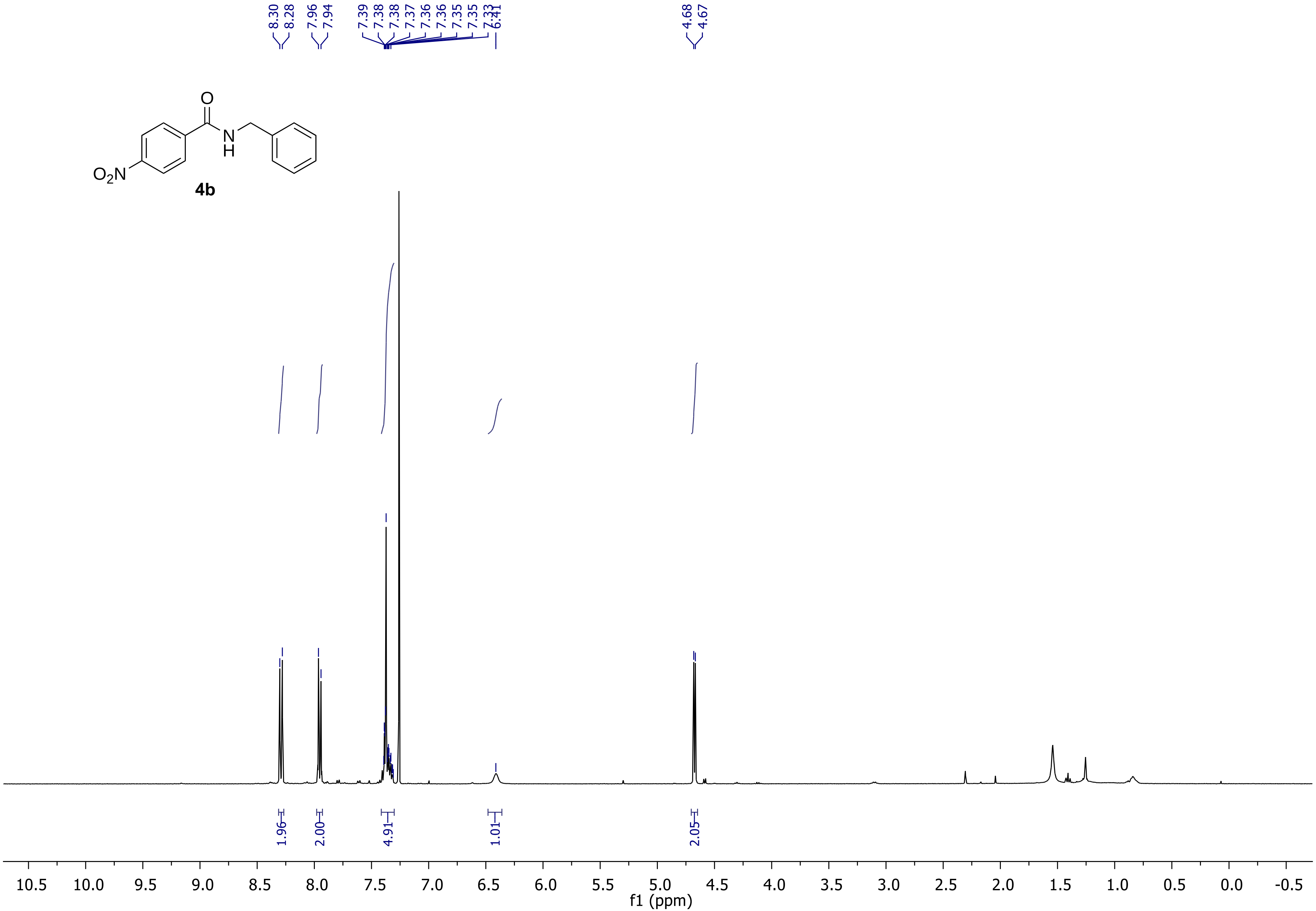

$^{13}C\{^1H\}$ NMR: 101 MHz, $CDCl_3$

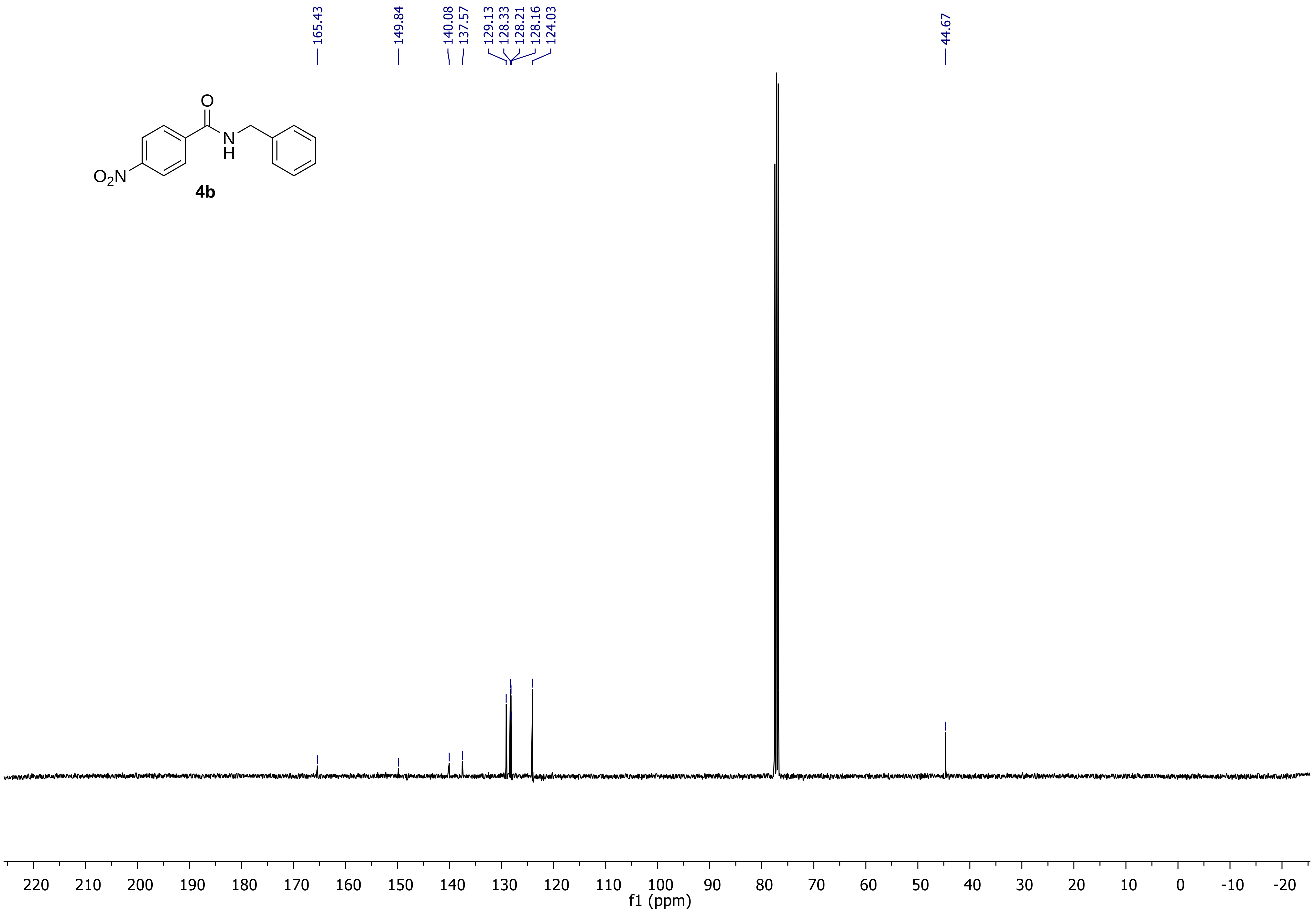

4b

^{1}H NMR: 500 MHz, $CDCl_3$

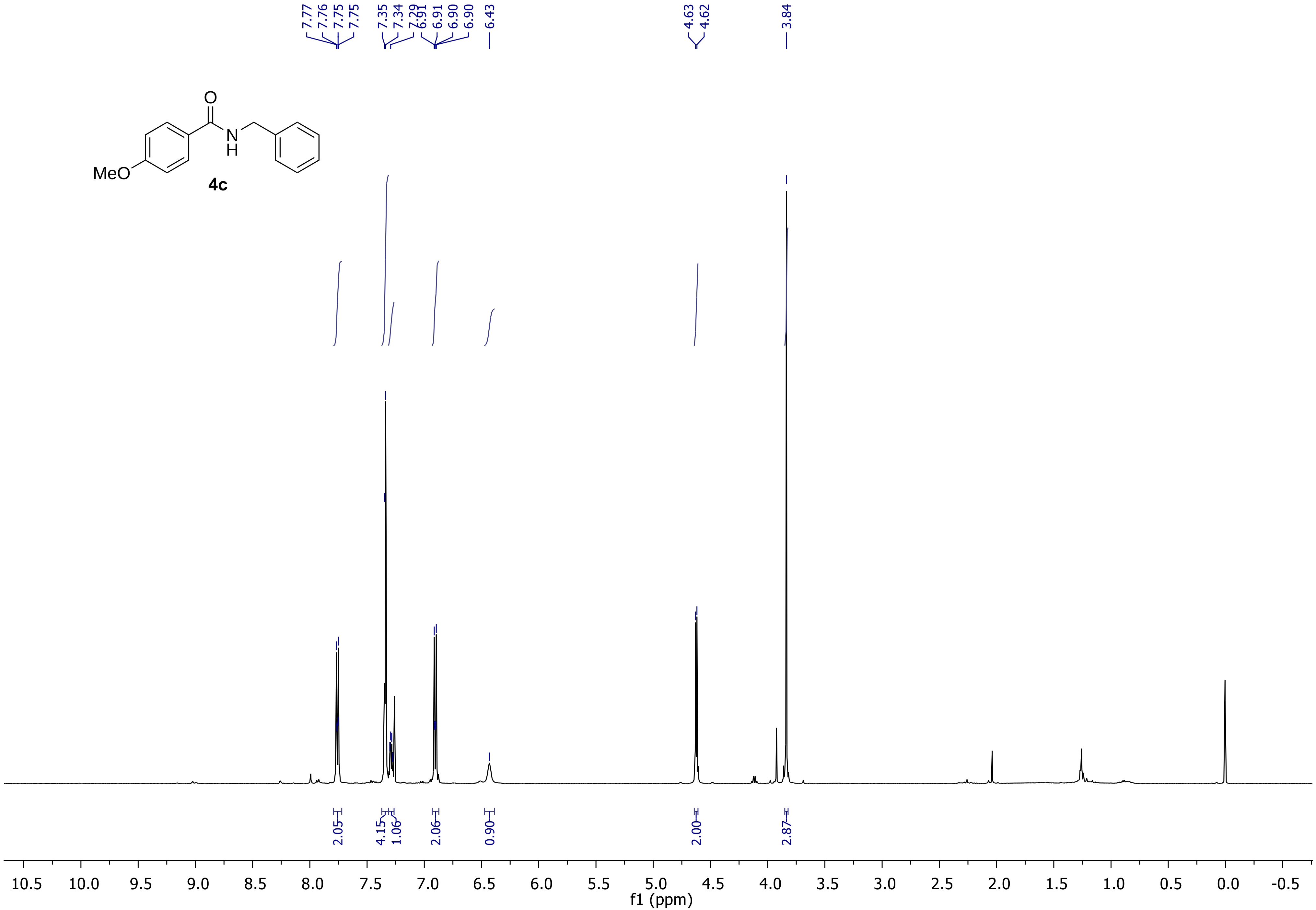

$^{13}C\{^{1}H\}$ NMR: 101 MHz, $CDCl_3$

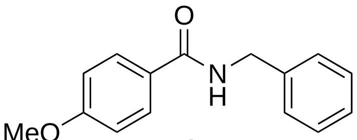

4c

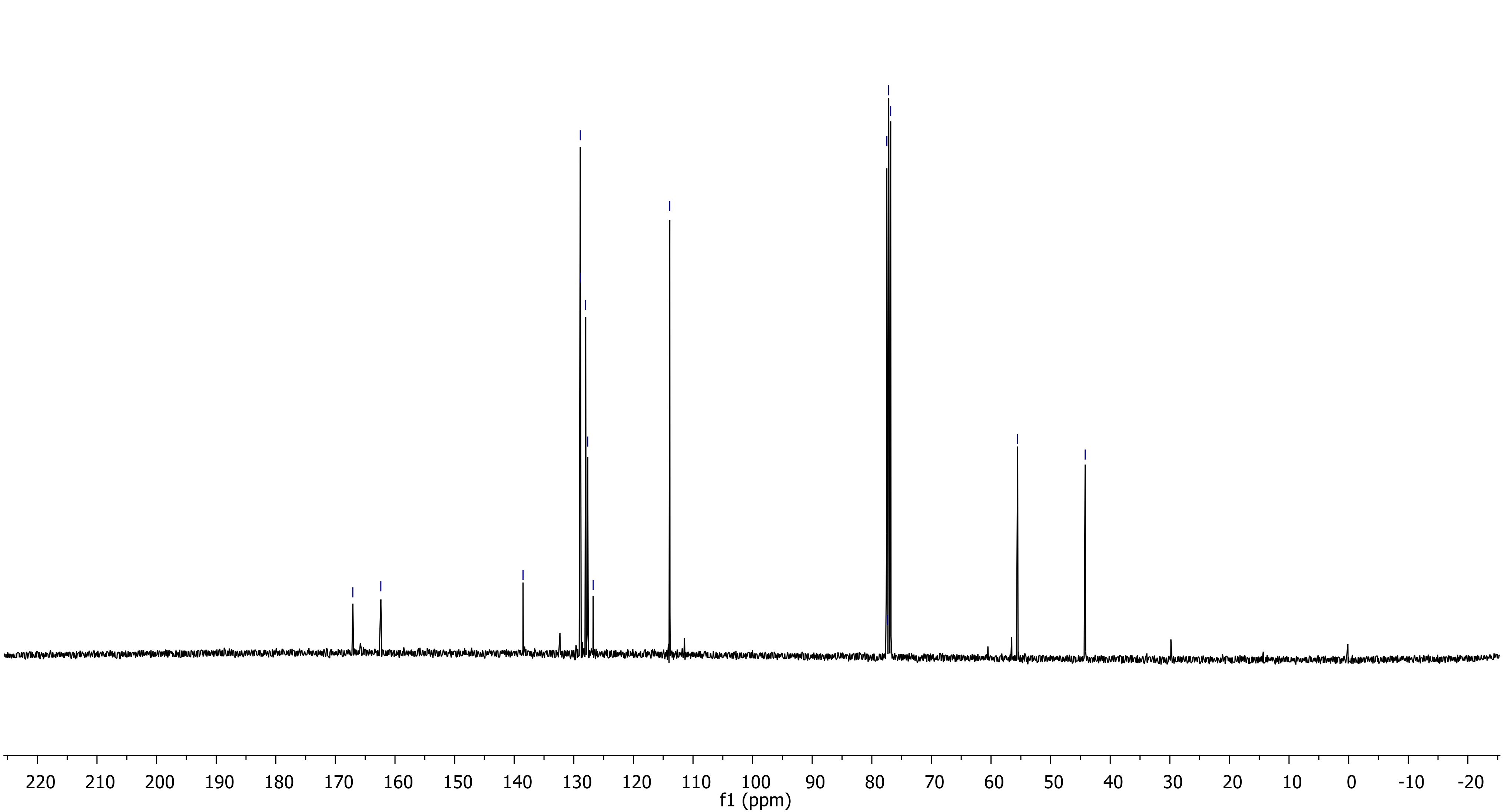

^{1}H NMR: 500 MHz, D6-DMSO

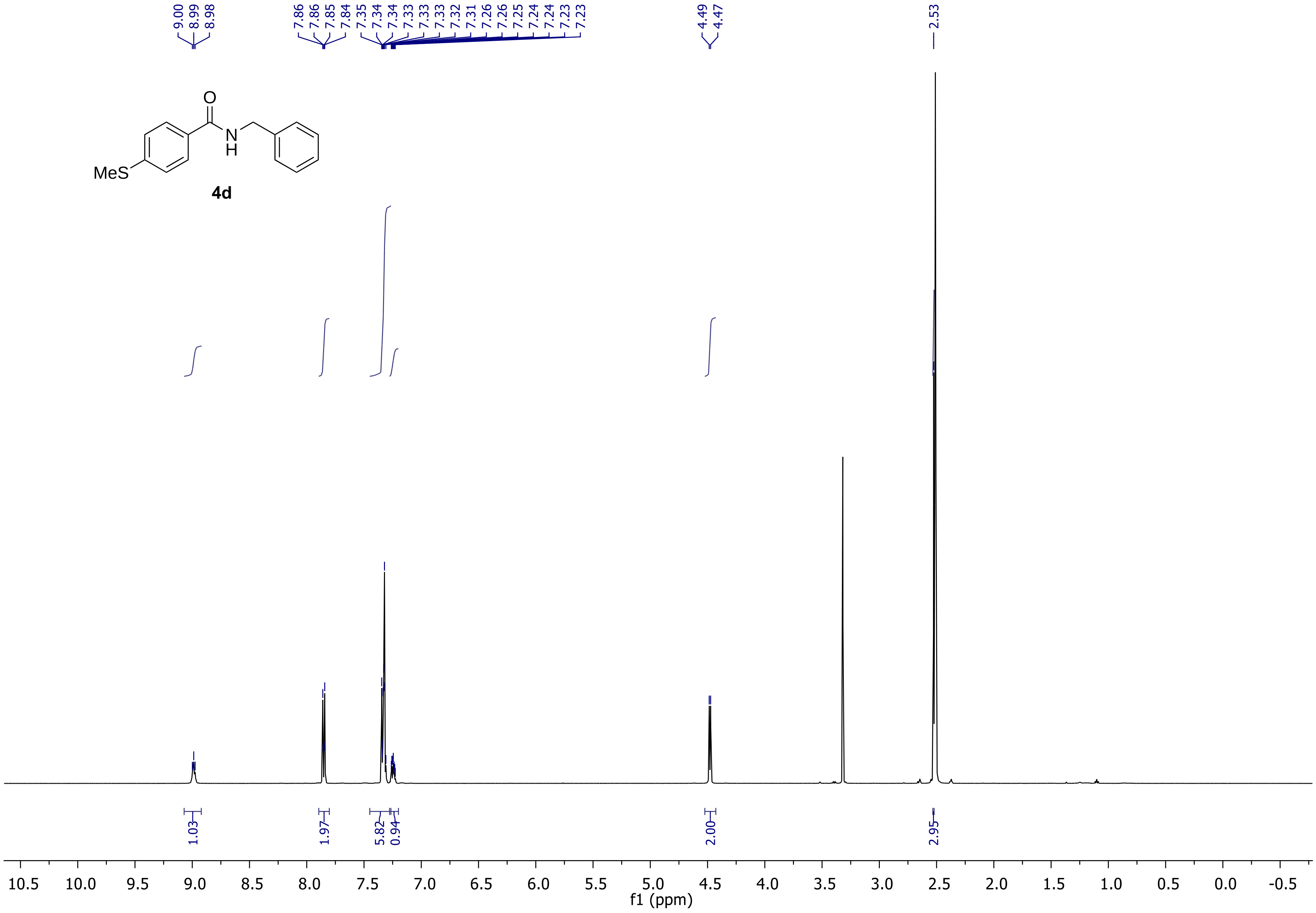

$^{13}C\{^{1}H\}$ NMR: 126 MHz, D_6-DMSO

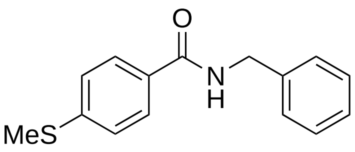

4d

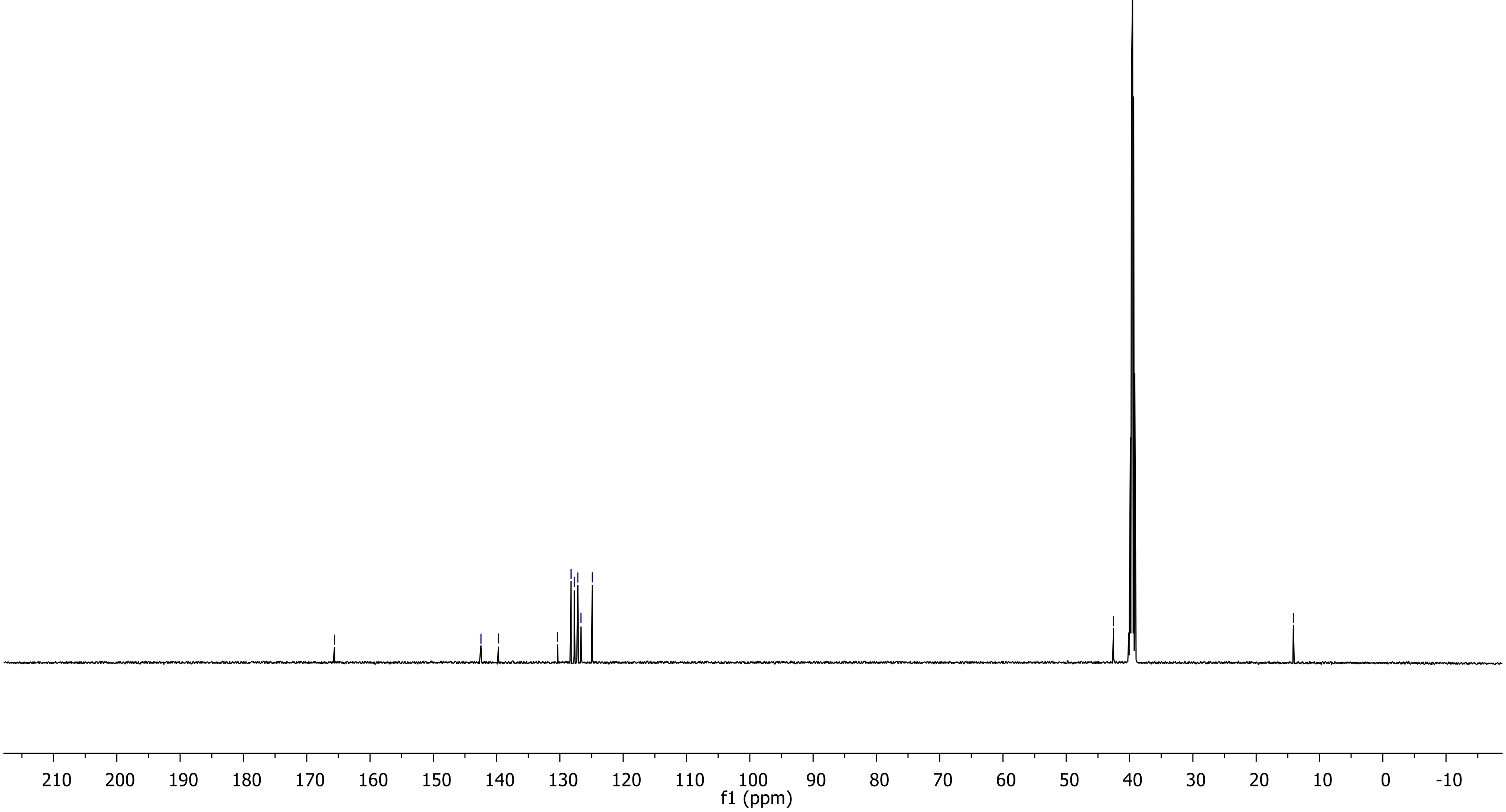

^{1}H NMR: 500 MHz, $CDCl_3$

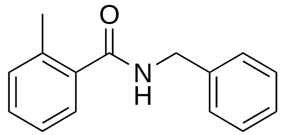

4e

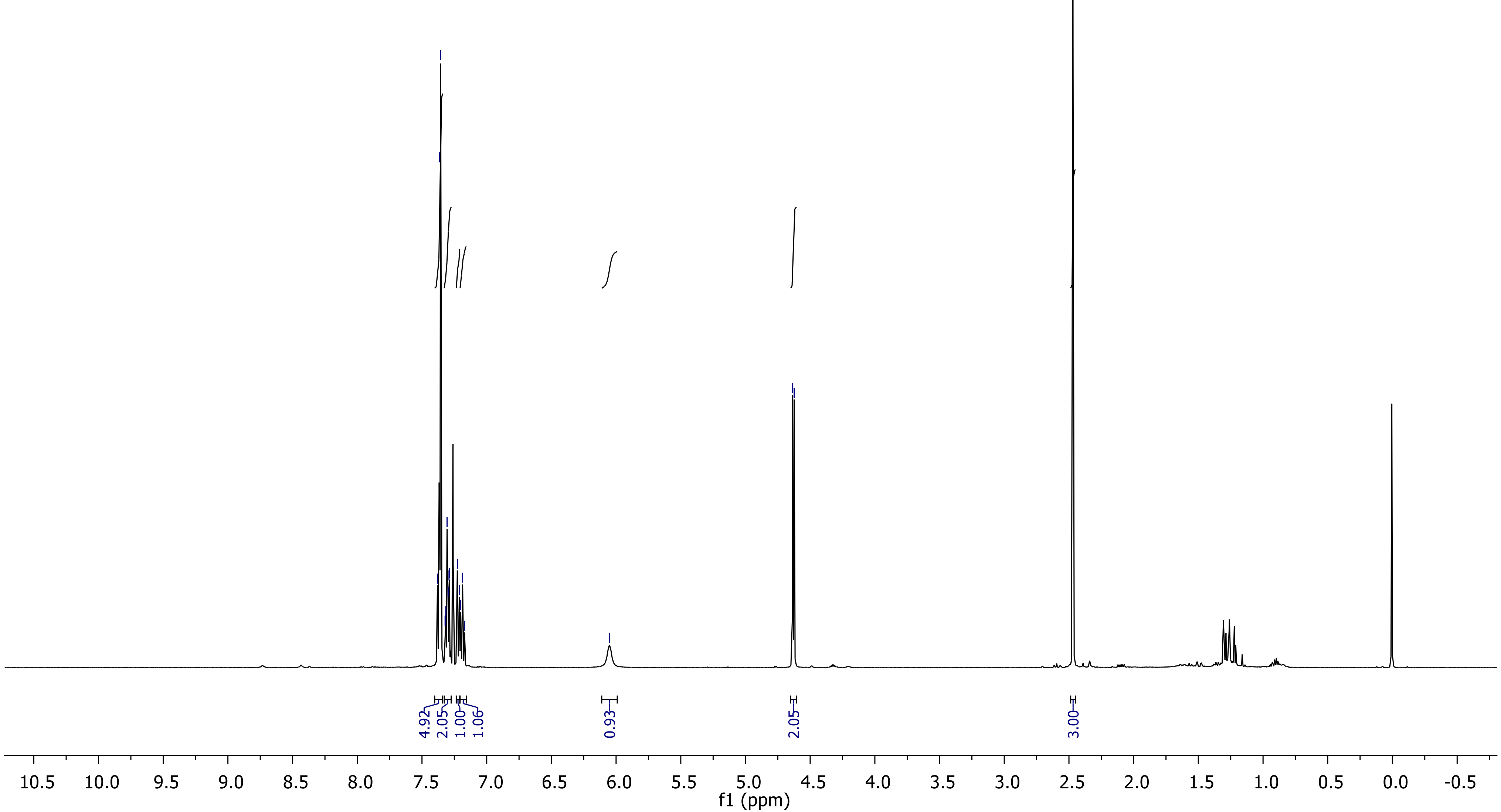

$^{13}C\{^{1}H\}$ NMR: 101 MHz, $CDCl_3$

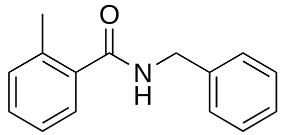

4e

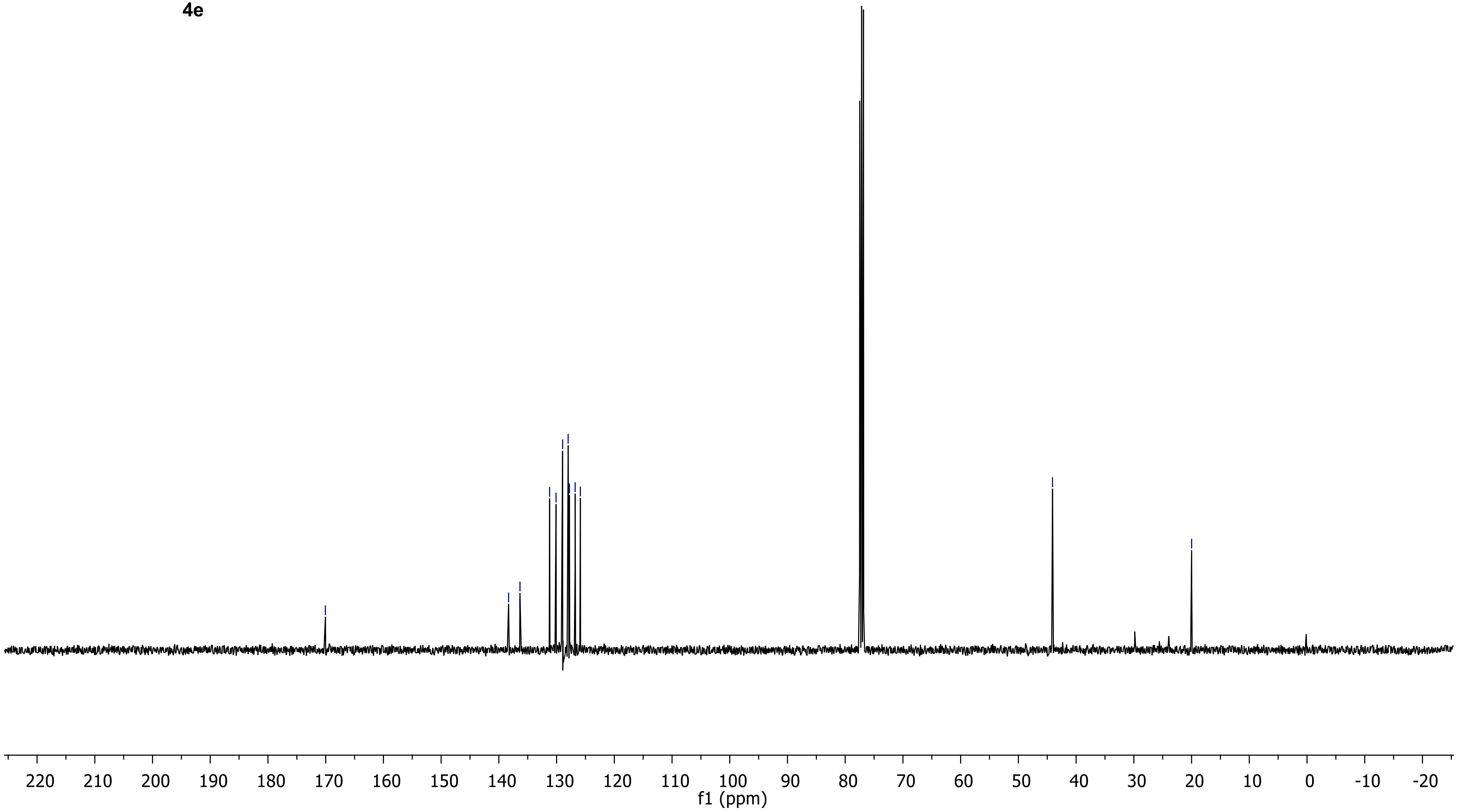

^{1}H NMR: 500 MHz, $CDCl_3$

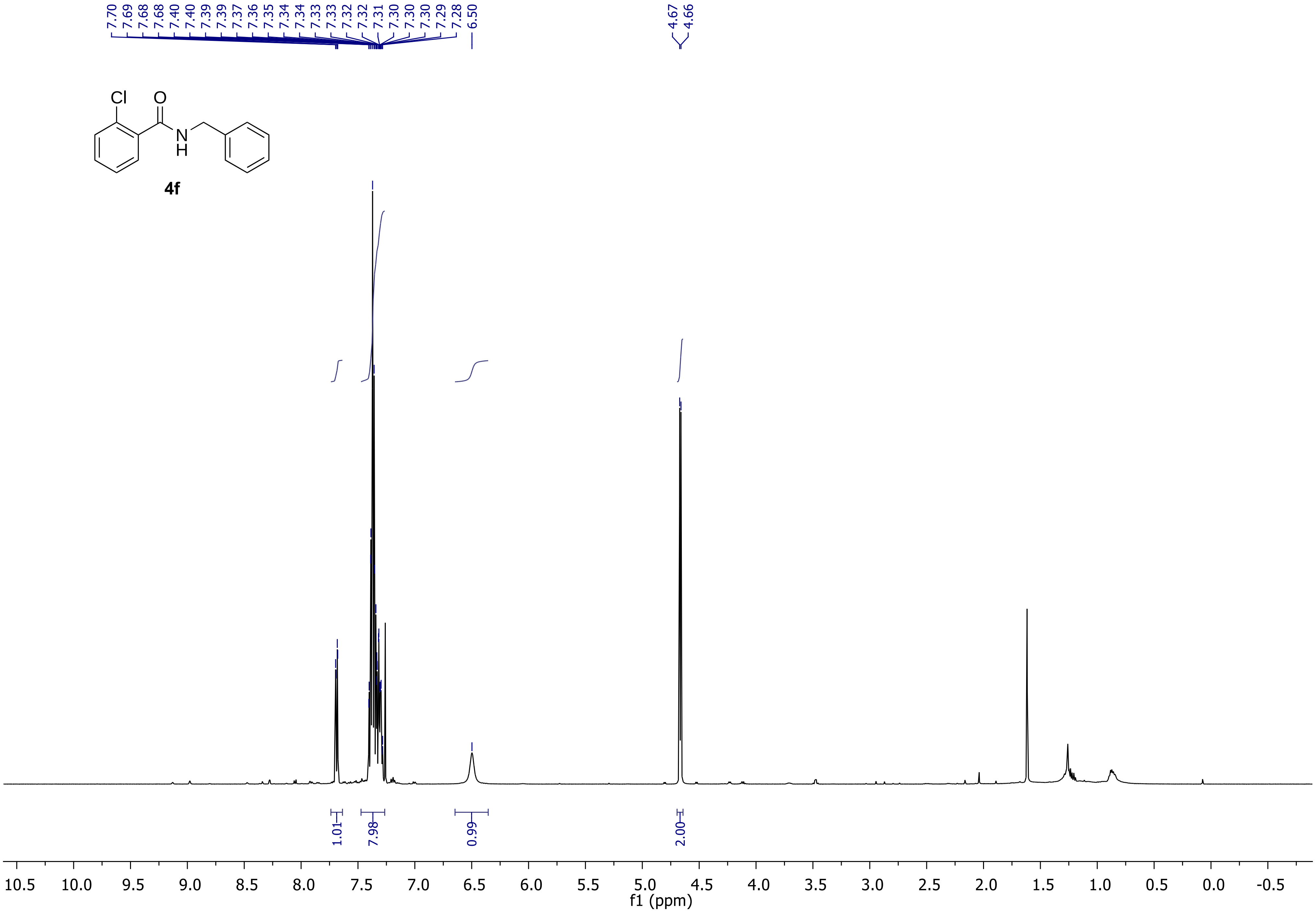

$^{13}C\{^{1}H\}$ NMR: 126 MHz, $CDCl_3$

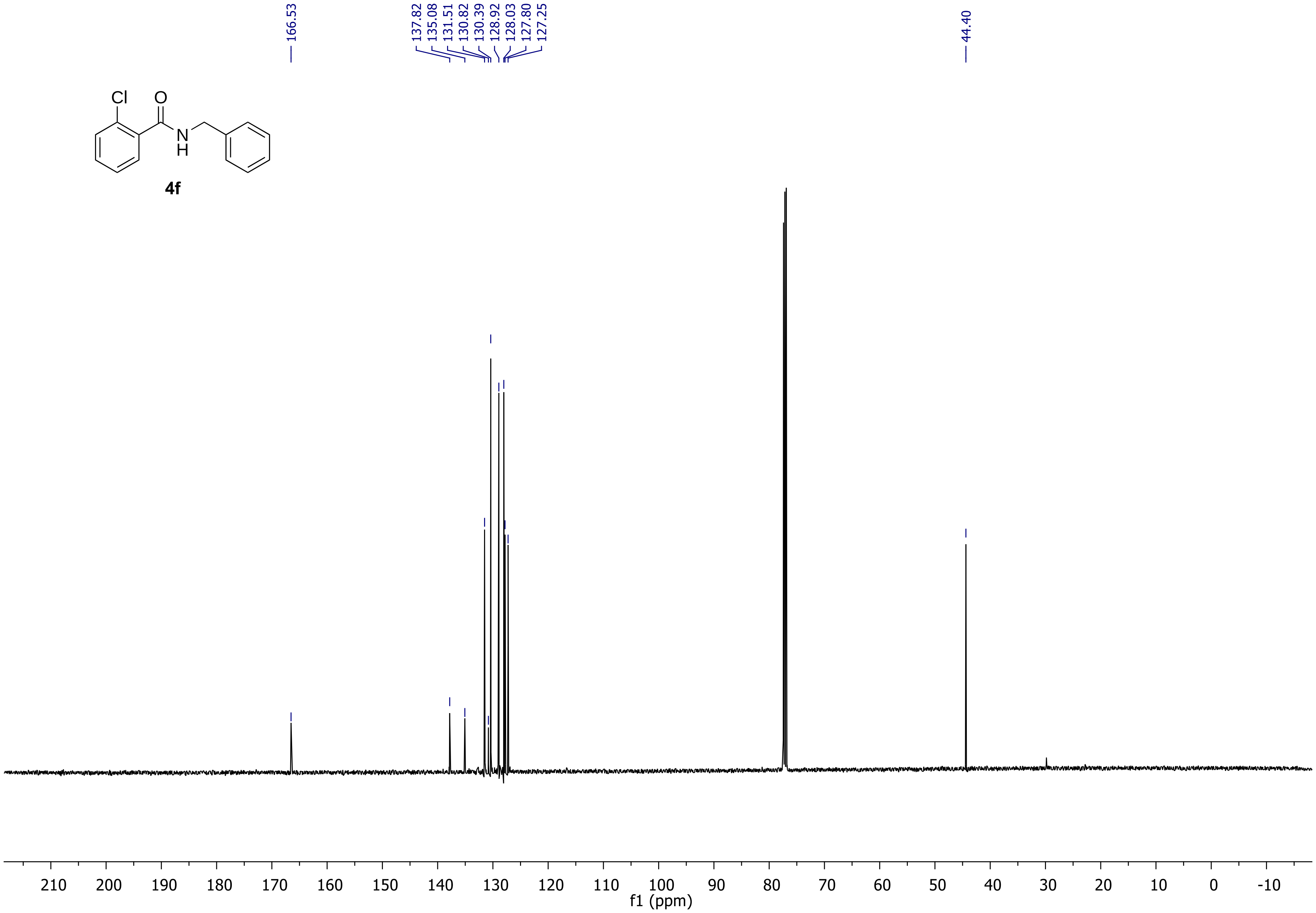

^{1}H NMR: 500 MHz, $CDCl_3$

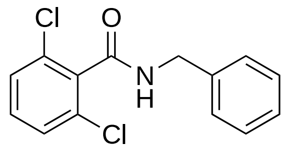

4g

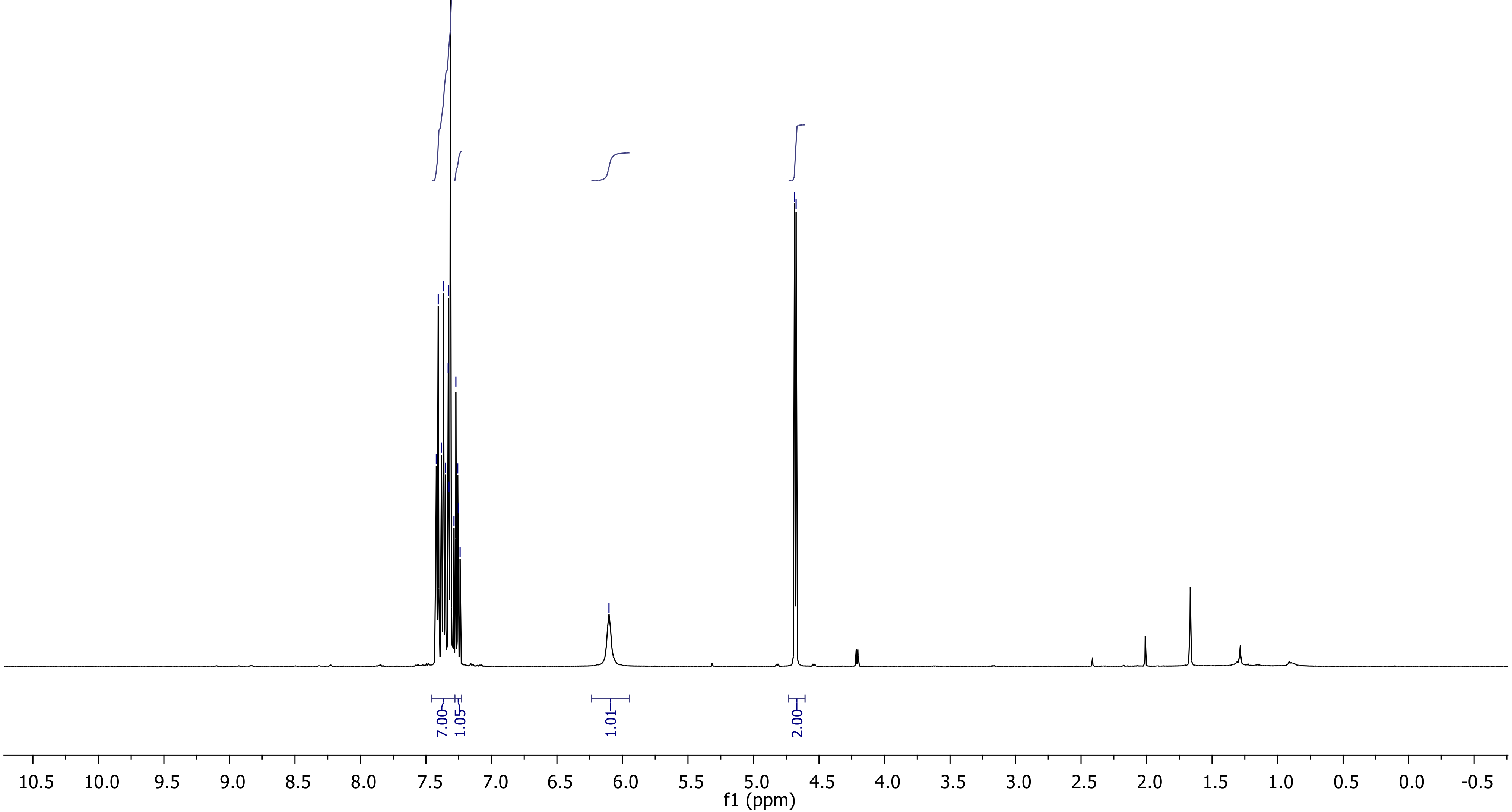

$^{13}C\{^{1}H\}$ NMR: 126 MHz, $CDCl_3$

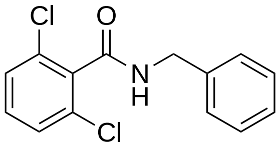

4g

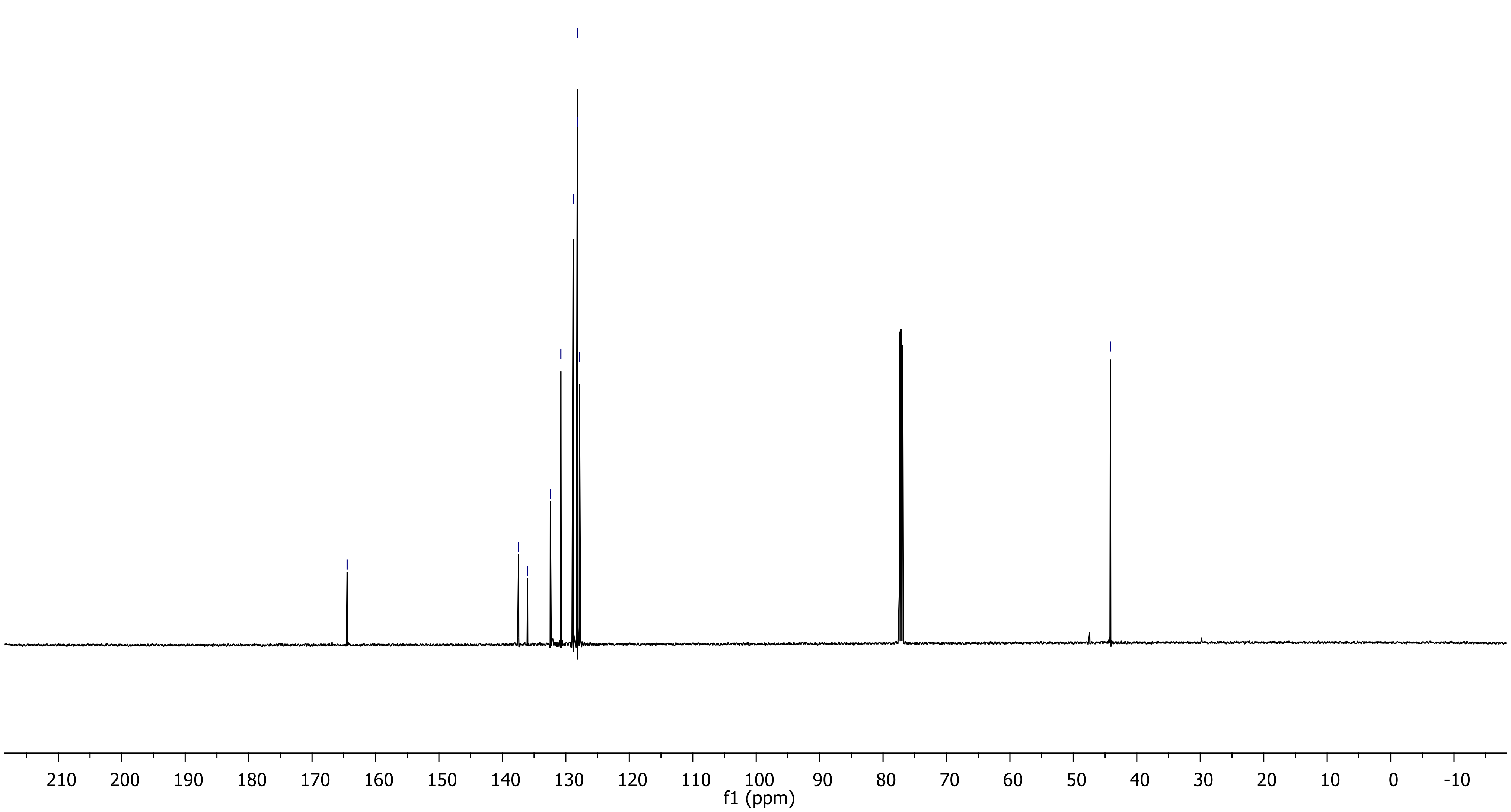

^{1}H NMR: 500 MHz, D_6-DMSO

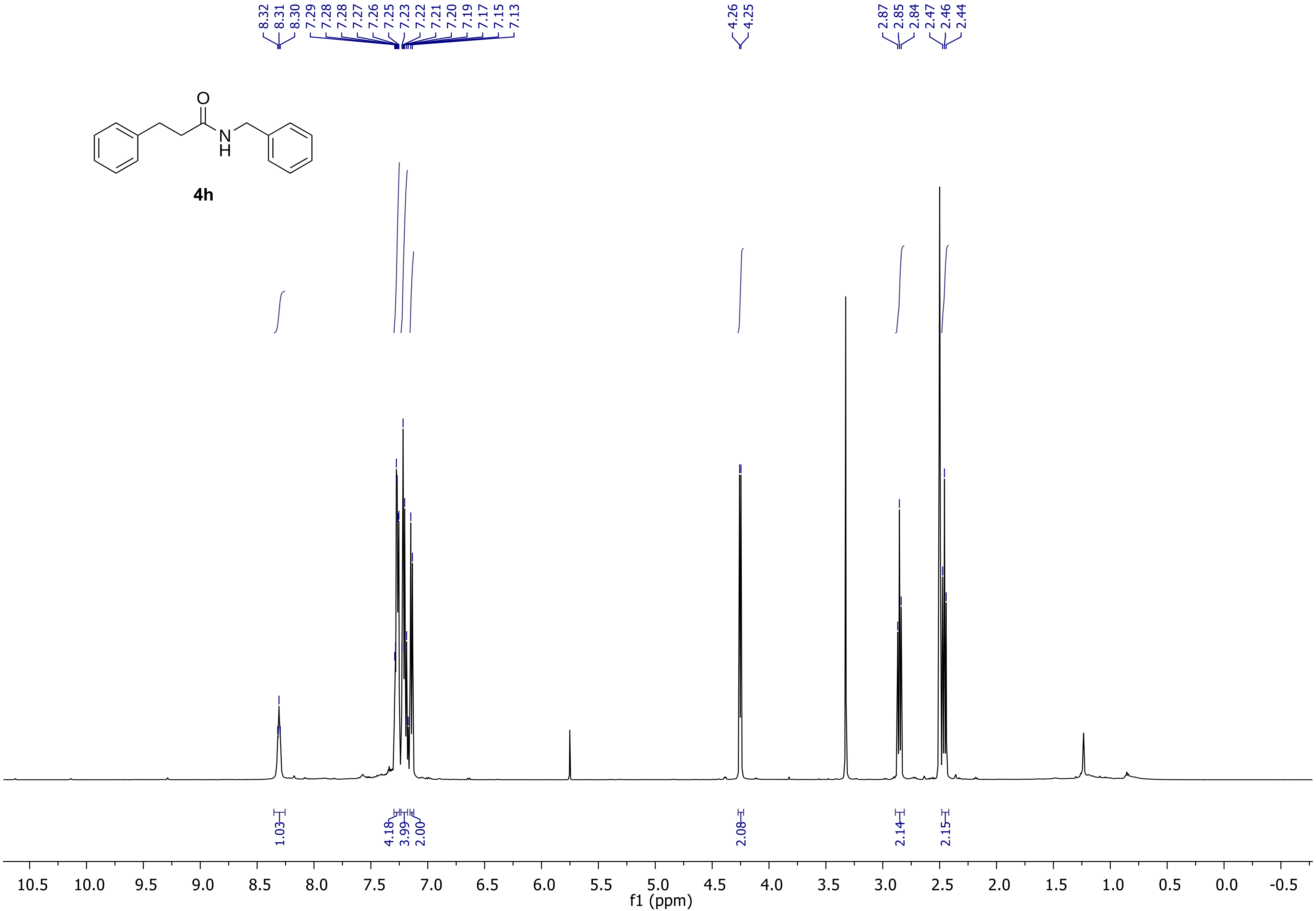

$^{13}C\{^{1}H\}$ NMR: 101 MHz, D_6-DMSO

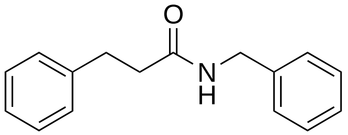

4h

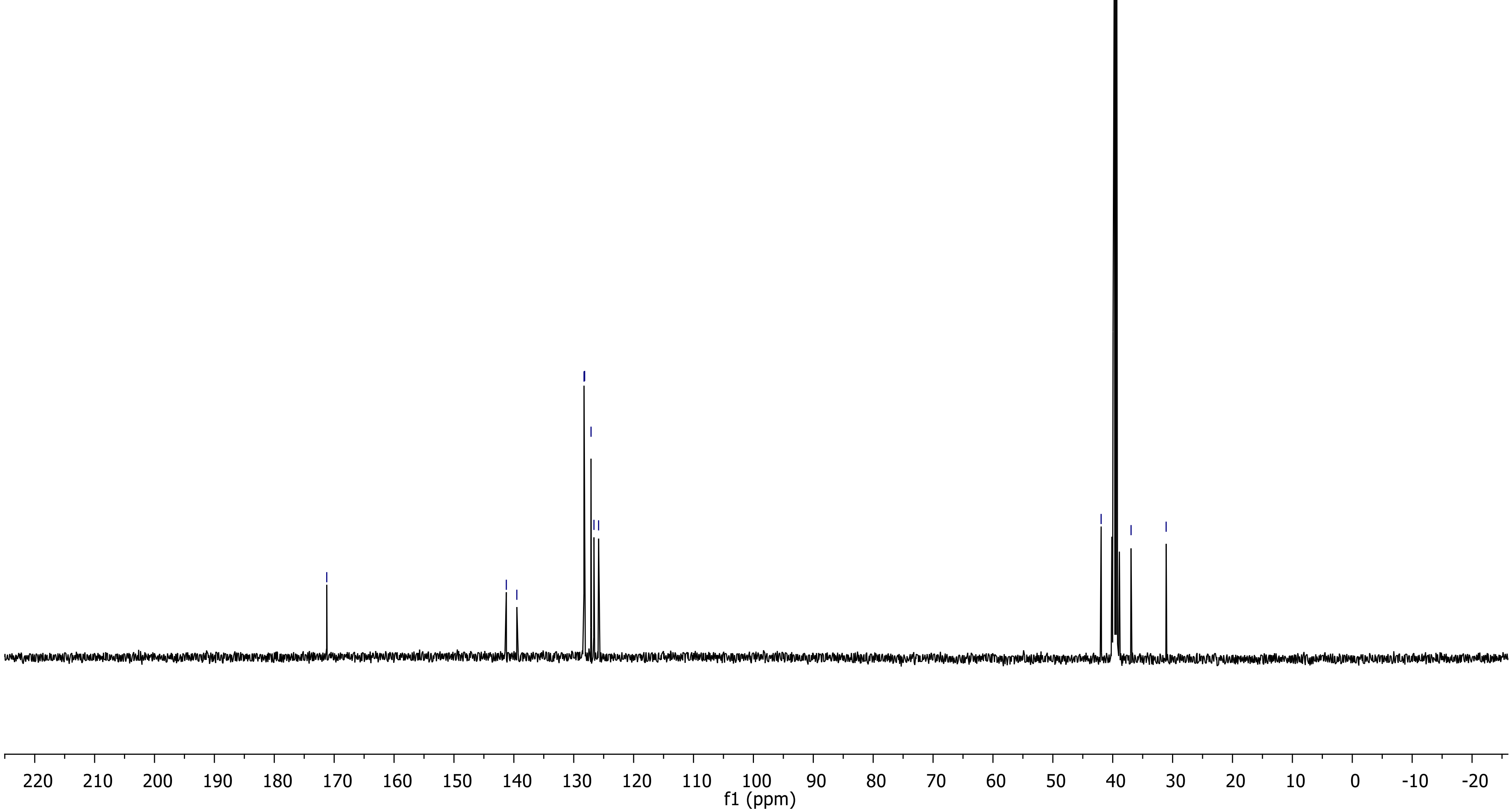

^{1}H NMR: 400 MHz, D_6-DMSO

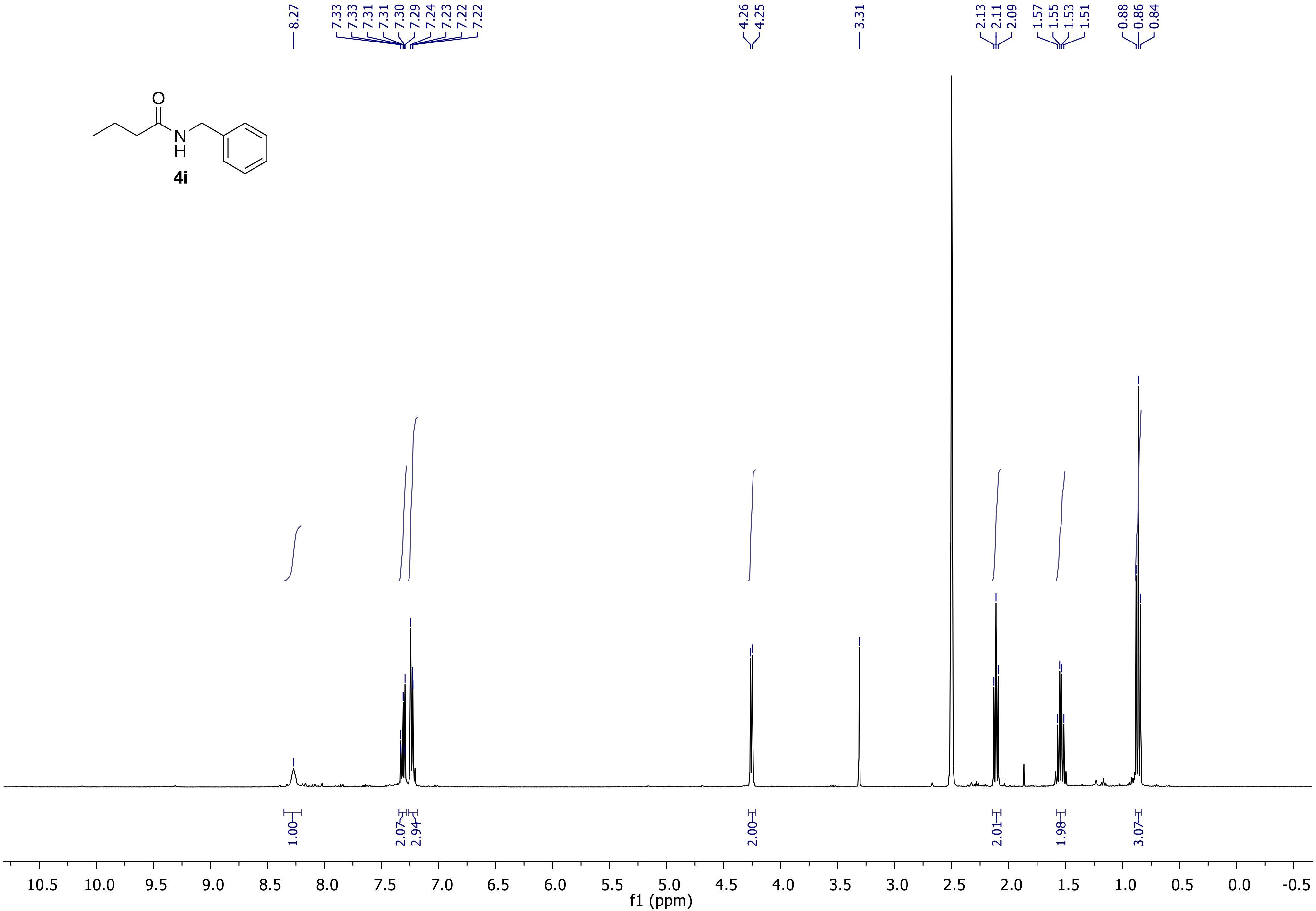

$^{13}C\{^{1}H\}$ NMR: 126 MHz, D_6-DMSO

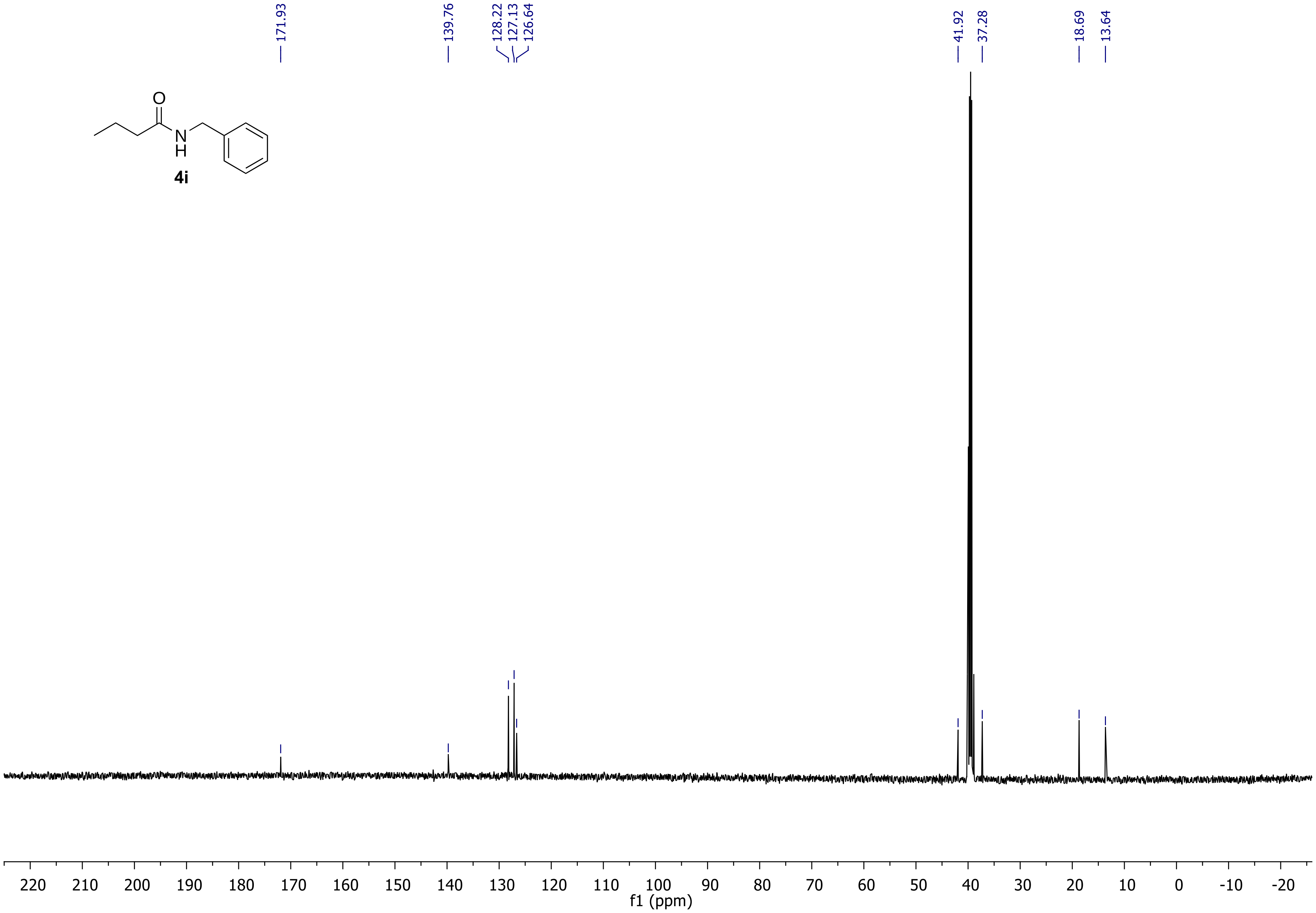

4i

^{1}H NMR: 500 MHz, $CDCl_3$

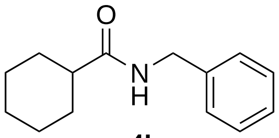

4j

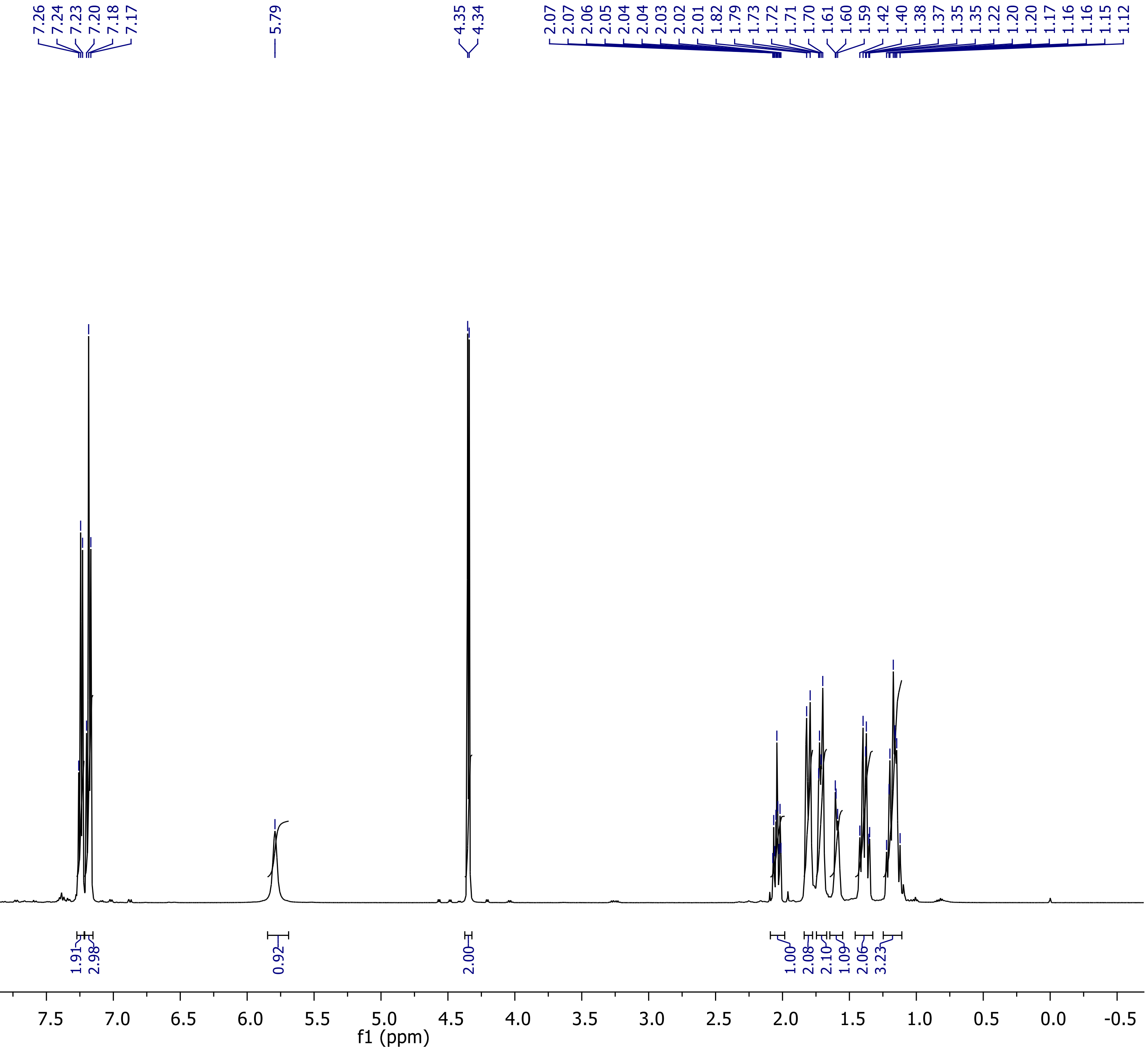

$^{13}C\{^{1}H\}$ NMR: 126 MHz, $CDCl_3$

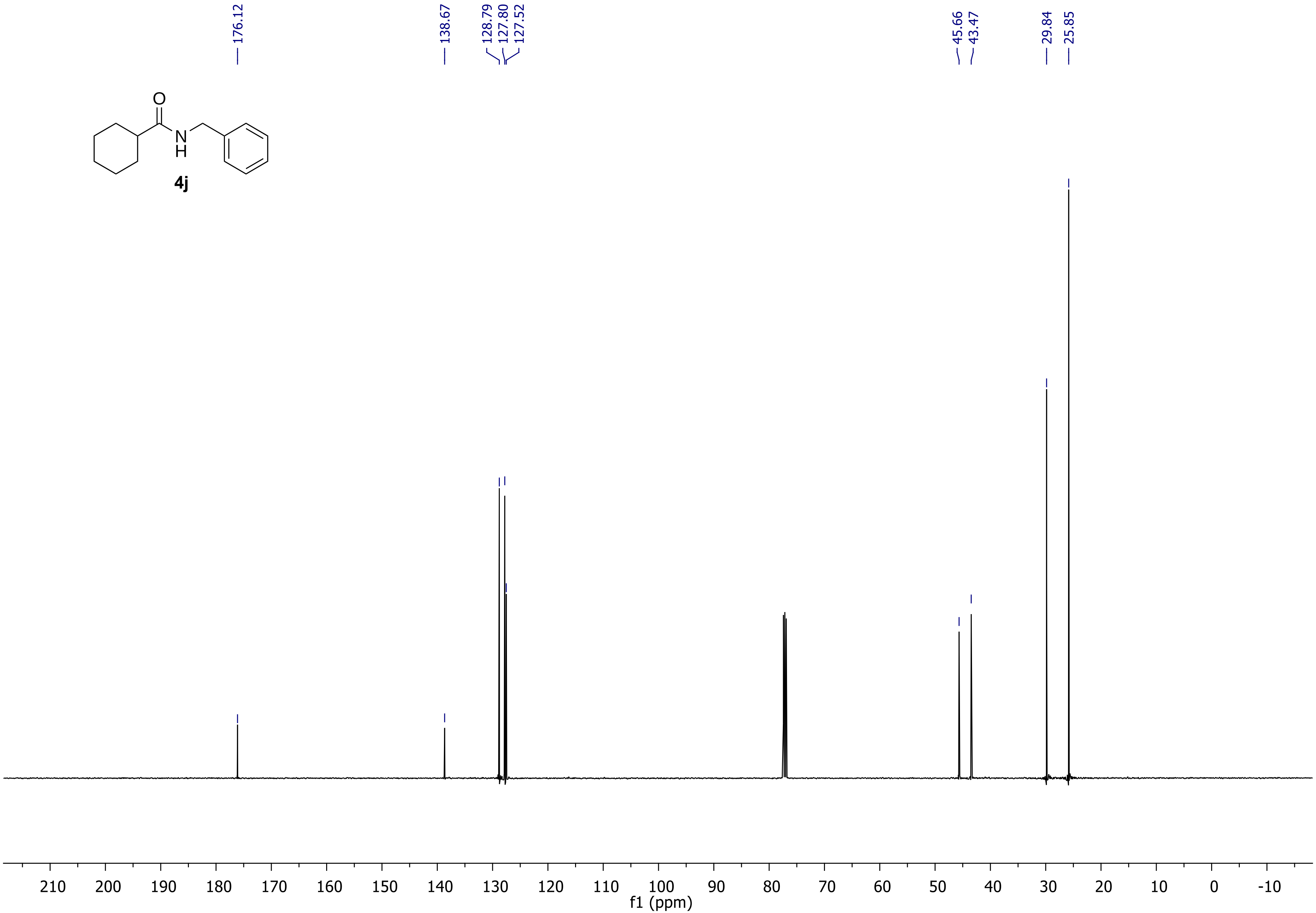

^1H NMR: 500 MHz, $CDCl_3$

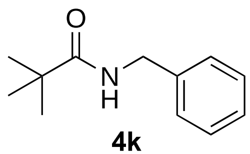

4k

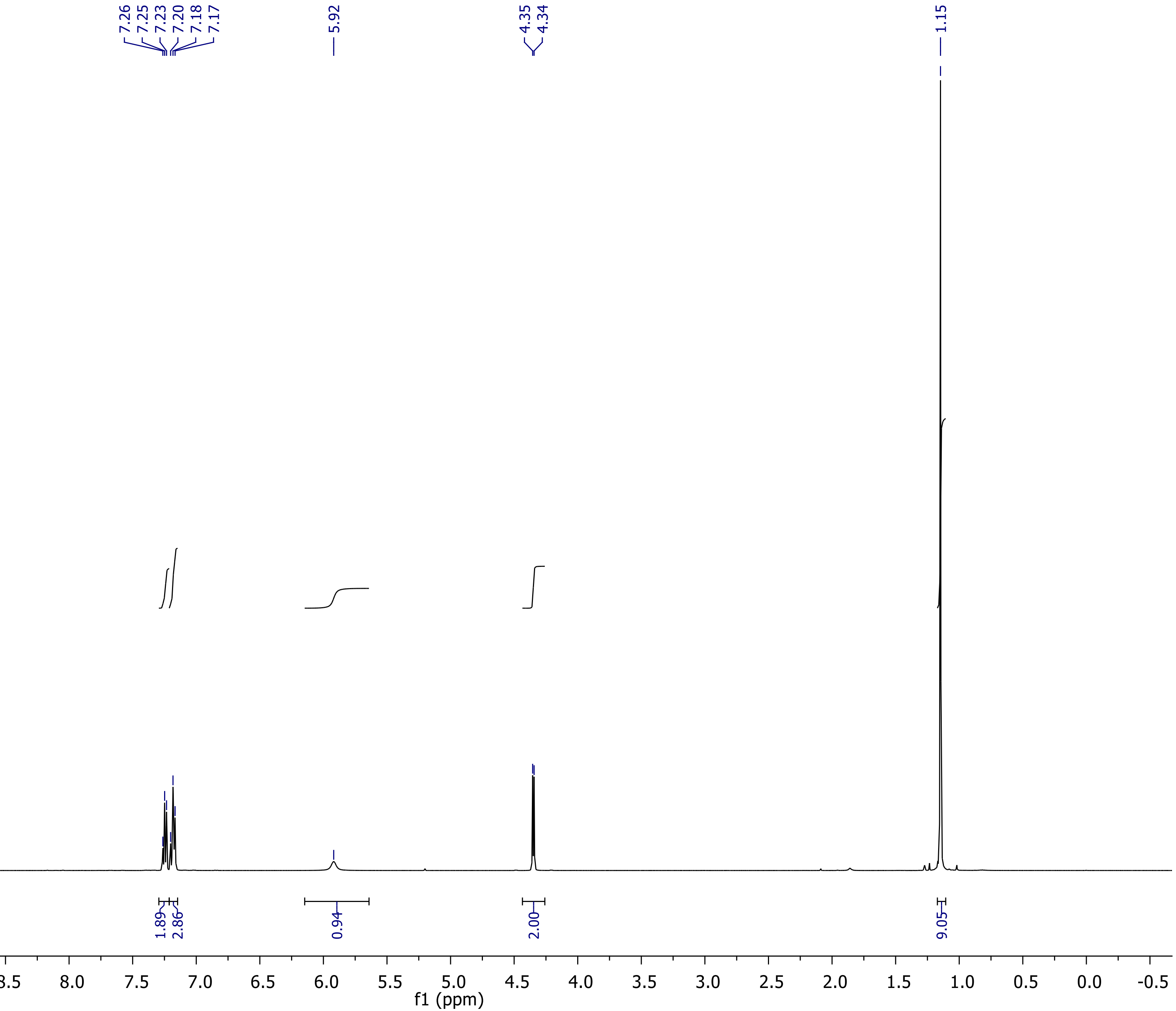

$^{13}C\{^{1}H\}$ NMR: 126 MHz, $CDCl_3$

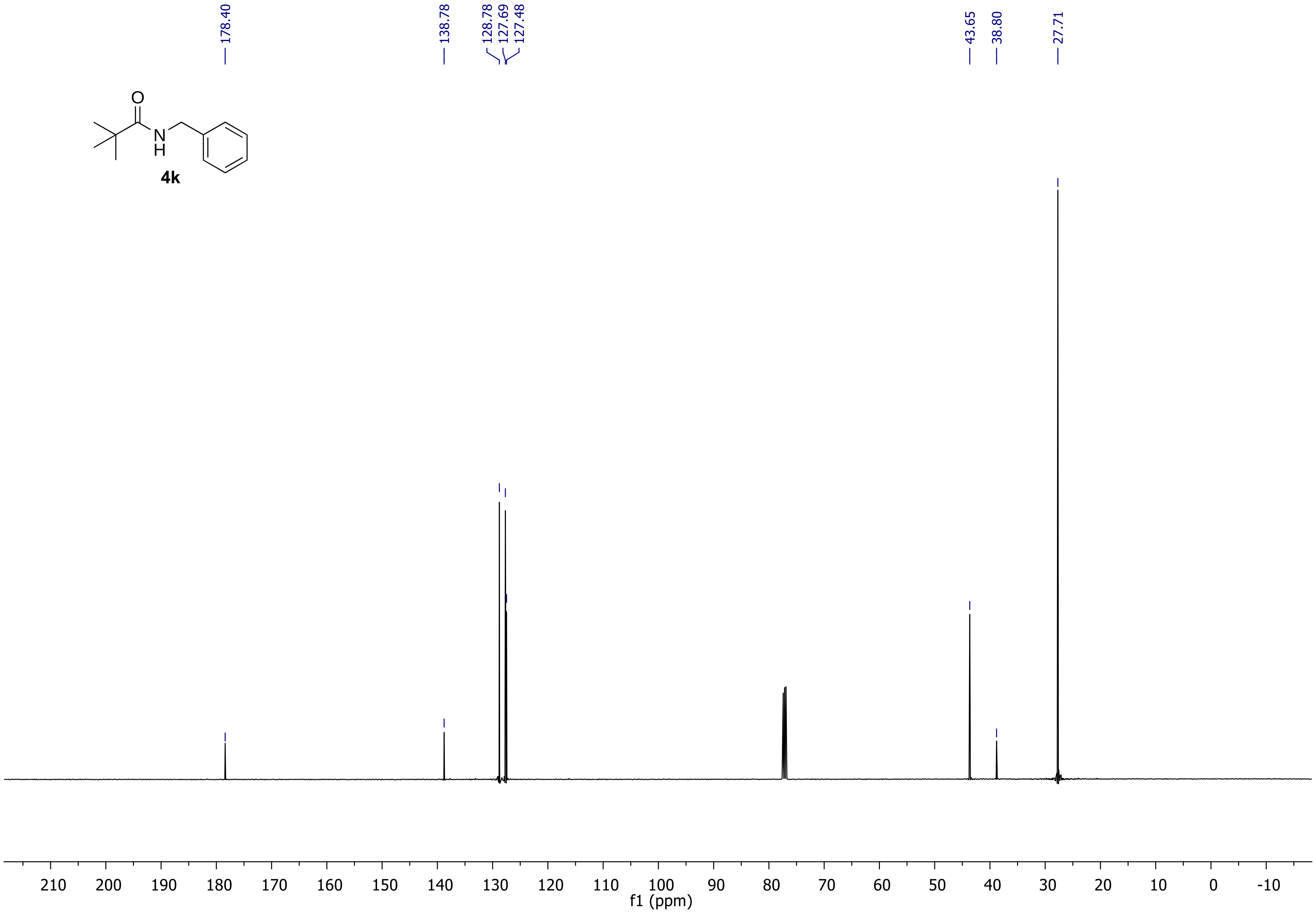

^1H NMR: 500 MHz, $CDCl_3$

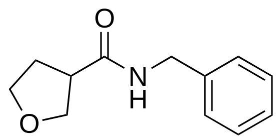

4l

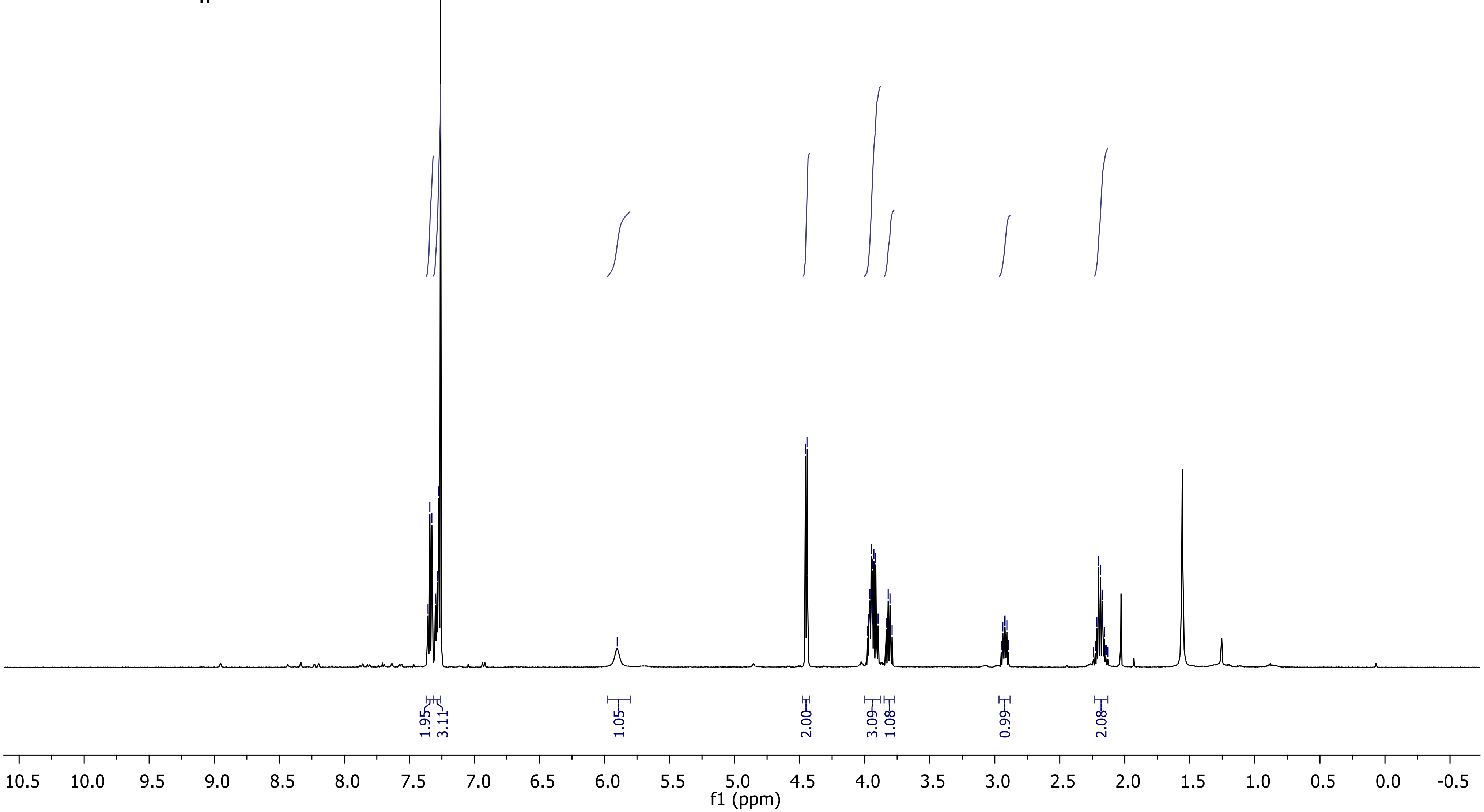

$^{13}C\{^1H\}$ NMR: 126 MHz, $CDCl_3$

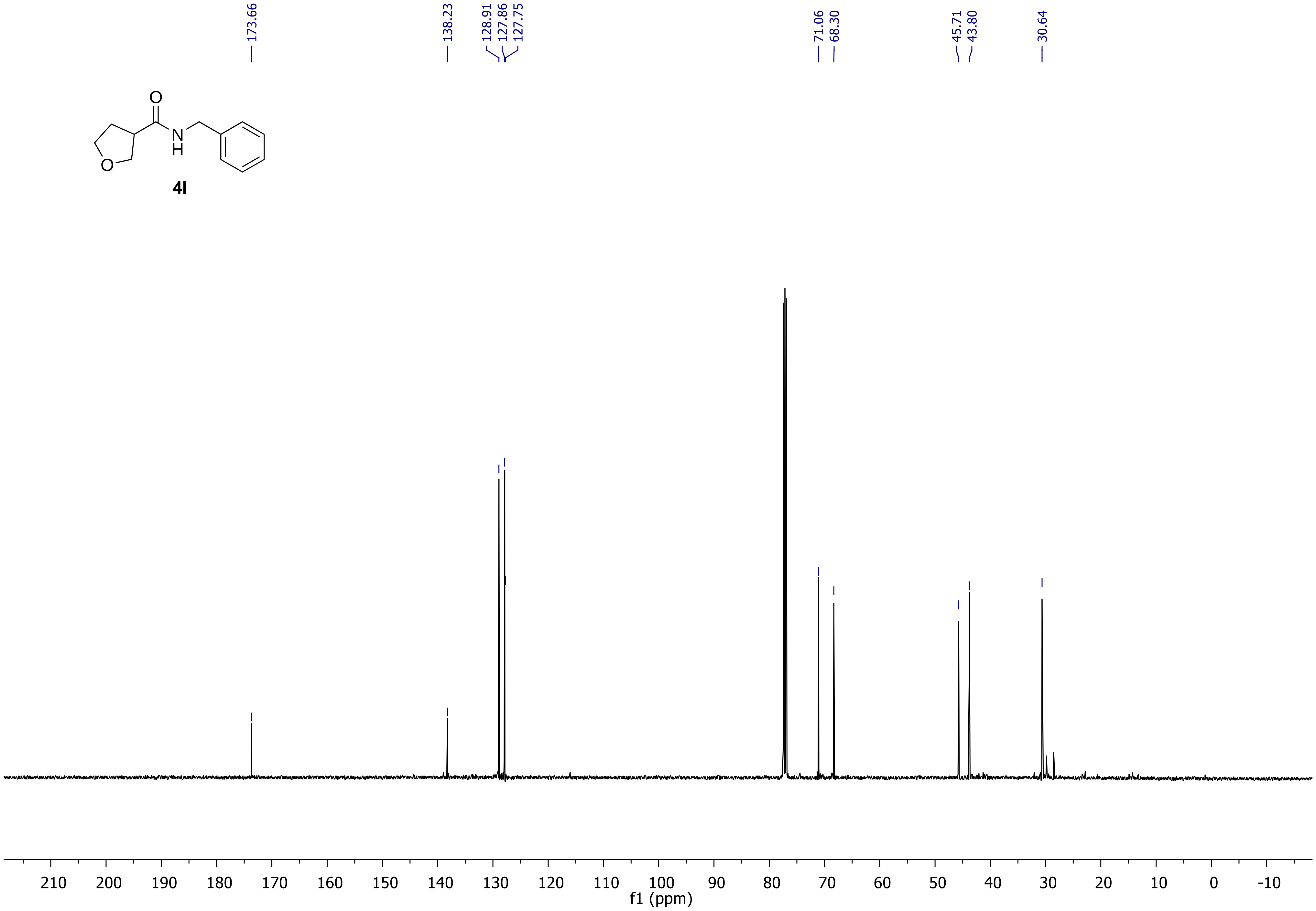

^1H NMR: 400 MHz, $CDCl_3$

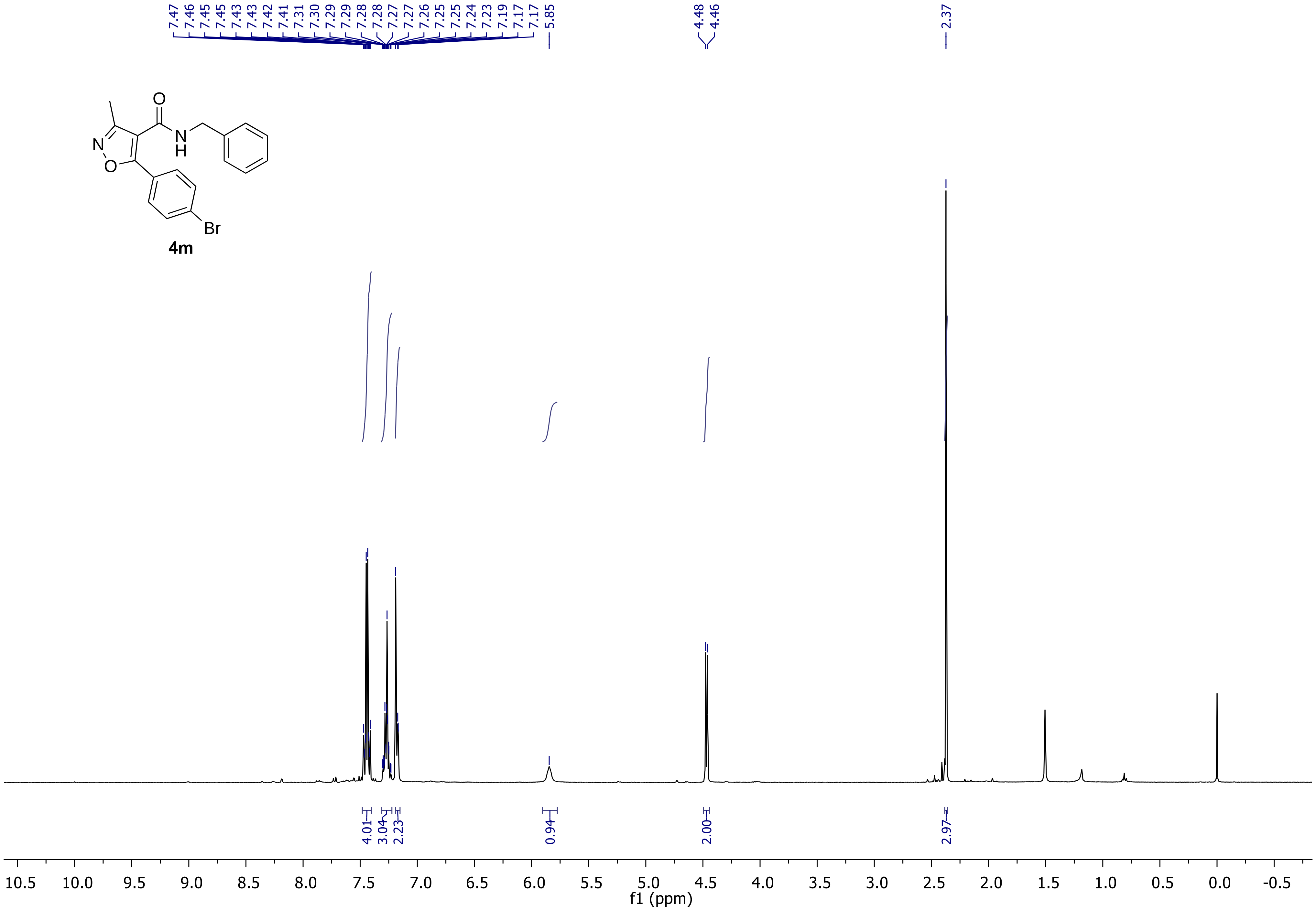

$^{13}C\{^{1}H\}$ NMR: 101 MHz, $CDCl_3$

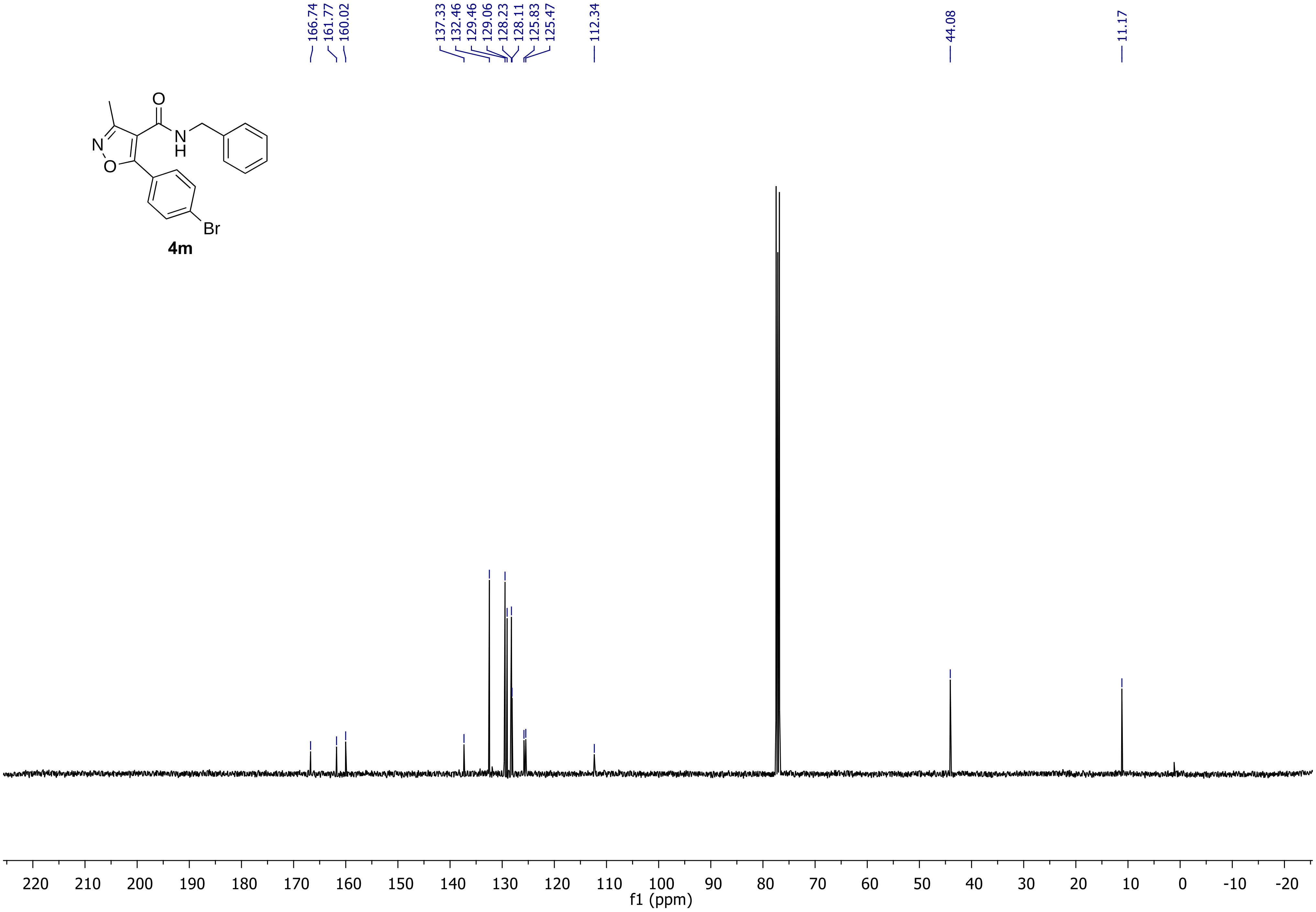

^{1}H NMR: 500 MHz, $CDCl_3$

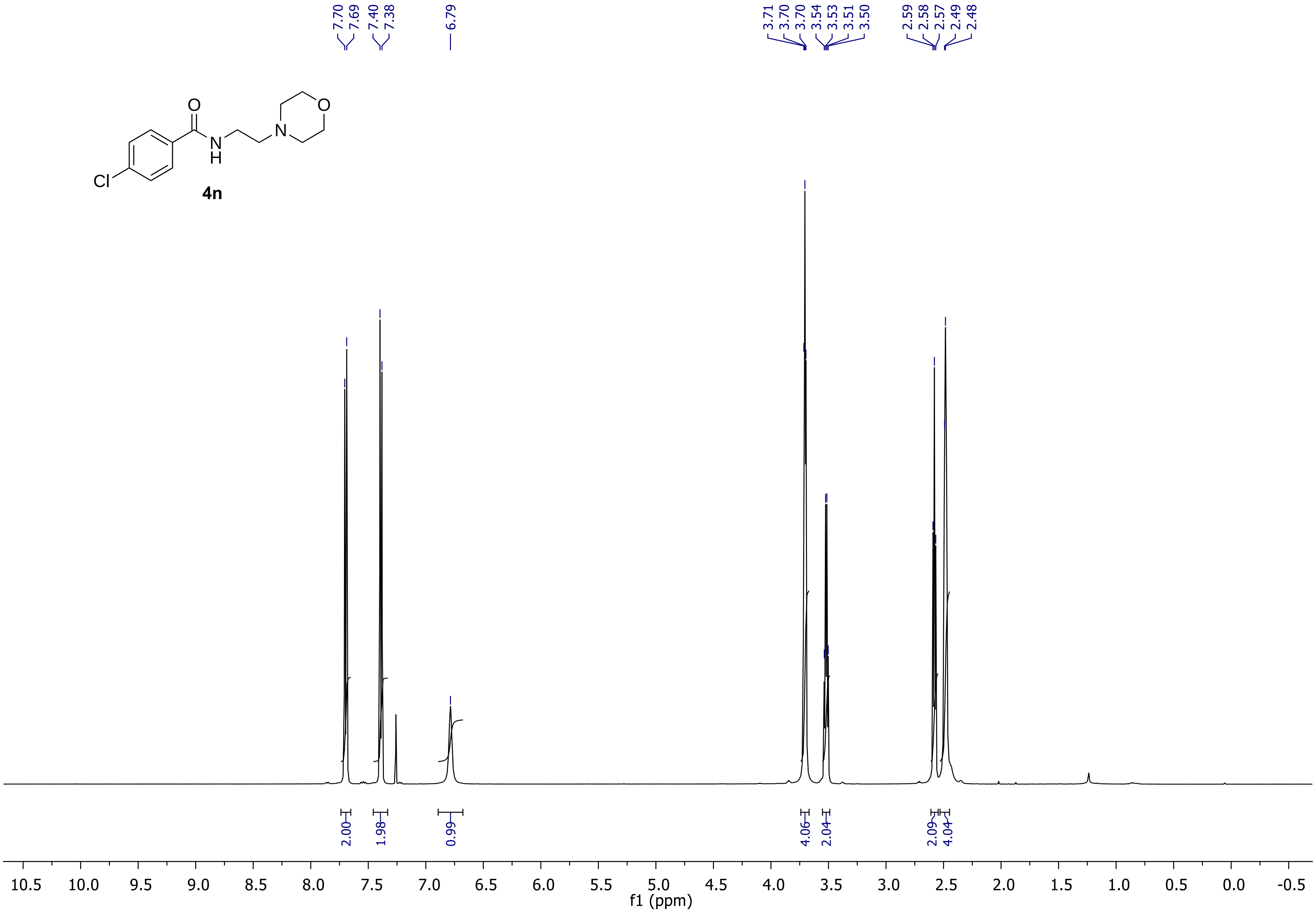

$^{13}C\{^1H\}$ NMR: 126 MHz, $CDCl_3$

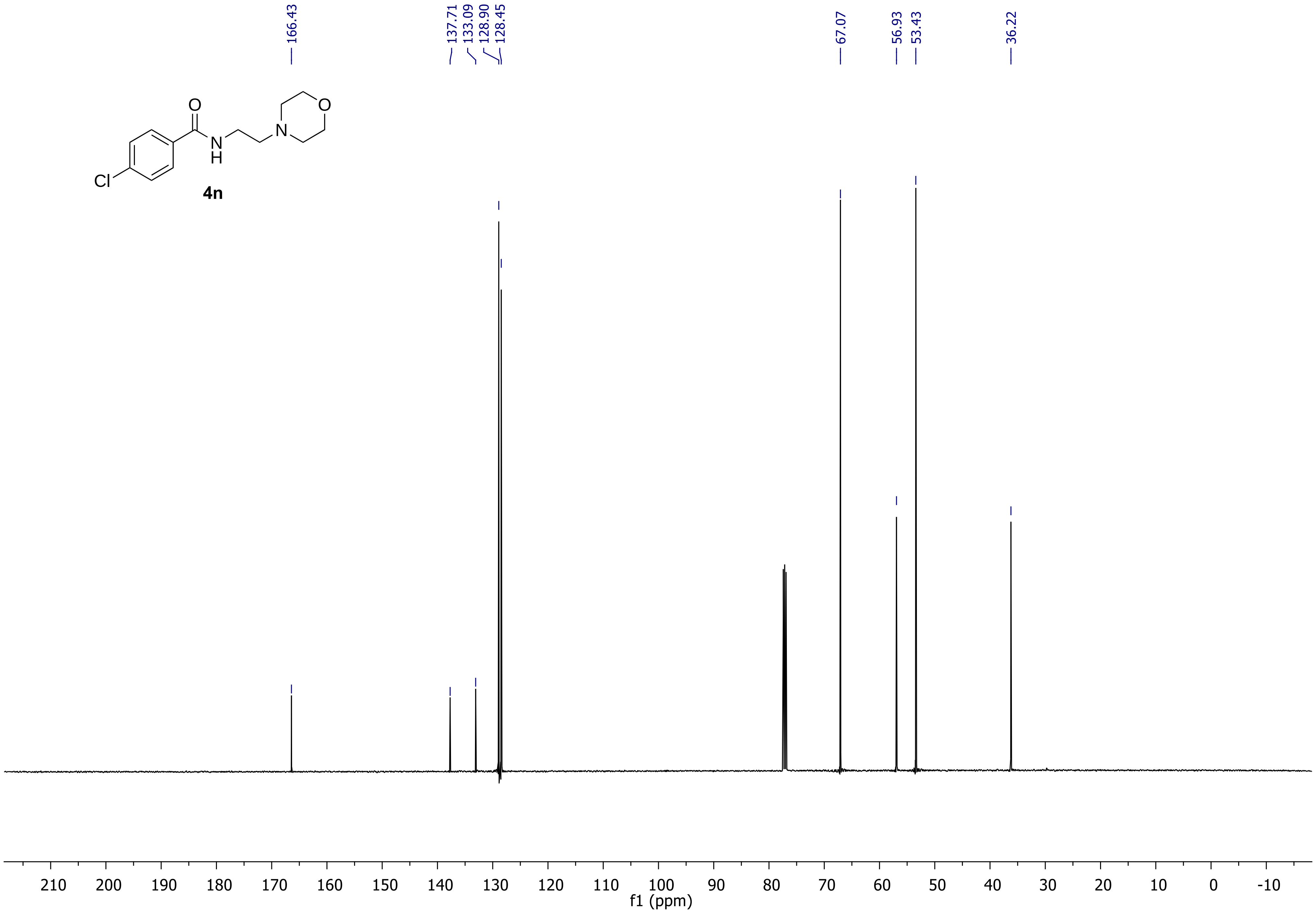

^1H NMR: 500 MHz, $CDCl_3$

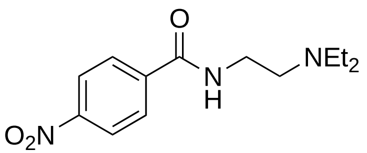

4o

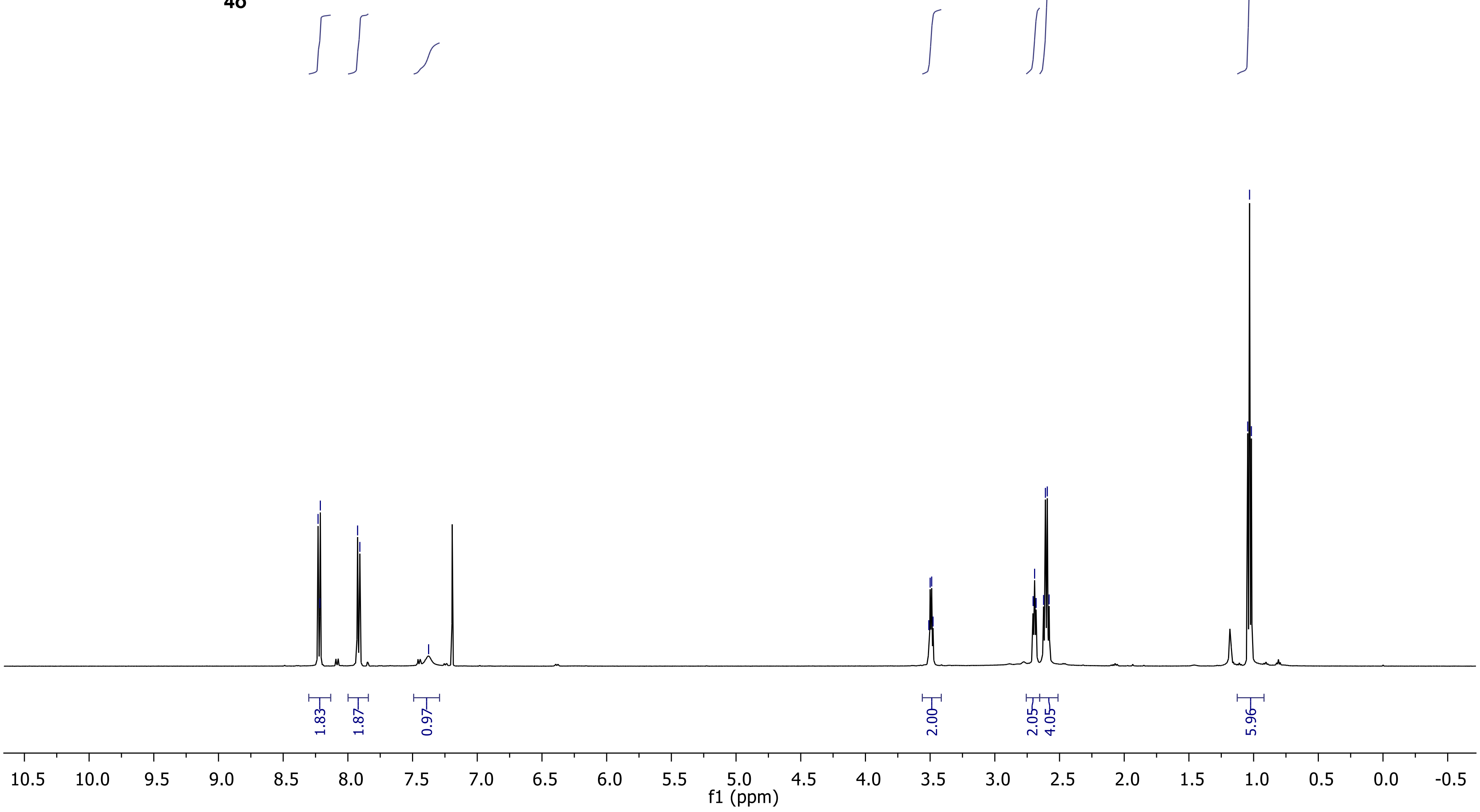

$^{13}C\{^{1}H\}$ NMR: 126 MHz, $CDCl_3$

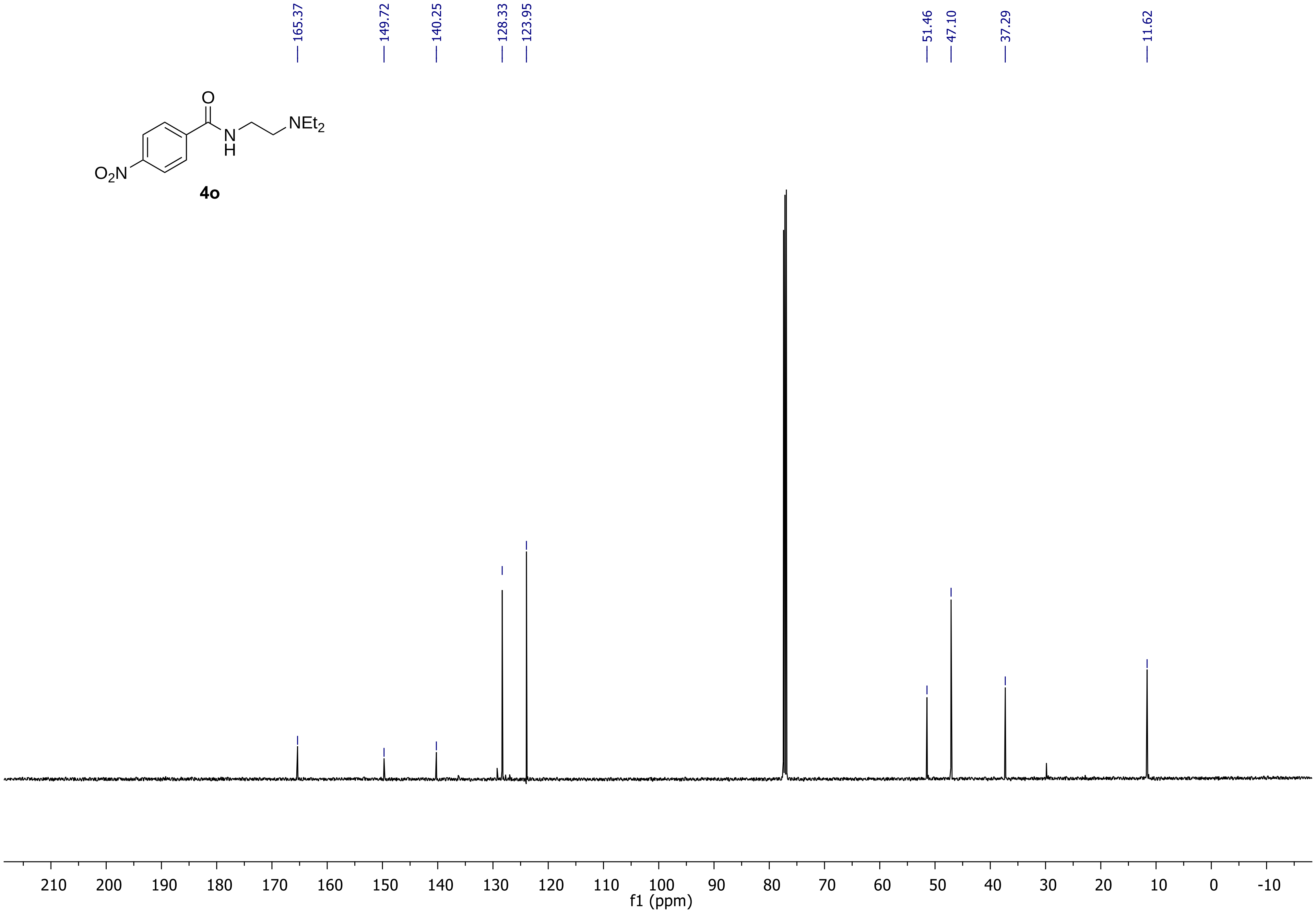

^1H NMR: 500 MHz, $CDCl_3$

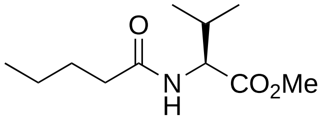

4p

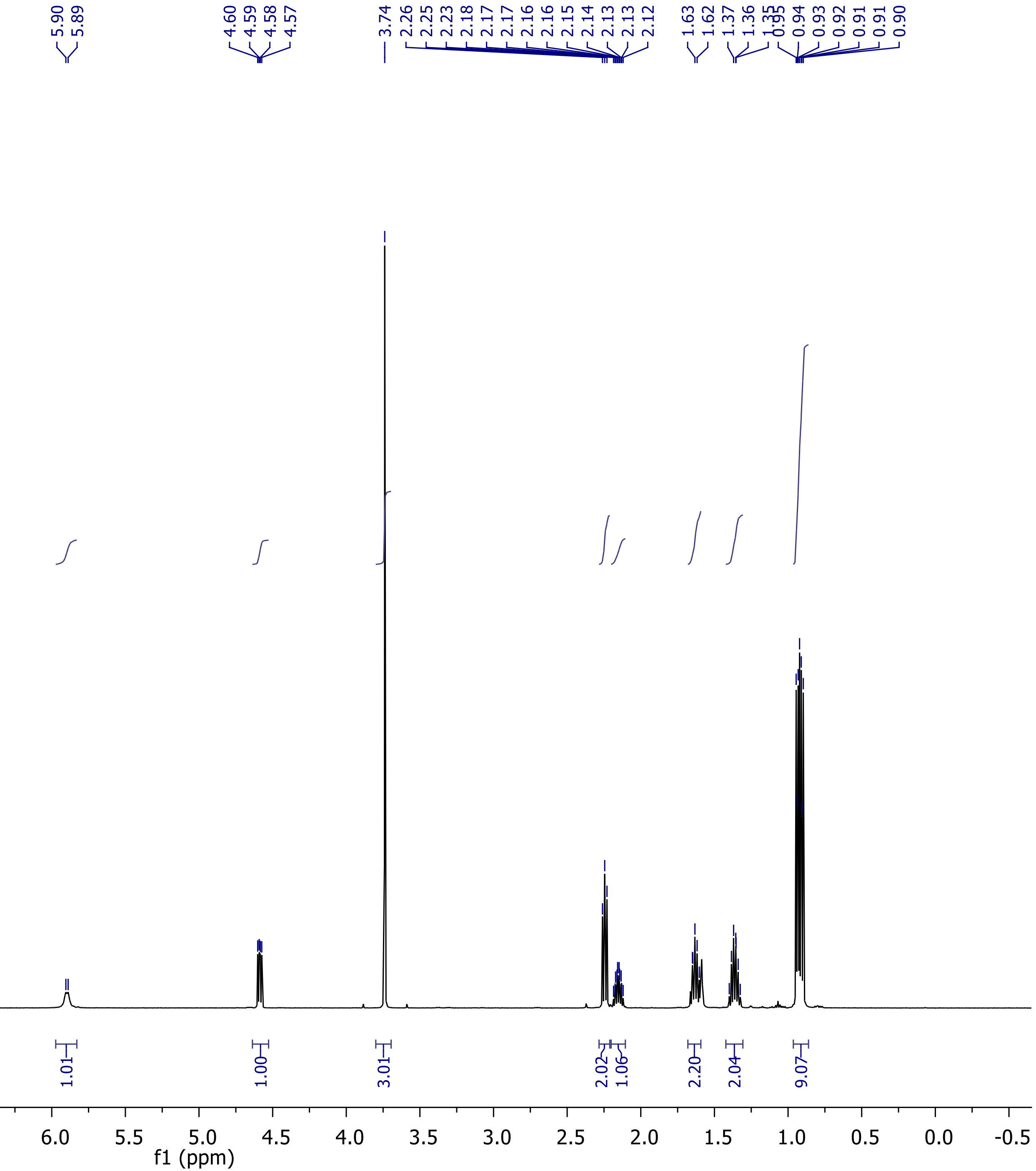

$^{13}C\{^1H\}$ NMR: 126 MHz, $CDCl_3$

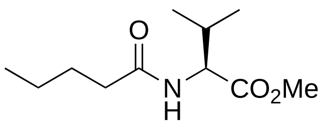

4p

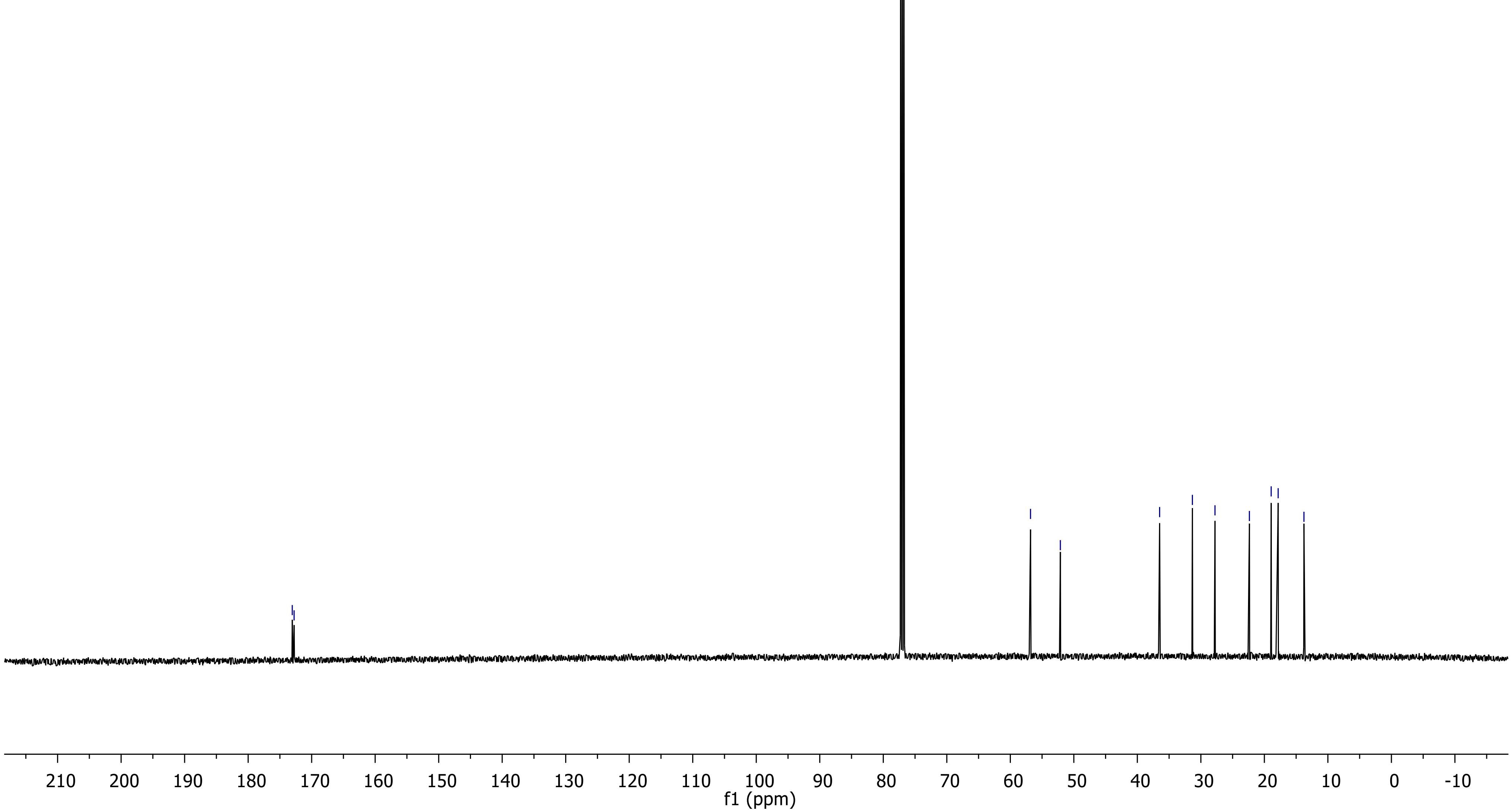

^{1}H NMR: 500 MHz, D_6-DMSO

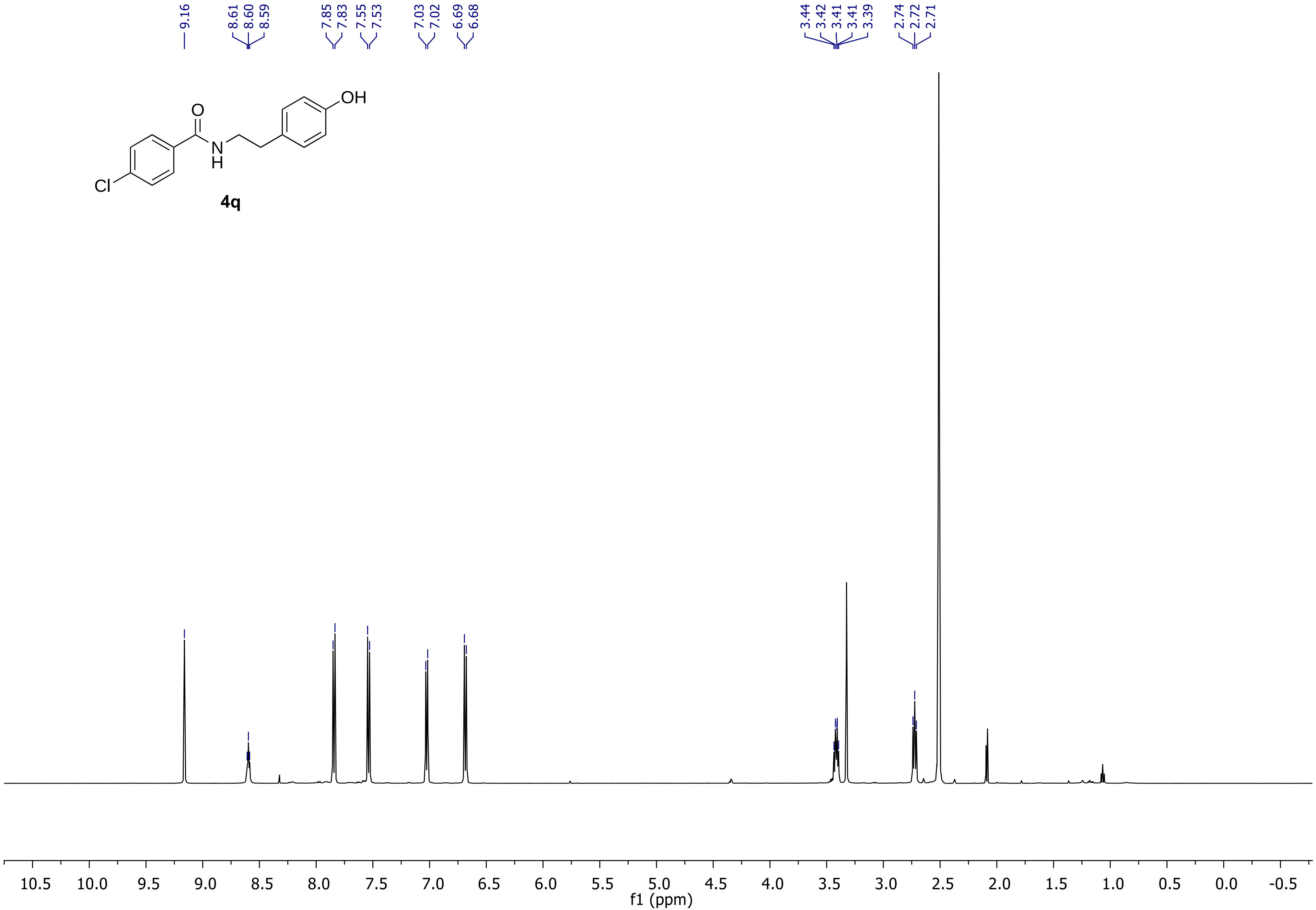

4q

$^{13}C\{^1H\}$ NMR: 126 MHz, D_6-DMSO

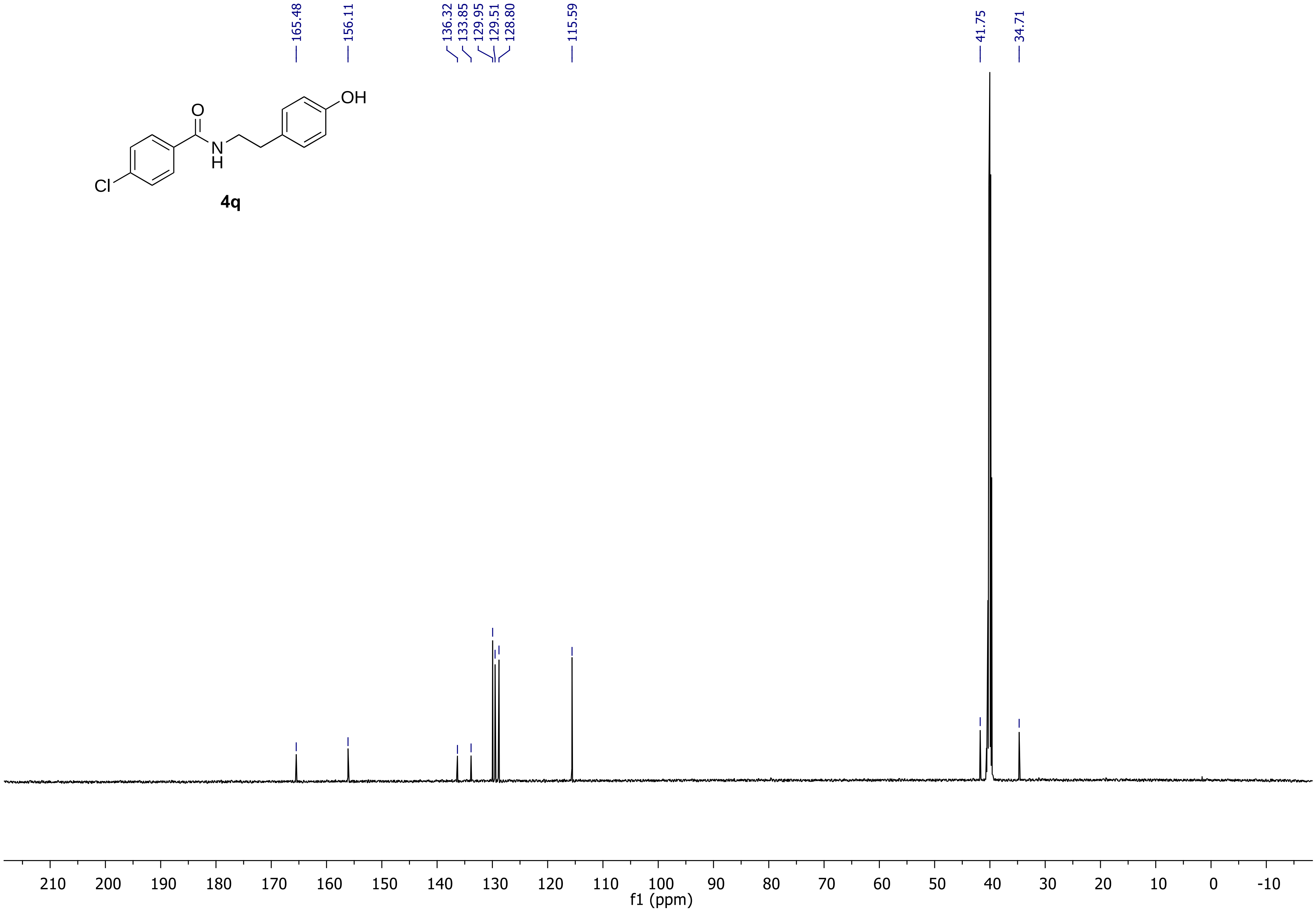

^{1}H NMR: 500 MHz, $CDCl_3$

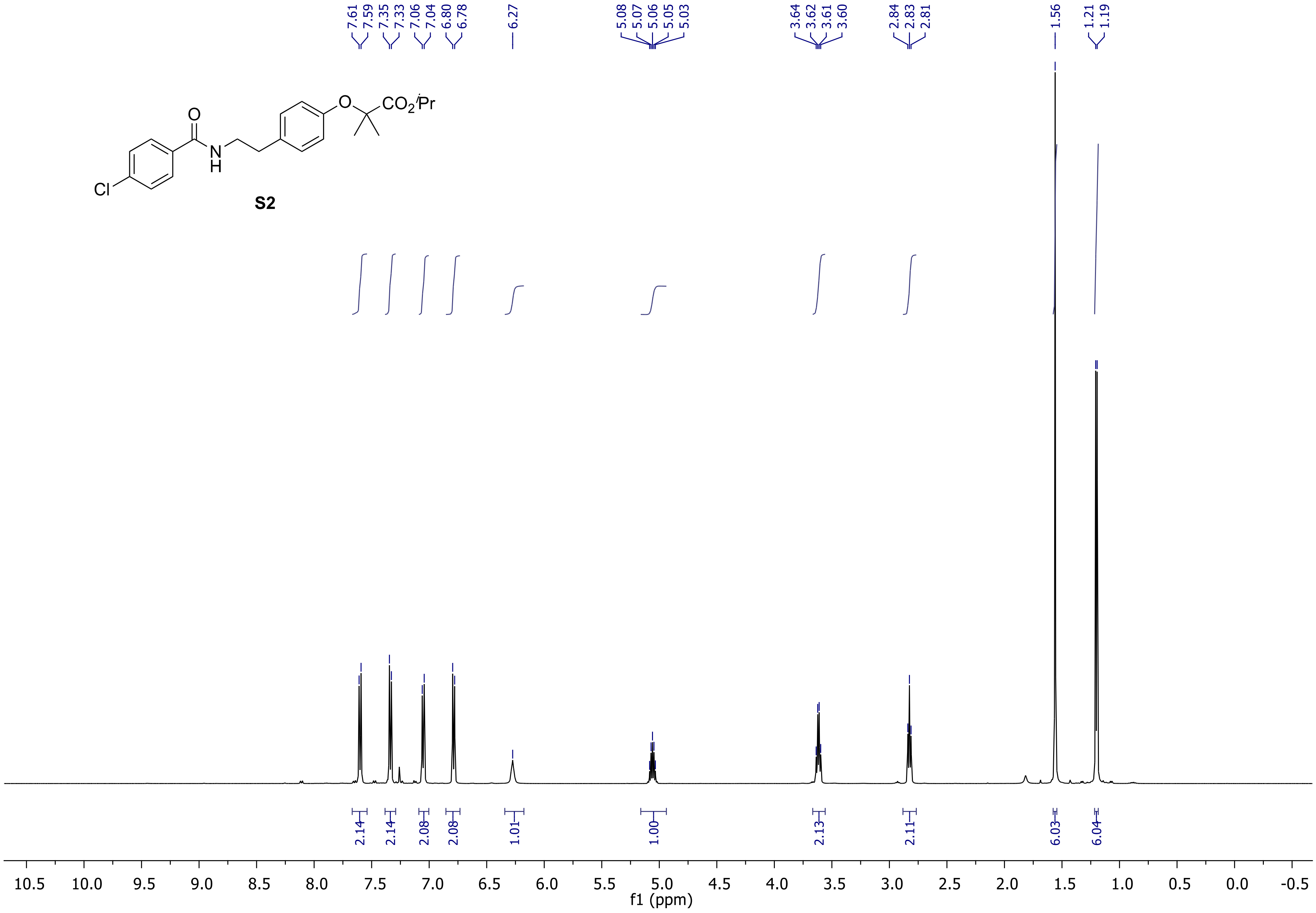

$^{13}C\{^1H\}$ NMR: 126 MHz, $CDCl_3$

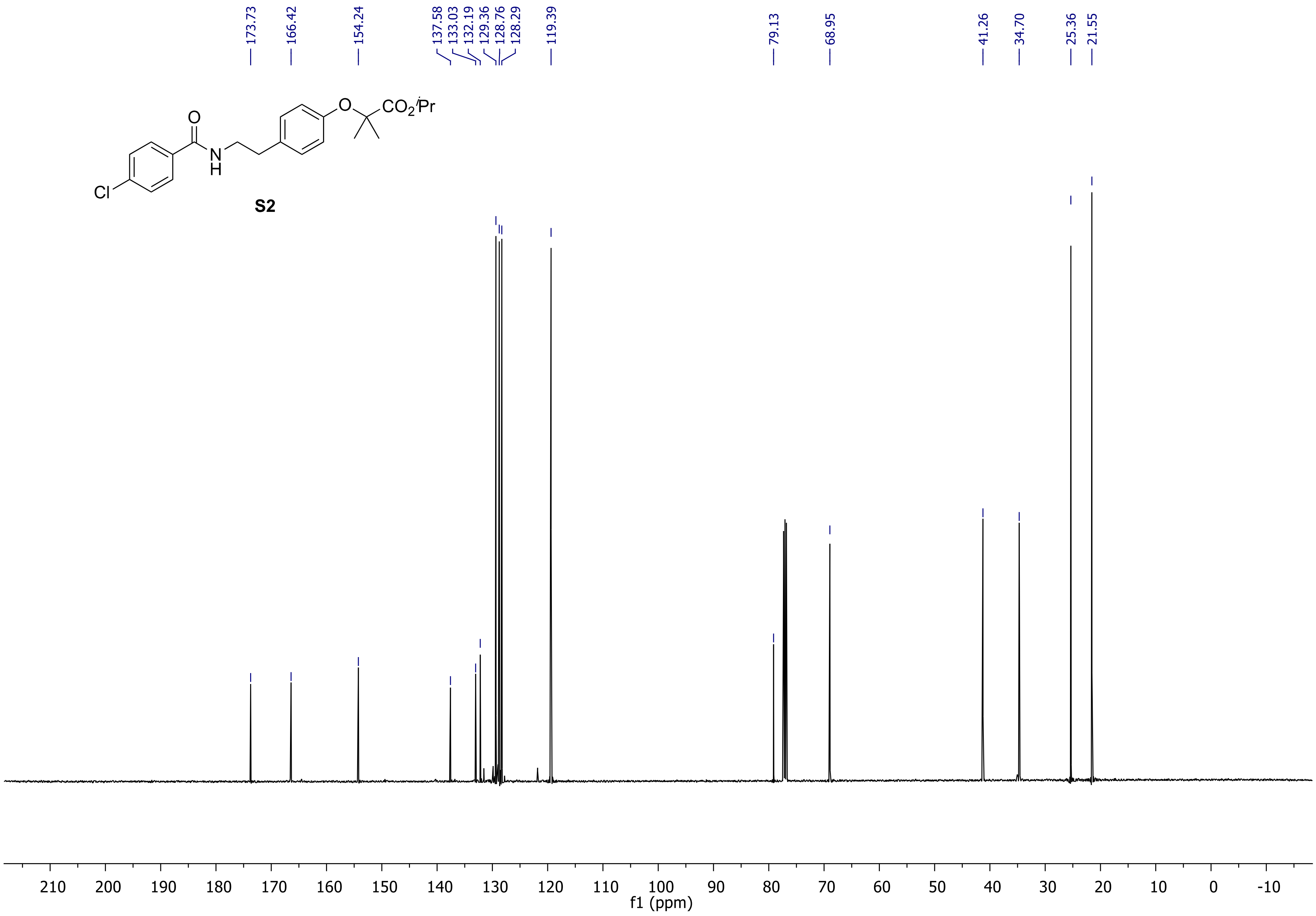

S2

^1H NMR: 500 MHz, D_6-DMSO

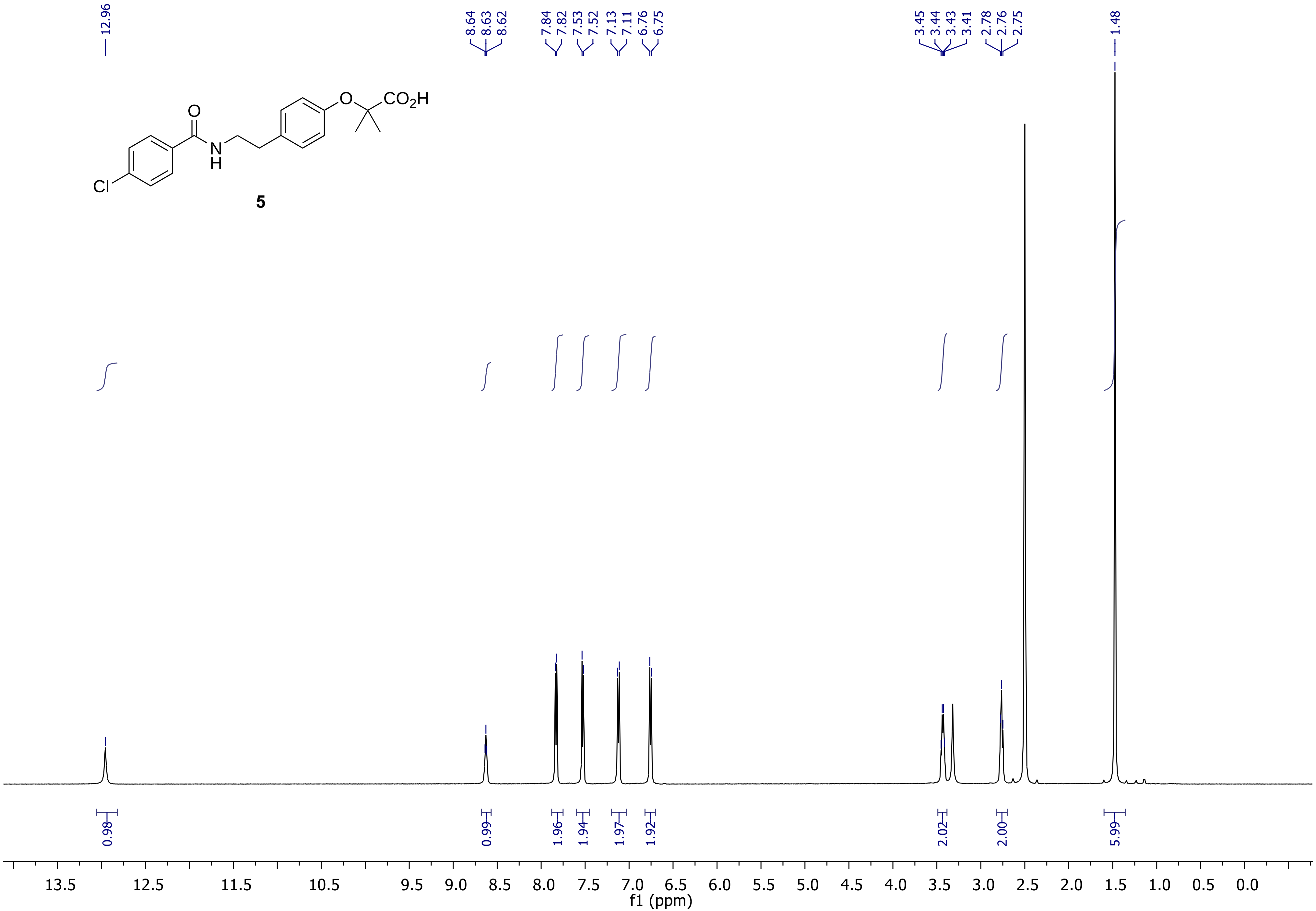

$^{13}C\{^1H\}$ NMR: 126 MHz, D_6-DMSO

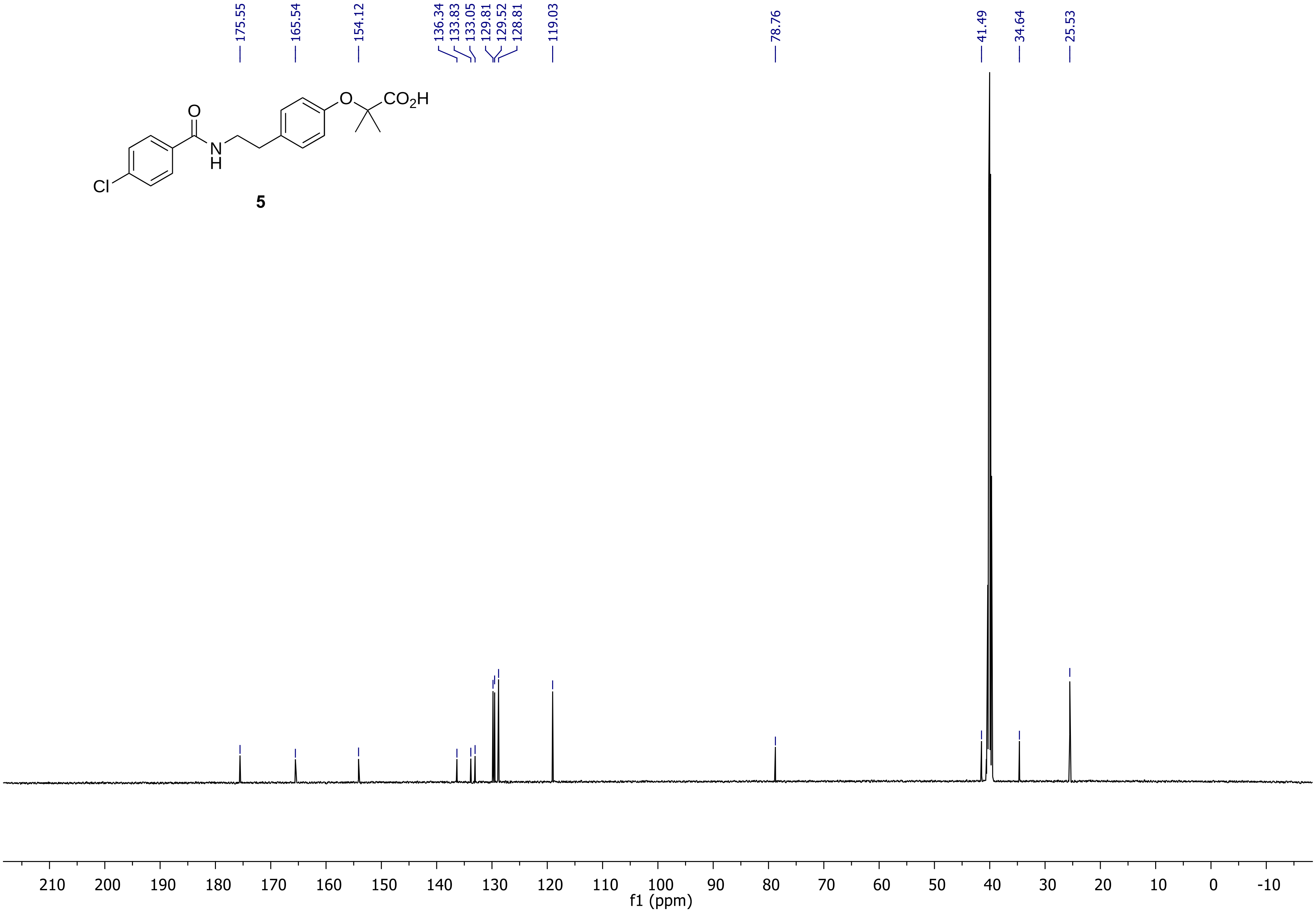

^{1}H NMR: 500 MHz, D_6-DMSO

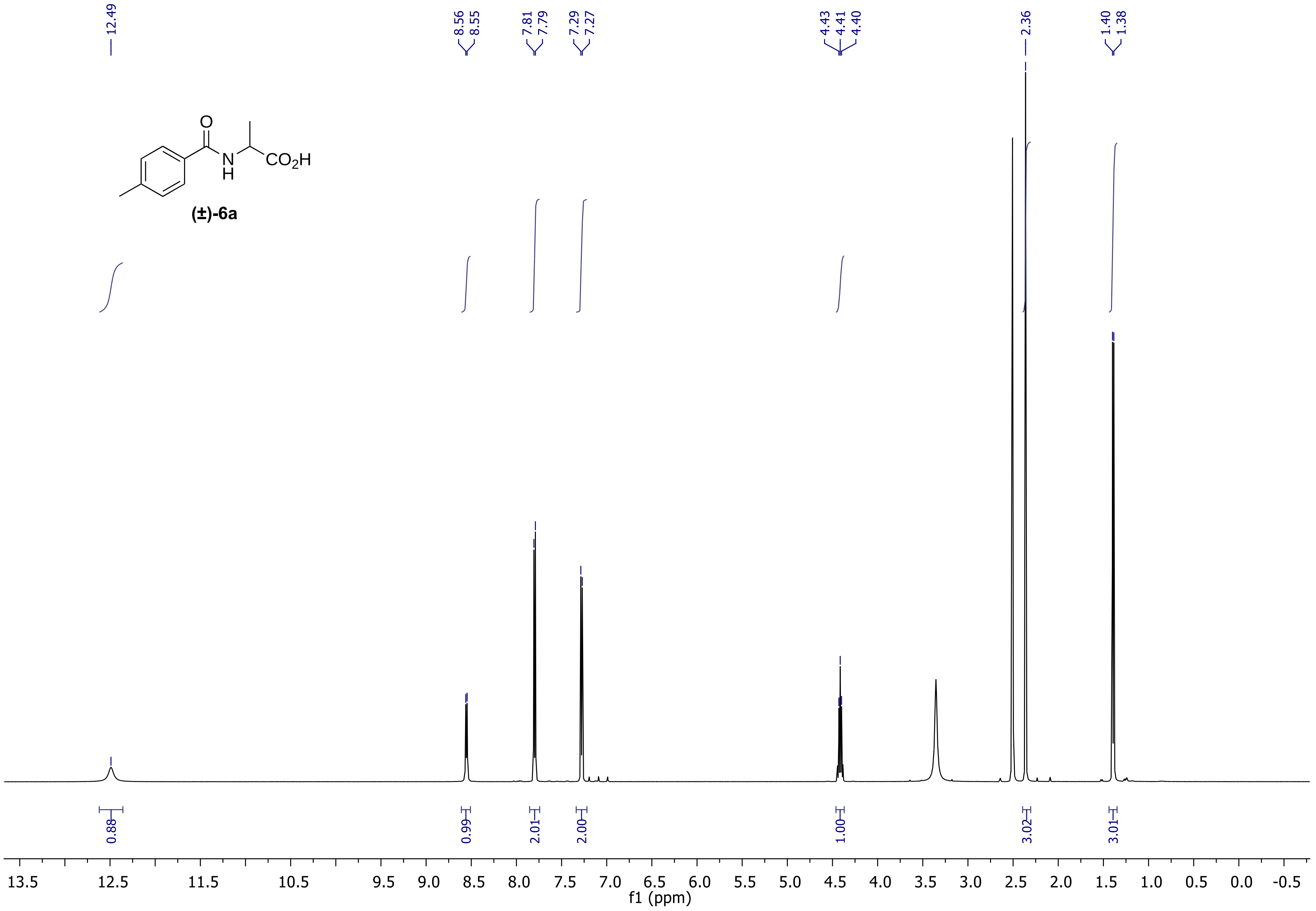

$^{13}C\{^1H\}$ NMR: 126 MHz, D_6-DMSO

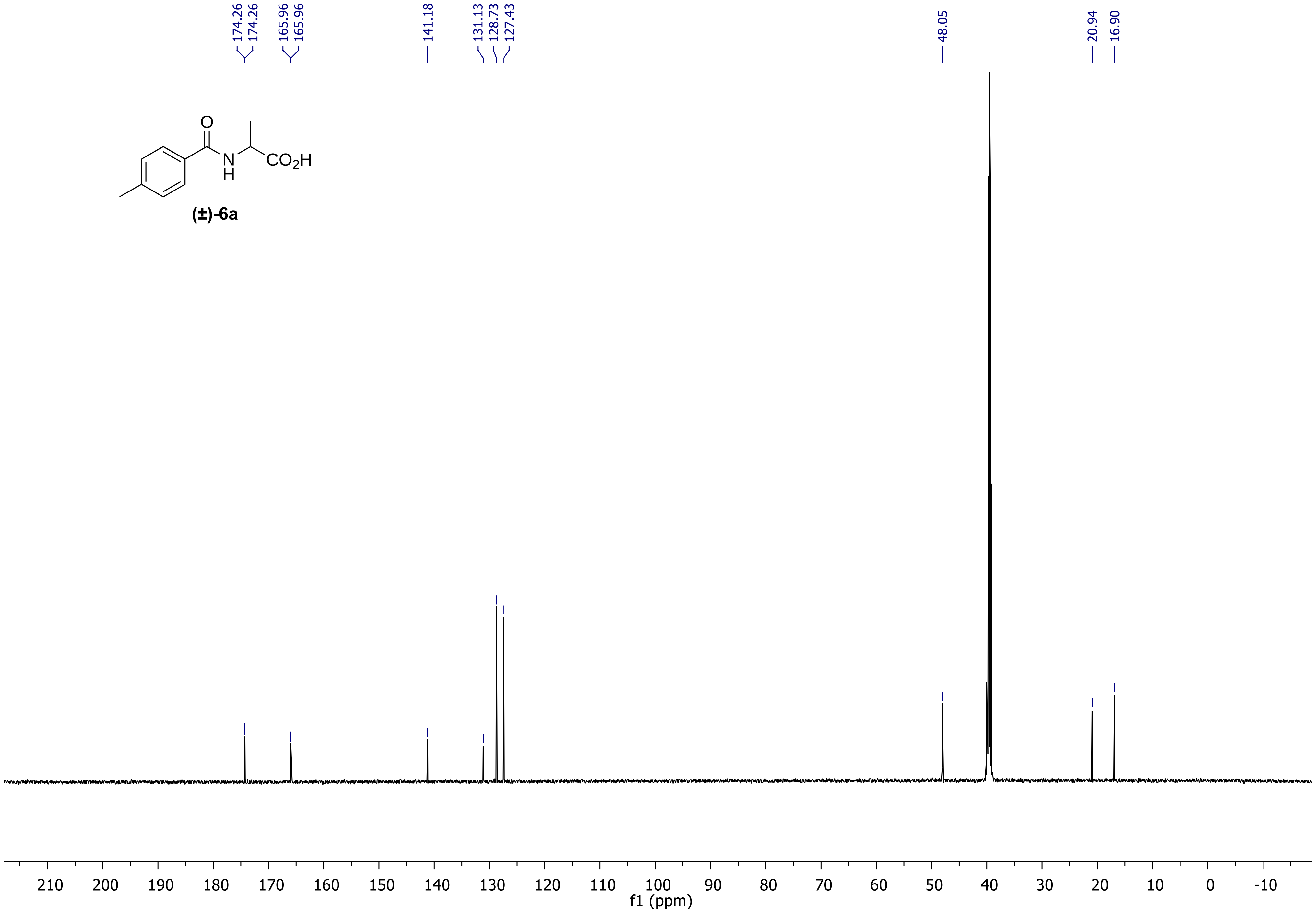

^1H NMR: 500 MHz, D_6-DMSO

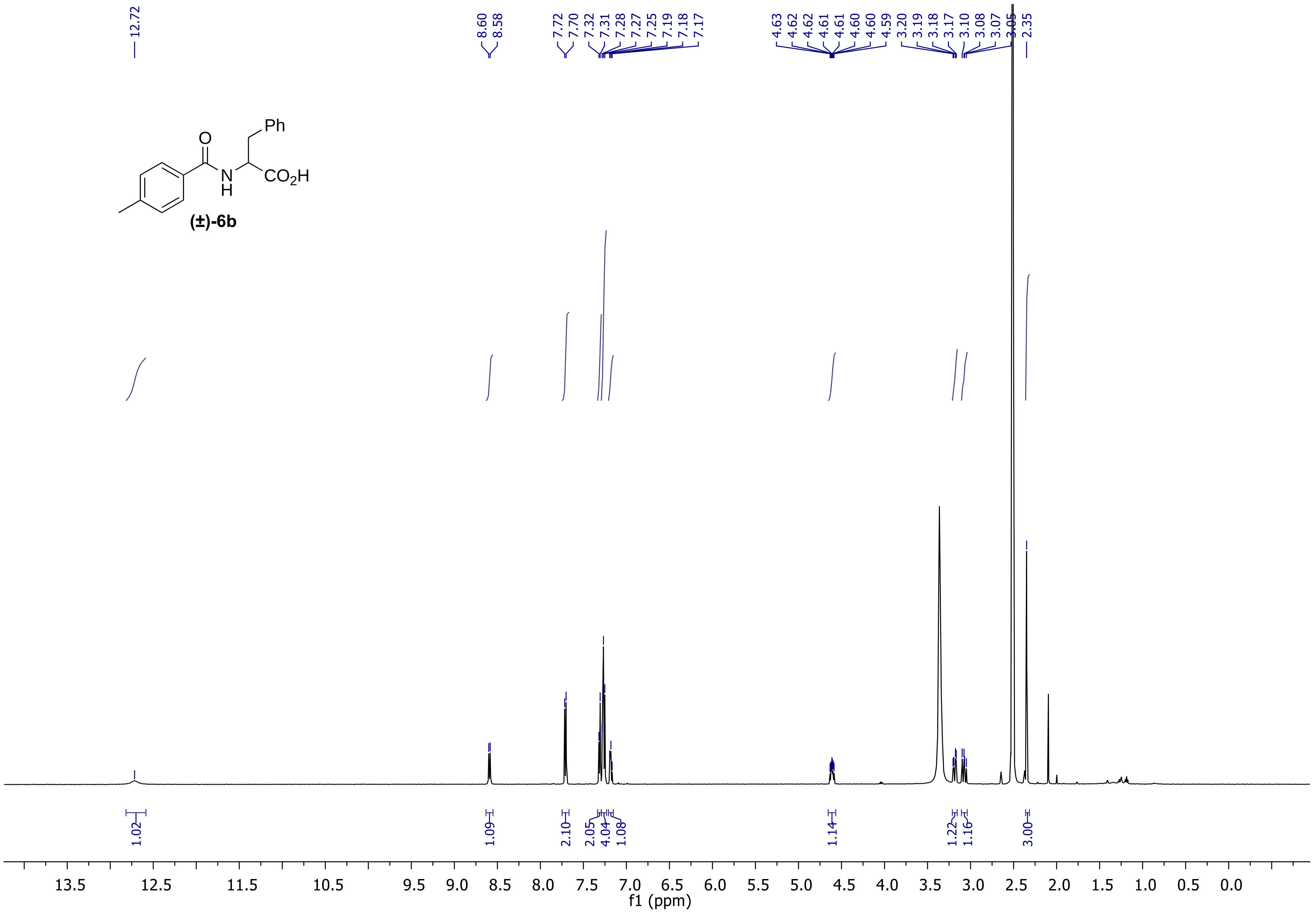

(±)-6b

$^{13}C\{^{1}H\}$ NMR: 126 MHz, D_6-DMSO

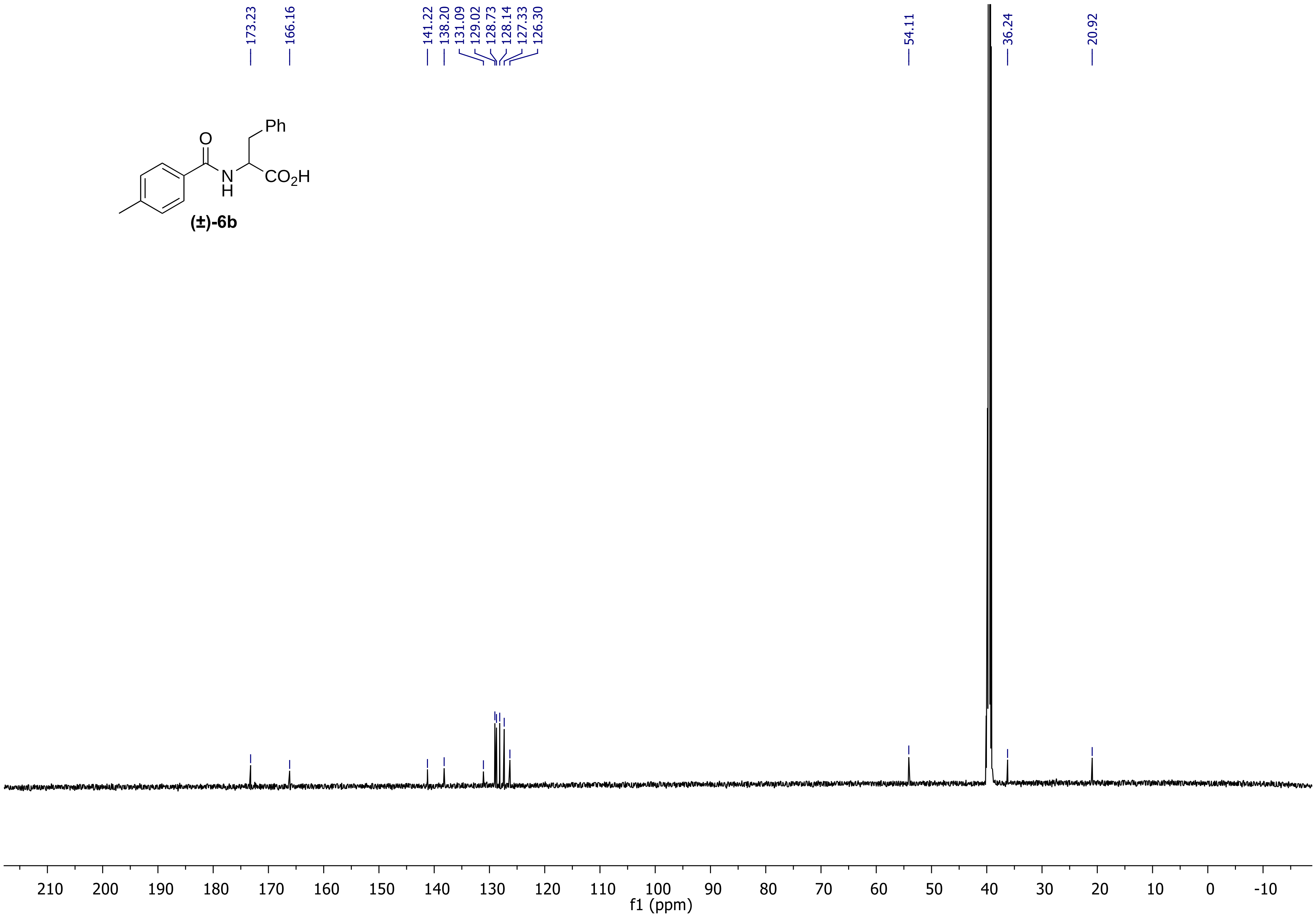

^{1}H NMR: 400 MHz, D_6-DMSO

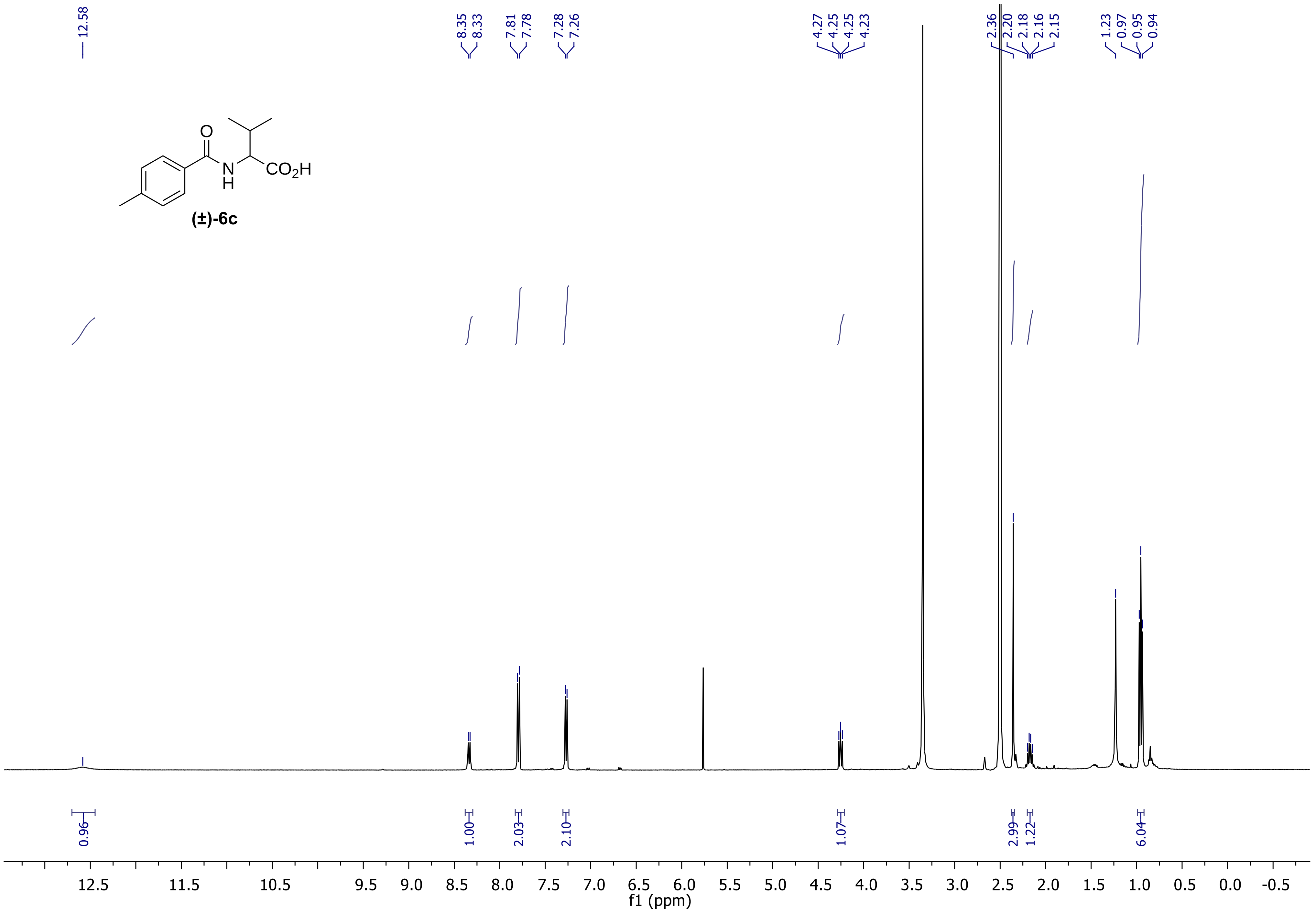

$^{13}C\{^1H\}$ NMR: 101 MHz, D_6-DMSO

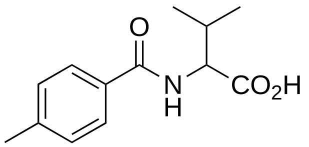

(±)-6c

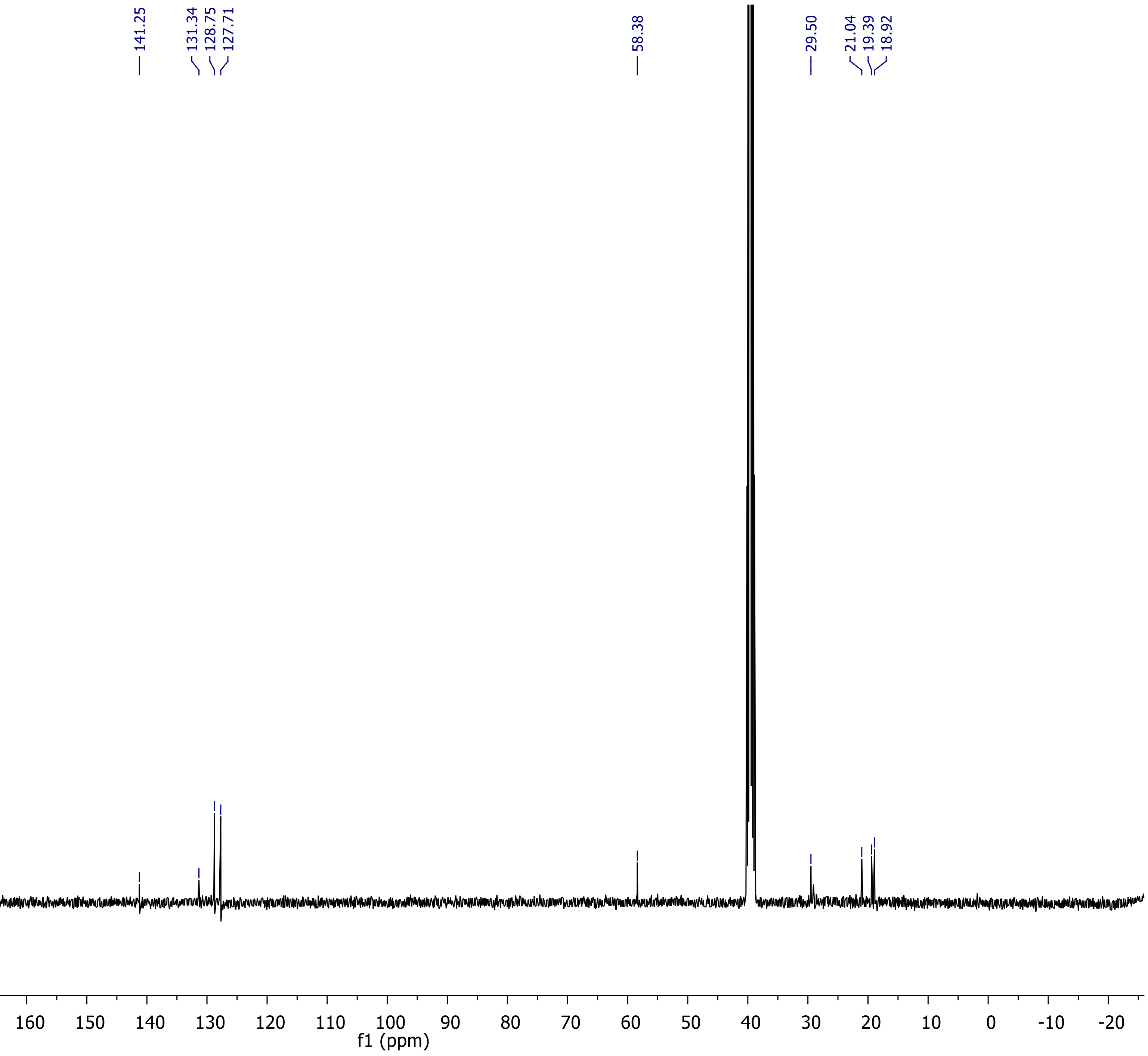

^1H NMR: 500 MHz, CD_3OD

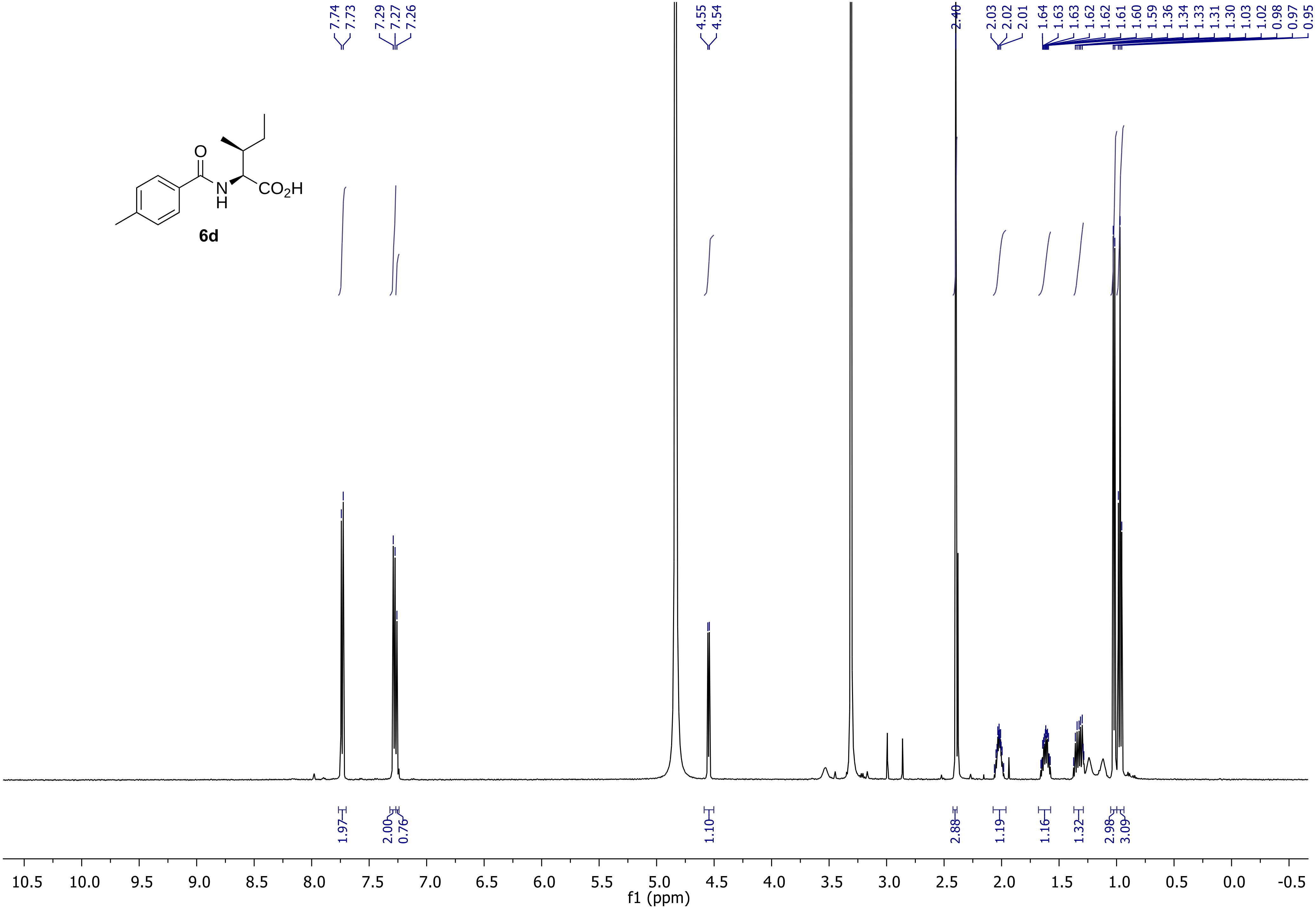

$^{13}C\{^1H\}$ NMR: 126 MHz, CD_3OD

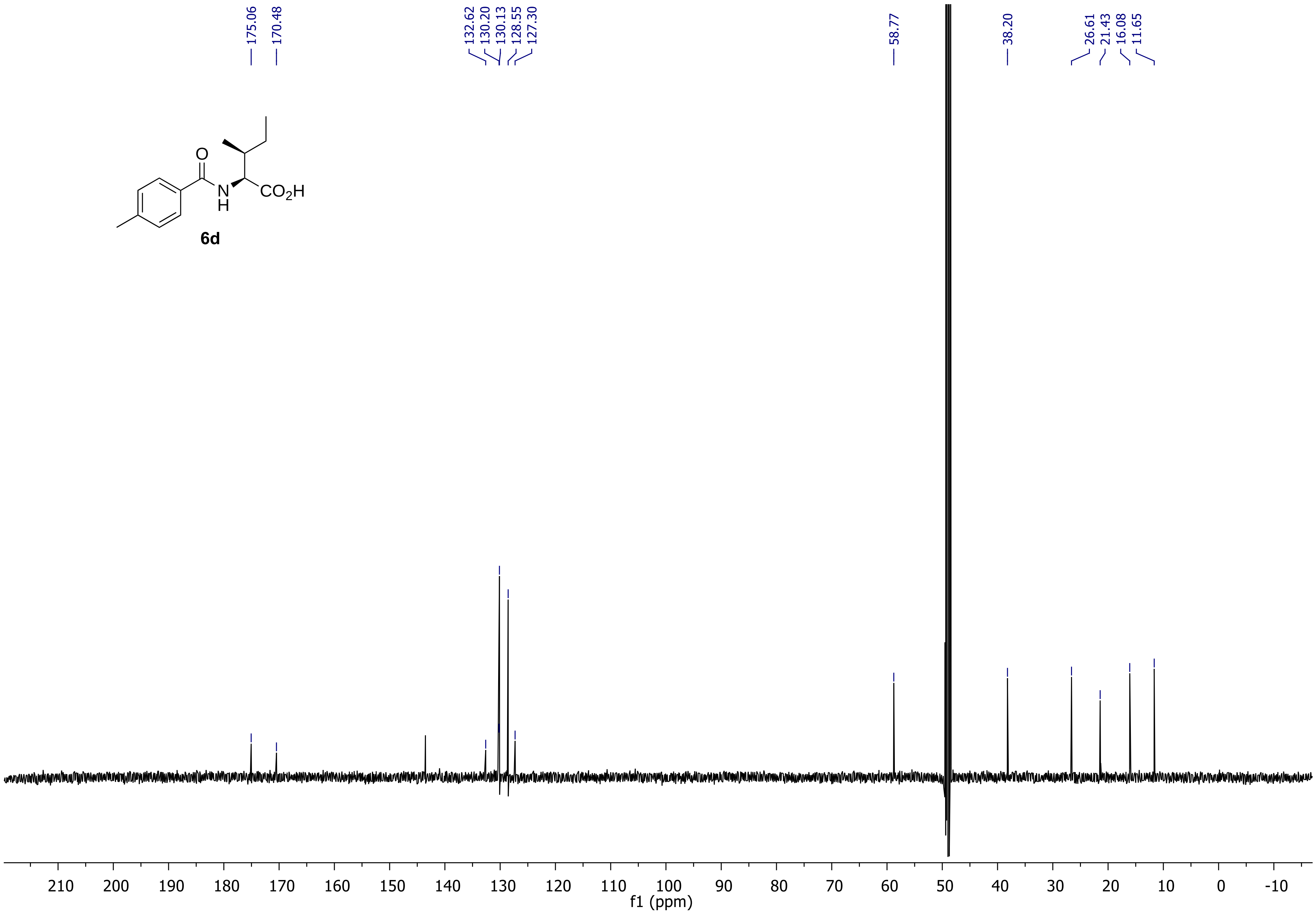

^{1}H NMR: 500 MHz, D_6-DMSO

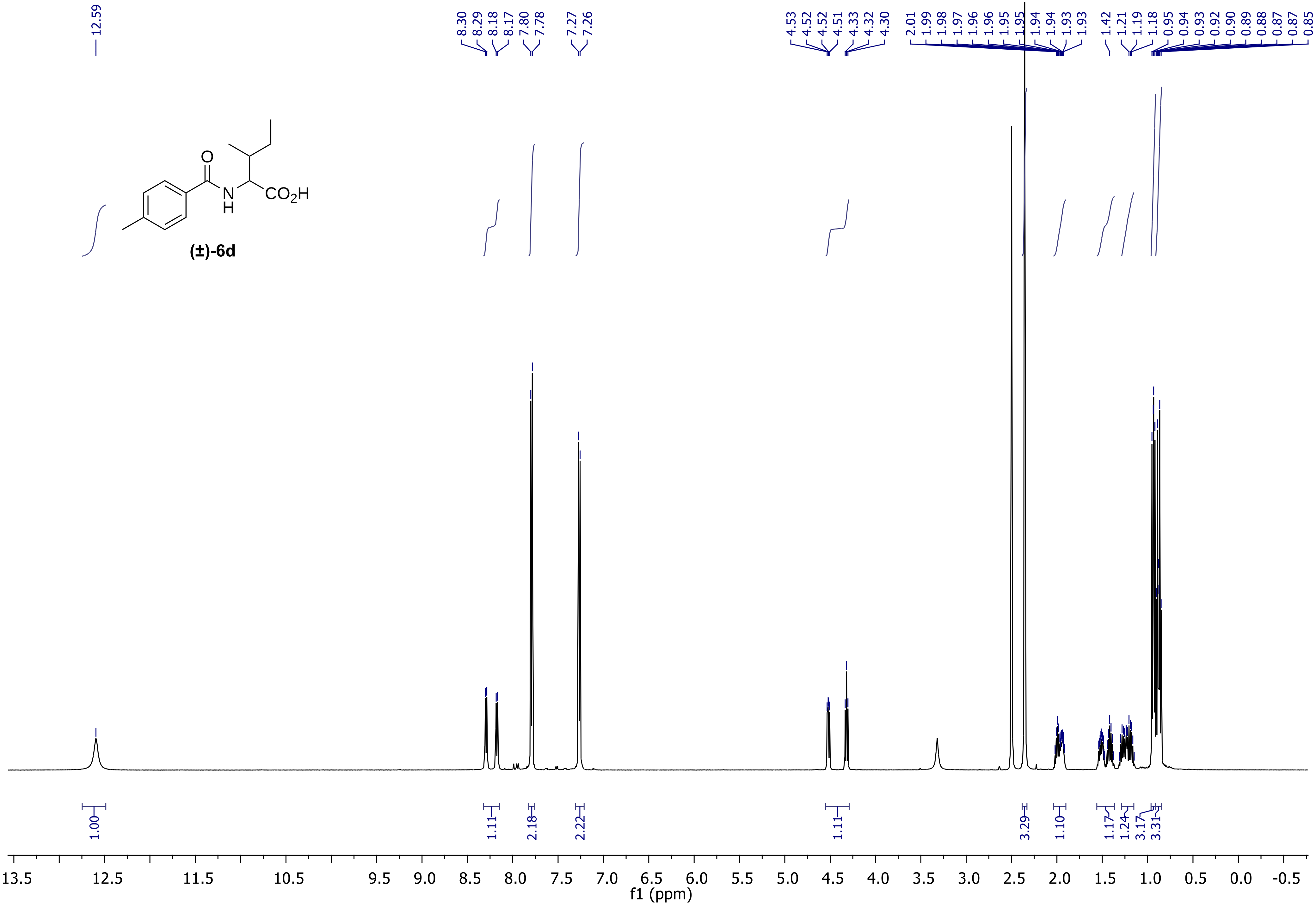

$^{13}C\{^1H\}$ NMR: 126 MHz, D_6-DMSO

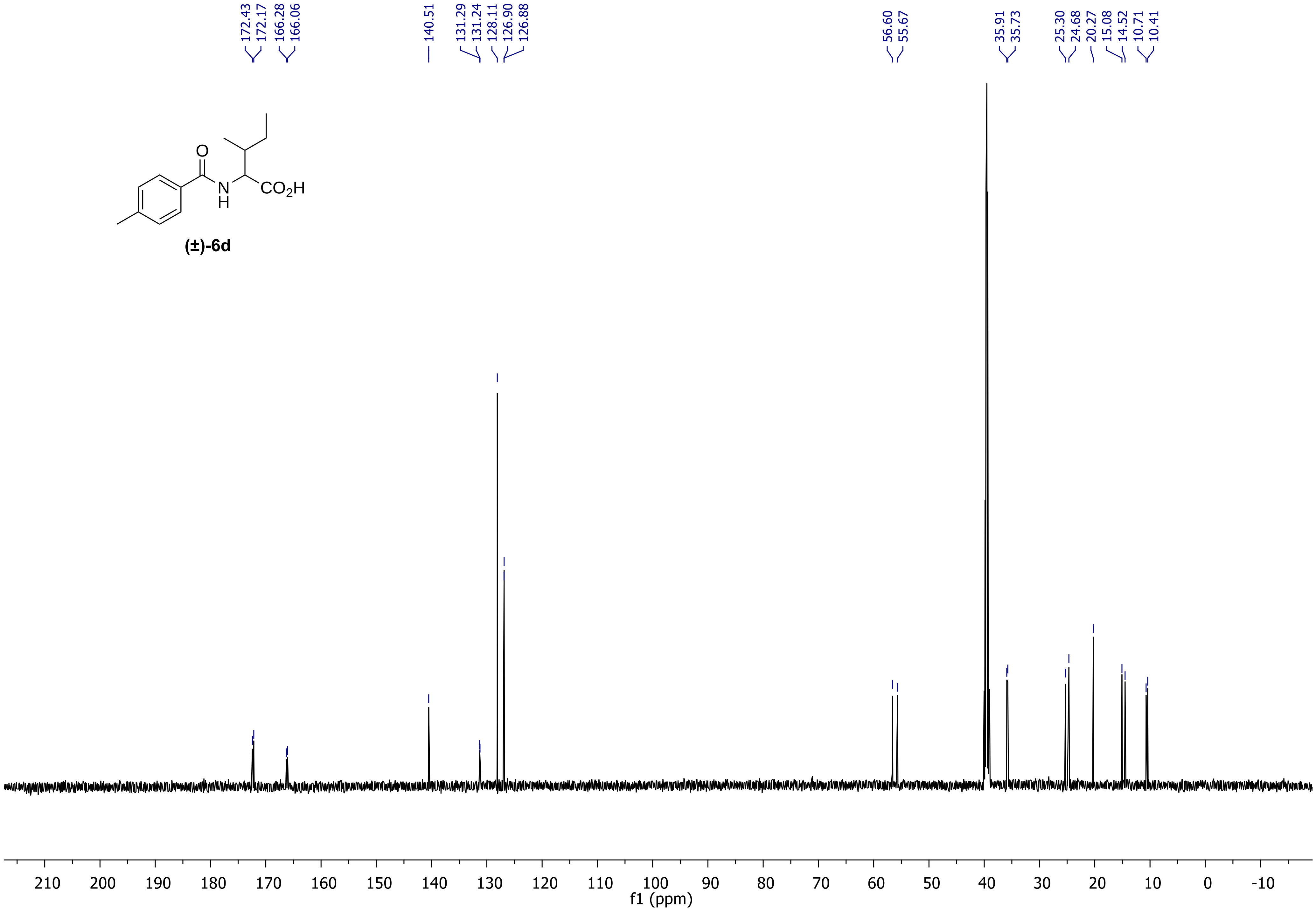

^{1}H NMR: 500 MHz, D6-DMSO, 100 °C

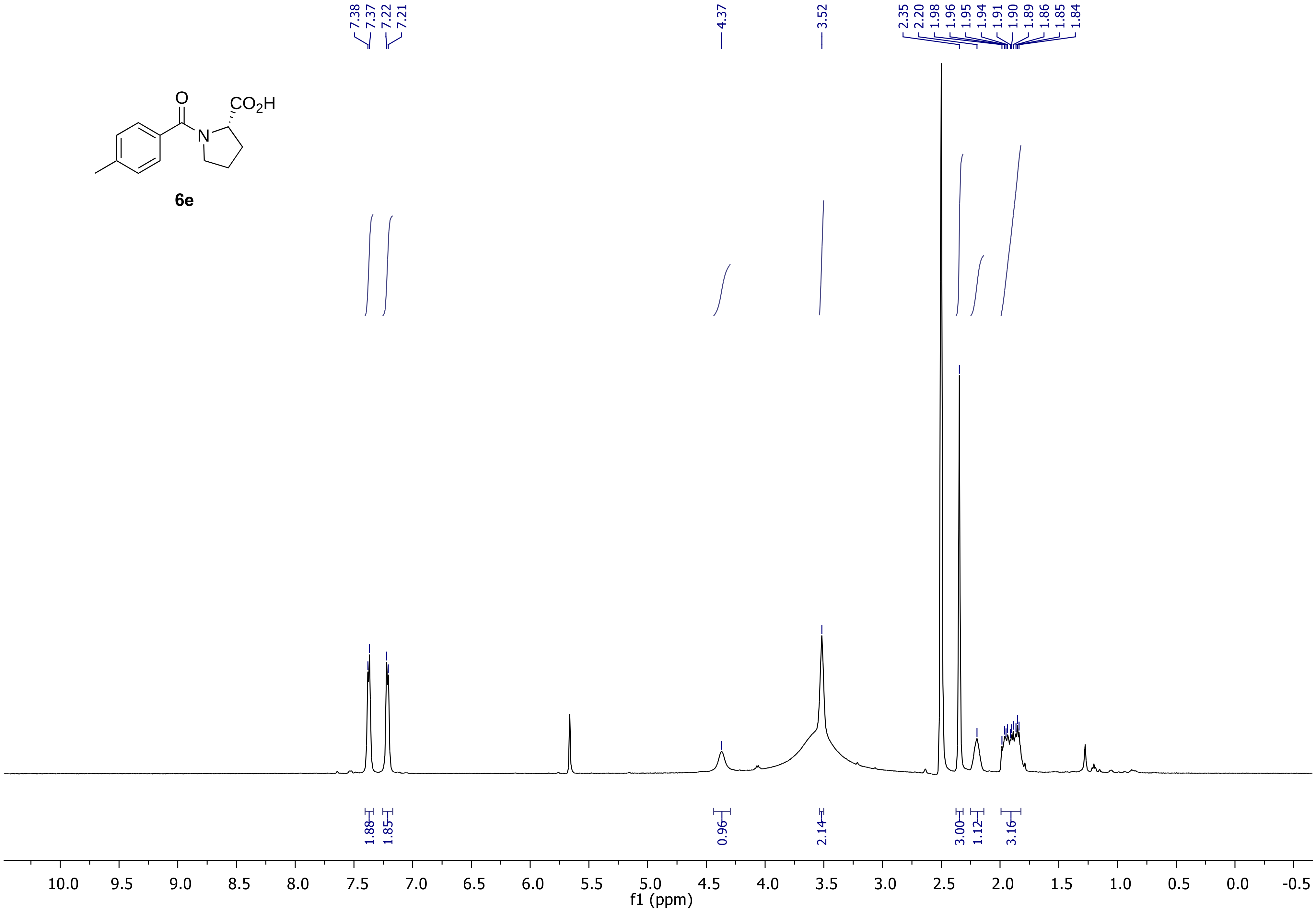

$^{13}C\{^1H\}$ NMR: 126 MHz, D_6-DMSO, 100 °C

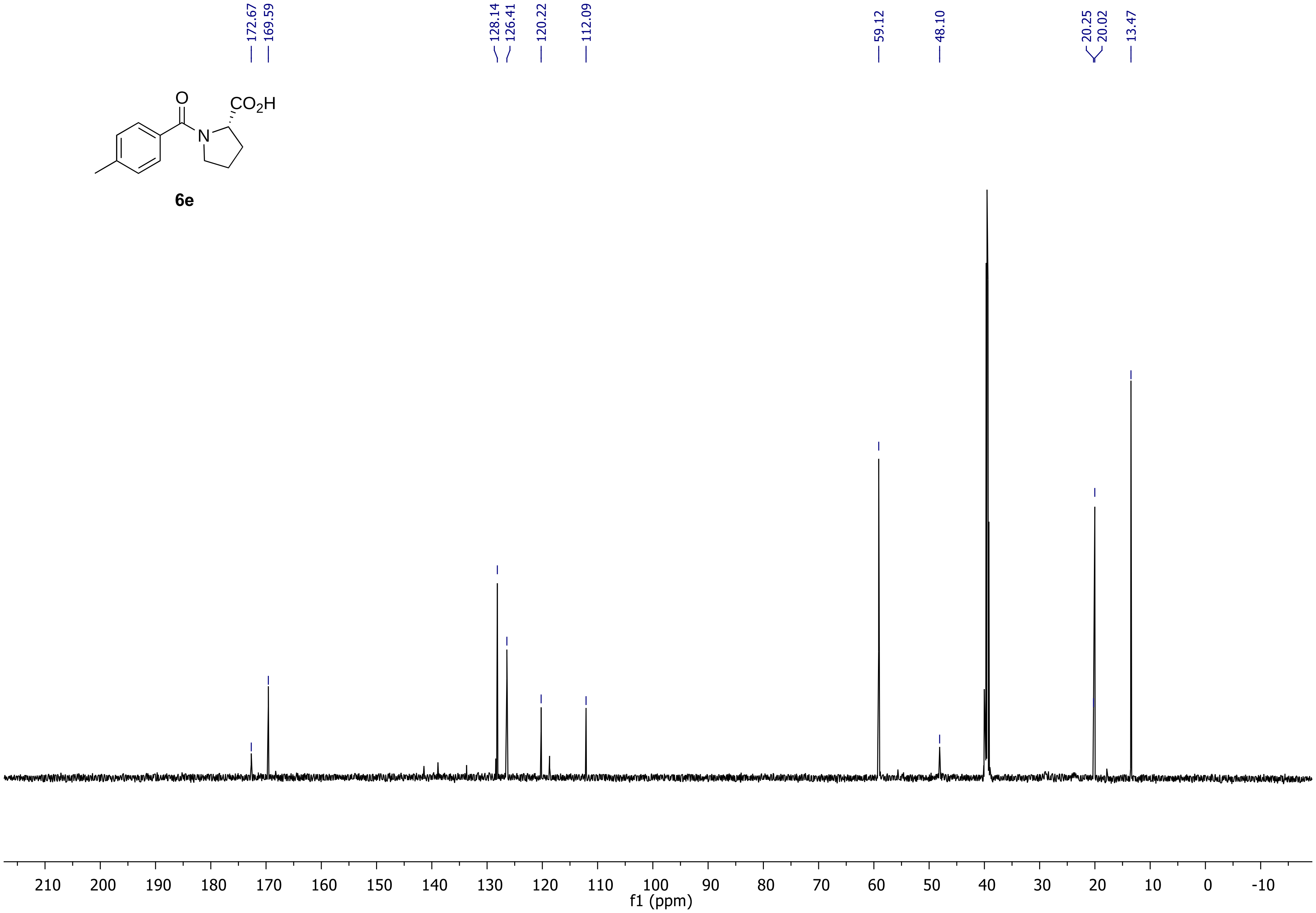

^{1}H NMR: 500 MHz, D6-DMSO

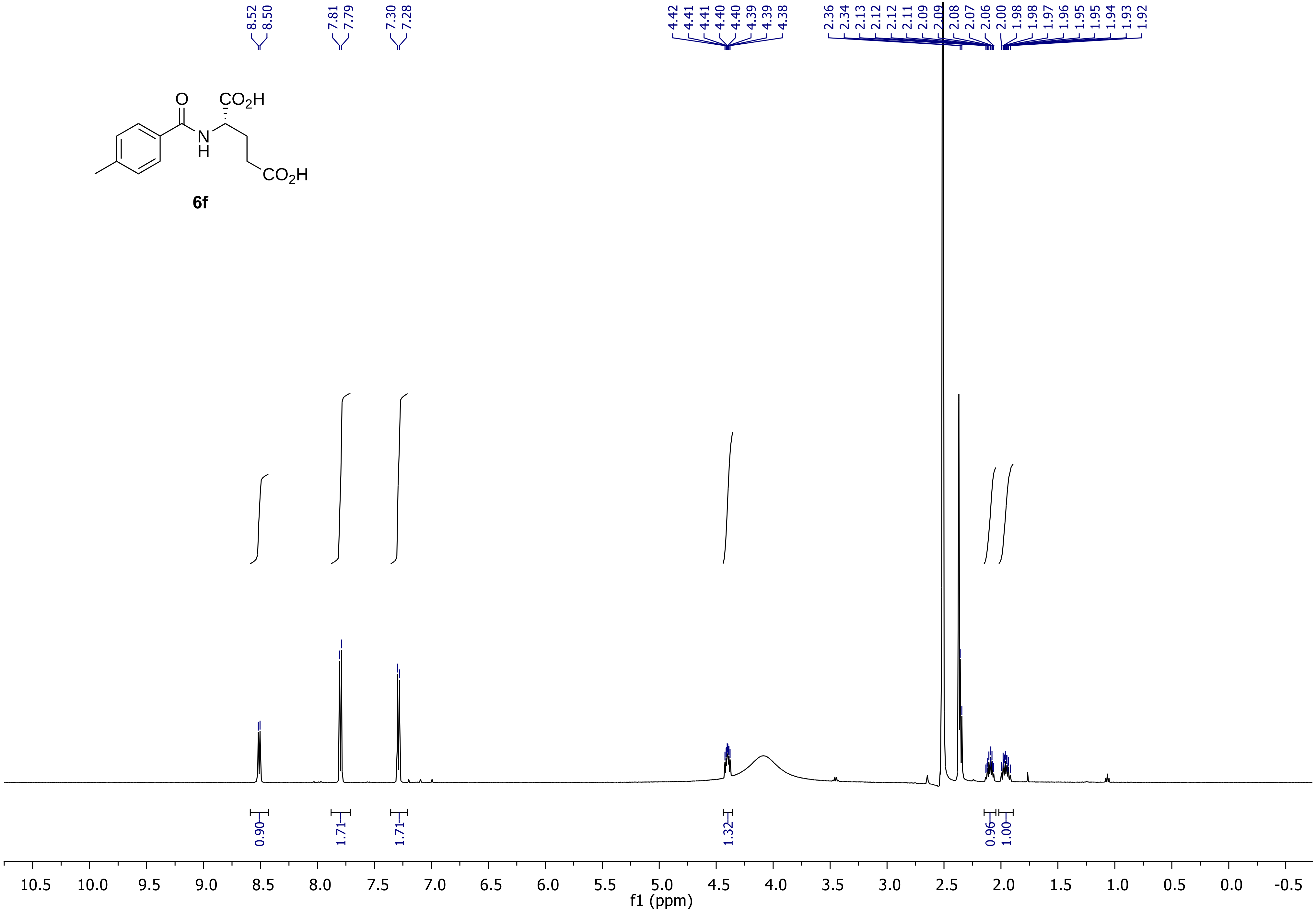

$^{13}C\{^{1}H\}$ NMR: 126 MHz, D_6-DMSO

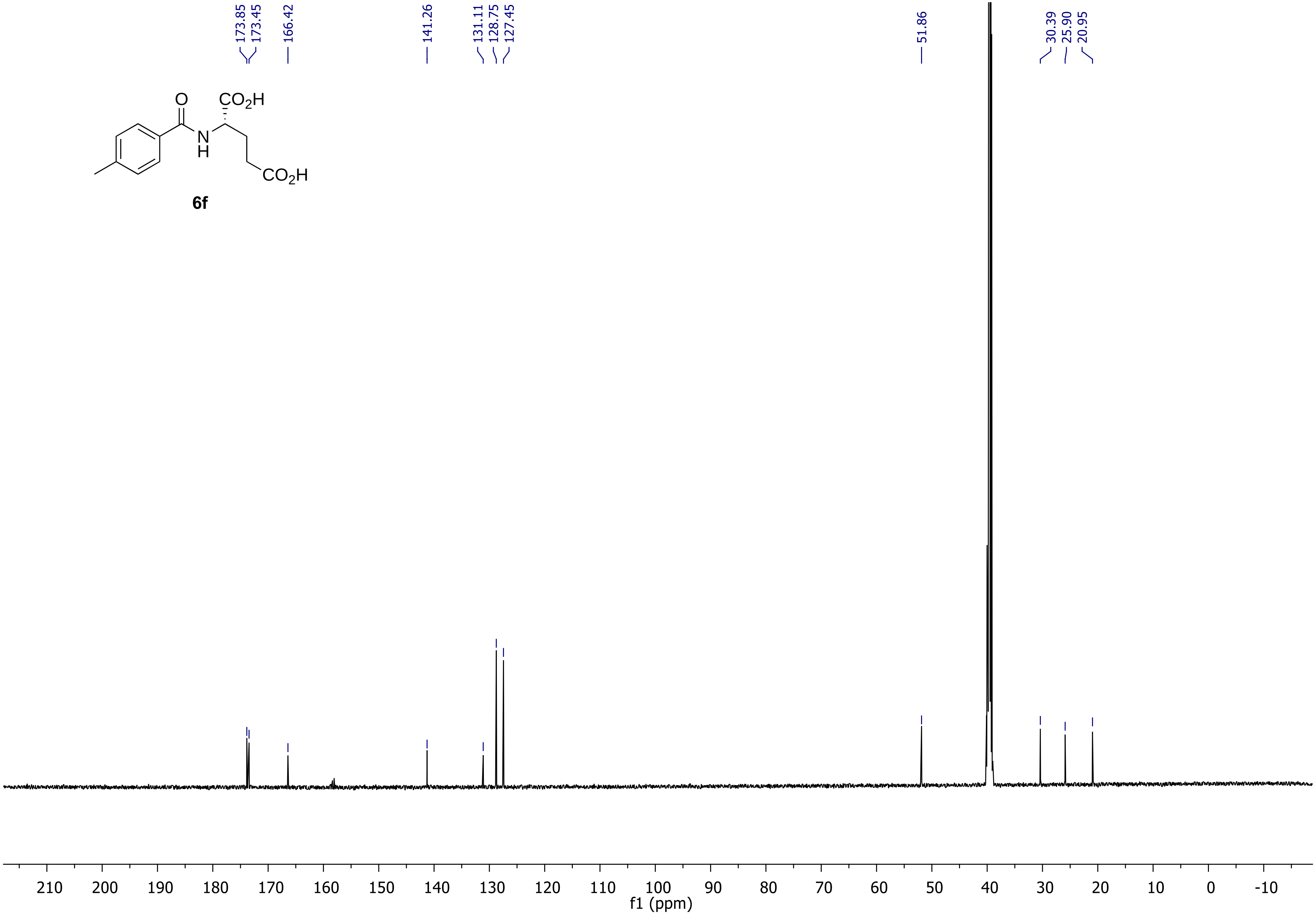

Supplement: Supplementary file 1 — ol1c03993_si_001.pdf [file ol1c03993_si_001.pdf]
